# Adduct-catalyzed tandem electro-thermal synthesis of organophosphorus (III) compounds from white phosphorus

**DOI:** 10.1093/nsr/nwaf008

**Published:** 2025-01-14

**Authors:** Jingcheng Hu, Haoyu He, Minghao Xu, Xiaotian Qi, Chao Fu, Hong Yi, Aiwen Lei

**Affiliations:** College of Chemistry and Molecular Sciences, The Institute for Advanced Studies (IAS), Wuhan University, Wuhan 430072, China; College of Chemistry and Molecular Sciences, The Institute for Advanced Studies (IAS), Wuhan University, Wuhan 430072, China; College of Chemistry and Molecular Sciences, The Institute for Advanced Studies (IAS), Wuhan University, Wuhan 430072, China; College of Chemistry and Molecular Sciences, The Institute for Advanced Studies (IAS), Wuhan University, Wuhan 430072, China; Department of Electrical Engineering, North China Electric Power University, Baoding 071003, China; College of Chemistry and Molecular Sciences, The Institute for Advanced Studies (IAS), Wuhan University, Wuhan 430072, China; College of Chemistry and Molecular Sciences, The Institute for Advanced Studies (IAS), Wuhan University, Wuhan 430072, China

**Keywords:** organic electrosynthesis, P_4_ activation, trivalent organophosphorus, adduct-catalyzed, green electricity

## Abstract

Electrooxidation strategies for synthesizing readily oxidizable products face notable challenges, especially when the oxidation potential of the products is lower than that of the reactants or when high current densities are necessary. The electrooxidation synthesis of trivalent organophosphorus compounds (OPCs (III)) from white phosphorus (P_4_) has demonstrated potential but is hindered by selectivity issues due to over-oxidation. Herein, we report a tandem electro-thermal synthesis pathway that addresses these challenges in producing OPCs (III) from P_4_. The process begins with an electrooxidation step that generates a stable trivalent phosphorus transfer reagent, then thermochemically converted into various high-value OPCs (III). Utilizing hexafluoroisopropanol (HFIP) as the nucleophile and optimizing a tetrabutylammonium iodide (TBAI)–4-dimethylaminopyridine (DMAP)-adduct catalytic system, we developed an efficient electrophilic phosphorus transfer reagent via electrosynthesis. The adduct facilitates the oxidation of P_4_ and enhances the nucleophilicity of HFIP, thereby improving the electrooxidation process. This approach supports high current density, scales up to the hundred-gram level without yield loss, and remains compatible with fluctuating green electricity.

## Teaser

This study leverages electrosynthesis strategy to facilitate the sustainable production of diverse trivalent phosphorus compounds. This innovative approach provides a promising avenue for the industrial upgrading of the phosphorus chemical industry.

## INTRODUCTION

Electrooxidation synthesis offers a sustainable approach to synthesizing readily oxidizable products by utilizing renewable electrical energy instead of stoichiometric oxidants, thereby minimizing waste [1–5]. However, synthesizing easily oxidizable products poses significant challenges, especially when their oxidation potentials are lower than that of the reactants [6–8]. A key issue is the tendency for over-oxidation of the target molecule, complicating the achievement of high yields. This challenge is further amplified under high current density conditions, typically associated with increased oxidation overpotentials, creating harsher oxidative environments [9]. Despite these difficulties, high current densities are crucial for enhancing the practicality and industrial viability of electrochemical synthesis methods [10,11]. Therefore, achieving the synthesis of readily oxidizable molecules at high current densities remains a critical challenge.

**Figure 1.**
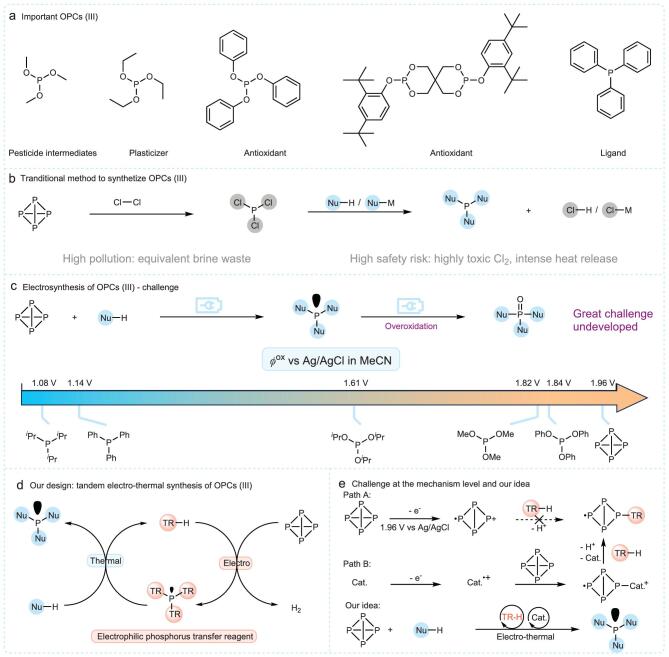
Importance of OPCs (III), industrial production of OPCs (III), electrosynthesis of OCPs (III) and our design. (a) Importance of OPCs (III) in industry and organic synthesis; (b) industrial production of OPCs (III) and its problem; (c) challenge in electrosynthesis of OPCs (III); (d) our design: tandem electro-thermal synthesis of OPCs (III). (e) Challenge at the mechanism level and our idea.

Trivalent organophosphorus compounds (OPCs (III)) hold significant industrial importance due to their role as antioxidants, owing to their reducibility. They are also essential in the production of pesticides, plasticizers, and ligands, making them vital to both the chemical industry and fundamental research (Fig. 1a) [12]. Despite their importance, OPCs (III) production is tightly regulated due to the pollution and high risks associated with the traditional chlorination process. Additionally, the synthesis involves the use of extremely dangerous and highly toxic chemical reagents (Fig. 1b) [13,14]. To address these issues, researchers have explored the synthesis of OPCs (III) from P_4_ [15–25]. Recently, Lennert *et al*. [26] and Chen *et al*. [27] have successfully employed photocatalytic strategies to activate P_4_ through reduction, generating free radicals, and synthesizing triarylphosphines and dialkyl phosphonates. Beyond direct synthesis, advancements have been made in generating OPCs (III) via phosphorus transfer reagents derived from P_4_ [19,28]. The Weigand and Wolf groups reported the synthesis of (SnBu_3_)*_x_*PH_(3−_*_x_*_)_ and [P(DMAP)_3_]^3+^, both of which are effective phosphorus transfer reagents capable of reacting with electrophiles or nucleophiles to produce OPCs (III) [29,30]. This method offers the advantage of enabling the green synthesis of various OPCs (III) through the development of a single phosphorus transfer reagent, thereby providing broader applicability.

Electrochemical methods [31–36] for synthesizing OPCs from P_4_ offer a promising solution to pollution and safety concerns. However, electrochemical oxidation of P_4_ to OPCs is rarely reported [37–41]. Among these studies, only a few OPCs (III), including diethyl phosphite and three triaryl phosphites, have been synthesized at extremely low current densities with poor selectivity. Consequently, the prospects for industrial applications are limited. The main challenge lies in the fact that OPCs (III) have lower oxidation potentials than P_4_, leading to overoxidation during electrolysis (Fig. 1c). As an illustration, trialkylphosphines and triarylphosphines, where carbon is directly bonded to phosphorus, have oxidation potentials around 1.1 V—significantly lower than P_4_’s 1.96 V—rendering their direct electrooxidation synthesis from P_4_ nearly impossible. Similarly, trialkoxyphosphines, with oxygen bonded to phosphorus, have oxidation potentials ∼1.8 V, also lower than that of P_4_, posing substantial selectivity challenges. Considering the distinct oxidation potentials of trialkyl phosphines and trialkoxyphosphines, we propose that the inductive effect of oxygen atoms attached to the phosphorus center plays a crucial role in enhancing the oxidation potential of OPCs (III). Therefore, using more electron-deficient and weakly nucleophilic reagents to bond with phosphorus could facilitate the synthesis of OPCs (III) with elevated oxidation potentials.

To synthesize diverse electron-rich trivalent phosphorus compounds, we propose a tandem electro-thermal approach. This approach initiates with the electrooxidation of P_4_ and an electron-deficient, weakly nucleophilic reagent (employed as a transfer reagent for cyclical reuse, designated as TR-H) to produce electrophilic P(TR)_3_. The resulting P(TR)_3_, exhibiting greater resistance to oxidation than P_4_, functions as a versatile phosphorus transfer reagent, analogous to PCl_3_, and is subsequently converted thermochemically into various high-value trivalent phosphorus compounds, with TR-H being regenerated in the process (Fig. 1d). Direct electrooxidation of P_4_ to form P(TR)_3_ poses challenges due to the difficulty in oxidizing P_4_ and the weak nucleophile TR-H reacting with the P_4_ radical cation intermediate (Fig. 1e, Path A). Therefore, a catalytic system is essential, where the catalyst induces P_4_ oxidation to form an active intermediate, promoting its reaction with the weak nucleophile (Fig. 1e, Path B).

In this work, we identified hexafluoroisopropanol (HFIP) as the optimal TR-H for efficiently generating the trivalent phosphorus reagent P[OCH(CF_3_)_2_]_3_, facilitating the synthesis of a broad range of OPCs (III) (51 examples). The multiple fluorine atoms in HFIP effectively disperse the charge on the phosphorus center, enhancing the reaction efficiency. To further improve this transformation, we developed an effective tetrabutylammonium iodide (TBAI)–4-dimethylaminopyridine (DMAP)-adduct catalytic system. This adduct not only promotes the electrooxidation of P_4_ but also enhances the nucleophilicity of HFIP, significantly boosting reaction efficiency. The process achieves a current density of 45 mA/cm² and scales up to the hundred-gram level, utilizing wind and solar energy to reduce carbon emissions.

## RESULT AND DISCUSSION

### Condition optimizations and control experiments

**Figure 2.**
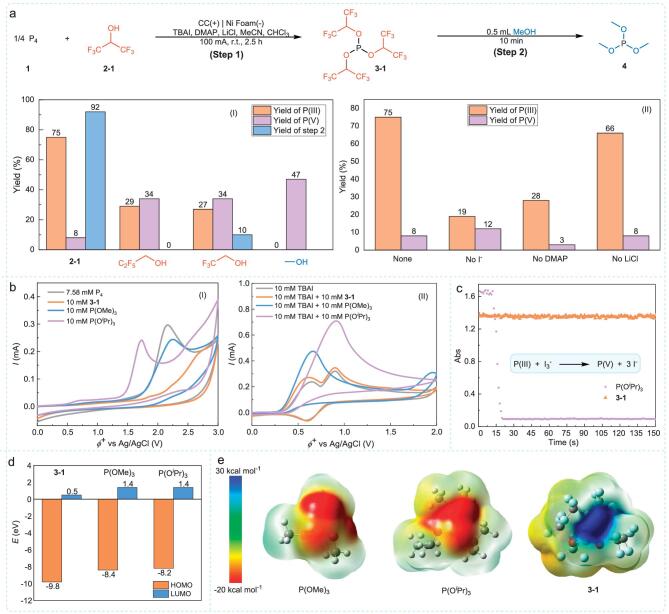
Control experiment, CV studies for the importance of the –CF_3_ group, UV-Vis studies for the importance of the –CF_3_ group, and theoretical calculation. (a) Control experiment: (I) the number of –CF_3_ group; (II) reaction condition. Standard condition: P_4_ (0.75 mmol), TBAI (0.6 mmol), LiCl (1.2 mmol), DMAP (3.0 mmol), HFIP (24 mmol), MeCN (4 mL), CHCl_3_ (3 mL) in an undivided cell with carbon cloth (2.25 cm^2^) as the anode and Ni foam (3 cm^2^) as the cathode, r.t., 100 mA, 2.5 h. The yields were determined by assured ^31^P NMR. (b) CV studies for the importance of the –CF_3_ group. Conditions: *^n^*Bu_4_NPF_6_ (0.05 M) was used as the supporting electrolyte, MeCN:CHCl_3 _= 4:1 as solvent, the scan rate was 10 mV/s, and all potentials are referenced against the Ag/AgCl reference electrode. (c) UV-Vis studies for the importance of the –CF_3_ group. (d and e) Theoretical calculation.

The search for a suitable TR-H led to the identification of HFIP as the optimal reagent, effectively introducing two –CF_3_ groups into the nucleophilic reagent, aligning with the design criteria (Fig. S4). The optimized reaction conditions utilized a mixed solvent of MeCN and CHCl_3_, with TBAI serving as the electrolyte and mediator, DMAP and LiCl as additives, carbon cloth (CC) as the anode, and nickel foam (Ni Foam) as the cathode (Fig. 2a, I, step 1). Under these conditions, a 75% yield of **3–1** and an 8% yield of **3–1′** (pentavalent phosphorus) were achieved at 100 mA (44.4 mA/cm²) over 2.5 hours (Table S1). Subsequent addition of methanol converted **3–1** into trimethyl phosphite (P(OMe)_3_) with a 92% yield (Fig. 2a, I, step 2), demonstrating that **3–1** can be effectively transformed into electron-rich OPCs (III), thus meeting the design objectives.

In comparison, using trifluoroethanol, which has one less –CF_3_ group, resulted in a 27% yield of the corresponding OPCs (III). When reacted with methanol, it produced only a 10% yield of P(OMe)_3_, indicating its inefficiency as a phosphorus transfer reagent. Using methanol, which has no –CF_3_ group, yielded 47% trimethyl phosphate, with no formation of OPCs (III), P(OMe)_3_ (Fig. 2a, I). These results highlight the importance of introducing two –CF_3_ groups. Control experiments demonstrated that the addition of DMAP and TBAI was crucial for improving the yield and selectivity. Omitting lithium chloride also decreased the yield (Fig. 2a, II).

### Investigation of the stability of 3-1

After synthesizing **3–1** and confirming its stability under electrooxidation conditions, we investigated the factors contributing to this stability. Cyclic voltammetry (CV) tests revealed that the oxidation potential of **3–1** was 2.35 V (Fig. 2b, I, orange line), higher than that of P_4_ (1.96 V, Fig. 2b, I, gray line). This indicates that P_4_ can be preferentially oxidized during the reaction, enabling selective control. To further validate the effect of introducing the –CF_3_ group, we compared the oxidation potential of **3–1** with that of P(OMe)_3_ (1.94 V, Fig. 2b, I, blue line) and triisopropyl phosphite (P(O*^i^*Pr)_3_) (1.57 V, Fig. 2b, I, purple line). The oxidation potential of **3–1** was 0.41 V higher than P(OMe)_3_ and 0.78 V higher than P(O*^i^*Pr)_3_, indicating that the stabilization is due to electronic effects rather than steric hindrance. Upon the addition of P(OMe)_3_, P(O*^i^*Pr)_3_, and **3–1** to the TBAI solution individually, only **3–1** failed to produce a noticeable catalytic current (Fig. 2b). This result indicates that **3–1** exhibits a higher resistance to oxidation by oxidized iodine species compared to P(OMe)_3_ and P(O*^i^*Pr)_3_. UV-Vis spectroscopy at −30°C further confirmed this: the absorption intensity of I_3_^−^ decayed rapidly with P(O*^i^*Pr)_3_ and remained almost unchanged with **3–1** (Fig. 2c). DFT calculations suggested that the introduction of –CF_3_ groups significantly lowers the highest occupied molecular orbital and lowest unoccupied molecular orbital energies, consistent with the observed increased resistance to oxidation (Fig. 2d). Electrostatic potential surface calculations showed that the introduction of –CF_3_ groups reverses the phosphorus center's character from nucleophilic to electrophilic (Fig. 2e), attributed to the strong electric field generated by the highly electronegative fluorine atoms dispersing electron density at the phosphorus center. This enhances its resistance to oxidation, making it an effective phosphorus transfer reagent.

### Mechanistic experiments

**Figure 3.**
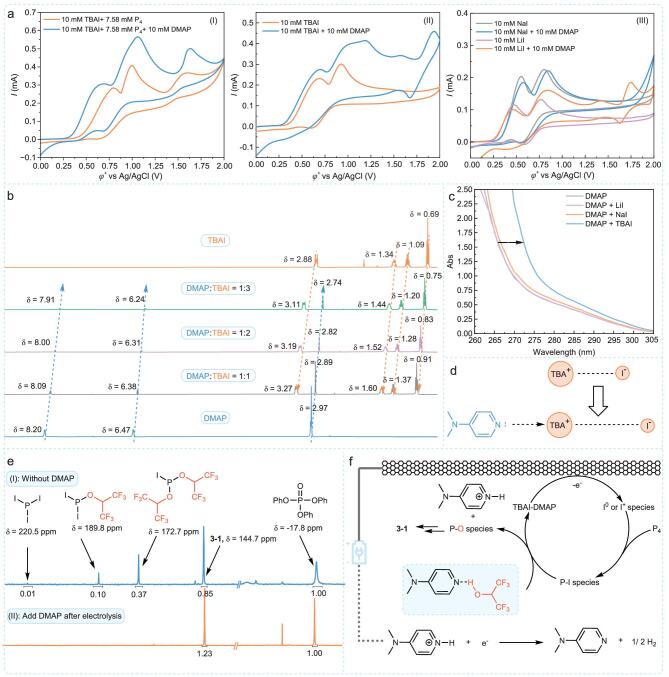
Mechanistic experiments. (a) CV studies for the importance of TBAI and DMAP. Conditions: *^n^*Bu_4_NPF_6_ (0.05 M) was used as the supporting electrolyte, MeCN:CHCl_3 _= 4:1 as solvent, the scan rate was 10 mV/s, and all potentials are referenced against the Ag/AgCl reference electrode. (b) ^1^H NMR test. Conditions: *^n^*Bu_4_NPF_6_ (0.05 M) was used as the supporting electrolyte, MeCN:CHCl_3 _= 4:1 as solvent, the scan rate was 10 mV/s, and all potentials are referenced against the Ag/AgCl reference electrode. (c) UV-Vis studies for the interaction between DMAP and TBAI, NaI or LiI. (d) Interaction between DMAP and TBAI. (e) Control experiment and ^31^P NMR study. (f) Possible mechanisms.

To gain insights into the mechanism of the electrochemical synthesis of **3–1**, we conducted a series of mechanistic experiments. First, we employed CV to demonstrate that iodide ions function as mediators in the activation of white phosphorus, corroborating existing reports (see Figs S24 and S25 for details) [38,42,43]. The absence of DMAP significantly reduced the yield (Fig. 2a, II), prompting us to investigate its importance. Adding DMAP to a solution of iodine and P_4_ greatly increased the peak current of iodide oxidation and advanced the onset potential of I^−^ from 0.46 V to 0.37 V (Fig. 3a, I). This indicates that DMAP may facilitate iodide oxidation, promoting P_4_ oxidation. To confirm this, we assessed the behavior of DMAP without P_4_ and observed an increase in peak current and a shift in onset potential from 0.38 V to 0.32 V (Fig. 3a, II). We also examined different pyridines and found that they consistently increased peak current and advanced onset potential (Fig. S27). More electron-rich pyridines caused greater potential shifts. These results suggest that pyridine and TBAI form an adduct with a lower oxidation potential and higher peak current. Additionally, the onset potential for iodide oxidation did not shift when DMAP was mixed with lithium iodide (LiI) or sodium iodide (NaI) (Fig. 3a, III). These findings imply that TBA⁺ is essential for the formation of the adduct.

Further ¹H NMR experiments revealed that increasing TBAI concentration caused a downfield shift in DMAP's proton peaks (Fig. 3b, blue arrow), while increasing DMAP concentration led to an upfield shift in TBA^+^’s proton peaks (Fig. 3b, orange arrow). However, no proton chemical shift changes were detected in the presence of NaI or LiI (Figs S37 and S38). The interaction between DMAP and TBAI was further corroborated by UV-Vis spectroscopy. The addition of TBAI resulted in a noticeable red shift in the absorption peak of DMAP, indicating the formation of a TBAI-DMAP–adduct. In contrast, no red shift was observed with NaI or LiI, suggesting that TBA^+^ is essential for this adduct formation (Fig. 3c). We hypothesize that this interaction weakens the electrostatic binding of TBA^+^ to I^−^, facilitating the oxidation of I^−^. Consequently, this adduct is more easily oxidized compared to TBAI (Fig. 3d, details provided in Fig. S32). Comparisons of anodic oxidation potentials revealed a significantly higher oxidation potential in the absence of DMAP (Fig. S39), further confirming that the adduct is more easily oxidized than TBAI.

To further clarify DMAP's role in the reaction, we conducted ³¹P NMR spectroscopy to analyze the products and by-products formed in the absence of DMAP. Without DMAP under standard conditions, the yield of the target product was only 28%, accompanied by 1% PI_3_ and 15% PI*_X_*[OCH(CF_3_)_2_]_(3−_*_X_*_)_ (*X* = 1, 2) by-products (Fig. 3e, I). Adding DMAP after the reaction fully converted these by-products, raising the product yield to 41% (Fig. 3e, II). This indicates that DMAP enhances the nucleophilicity of HFIP, promoting its reaction with P–I species to form P–O species. ¹H NMR experiments revealed that increasing the DMAP ratio caused the –OH hydrogen of HFIP to shift downfield (Fig. S34), suggesting hydrogen bond formation between DMAP and HFIP. This interaction enhances oxygen's nucleophilicity, facilitating the alcoholysis of P–I bonds. Based on these findings, TBAI-DMAP–adduct is likely oxidized at the anode to form oxidized iodine species. These species activate P_4_ to form P–I species, which react with the HFIP-DMAP hydrogen-bond complex to form P–O species, leading to the final product, **3–1** (Fig. 3f).

### Reaction scale-up

**Figure 4.**
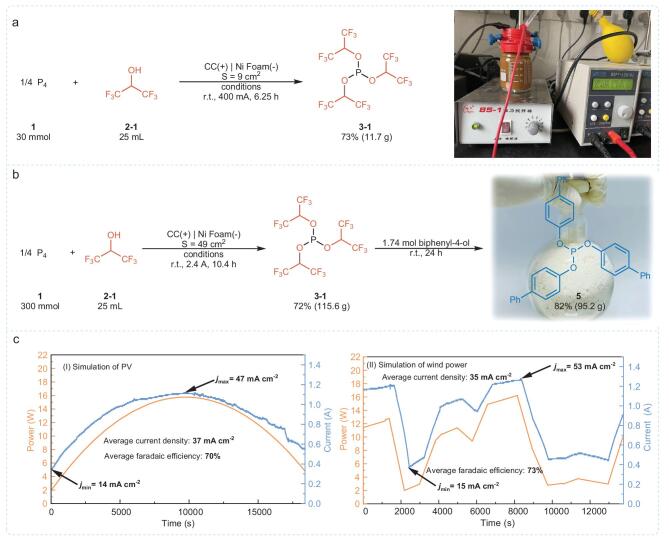
Reaction scale-up. (a) 10-gram scale. (b) 100-gram scale. (c) Scale-up using photovoltaic (PV) and wind power.

To evaluate the potential applications of this strategy, we scaled up the electrosynthesis process. By increasing the electrode surface area, we initially synthesized 11.3 g of compound **3–1** with a 73% yield over 6.25 hours (Fig. 4a). Further scaling, we expanded the electrode area to 49 cm² and increased the current to 2.4 A (49 mA/cm²). After 10.4 hours, we achieved a 72% yield, producing 115.6 g of **3–1**. Following the reaction, we added biphenyl-4-ol and stirred at room temperature for 24 hours, synthesizing 95.2 g of tri([1,1′-biphenyl]-4-yl) phosphite via a one-pot two-step method (Fig. 4b). Scaling the reaction by 100-fold did not significantly impact the yield, underscoring the scalability and practical potential of this reaction. We subsequently recovered the solvent and HFIP via distillation, demonstrating that the reclaimed solvent was suitable for reuse (Fig. S16).

While electrosynthesis is green and safe, using electricity from fossil fuels leads to significant carbon emissions [44]. Therefore, using green electricity from renewable sources like wind and solar power is more environmentally friendly [45]. However, renewable energy output is variable and depends on environmental conditions [46,47], requiring costly energy storage facilities. We aimed to utilize fluctuating green electricity for electrosynthesis by developing a system to control the power output, simulating the variable nature of solar and wind power. First, we simulated the power variation of photovoltaic electricity, gradually increasing from 2 W to 15.75 W and then slowly decreasing to 4.8 W, corresponding to current densities rising from 14 mA/cm² to 47 mA/cm² and then falling to 17 mA/cm². Under these fluctuating power conditions, we achieved a Faradaic efficiency of 70% for **3–1** (Fig. 4c, I). Next, we simulated the more volatile output of wind power, with a maximum output of 16.2 W and a minimum of 2 W, corresponding to maximum and minimum current densities of 53 mA/cm² and 15 mA/cm², respectively. The Faradaic efficiency under these conditions was 73% (Fig. 4c, II). These results demonstrate that this strategy can effectively utilize fluctuating green electricity.

### Substrate scope

We further evaluated the applicability of **3–1** as a phosphorus transfer reagent (Fig. 5). First, **3–1** was isolated from the 10-gram scale reaction by vacuum distillation (Fig. S14). We first assessed the functional group compatibility of alcohols. As the chain length of alcohols increased, the reaction yield remained consistent (**4**, **6–9**). Alcohols containing halogens, alkenyl, alkynyl, cyano, ester, or phenyl groups yielded trialkyl phosphites with moderate to excellent yields (**10–14**, **16**). When the α-position of the alcohol contained a bulky cyclohexyl group, the product was obtained in 89% yield (**15**). Next, we examined secondary alcohols with larger steric hindrance, which typically resulted in a mixture of trialkyl phosphites and dialkyl phosphites due to steric effects. To obtain a single product, we added water and acid after the reaction to convert the trialkyl phosphites into dialkyl phosphites. Both linear and cyclic secondary alcohols produced dialkyl phosphites with excellent yields (**18–22**). For 1,3-dichloropropan-2-ol, we selectively obtained the trialkyl phosphite in 82% yield (**17**). Even with highly hindered tert-butanol, we successfully obtained the target product (**23**). Expanding the scope to phenols, both phenol and 4-substituted phenols converted to the target products with high yields (**24–27**, **5**). 1-Naphthol was also compatible with this process (**28**). Additionally, 2,4- or 2,3-disubstituted phenols smoothly produced the desired products (**29**, **30**).

**Figure:**
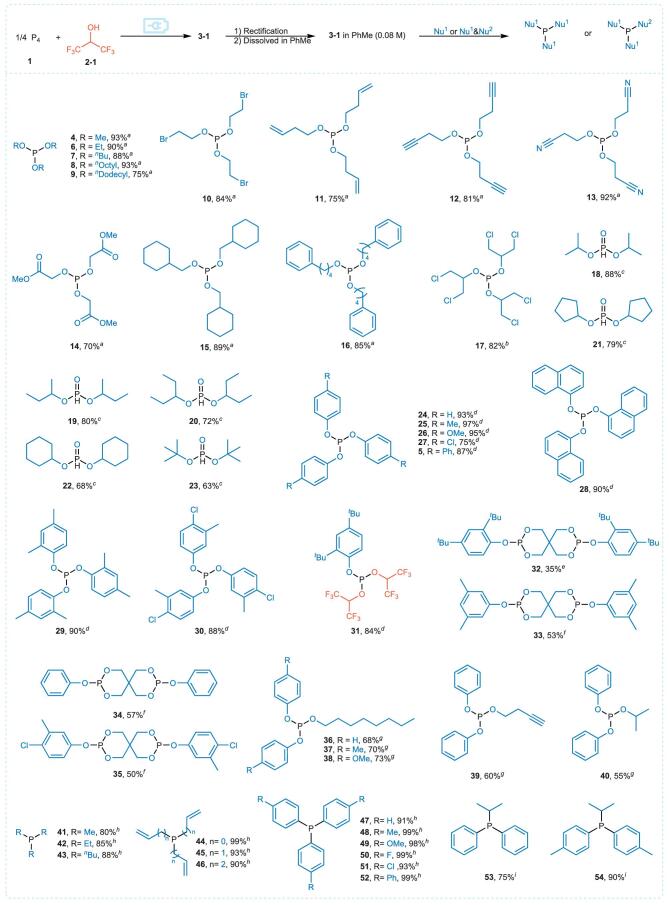
Substrate scope. Method a: **3–1** (0.2 mmol), primary alcohol (1.0 mmol), K_2_CO_3_ (0.3 mmol), PhMe (2.5 mL), r.t., 2 h. Method b: **3–1** (0.2 mmol), secondary alcohol (1.0 mmol), K_2_CO_3_ (0.3 mmol), PhMe (2.5 mL), 40°C, 4 h. Method c: **3–1** (0.2 mmol), secondary or tertiary alcohol (1.0 mmol), K_2_CO_3_ (0.3 mmol), PhMe (2.5 mL), 40°C, 12 h, then HOAc (0.2 mmol), H_2_O (0.2 mL) was added and stirred for 30 min. Method d: **3–1** (0.2 mmol), phenol (1.0 mmol), K_2_CO_3_ (0.3 mmol), PhMe (2.5 mL), 40°C, 2 h. Method e: **3–1** (0.4 mmol), pentaerythritol (0.2 mmol), K_2_CO_3_ (0.3 mmol), PhMe (5.0 mL), 80°C, 6 h, then phenol (1.0 mmol) was added, 120°C, 12 h. Method f: the second step was 40°C, 4 h. Method g: **3–1** (0.2 mmol), alcohol (0.2 mmol), K_2_CO_3_ (0.3 mmol), PhMe (2.5 mL), 40°C, 4 h, then phenol (0.8 mmol) was added, 40°C, 2 h. Method h: **3–1** (0.2 mmol), Grignard reagent (1.0 mmol), DMAP (0.2 mmol), PhMe (2.5 mL), r.t., 2 h. Method i: **3–1** (0.2 mmol), Grignard reagent (0.4 mmol), DMAP (0.2 mmol), PhMe (2.5 mL), r.t., 2 h, then another Grignard reagent (1.0 mmol) was added, r.t., 1 h.

When using 2,4-di-*tert*-butylphenol as the nucleophile, the reaction halted at the mono-substituted intermediate (**31**), likely due to the significant steric hindrance of the *tert*-butyl groups. This prompted us to synthesize organophosphorus with two different substituents, which are an important class of OPCs (III). Through optimization of the reaction conditions, we achieved the successful synthesis of **32**, an antioxidant of industrial significance, with a yield of 35% (Fig. S15). Using this strategy, we successfully synthesized similar OPCs (III) compounds (**33–35**) with moderate to high yields using pentaerythritol and different phenols as nucleophiles. Further, replacing pentaerythritol with linear monohydric alcohols also afforded the corresponding target products (**36–40**) with moderate yields. We then extended the nucleophiles to carbon nucleophiles. Using toluene (PhMe) as solvent, Grignard reagent as nucleophilic reagent, we obtained trialkylphosphines, trialkenylphosphines, and triarylphosphines (**41–52**) in high yields, all of which are important ligands in organic synthesis. Additionally, we synthesized OPCs (III) containing both alkyl and aryl groups (**53, 54**) via a one-pot two-step method.

## CONCLUSION

In conclusion, the adduct-catalyzed tandem electro-thermal synthesis strategy developed here effectively enables the production of various OPCs (III), broadening the scope of P_4_ electrooxidation. This method operates at high current densities, scales up to the hundred-gram level, and adapts well to fluctuating green electricity sources. The field effect enhanced the stability of P[OCH(CF_3_)_2_]_3_ during the electrooxidation process while also improving its effectiveness as a phosphorus transfer reagent. The TBAI-DMAP–adduct plays a crucial role in addressing the challenges of inefficient P_4_ electrooxidation and the reactivity of intermediates with weak nucleophiles. Overall, this approach not only advances the scalable synthesis of OPCs (III) but also promotes sustainability by integrating renewable energy sources.

## METHODS

Full experimental details and characterization of the compounds are given in the Supplementary data.

*Electrochemical functionalization of P_4_ to access* ***3–1***: *^n^*Bu_4_NI (221.6 mg, 0.6 mmol) and DMAP (366.5 mg, 3.0 mmol) were added into the tube. The tube was equipped with carbon cloth (1.5 × 1.5 cm^2^) as the anode and Ni foam (2.0 × 1.5 cm^2^) as the cathode. This was followed by the addition of P_4_ (93 mg, 0,75 mmol), LiCl (50.9 mg, 1.2 mmol) in a glovebox (H_2_O and O_2_ <0.1 ppm). Then MeCN (4 mL), CHCl_3_ (3 mL) and HFIP (2.5 mL, 24 mmol) were added to the tube through a syringe. The mixtures were stirred at a constant current of 100 mA at room temperature for 2.5 h (J = 44.4 mA/cm^2^, 3 F/mol).

## Supplementary Material

nwaf008_Supporting_Information
