# Supplementary material for: Adduct-catalyzed tandem electro-thermal synthesis of organophosphorus (III) compounds from white phosphorus
Source: Natl Sci Rev. 2025 Jan 14;12(3):nwaf008. doi: 10.1093/nsr/nwaf008 (PMC11879454; doi:10.1093/nsr/nwaf008)
Supplement: nwaf008_Supporting_Information [file nwaf008_Supporting_Information.pdf]

Supporting Information

Adduct-Catalyzed Tandem Electro-thermal Synthesis of Organophosphorus (III) Compounds from White Phosphorus

Jingcheng Hu[1 ξ], Haoyu He[1 ξ], Minghao Xu[1], Xiaotian Qi[1], Chao Fu[2], Hong Yi[1]*, Aiwen Lei[1]*

[1] College of Chemistry and Molecular Sciences; The Institute for Advanced Studies (IAS), Wuhan University; Wuhan, 430072, P. R. China

[2] Department of Electrical Engineering, North China Electric Power University, Baoding, 071003, P.R. China

[ξ] These authors contributed equally: Jingcheng Hu, Haoyu He.

* Aiwen Lei. aiwenlei@whu.edu.cn, hong.yi@whu.edu.cn.

Content

General information 2

Experimental procedures for the assure ^{31}P NMR 3

Experimental procedures for the electrolysis 5

Experimental procedures for gram-scale reaction 7

Experimental procedures for the reaction scale-up with PV and wind power 8

Experimental procedures for 100 gram-scale reaction 10

Experimental procedures for the separation of **3-1** 11

Experimental procedures for converting **3-1** to OPC (III) 12

Substrate expansion for **3-1** 15

Experimental procedures for the recycling of HFIP 15

Experimental procedures for the UV-Vis Spectroscopy Study 17

Experimental Procedures for Cyclic Voltammetry 19

Density Functional Theory (DFT) Studies 26

Experimental procedures for the NMR tests 40

Experimental Procedures for Anode potential monitoring 43

Detailed Descriptions for Products 44

References 57
Copies of product NMR spectra 59

General information

All glassware was oven dried at 110 °C for hours and cooled down under vacuum. The instrument for electrolysis was Current (HSPY-120-01) (made in China). Chronoamperometry and cyclic voltammograms were obtained on a CorrTest® CS2350H bipotentiostat. Hydrogen gas content was analyzed by gas chromatography (GC9790 Plus, Fuli, China, TCD, N_2 as a carrier gas and 5 Å molecular sieve column, a thermal conductivity detector). Molecular weights of products were determined by SHIMADZU™ GCMS-QP2010 SE gas chromatgraphy mass spectrometrometry. All new compounds were characterized by High resolution mass spectra (HRMS). All undivided cells were purchased from Jiehengda® limited liability company (https://www.whjiehengda.com). The anodic electrode was carbon cloth (S= 2.25 cm^2) and cathodic electrode was Ni foam (S= 3 cm^2) unless otherwise noted. Unless otherwise noted, materials and solvents were obtained from commercial suppliers and used without further purification. Thin layer chromatography (TLC) employed glass 0.25 mm silica gel plates. Visualization of spots on TLC plate was accomplished with iodine cylinder or UV light (254 nm). Flash chromatography columns were packed with 200-300 silica gel in DCM. ^{1}H, ^{13}C, ^{31}P and ^{19}F NMR datas were recorded with Bruker Advance III (400 MHz) spectrometers with tetramethylsilane as an internal standard. High resolution mass spectra (HRMS) were measured with a Waters Micromass GCT instrument. All chemical shifts (δ) are reported in ppm and coupling constants (*J*) in Hz. All chemical shifts are reported relative to tetramethylsilane and d-solvent peaks (77.00 ppm, chloroform), respectively.

$$Faradiac\ efficiency\ \% = \frac{n\ (mmol) * Z * 96500}{I\ (mA) * T\ (s)} * 100\%$$

n is the amount of product obtained in the reaction

Z is the electron transfer number of the reaction

Eq.1. Calculation of Faradaic efficiency

Experimental procedures for the assure ^{31}P NMR

Experimental procedure for standard curve of the assure ^{31}P NMR

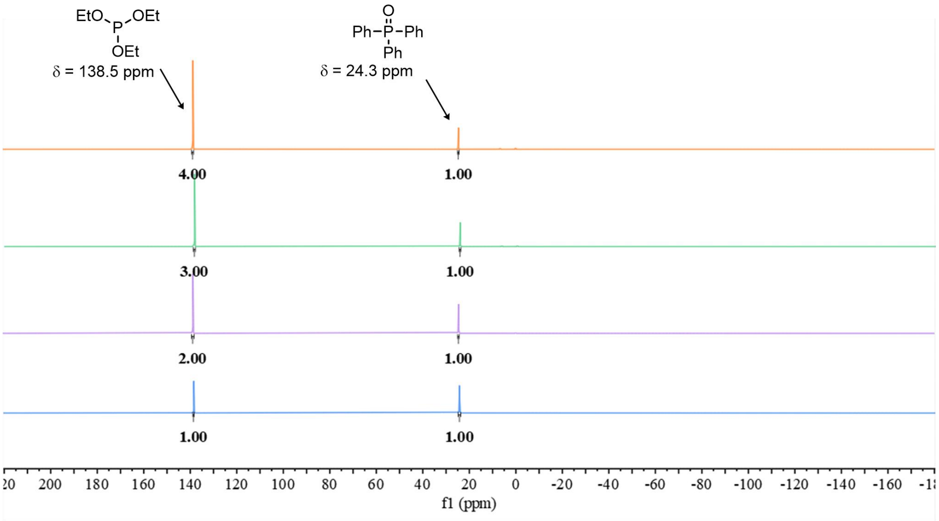

Fig. S1. Assure ^{31}P NMR for different concentration of triethyl phosphite

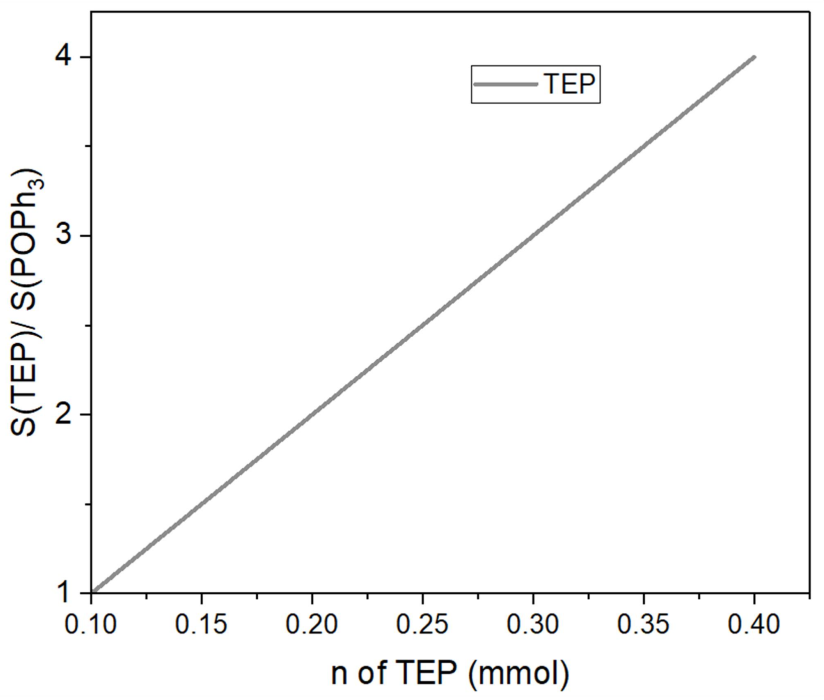

Fig. S2. Standard curve for triethyl phosphite

The triphenylphosphorus oxide (27.8 mg, 0.1 mmol) was dissolved in PhMe (1 mL) in an NMR tube as an internal standard. then different equivalents of triethyl phosphite was added into the NMR tube, and made assure ^{31}P NMR test. 1) 1 equiv. triethyl phosphite (166.2 mg, 0.1 mmol). 2) 2 equiv. triethyl phosphite (332.3 mg, 0.2 mmol). 3) 3 equiv. triethyl phosphite (498.5 mg, 0.3 mmol). 4) 4 equiv. triethyl phosphite (664.6 mg, 0.4 mmol).

Assured ^{31}P NMR Method:

Relaxation delay (D1): 25s, Number of dummy scans: 4, Numbers of scans: 8

Measurement of ^{31}P Longitudinal Relaxation Times (T1):

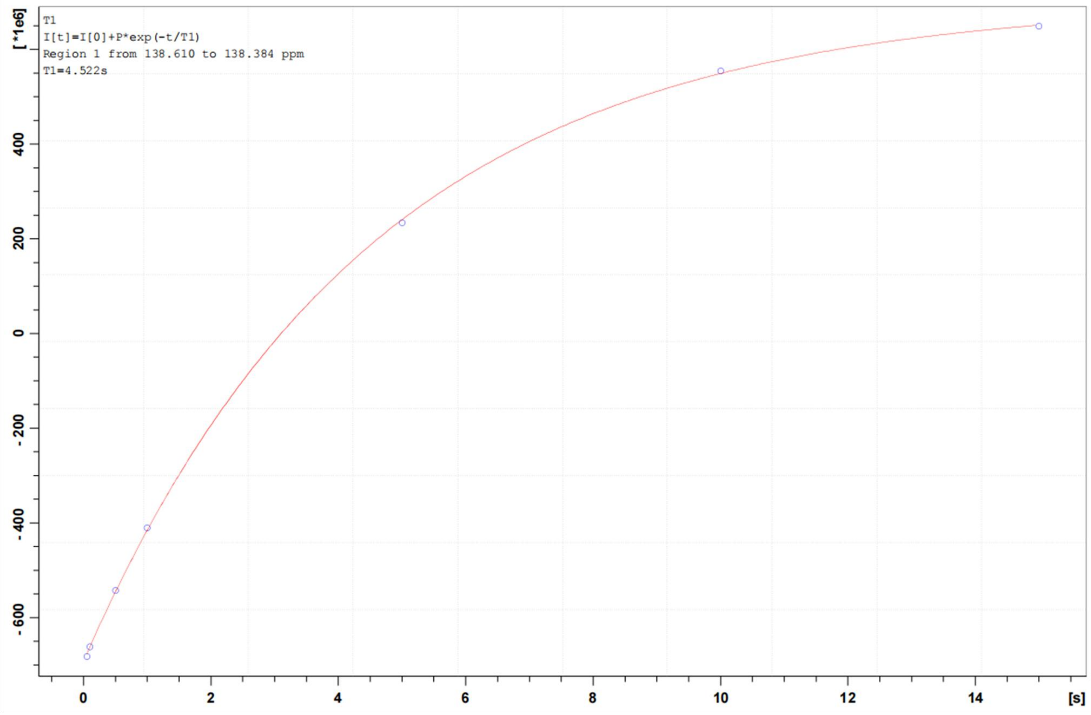

Fig. S3. Standard curve for ^{31}P longitudinal relaxation times of triethyl phosphite

The ^{31}P longitudinal relaxation times (T1) of the sample was measured using a Bruker AVANCE NEO 600 MHz NMR spectrometer with the t1irpg pulse sequence, which employs an inversion recovery method with gradients.

The measured T1 value was 4.522 seconds.

Report:

Sample:

7 points for Integral 1, Integral Region from 138.610 to 138.384 ppm

I[t]=I[0]+P*exp(-t/T1)

Results Comp. 1

I[0] = 1.029e+00

P = -2.043e+00

T1 = 4.522s

SD = 6.694e-03

tau	ppm	integral	intensity
15.000s	138.515	6.4893e+08	1.7769e+08
10.000s	138.517	5.5486e+08	1.905e+08
5.000s	138.517	2.3421e+08	8.3701e+07
1.000s	138.517	-4.1039e+08	-1.6973e+08
500.000m	138.518	-5.4254e+08	-2.357e+08
100.000m	138.518	-6.6166e+08	-3.0083e+08
50.000m	138.518	-6.8211e+08	-3.2126e+08

Experimental procedures for the electrolysis

General procedures for the investigation of EWG-XH:

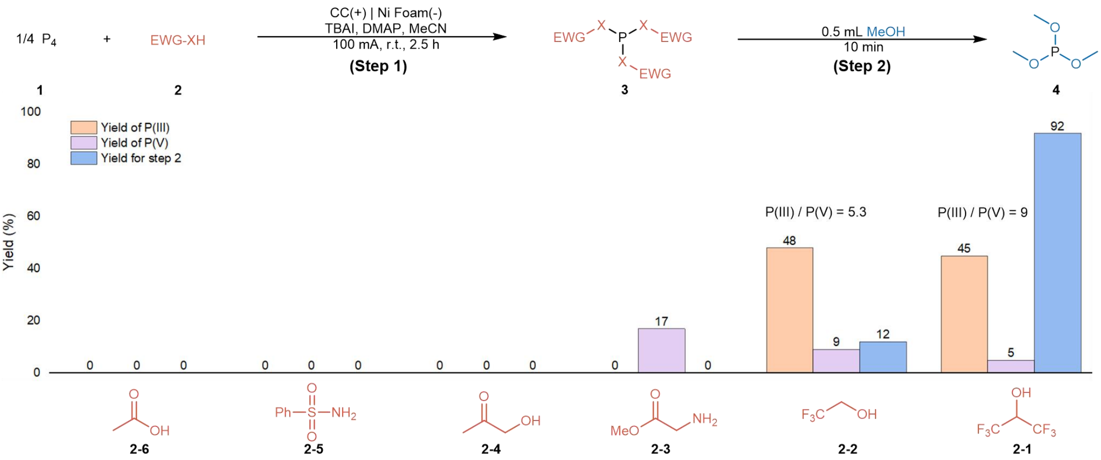

Fig. S4. Choice of electron withdrawing groups

In a 25 mL oven-dried two-necked undivided cell equipped with a stir bar, $^{n}Bu_4NI$ (332.4 mg, 0.9 mmol), DMAP (366.5 mg, 3.0 mmol) were added into the tube. The tube was equipped with carbon cloth (1.5 × 1.5 cm^2) as the anode and Ni foam (2.0 × 1.5 cm^2) as the cathode. This was followed by the addition of P_4 (93.0 mg, 0.75 mmol) in a glovebox (H_2O and O_2 < 0.1 ppm). Then MeCN (4 mL), $CHCl_3$ (3 mL) and EWG-XH (HFIP, TFE, HOAc, PhCOOH, NaCNO, benzene sulfonamide) (9.0 mmol) were added to the tube through a syringe. The mixtures were stirred at a constant current of 100 mA at room temperature for 2.5 h (J = 44.4 mA/cm^2, 3 F/mol). At the end of the reaction, the triethyl phosphate (182.2 mg, 1.0 mmol) was added as an internal standard and stirred for 5 min. Then the yield was determined by assure ^{31}P NMR.

The condition optimized for the synthesis of 3-1:

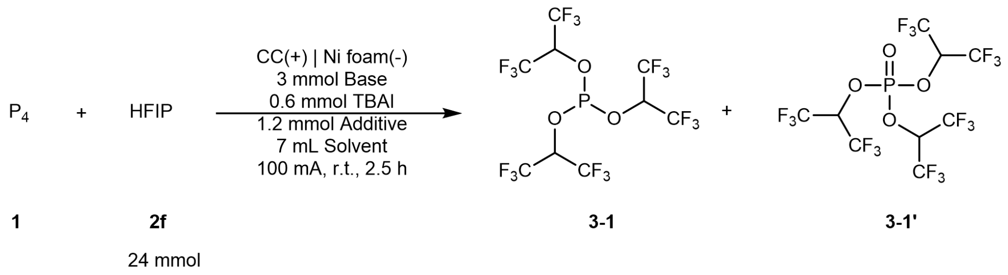

Entry	Solvent	Base	Additive	Yield of **3-1**[a]	Yield of **3-1'**[a]
1	MeCN	DMAP	--	45%	13%
2	MeCN: THF= 4: 3	DMAP	--	33%	13%
3	MeCN: DCM= 4: 3	DMAP	--	50%	10%
4	MeCN: DCE= 4: 3	DMAP	--	53%	10%

5	MeCN: $CHCl_3$= 4: 3	DMAP	--	66%	8%
6	MeCN: $CHCl_3$= 4: 3	4-OMe-py	--	43%	5%
7	MeCN: $CHCl_3$= 4: 3	4-Me-py	--	41%	5%
8	MeCN: $CHCl_3$= 4: 3	DBU	--	35%	9%
9	MeCN: $CHCl_3$= 4: 3	K_2CO_3	--	n.d.	36%
10	MeCN: $CHCl_3$= 4: 3	--	--	19%	11%
11	MeCN: $CHCl_3$= 4: 3	DMAP	LiCl	75%	8%
12	MeCN: $CHCl_3$= 4: 3	DMAP	LiI	57%	13%
13	MeCN: $CHCl_3$= 4: 3	DMAP	LiOAc	n.d.	9%
14	MeCN: $CHCl_3$= 4: 3	DMAP	Li_2SO_4	32%	12%

Table S1. [a] $^nBu_4NI$ (221.6 mg, 0.6 mmol), base (3.0 mmol) were added into the tube. The tube was equipped with carbon cloth (1.5 × 1.5 cm^2) as the anode and Ni foam (2.0 × 1.5 cm^2) as the cathode. This was followed by the addition of P_4 (93.0 mg, 0.75 mmol), additive (1.2 mmol) in a glovebox (H_2O and O_2 < 0.1 ppm). Then solvent (7 mL) and HFIP (2.5 mL, 24 mmol) were added to the tube through a syringe. The mixtures were stirred at a constant current of 100 mA at room temperature for 2.5 h (J = 44.4 mA/cm^2, 3 F/mol).

The control experiment for different nucleophile in standard condition

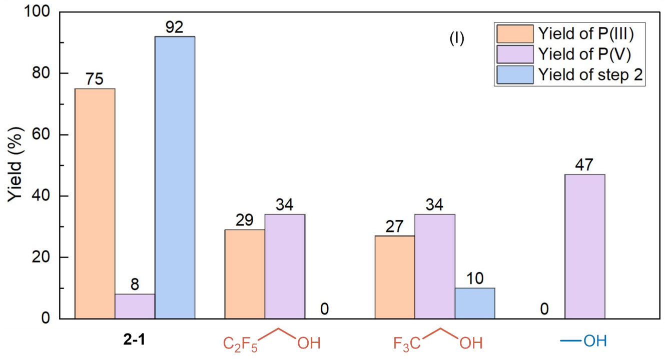

Fig. S5. Different nucleophile in standard condition

In a 10 mL oven-dried two-necked undivided cell equipped with a stir bar, $^nBu_4NI$ (221.6 mg, 0.6 mmol), DMAP (366.5 mg, 3.0 mmol) were added into the beaker. The tube was equipped with carbon cloth (1.5 × 1.5 cm^2) as the anode and Ni foam (2.0 × 1.5 cm^2) as the cathode. This was followed by the addition of P_4 (93.0 mg, 0.75 mmol), LiCl (50.9 mg, 1.2 mmol) in a glovebox (H_2O and O_2 < 0.1 ppm). Then MeCN (4 mL), $CHCl_3$ (3 mL) and EWG-XH (24 mmol) were added to the tube through a syringe. The mixtures were stirred at a constant current of 100 mA at room temperature for 2.5 h (J = 44.4 mA/cm^2, 3 F/mol). At the end of the reaction, the triphenyl phosphate (326.3 mg, 1.0 mmol) was added as an internal standard and stirred for 5 min. Then the yield was determined by assure ^{31}P NMR.

The control experiment for the synthesis of 3-1

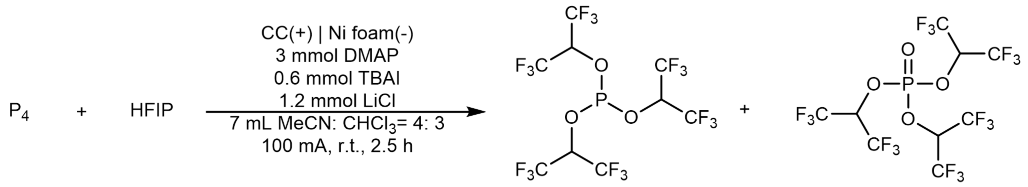

1 **2-1** (24 mmol) **3-1** **3-1'**

Entry	Variation from the standard condition	Yield of **3-1**[a]	Yield of **3-1'**[a]
1	None	75%	8%
2	No DMAP	28%	3%
3	No LiCl	66%	8%
4	6 mmol HFIP	16%	4%
5	$TBABF_4$ instead of TBAI	19%	12%
6	No electricity	n.d.	n.d.

Table S2. [a] $^nBu_4NI$ (221.6 mg, 0.6 mmol), DMAP (366.5 mg, 3.0 mmol) were added into the tube. The tube was equipped with carbon cloth (1.5 × 1.5 cm^2) as the anode and Ni foam (2.0 × 1.5 cm^2) as the cathode. This was followed by the addition of P_4 (93.0 mg, 0.75 mmol), LiCl (50.9 mg, 1.2 mmol) in a glovebox (H_2O and O_2 < 0.1 ppm). Then MeCN (4 mL), $CHCl_3$ (3 mL) and HFIP (2.5 mL, 24 mmol) were added to the tube through a syringe. The mixtures were stirred at a constant current of 100 mA at room temperature for 2.5 h (J = 44.4 mA/cm^2, 3 F/mol).

Experimental procedures for gram-scale reaction

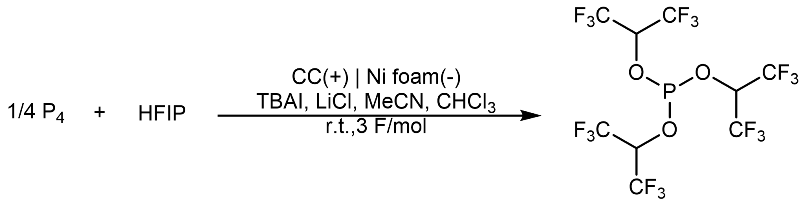

1 **2-1** **3-1**

73% (11.7 g)

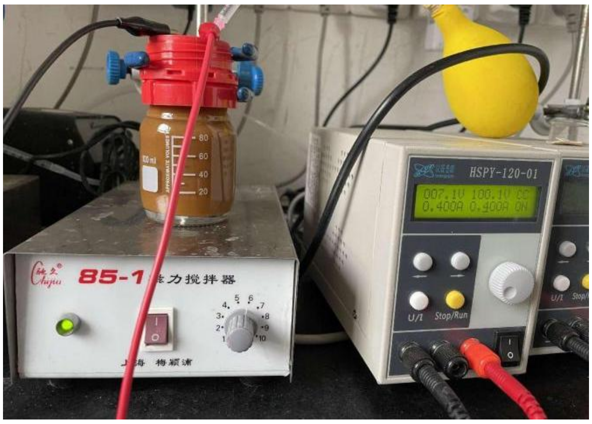

Fig. S6. Device chart for gram-scale reaction

In a 100 mL beaker equipped with a stir bar, $^{n}Bu_4NI$ (3.32 g, 9.0 mmol), DMAP (3.67 g, 30.0 mmol) were added into the beaker. The tube was equipped with carbon cloth (3 × 3 cm^2) as the anode and Ni foam (3 × 3 cm^2) as the cathode. This was followed by the addition of P_4 (930.0 mg, 7.5 mmol), LiCl (508.7 mg, 12 mmol) in a glovebox (H_2O and O_2 < 0.1 ppm). Then MeCN (40 mL), $CHCl_3$ (30 mL) and HFIP (25 mL, 240 mmol) were added to the tube through a syringe. The mixtures were stirred at a constant current of 400 mA at room temperature for 6.25 h (J = 44.4 mA/cm^2, 3 F/mol). At the end of the reaction, the triethyl phosphate (830.8 mg, 5.0 mmol) was added as an internal standard and stirred for 5 min. Then the yield was determined by assure ^{31}P NMR.

Experimental procedures for the reaction scale-up with PV and wind power

The simulation of PV and wind power is achieved by connecting a computer to a power supply and controlling the output power of the power supply through software. The control interface is shown in the following figure, which includes the imported power waveform, real-time recorded actual output power, current, and voltage. The recorded actual power is basically consistent with the preset power. The preset power completely overlaped with the actual power curve, indicating the accuracy of control (Fig. S8 and Fig. S10).

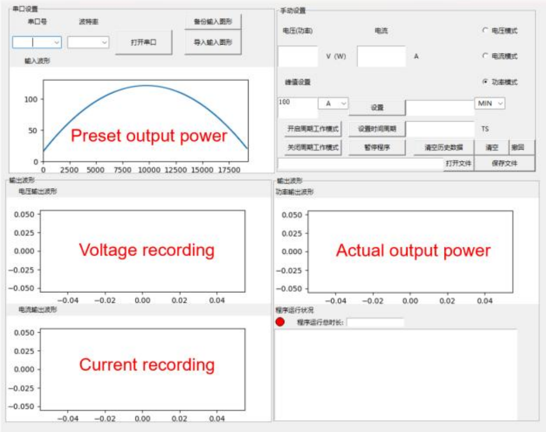

Fig. S7. Software interface for controlling power output

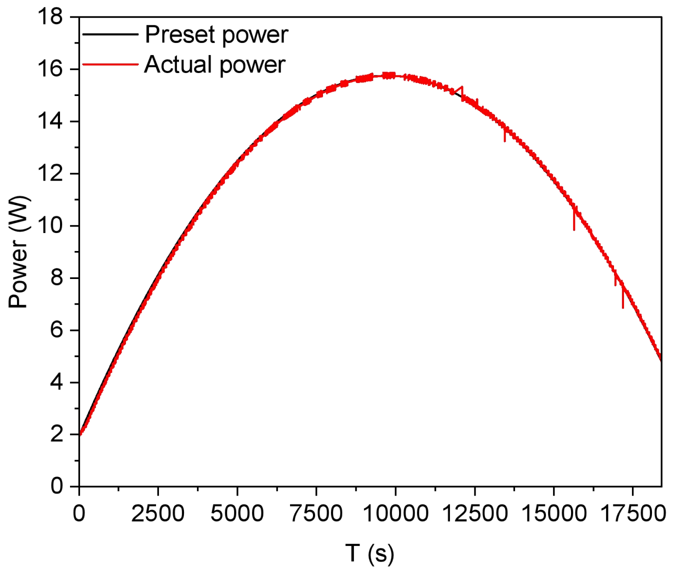

Fig. S8. Preset and actual power of PV

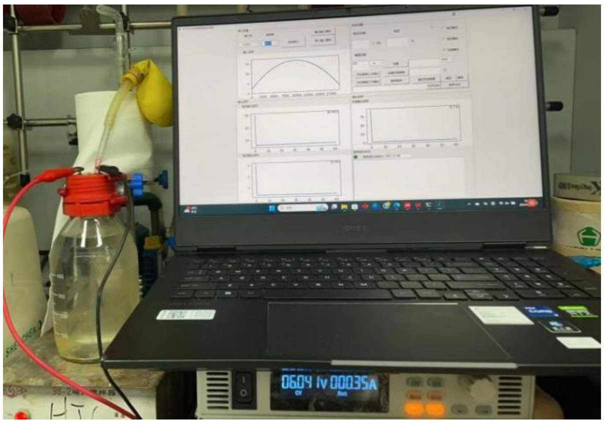

Fig. S9. Device chart for scale-up reaction with PV

Reaction scale-up with PV: In a 500 mL beaker equipped with a stir bar, $^{n}Bu_4NI$ (16.60 g, 45.0 mmol), DMAP (18.35 g, 150.0 mmol) were added into the beaker. The tube was equipped with carbon cloth (8 × 3 cm^2) as the anode and Ni foam (10 × 2.5 cm^2) as the cathode. This was followed by the addition of P_4 (4.65 g, 37.5 mmol), LiCl (2.54 g, 60 mmol) in a glovebox (H_2O and O_2 < 0.1 ppm). Then MeCN (200 mL), $CHCl_3$ (150 mL) and HFIP (150 mL, 1.44 mol) were added to the beaker through a syringe. At the end of the reaction, the triphenyl phosphate (8.16 g, 25.0 mmol) was added as an internal standard and stirred for 10 min. Then the yield was determined by assure ^{31}P NMR.

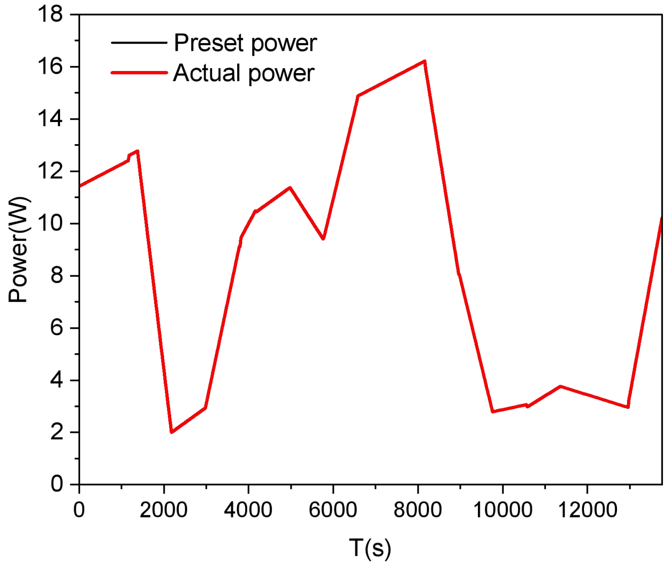

Fig. S10. Preset and actual power of wind power

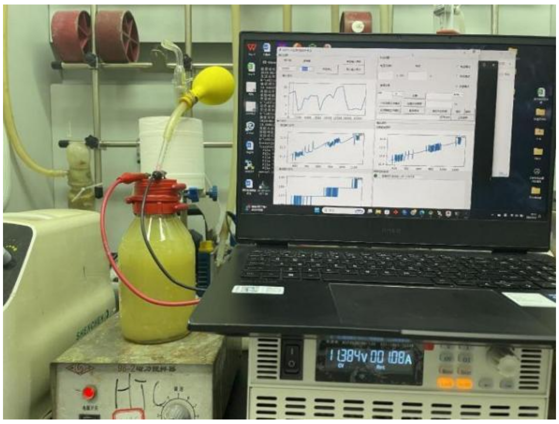

Fig. S11. Device chart for scale-up reaction with wind power

Reaction scale-up with wind power: In a 500 mL beaker equipped with a stir bar, ${}^{n}Bu_4NI$ (16.60 g, 45.0 mmol), DMAP (18.35 g, 150.0 mmol) were added into the beaker. The tube was equipped with carbon cloth (8 × 3 cm^2) as the anode and Ni foam (10 × 2.5 cm^2) as the cathode. This was followed by the addition of P_4 (4.65 g, 150.0 mmol), LiCl (2.54 g, 60 mmol) in a glovebox (H_2O and O_2 < 0.1 ppm). Then MeCN (200 mL), $CHCl_3$ (150 mL) and HFIP (150 mL, 1.44 mol) were added to the beaker through a syringe. At the end of the reaction, the triphenyl phosphate (8.16 g, 25.0 mmol) was added as an internal standard and stirred for 10 min. Then the yield was determined by assure ^{31}P NMR.

Experimental procedures for 100 gram-scale reaction

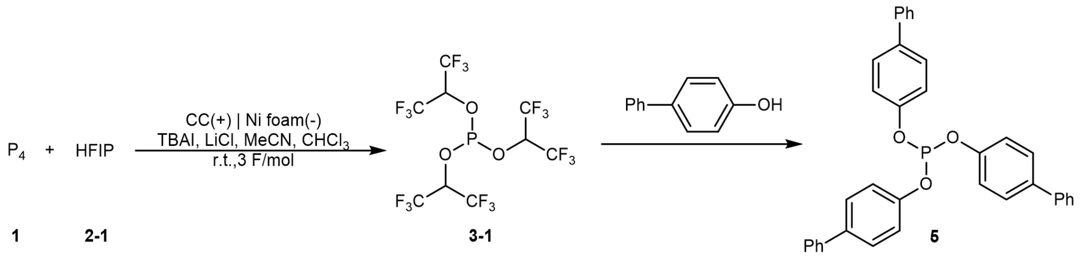

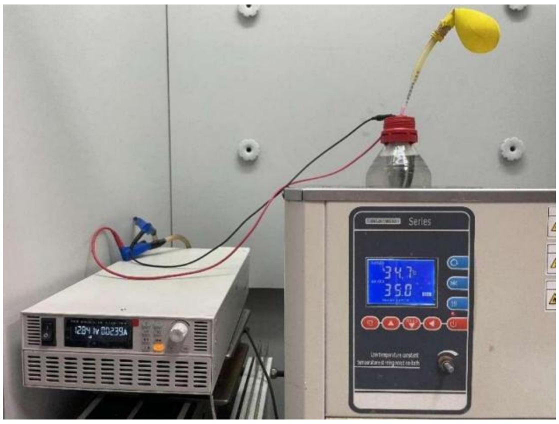

Fig. S12. Device chart for 100 g scale-up reaction

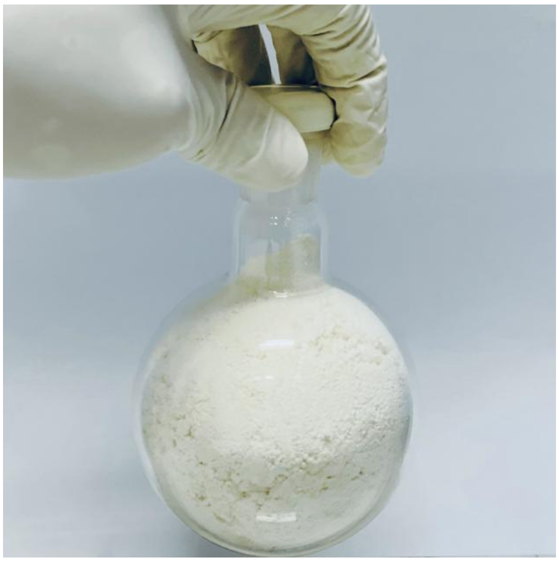

Fig. S13. Isolated product of 5

In a 1000 mL beaker equipped with a stir bar, $^{n}Bu_4NI$ (33.30 g, 90.0 mmol), DMAP (36.70 g, 300.0 mmol) were added into the beaker. The tube was equipped with carbon cloth (14 × 3.5 cm^2) as the anode and Ni foam (14 × 2.5 cm^2) as the cathode. This was followed by the addition of P_4 (9.30 g, 75.0 mmol), LiCl (5.09 g, 120 mmol) in a glovebox (H_2O and O_2 < 0.1 ppm). Then MeCN (400 mL), $CHCl_3$ (300 mL) and HFIP (300 mL, 2.88 mol) were added to the beaker through a syringe. The reagent bottle was cooled by a cold trap. The mixtures were stirred at a constant current of 2.4 A at 35 °C for 10.4 h (J = 49 mA/cm^2, 3 F/mol). At the end of the reaction, the triphenyl phosphate (8.16 g, 25.0 mmol) was added as an internal standard and stirred for 10 min. Then the yield was determined by assure ^{31}P NMR. After that, biphenyl-4-ol (295.8 g, 1.74 mol) was added into the beaker and stirred for another 24 h. Then the desired products were obtained after purification by flash chromatography on 200-300 silica gel (petroleum: EtOAc = 200:1). The isolated yield: 95.2 g, 59%.

Experimental procedures for the separation of 3-1

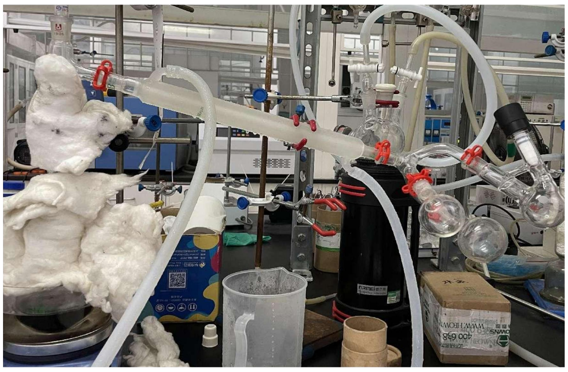

Fig. S14. Diagram of vacuum distillation device

As shown in Fig. S14, the distillation apparatus was purged with nitrogen throughout the process to ensure a nitrogen atmosphere before distillation. The distillation column had seven plates. The mixture was then added to a 250 mL three-necked flask, which was heated while the pump was turned on to bring the solution to a boil. The initial distillate collected was the solvent mixture (MeCN, $CHCl_3$ and HFIP). Subsequently, the target distillate was evaporated and collected under vacuum (boiling point: 50 °C at 10 Torr). Yield: 3.30 g (32%). Due to its susceptibility to air oxidation and hydrolysis, **3-1** was dissolved in PhMe and stored in a glovebox (H_2O and O_2 < 0.1 ppm). The concentration of **3-1** was 0.08 M.

Experimental procedures for converting 3-1 to OPC (III)

General procedure for reaction of 3-1 with nucleophiles to phosphite esters (from primary alcohol):

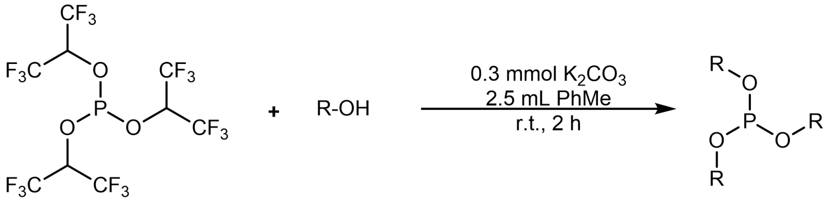

3-1 **4, 6-17**

Method A: In a 10 mL oven-dried schlenk tube equipped with a stir bar, K_2CO_3 (41.5 mg, 0.3 mmol) was added into the tube. This was followed by the addition of **3-1** (0.08 M in PhMe, 2.5 mL) in a glovebox (H_2O and O_2 < 0.1 ppm). Then alcohol (1.0 mmol) was added to the tube through a syringe. The mixtures were stirred at room temperature for 2 h. At the end of the reaction, the triphenylphosphorus oxide (27.8 mg, 0.1 mmol) was added as an internal standard and stirred for 5 min. Then the yield was determined by assure ^{31}P NMR. **Method B:** Otherwise above general procedure, the mixtures were stirred at 40 ℃ for 4 h.

General procedure for reaction of 3-1 with nucleophiles to phosphite esters (from secondary alcohol and tertiary alcohol):

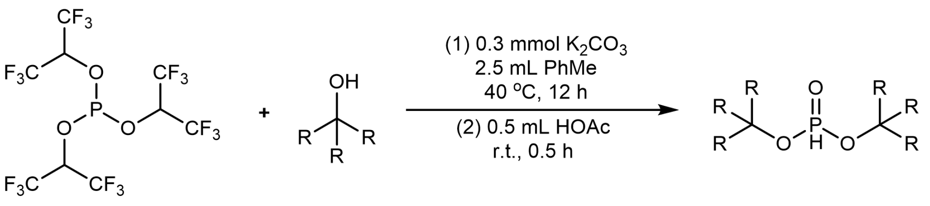

3-1 **18-23**

Method C: In a 10 mL oven-dried schlenk tube equipped with a stir bar, K_2CO_3 (41.5 mg, 0.3 mmol) was added into the tube. This was followed by the addition of **3-1** (0.08 M in PhMe, 2.5 mL) in a glovebox (H_2O and O_2 < 0.1 ppm). Then alcohol (1.0 mmol) was added to the tube through a syringe. The mixtures were stirred at 40 °C for 12 h. At the end of the reaction, the triphenylphosphorus oxide (27.8 mg, 0.1 mmol) was added as an internal standard. Then HOAc (0.2 mmol) and H_2O (0.2 mL) were added to the tube through a syringe and stirred at room temperature for 0.5 h . Then the yield was determined by assure ^{31}P NMR. After that, the desired products were obtained after purification by flash chromatography on 200-300 silica gel (petroleum: EtOAc = 5:1).

General procedure for reaction of 3-1 with nucleophiles to phosphite esters (from phenol):

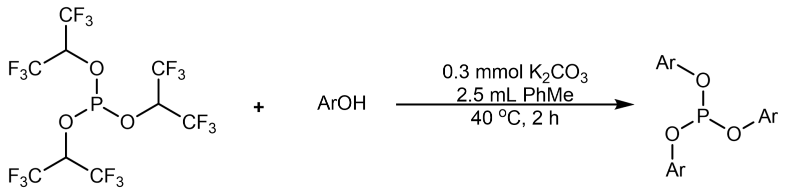

3-1 **5, 24-31**

Method D: In a 10 mL oven-dried schlenk tube equipped with a stir bar, K_2CO_3 (41.5 mg, 0.3 mmol), phenol (1.0 mmol) were added into the tube. This was followed by the addition of **3-1** (0.08 M in PhMe, 2.5 mL) in a glovebox (H_2O and O_2 < 0.1 ppm) The mixtures were stirred at 40 °C for 2 h. At the end of the reaction, the triphenylphosphorus oxide (27.8 mg, 0.1 mmol) was added as an internal standard and stirred for 5 min. Then the yield was determined by assure ^{31}P NMR. After that, the desired products were obtained after purification by flash chromatography on 200-300 silica gel (petroleum: DCM = 10:1).

General procedure for reaction of 3-1 with nucleophiles to phosphite esters (from phenol and alcohol):

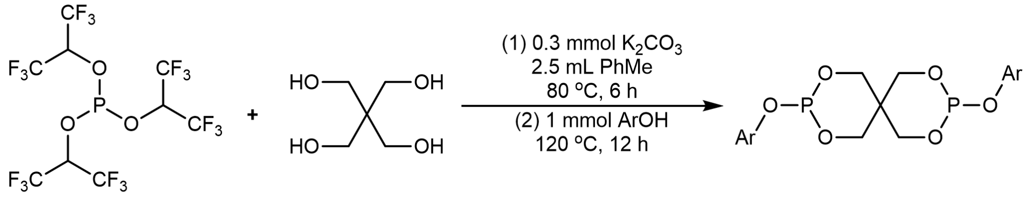

3-1 **32-35**

Method E: In a 10 mL oven-dried sealed tube equipped with a stir bar, K_2CO_3 (41.5 mg, 0.3 mmol), pentaerythritol (0.2 mmol) were added into the tube. This was followed by the addition of

3-1 (0.08 M in PhMe, 5.0 mL) in a glovebox (H_2O and O_2 < 0.1 ppm). The mixtures were stirred at 80 °C for 6 h. Then the mixtures were transfered to sealed tube. Phenol (1.0 mmol) was added into the tube and stirred at 120 °C for 12 h. At the end of the reaction, the triphenylphosphorus oxide (27.8 mg, 0.1 mmol) was added as an internal standard and stirred for 5 min. Then the yield was determined by assure ^{31}P NMR. After that, the desired products were obtained after purification by flash chromatography on 200-300 silica gel (petroleum: DCM = 10:1). **Method F:** Otherwise above general procedure, the second step was stirred at 40 °C for 4 h.

General procedure for reaction of 3-1 with nucleophiles to phosphite esters (from phenol and alcohol):

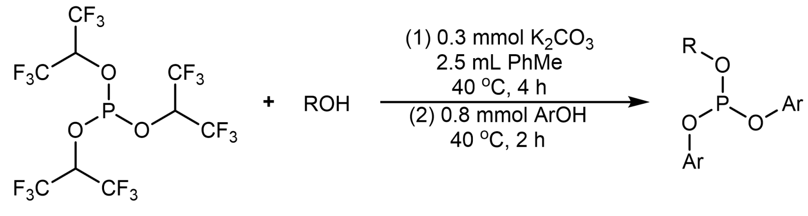

3-1 **36-40**

Method G: In a 10 mL oven-dried schlenk tube equipped with a stir bar, K_2CO_3 (41.5 mg, 0.3 mmol) was added into the tube. This was followed by the addition of **3-1** (0.08 M in PhMe, 2.5 mL) in a glovebox (H_2O and O_2 < 0.1 ppm). Then alcohol (0.2 mmol) was added to the tube through a syringe. The mixtures were stirred at 40 °C for 4 h. After that, phenol (0.8 mmol) was added into the tube and stirred at 40 °C for 2 h. At the end of the reaction, the triphenylphosphorus oxide (27.8 mg, 0.1 mmol) was added as an internal standard and stirred for 5 min. Then the yield was determined by assure ^{31}P NMR. After that, the desired products were obtained after purification by flash chromatography on 200-300 silica gel (petroleum: EtOAc = 10:1).

General procedure for reaction of 3-1 with nucleophiles to trialkylphosphine or triarylphosphine (from alkyl and aryl grignard reagent):

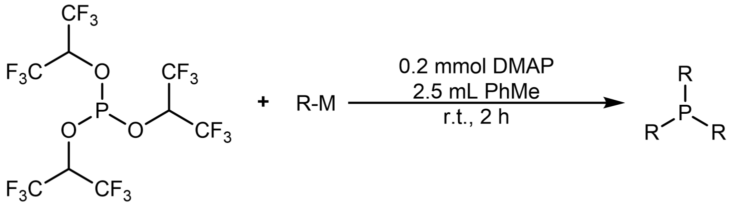

3-1 **41-54**

Method H: In a 10 mL oven-dried schlenk tube equipped with a stir bar, DMAP (24.4 mg, 0.2 mmol) was added into the tube. This was followed by the addition of **3-1** (0.08 M in PhMe, 2.5 mL) in a glovebox (H_2O and O_2 < 0.1 ppm). Then alkyl grignard reagent (1.0 mmol, 1 M in THF) was added to the tube through a syringe. The mixtures were stirred at room temprature for 2 h. At the end of the reaction, the triethyl phosphite (17 μL, 0.1 mmol) was added as an internal standard and stirred for 5 min. Then the yield was determined by assure ^{31}P NMR. After that, S_8 (16.0 mg, 0.5 mmol) was added to the tube to avoid oxidation and stirred for 30 min. Then the desired products were obtained after purification by flash chromatography on 200-300 silica gel (petroleum: DCM = 1:1). **Method I:** the first step was ^{i}PrMgCl (0.4 mmol), after that, aryl grignard reagent (1.0 mmol) was added into the tube and stirred at room temperature for another 1 h.

Substrate expansion for 3-1

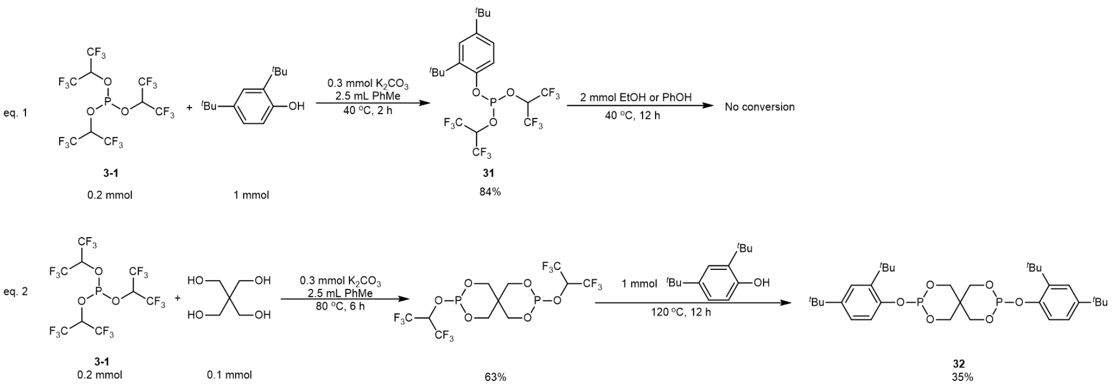

Fig. S15. The reaction attempt for 3-1

Eq.1: In a 10 mL oven-dried schlenk tube equipped with a stir bar, K_2CO_3 (41.5 mg, 0.3 mmol), 2,4-di-tert-butylphenol (619.0 mg, 1 mmol) were added into the tube. This was followed by the addition of **3-1** (0.08 M in PhMe, 2.5 mL) in a glovebox (H_2O and O_2 < 0.1 ppm). The mixture was stirred at 40 °C for 2 h. At the end of the reaction, the triphenylphosphorus oxide (27.8 mg, 0.1 mmol) was added as an internal standard and stirred for 5 min and the yield was determined by assure ^{31}P NMR. Then EtOH or Phenol (2 mmol) was added to the tube and stirred at 40 °C for another 12 h. Finally, there was no significant peak on ^{31}P NMR.

Eq.2: In a 10 mL oven-dried schlenk tube equipped with a stir bar, K_2CO_3 (41.5 mg, 0.3 mmol), pentaerythritol (13.6 mg, 0.1 mmol) were added into the tube. This was followed by the addition of **3-1** (0.08 M in PhMe, 2.5 mL) in a glovebox (H_2O and O_2 < 0.1 ppm). The mixture was stirred at 80 °C for 6 h. At the end of the reaction, the triphenylphosphorus oxide (27.8 mg, 0.1 mmol) was added as an internal standard and stirred for 5 min and the yield was determined by assure ^{31}P NMR. Then the mixture was transferred into a 25 mL sealed tube, 2,4-di-tert-butylphenol (206.3 mg, 1 mmol) was added into the tube and stirred at 120 °C for another 12 h.

Experimental procedures for the recycling of HFIP

Additionally, we explored the feasibility of recovering HFIP and the solvents used in the reaction. In a 10 g-scale synthesis, we employed 25 mL of HFIP, 40 mL of MeCN, and 25 mL of $CHCl_3$. Following the tandem electro-thermal synthesis, the solvents were recovered by distillation (Fig. S16). The recovered solvent mixture was then reused in the synthesis of **3-1**, yielding the **3-1** with a 72% yield (Fig. S16). These results confirm the method' s scalability and the effective recovery and reuse of HFIP and solvents.

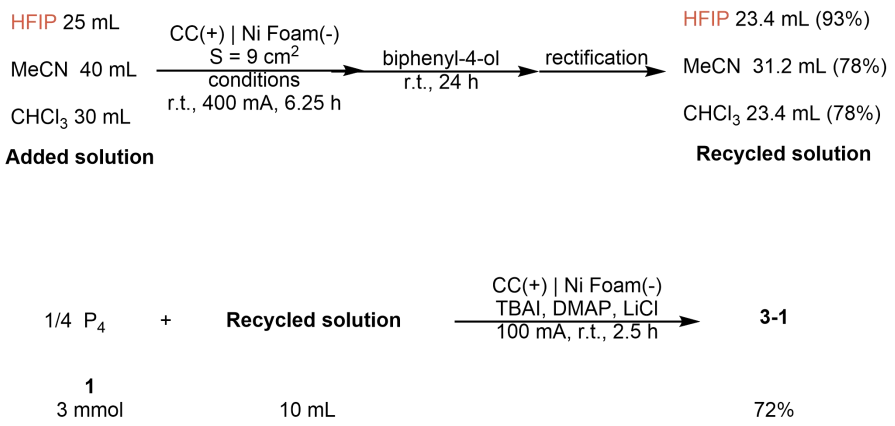

Fig. S16. Recycling of the HFIP moiety

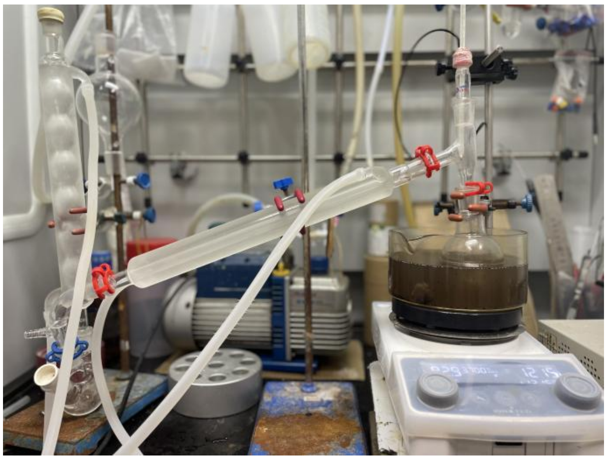

Fig. S17. The diagram of the recovery device of HFIP

In a 100 mL beaker equipped with a stir bar, ^{n}Bu$_4$NI (3.32 g, 9.0 mmol), DMAP (3.67 g, 30.0 mmol) were added into the beaker. The tube was equipped with carbon cloth (3 × 3 cm^2) as the anode and Ni foam (3 × 3 cm^2) as the cathode. This was followed by the addition of P_4 (930.0 mg, 7.5 mmol), LiCl (508.7 mg, 12 mmol) in a glovebox (H_2O and O_2 < 0.1 ppm). Then MeCN (40 mL), $CHCl_3$ (30 mL) and HFIP (25 mL, 240 mmol) were added to the tube through a syringe. The mixtures were stirred at a constant current of 400 mA at room temperature for 6.25 h (J = 44.4 mA/cm^2, 3 F/mol). After that, biphenyl-4-ol (25.5 g, 150.0 mmol) was added into the beaker and stirred for another 24 h. At the end of the reaction, the reaction mixture was transferred into a three-necked flask as shown in Fig. S17 and perform distillation under vacuum to recover 78 mL of the solution. According to the ^{1}H NMR spectrum, the molar ratio of the three substances is MeCN: HFIP: $CHCl_3$ = 2.7: 1: 1.3. The conversion from molar ratio to volume ratio can be done using the following formula Eq.2：

$$\frac{V_1}{V_2} = \frac{m_1/\rho_1}{m_2/\rho_2} = \frac{n_1 M_1/\rho_1}{n_2 M_2/\rho_2}$$

M is the molecular weight of the substance

n is the amount of substance

ρ is the density of the substance

Eq.2. the formula for converting molar amount to volume

Using the above formula, the recovered volumes of the three substances were obtained: MeCN: 31.2 mL (78%), HFIP: 23.4 mL (93%), $CHCl_3$: 23.4 mL (78%). The recycled solution was treated

with molecular sieves for dehydration.

The second reaction：In a 10 mL oven-dried two-necked undivided cell equipped with a stir bar, ${}^{n}Bu_4NI$ (221.6 mg, 0.6 mmol), DMAP (366.5 mg, 3.0 mmol) were added into the beaker. The tube was equipped with carbon cloth (1.5 × 1.5 cm^2) as the anode and Ni foam (2.0 × 1.5 cm^2) as the cathode. This was followed by the addition of P_4 (93.0 mg, 0.75 mmol), LiCl (50.9 mg, 1.2 mmol) in a glovebox (H_2O and O_2 < 0.1 ppm). Then, 10 mL of the recycled solution was added to the reaction tube through a syringe. The mixtures were stirred at a constant current of 100 mA at room temperature for 2.5 h (J = 44.4 mA/cm^2, 3 F/mol). At the end of the reaction, the triphenyl phosphate (326.3 mg, 1.0 mmol) was added as an internal standard and stirred for 5 min. Then the yield was determined by assure ${}^{31}P$ NMR.

Experimental procedures for the UV-Vis Spectroscopy Study

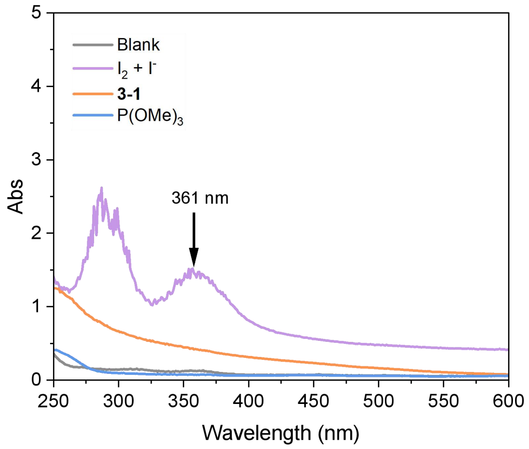

Fig. S18. UV-Vis absorbance at -30 ℃

UV-Vis absorption data was acquired on an Agilent Cary 8454 spectrophotometer. I_2 (10.2 mg, 0.04 mmol) and TBAI (14.8 mg, 0.04 mmol) was dissolved in solvents (MeCN: $CHCl_3$= 4: 1, 10 mL), which called Solution A. **3-1** (0.04 mmol) was dissolved in solvents (MeCN: $CHCl_3$= 4: 1, 10 mL), which called Solution B. $P(OMe)_3$ (0.04 mmol) was dissolved in solvents (MeCN: $CHCl_3$= 4: 1, 10 mL), which called Solution C. In a screw-top quartz cuvette equipped with a stir bar, each solution (10 μL) was added into the screw-top quartz cuvette through a microsyringe, then MeCN (3 mL) was added, absorption was subtracted as background. UV-Vis spectra was recorded at -30 °C. The UV-Vis absorption kinetic data was collected from 600 nm to 250 nm, scan speed: 2400 nm/min. We found that the absorption peak at about 360 nm was the absorption peak of I_3^-[12], and I_3^- was a mixture of iodide ions and iodide elements. Therefore, we hoped to observe the change of the absorption peak of I_3^- in the reaction to reflect the change of iodide elements, so as to reflect the oxidation difficulty of different phosphite esters.

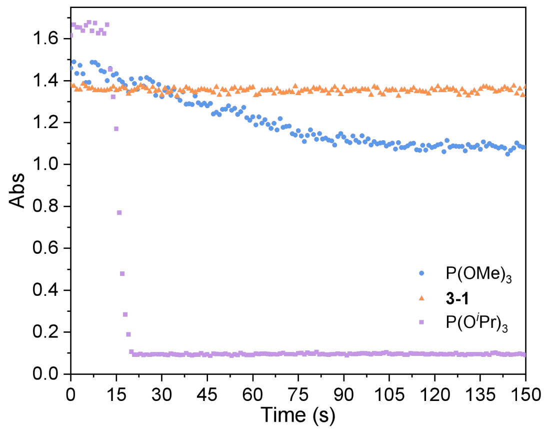

Fig. S19. UV-Vis kinetics

I_2 (10.2 mg, 0.04 mmol) and TBAI (14.8 mg, 0.04 mmol) was dissolved in solvents (MeCN: $CHCl_3$= 4: 1, 10 mL), which called Solution A. Phosphite triester (0.04 mmol) was dissolved in solvents (MeCN: $CHCl_3$= 4: 1, 10 mL), which called Solution B. In a screw-top quartz cuvette equipped with a stir bar, the Solution A (10 μL) was added into the screw-top quartz cuvette through a microsyringe, then MeCN (3 mL) was added and stirred at -30 °C. Then Solution B (10 μL) was added into the screw-top quartz cuvette through a microsyringe and UV-Vis spectra was recorded at -30 °C. The UV-Vis absorption kinetic data was collected at 361 nm, scan speed: 60 times/min.

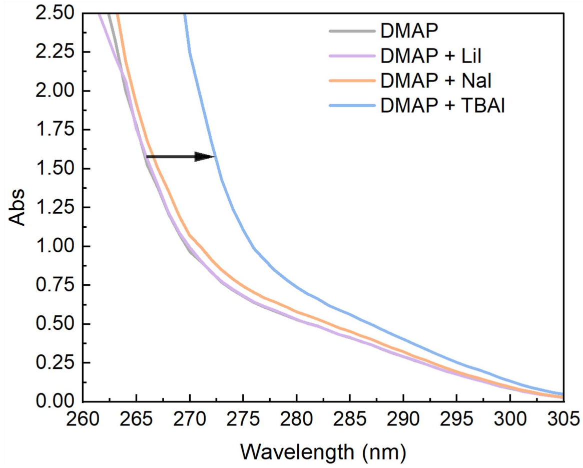

Fig. S20. UV-Vis absorbance for DMAP and different iodides

DMAP (122.2 mg, 1.0 mmol) was dissolved in 5 mL MeCN, which called Solution A. TBAI (369.3 mg, 1.0 mmol) was dissolved in 5 mL MeCN, which called Solution B. In a screw-top quartz cuvette equipped with a stir bar, the Solution A (2 μL) was added into the screw-top quartz cuvette through a microsyringe, then MeCN (3 mL) was added and stirred at room temperature. Then Solution B (2 μL) was added into the screw-top quartz cuvette through a microsyringe and UV-Vis spectra was recorded at room temperature. Absorption was subtracted as background. The UV-Vis absorption kinetic data was collected from 600 nm to 250 nm, scan speed: 2400 nm/min.

LiI (133.8 mg, 1.0 mmol) and NaI (149.9 mg, 1.0 mmol) were used instead of TBAI, and did UV-Vis study as the same method.

Experimental Procedures for Cyclic Voltammetry

Cyclic voltammograms were recorded with a CorrTest® CS2350H bipotentiostat at room temperature. $^nBu_4NPF_6$ (193.7 mg, 0.50 mmol) was used as the supporting electrolyte. The scan rate was 10 mV / s. The scan range is between 0 V to 2.0 V or 0 V to 3.0 V.

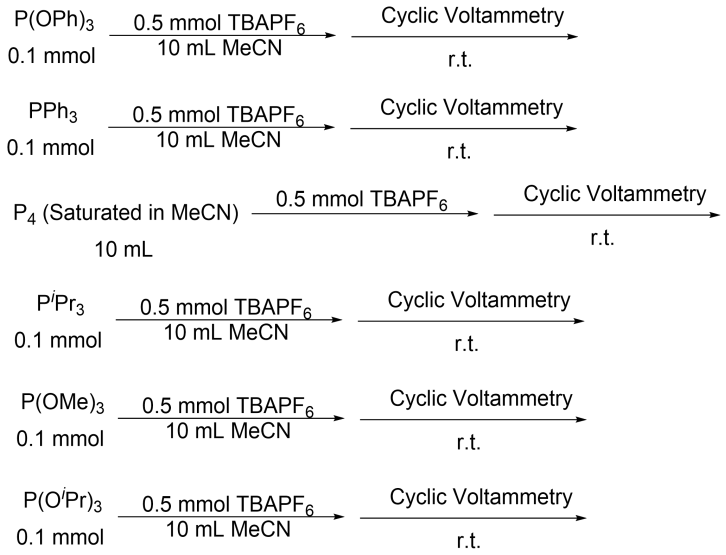

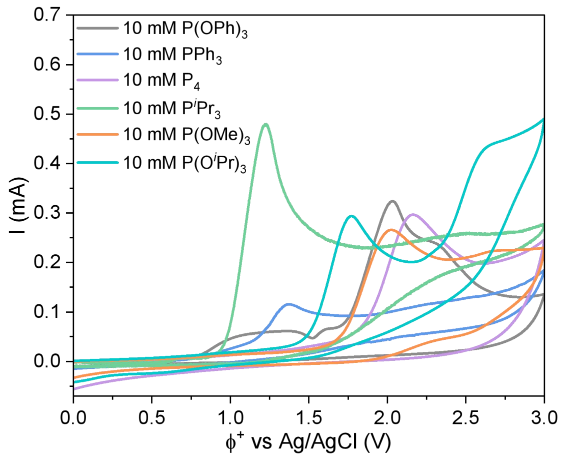

Fig. S21. Cyclic voltammetry curve for common trivalent phosphide and P_4

In an undivided cell equipped with a stir bar, $TBAPF_6$ (193.7 mg, 0.50 mmol), different common trivalent phosphide (0.10 mmol) or P_4 (saturated in MeCN, 5.2 mM, 10 mL) were added to the undivided cell respectively. Solvents (MeCN, 10 mL or 0 mL), were then added to the undivided cell. The undivided cell was equipped with glassy-carbon disk working electrode (diameter, 3.0 mm) and Pt wire auxiliary electrode. The scan range was 0 V to 3.0 V. All potentials are referenced against the Ag/AgCl reference electrode.

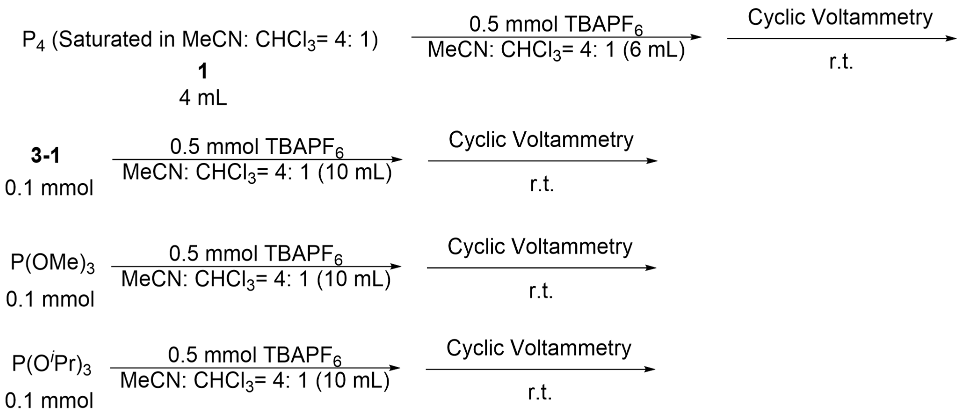

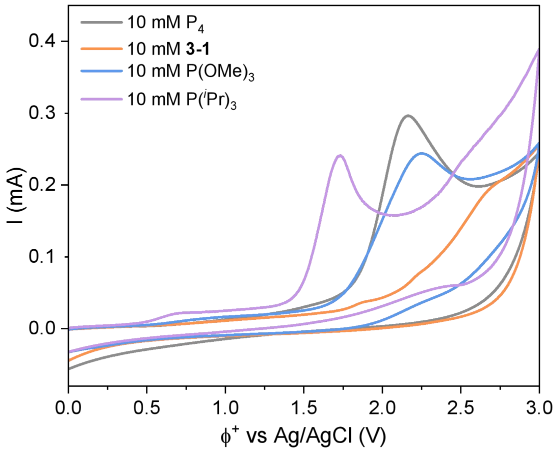

Fig. S22. Cyclic voltammetry curve for phosphite ester and P_4

In an undivided cell equipped with a stir bar, $TBAPF_6$ (193.7 mg, 0.50 mmol), phosphite ester (0.10 mmol) or P_4 (saturated in MeCN: $CHCl_3$= 4: 1, 10 mM, 4 mL) were added to the undivided cell respectively. Solvents (MeCN: $CHCl_3$= 4: 1, 10 mL or 6 mL), were then added to the undivided cell. The undivided cell was equipped with glassy-carbon disk working electrode (diameter, 3.0 mm) and Pt wire auxiliary electrode. The scan range was 0 V to 3.0 V. All potentials are referenced against the Ag/AgCl reference electrode.

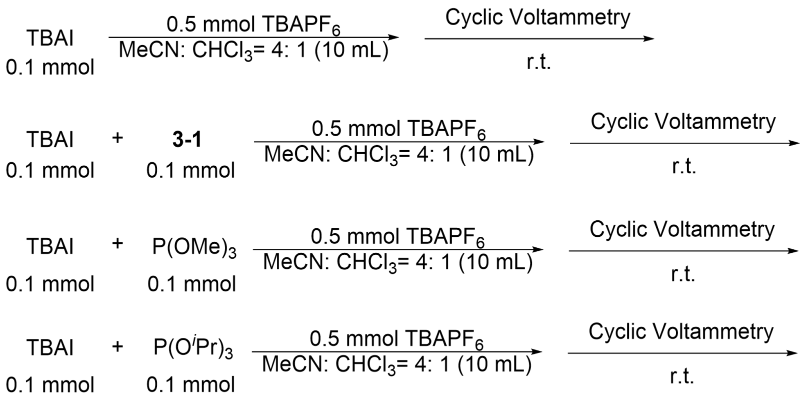

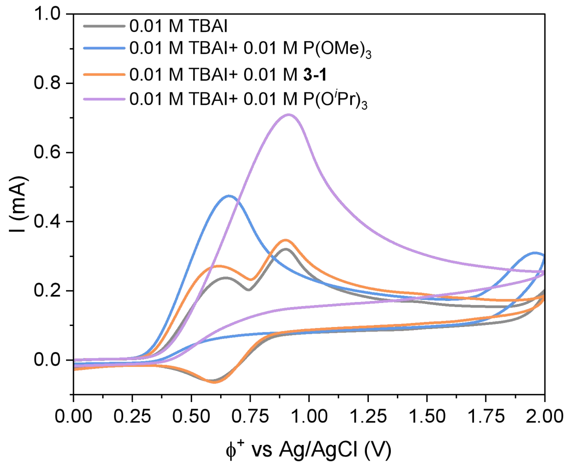

Fig. S23. Cyclic voltammetry curve for different concentrations of phosphite ester in TBAI solution

In an undivided cell equipped with a stir bar, $TBAPF_6$ (193.7 mg, 0.50 mmol), TBAI (36.9 mg, 0.10 mmol), phosphite ester (0.10 mmol) were added to the undivided cell respectively. Solvents (MeCN: $CHCl_3$= 4: 1, 10 mL), were then added to the undivided cell. The undivided cell was equipped with glassy-carbon disk working electrode (diameter, 3.0 mm) and Pt wire auxiliary electrode. The scan range was 0 V to 2.0 V. All potentials are referenced against the Ag/AgCl reference electrode.

We measured the oxidation potentials of P_4, TBAI, DMAP, and lithium chloride using CV tests. Iodide ions exhibited three oxidation peaks between 0-3 volts at 0.47 V, 0.86 V, and 2.32 V (Fig. S21, purple line). The oxidation potential of DMAP was higher than that of iodide ions, at 1.31 V (Fig. S21, orange line). The saturated P_4 solution, at a concentration of 7.58 mM, had an oxidation potential of 1.96 V (Fig. S21, blue line). Lithium chloride did not show any significant oxidation peaks between 0-3 V (Fig. S21, green line).Adding varying concentrations of P_4 to the TBAI solution increased the peak current, indicating that oxidized iodine species react with P_4 (Fig. S22).

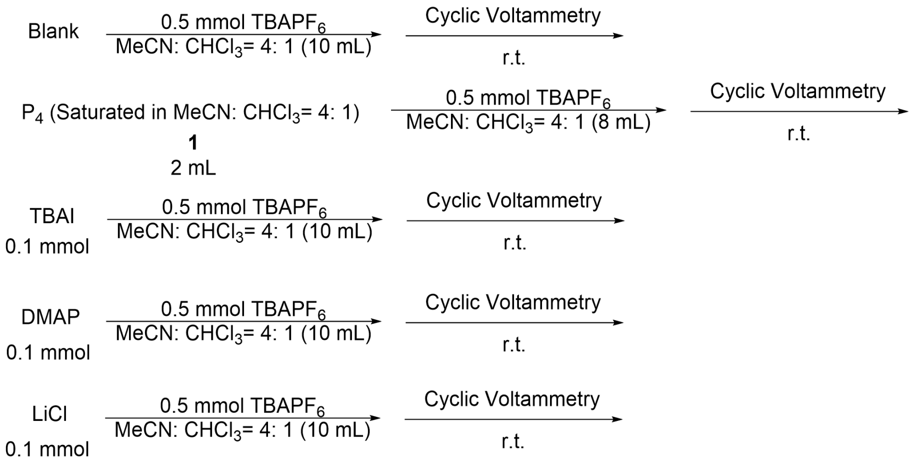

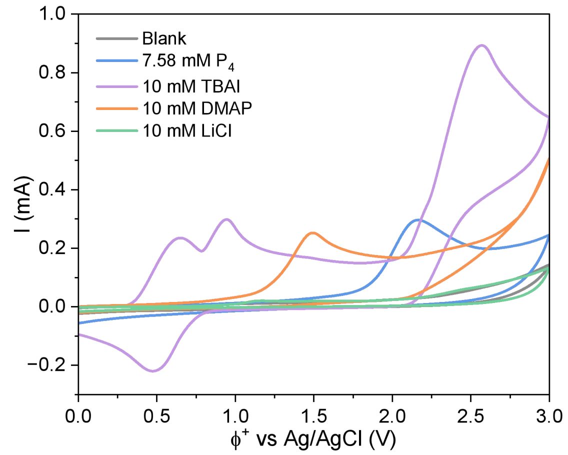

Fig. S24. Cyclic voltammetry curve for different concentrations of several substances in the reaction system

In an undivided cell equipped with a stir bar, $TBAPF_6$ (193.7 mg, 0.50 mmol), substance (0.10 mmol) or P_4 (saturated in MeCN: $CHCl_3$= 4: 1, 39 mM, 2 mL) were added to the undivided cell respectively. Solvents (MeCN: $CHCl_3$= 4: 1, 10 mL or 8 mL), were then added to the undivided cell. The undivided cell was equipped with glassy-carbon disk working electrode (diameter, 3.0 mm) and Pt wire auxiliary electrode. The scan range was 0 V to 3.0 V. All potentials are referenced against the Ag/AgCl reference electrode.

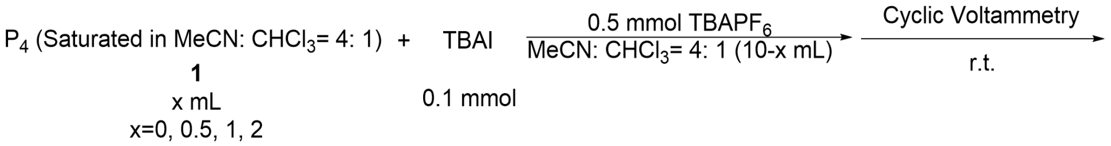

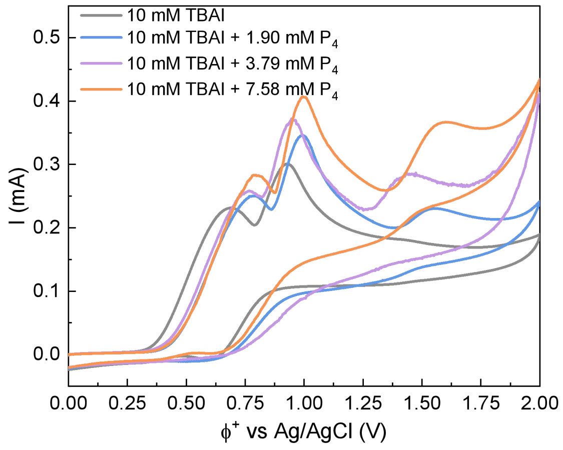

Fig. S25. Cyclic voltammetry curve for different concentrations of white phosphorus in TBAI solution

In an undivided cell equipped with a stir bar, $TBAPF_6$ (193.7 mg, 0.50 mmol), TBAI (36.9 mg, 0.10 mmol), P_4 (saturated in MeCN: $CHCl_3$= 4: 1, 39 mM, x mL) were added to the undivided cell. Solvents (MeCN: $CHCl_3$= 4: 1, 10-x mL), were then added to the undivided cell. The undivided cell was equipped with glassy-carbon disk working electrode (diameter, 3.0 mm) and Pt wire auxiliary electrode. The scan range was 0 V to 2.0 V. All potentials are referenced against the Ag/AgCl reference electrode.

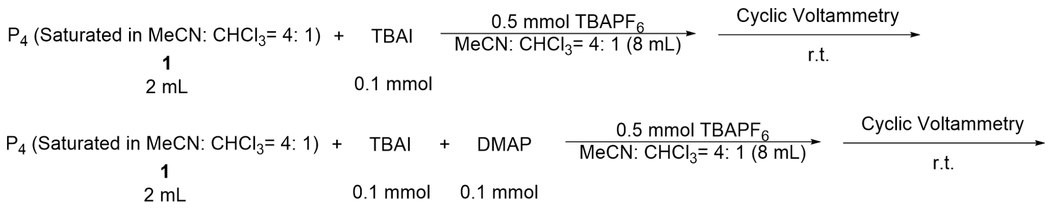

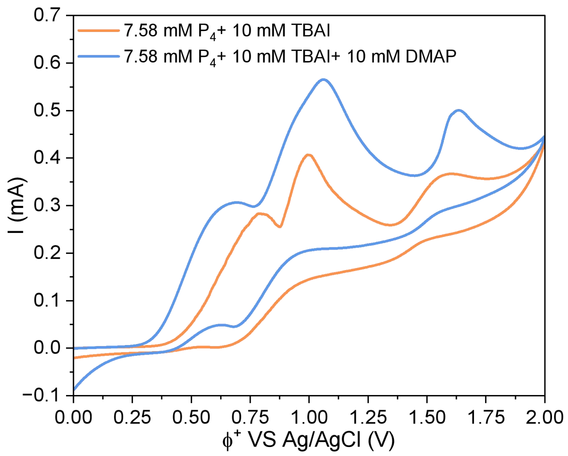

Fig. S26. Cyclic voltammetry curve for DMAP in TBAI and P_4 solution

In an undivided cell equipped with a stir bar, $TBAPF_6$ (193.7 mg, 0.50 mmol), TBAI (36.9 mg, 0.10 mmol), P_4 (saturated in MeCN: $CHCl_3$= 4: 1, 39 mM, 2 mL), DMAP (12.2 mg, 0.10 mmol) were added to the undivided cell. Solvents (MeCN: $CHCl_3$= 4: 1, 8 mL), were then added to the undivided cell. The undivided cell was equipped with glassy-carbon disk working electrode (diameter, 3.0 mm) and Pt wire auxiliary electrode. The scan range was 0 V to 2.0 V. All potentials are referenced against the Ag/AgCl reference electrode.

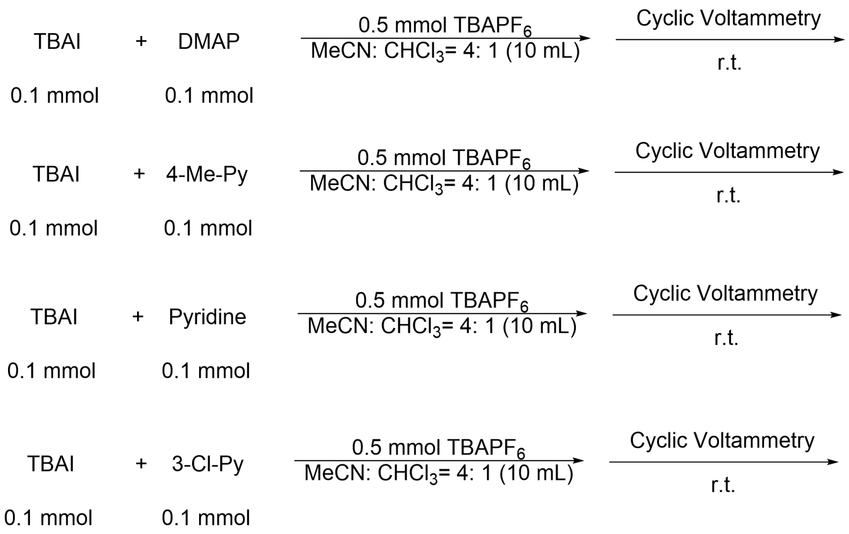

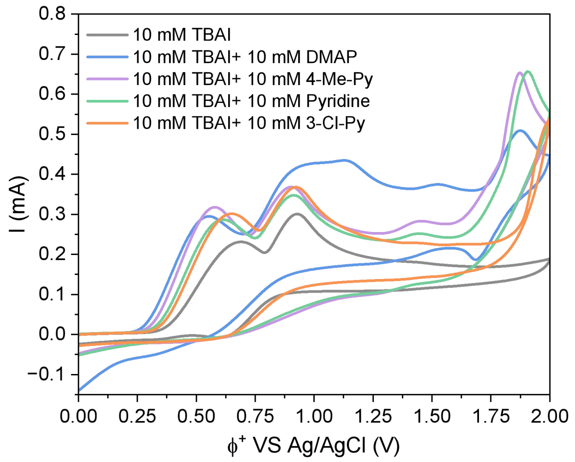

Fig. S27. Cyclic voltammetry curve for the interaction of different types of pyridines with TBAI

In an undivided cell equipped with a stir bar, $TBAPF_6$ (193.7 mg, 0.50 mmol), TBAI (36.9 mg, 0.10 mmol), pyridine (0.1 mmol) were added to the undivided cell. Solvents (MeCN: $CHCl_3$= 4: 1, 10 mL), were then added to the undivided cell. The undivided cell was equipped with glassy-carbon disk working electrode (diameter, 3.0 mm) and Pt wire auxiliary electrode. The scan range was 0 V to 2.0 V. All potentials are referenced against the Ag/AgCl reference electrode.

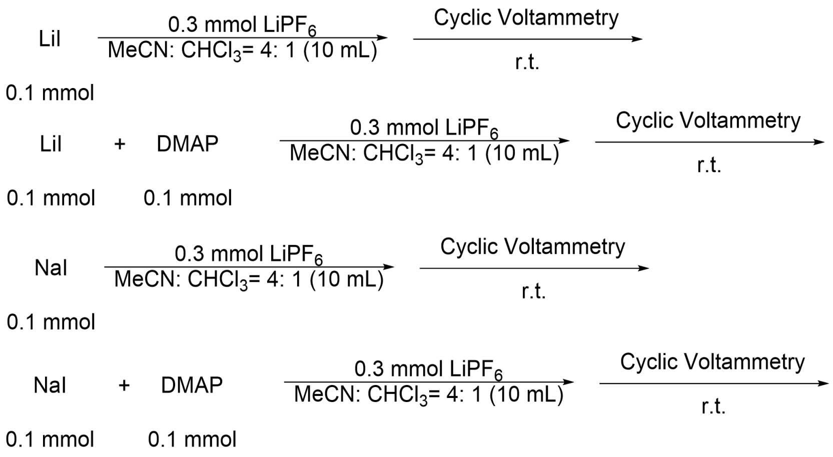

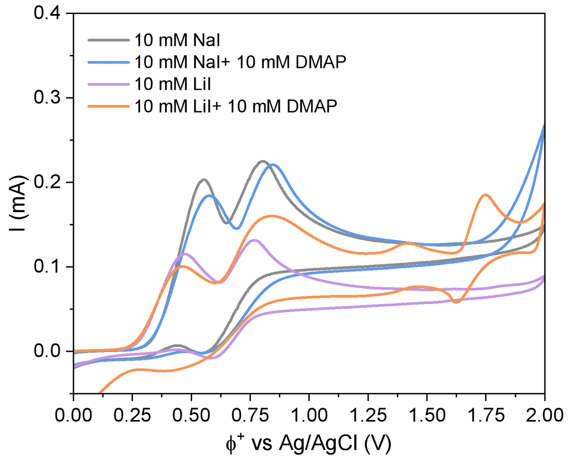

Fig. S28. Cyclic voltammetry curve for different iodides with DMAP

In an undivided cell equipped with a stir bar, $LiPF_6$ (45.3 mg, 0.30 mmol), LiI (13.4 mg, 0.10

mmol), DMAP (12.2 mg, 0.10 mmol) were added to the undivided cell. Solvents (MeCN: $CHCl_3$= 4: 1, 10 mL), were then added to the undivided cell. The undivided cell was equipped with glassy-carbon disk working electrode (diameter, 3.0 mm) and Pt wire auxiliary electrode. The scan range was 0 V to 2.0 V. All potentials are referenced against the Ag/AgCl reference electrode.
The NaI (15.0 mg, 0.10 mmol) was used instead of LiI, and did cyclic voltammetry as the same method.

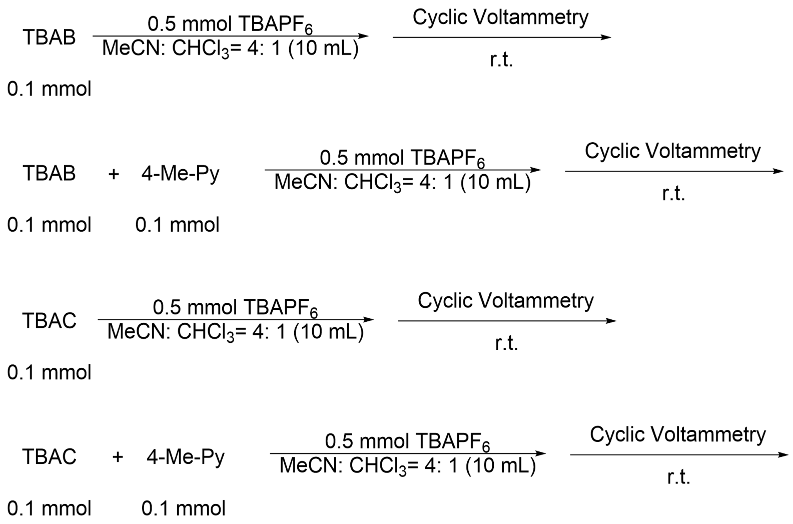

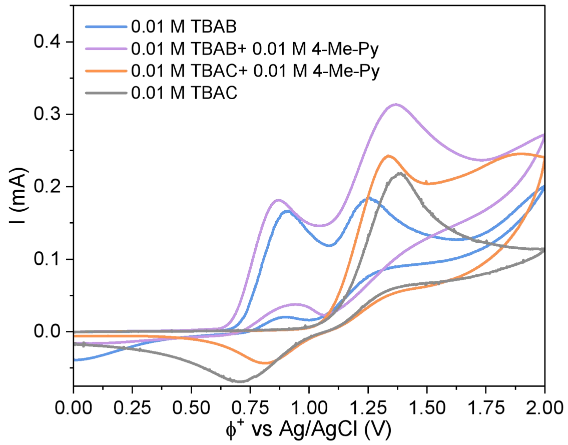

Fig. S29. Cyclic voltammetry curve for TBAB/ TBAC with 4-Me-Py

In an undivided cell equipped with a stir bar, $TBAPF_6$ (193.7 mg, 0.50 mmol), TBAB (32.2 mg, 0.10 mmol), 4-Me-Py (9.3 mg, 0.10 mmol) were added to the undivided cell. Solvents (MeCN: $CHCl_3$= 4: 1, 10 mL), were then added to the undivided cell. The undivided cell was equipped with glassy-carbon disk working electrode (diameter, 3.0 mm) and Pt wire auxiliary electrode. The scan range was 0 V to 2.0 V. All potentials are referenced against the Ag/AgCl reference electrode. The TBAC (27.8 mg, 0.10 mmol) was used instead of TBAB, and did cyclic voltammetry as the same method.

Density Functional Theory (DFT) Studies

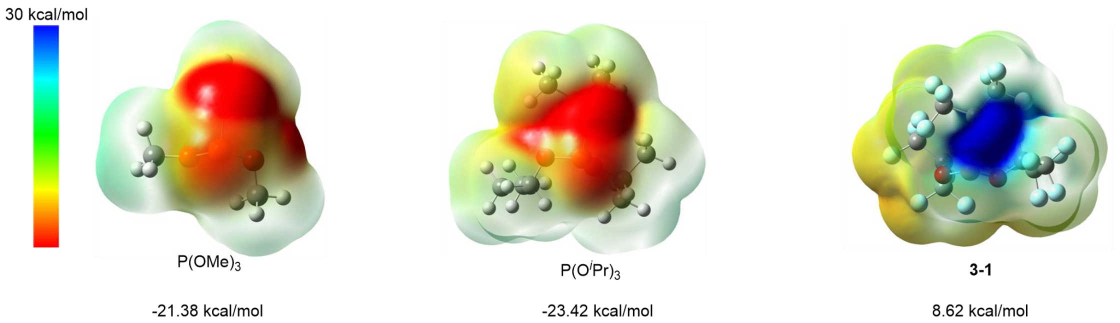

Fig. S30. DFT studies

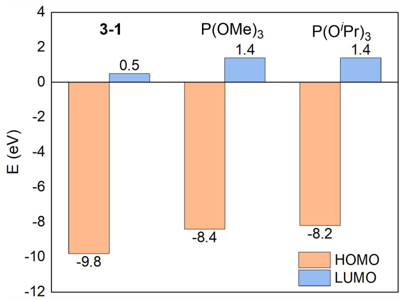

Fig. S31. Frontier orbitals of OPCs (III)

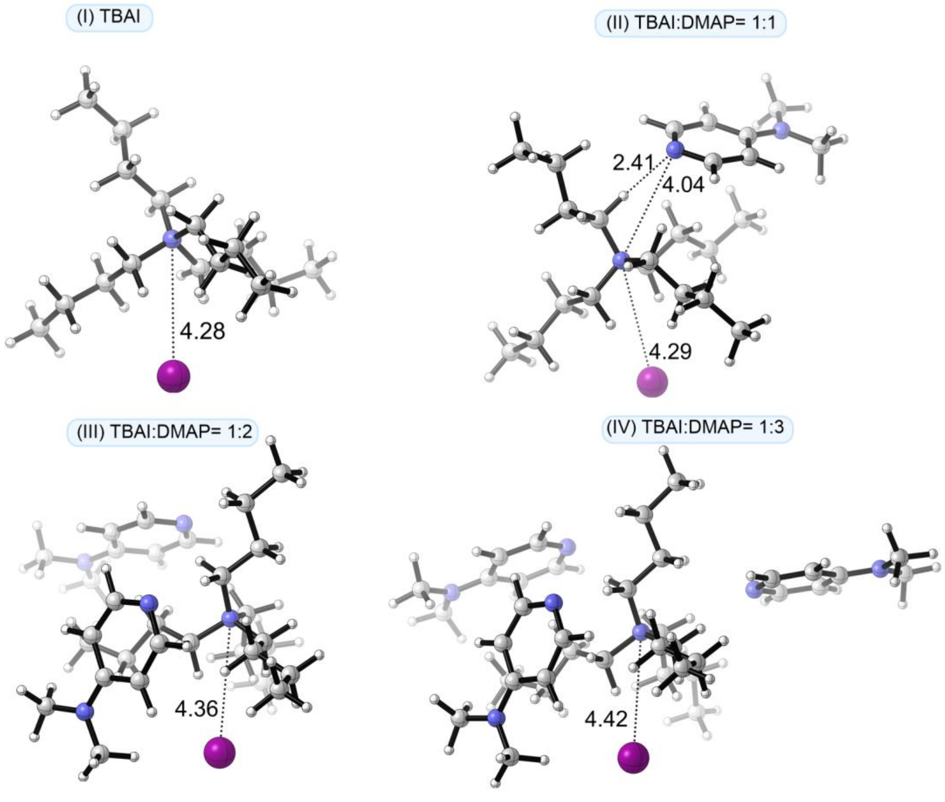

Fig. S32. Structure of TBAI-DMAP adduct

Theoretical calculations reveal that the pyridine nitrogen in DMAP can form a hydrogen bond with the N–CH_2 group of TBA^+, thereby increasing the N–I bond distance within TBAI. When TBAI forms hydrogen bonds with 1, 2, and 3 DMAP molecules, the N–I bond distance increases sequentially from 4.28 Å to 4.29, 4.36, and 4.42 Å, respectively. This interaction reduces the binding affinity of TBA^+ for I^-, making I^- more electron-rich, facilitating the oxidation of I^-.

Computational methods

All density functional theory (DFT) calculations were performed using the Gaussian 16 software package.[13] Geometries were optimized using the M06-2X[14] functional with a basis set of def2-SVP. Vibrational frequencies were calculated for all the stationary points to confirm if each optimized structure is a local minimum on the respective potential energy surface. Solvation energy corrections were calculated in acetonitrile solvent with the SMD continuum solvation model[15] based on the gas phase optimized geometries. The M06-2X functional with a basis set of def2-TZVP were used for single-point energy calculations, frontier orbital energies and Electrostatic Potential Surfaces. [16-18]The optimized transition state structures were plotted using CYLview. [19]

Cartesian coordinates (Å) and energies of optimized structures

$P(OMe)_3$

M06-2X SCF energy: -686.24516248 a.u.

M06-2X enthalpy: -686.104267 a.u.

M06-2X free energy: -686.151035 a.u.

M06-2X SCF energy in solution: -686.79787976 a.u.

M06-2X enthalpy in solution: -686.656984 a.u.

M06-2X free energy in solution: -686.703752 a.u.

Cartesian coordinates

ATOM	X	Y	Z
P	0.051144	-0.071078	0.757034
O	1.416285	0.352001	-0.056356
O	-0.753212	1.328062	0.570967
O	-0.578901	-0.998144	-0.469762
C	2.382102	-0.642801	-0.314575
H	3.283119	-0.144592	-0.692484
H	2.646556	-1.198671	0.601861
H	2.017890	-1.356553	-1.068939
C	-0.784503	2.007133	-0.676267

H	-1.616661	2.720376	-0.639149
H	0.158439	2.545418	-0.841750
H	-0.940882	1.298007	-1.502402
C	-1.737342	-1.755984	-0.201753
H	-1.811412	-2.547305	-0.958340
H	-1.695821	-2.224974	0.796420
H	-2.643311	-1.130987	-0.253955

3-1

M06-2X SCF energy: -2706.37765022 a.u.

M06-2X enthalpy: -2706.181885 a.u.

M06-2X free energy: -2706.276877 a.u.

M06-2X SCF energy in solution: -2709.33295522 a.u.

M06-2X enthalpy in solution: -2709.137190 a.u.

M06-2X free energy in solution: -2709.232182 a.u.

Cartesian coordinates

ATOM	X	Y	Z
P	0.026499	0.102177	1.539789
O	-1.196675	1.138760	1.228306
O	0.986036	0.419177	0.202391
O	-0.689325	-1.249193	0.885979
C	-0.137977	-2.100969	-0.065860
C	0.833583	-3.065755	0.611282
C	-1.309357	-2.786359	-0.761279
H	0.433718	-1.566751	-0.840571
C	-1.840459	1.210405	-0.016405
C	-3.308380	0.826064	0.174670
C	-1.637854	2.625409	-0.555848
H	-1.420670	0.511854	-0.757226
C	2.348667	0.678781	0.333178
C	3.030258	0.084861	-0.897586
C	2.587173	2.184241	0.483444
H	2.792411	0.192337	1.219677
F	1.531971	-3.748749	-0.279763
F	1.689121	-2.339622	1.349451
F	0.221162	-3.914398	1.419187
F	-2.117634	-3.384087	0.092936
F	-0.863186	-3.685235	-1.631302
F	-2.007716	-1.866367	-1.428639

F	2.885795	-1.241962	-0.879591
F	4.324280	0.368366	-0.896948
F	2.490904	0.532098	-2.019120
F	3.833679	2.411132	0.878935
F	1.757513	2.653811	1.412084
F	2.375821	2.842431	-0.643241
F	-3.386030	-0.378179	0.723485
F	-3.928040	0.795414	-0.999713
F	-3.940680	1.683602	0.962640
F	-0.336993	2.852054	-0.706443
F	-2.226292	2.758395	-1.739073
F	-2.125578	3.541284	0.264482

P(O^{i}Pr)$_3$

M06-2X SCF energy: -921.85898970 a.u.

M06-2X enthalpy: -921.541357 a.u.

M06-2X free energy: -921.605563 a.u.

M06-2X SCF energy in solution: -922.67621338 a.u.

M06-2X enthalpy in solution: -922.358581 a.u.

M06-2X free energy in solution: -922.422787 a.u.

Cartesian coordinates

ATOM	X	Y	Z
P	-0.070768	0.031434	-1.081798
O	0.631622	-1.412589	-0.832089
O	0.632188	0.983801	0.088279
O	-1.467677	-0.252377	-0.252703
C	-2.376776	0.828671	-0.041438
C	-3.762000	0.359557	-0.448275
C	-2.296689	1.263477	1.412411
H	-2.082165	1.679859	-0.686177
C	0.649257	-2.062478	0.451491
C	-0.406327	-3.154423	0.458818
C	2.052154	-2.598007	0.668345
H	0.413371	-1.312199	1.223724
C	1.706627	1.843829	-0.285545
C	1.692426	3.026154	0.665035
C	3.019164	1.075810	-0.253939
H	1.528923	2.205299	-1.316468

H	2.492334	3.737104	0.415169
H	0.726791	3.547284	0.612711
H	1.842009	2.675521	1.696720
H	3.858229	1.718714	-0.555742
H	2.977086	0.210542	-0.931581
H	3.207836	0.706535	0.765571
H	2.784213	-1.779729	0.662632
H	2.118784	-3.123071	1.631745
H	2.311039	-3.302594	-0.135374
H	-0.410768	-3.679174	1.425130
H	-0.192891	-3.883865	-0.336303
H	-1.396695	-2.716365	0.283950
H	-1.270247	1.569250	1.652679
H	-2.574081	0.421558	2.063972
H	-2.982564	2.100211	1.608634
H	-4.056144	-0.502842	0.167611
H	-3.767720	0.050042	-1.502026
H	-4.501888	1.160200	-0.308916

TBAI

M06-2X SCF energy: -982.89409573 a.u.

M06-2X enthalpy: -982.360682 a.u.

M06-2X free energy: -982.445126 a.u.

Cartesian coordinates

ATOM	X	Y	Z
N	1.196069	-0.348976	-0.001309
C	0.767458	1.024463	0.480878
C	0.745148	2.122711	-0.564075
H	1.435775	1.275772	1.315086
H	-0.259871	0.911823	0.857702
C	0.356989	3.440147	0.109117
H	1.715750	2.234210	-1.079569
H	-0.032231	1.894502	-1.309406
C	0.167276	4.564224	-0.900575
H	-0.587290	3.273024	0.652383
H	1.123453	3.717330	0.853372
H	-0.089768	5.507754	-0.400742
H	1.079714	4.732870	-1.493419

H	-0.651146	4.318029	-1.592411
C	2.614548	-0.336786	-0.499473
C	3.649456	0.265509	0.437179
H	2.605308	0.210779	-1.451904
H	2.871052	-1.381216	-0.722766
C	5.057566	0.072198	-0.126532
H	3.462506	1.342209	0.570864
H	3.594821	-0.199105	1.434691
C	6.127435	0.704679	0.755078
H	5.258249	-1.005393	-0.243342
H	5.104776	0.505220	-1.139280
H	7.130798	0.551190	0.336450
H	5.966381	1.787957	0.855168
H	6.113952	0.269783	1.765297
C	1.072621	-1.298543	1.170588
C	-0.351987	-1.679094	1.546634
H	1.561358	-0.797331	2.016843
H	1.667346	-2.190123	0.924140
C	-0.396197	-2.351257	2.917382
H	-1.019178	-0.801999	1.546768
H	-0.776439	-2.361528	0.793804
C	-1.811095	-2.791272	3.275559
H	0.287063	-3.218153	2.937795
H	-0.022821	-1.645264	3.677894
H	-1.852294	-3.231190	4.281073
H	-2.501697	-1.936557	3.240497
H	-2.180670	-3.541125	2.561052
C	0.287470	-0.775841	-1.133128
C	0.495261	-2.188587	-1.654785
H	0.454526	-0.054009	-1.942254
H	-0.744416	-0.612570	-0.779599
C	-0.650400	-2.543072	-2.605606
H	1.454478	-2.284170	-2.190254
H	0.511824	-2.923189	-0.834169
C	-0.509248	-3.940218	-3.195291
H	-1.603673	-2.448436	-2.061311
H	-0.693344	-1.795032	-3.413633
H	-1.343867	-4.171927	-3.870103
H	0.424601	-4.038503	-3.769200

H	-0.497311	-4.704277	-2.403557
I	-2.888189	0.934262	0.000141

TBAI-DMAP-1

M06-2X SCF energy: -1364.71476984 a.u.

M06-2X enthalpy: -1364.006219 a.u.

M06-2X free energy: -1364.114505 a.u.

Cartesian coordinates

ATOM	X	Y	Z
N	-0.828763	1.065338	0.109344
C	-2.321043	1.303302	0.207943
C	-3.046938	1.568310	-1.096512
H	-2.446736	2.138052	0.910811
H	-2.749582	0.389600	0.646138
C	-4.521022	1.846569	-0.799611
H	-2.607105	2.414172	-1.654267
H	-3.004902	0.664007	-1.722599
C	-5.347329	1.969052	-2.072968
H	-4.904904	1.008596	-0.195297
H	-4.612514	2.763260	-0.191861
H	-6.399007	2.189719	-1.845472
H	-4.966716	2.770440	-2.725234
H	-5.316768	1.024588	-2.635327
C	-0.095591	2.292342	-0.357087
C	-0.345087	3.564725	0.436185
H	-0.364116	2.437379	-1.412762
H	0.970694	2.041328	-0.298664
C	0.715723	4.607890	0.082817
H	-1.350283	3.965525	0.228644
H	-0.290385	3.366650	1.518015
C	0.481813	5.940548	0.782895
H	1.701479	4.199663	0.360691
H	0.729480	4.758049	-1.010077
H	1.263676	6.668580	0.528277
H	-0.488119	6.374220	0.497788
H	0.483638	5.815109	1.875752
C	-0.339013	0.689118	1.493006
C	-0.636389	-0.741350	1.916135

H	-0.830006	1.387311	2.183691
H	0.738287	0.905205	1.513917
C	-0.417563	-0.923226	3.416494
H	-1.668539	-1.032569	1.659307
H	0.011043	-1.444718	1.368731
C	-0.654483	-2.366825	3.844762
H	0.604621	-0.607966	3.691350
H	-1.103166	-0.256195	3.965063
H	-0.543418	-2.488649	4.930836
H	-1.665402	-2.691684	3.559519
H	0.058672	-3.043919	3.351841
C	-0.565260	-0.051029	-0.873739
C	0.890853	-0.472625	-1.007254
H	-0.940089	0.303039	-1.842007
H	-1.217762	-0.887612	-0.575527
C	0.971919	-1.830628	-1.705283
H	1.468402	0.268004	-1.584046
H	1.384948	-0.549233	-0.025172
C	2.409287	-2.315083	-1.843902
H	0.372975	-2.562496	-1.139402
H	0.496200	-1.761180	-2.696883
H	2.461827	-3.277287	-2.371571
H	3.018689	-1.583922	-2.398721
H	2.874524	-2.441292	-0.853749
I	-3.679172	-2.128155	-0.226781
C	3.656035	1.721257	-0.457582
C	4.474919	0.729676	-0.978784
C	4.760072	-0.413558	-0.195642
C	4.191372	-0.432003	1.100535
C	3.397575	0.630397	1.508583
H	3.424627	2.596019	-1.076165
H	4.873641	0.845600	-1.984538
H	4.363296	-1.258199	1.787242
H	2.963428	0.609498	2.514326
N	5.529798	-1.434908	-0.660774
C	6.109084	-1.353527	-1.983937
H	6.774911	-0.480430	-2.082221
H	6.699639	-2.255595	-2.174596
H	5.333198	-1.283110	-2.764226

C	5.770670	-2.590425	0.176118
H	4.828049	-3.088099	0.458642
H	6.382293	-3.312802	-0.374240
H	6.306279	-2.320373	1.101239
N	3.104610	1.695066	0.757601

TBAI-DMAP-2

M06-2X SCF energy: -1746.54132804 a.u.

M06-2X enthalpy: -1745.657639 a.u.

M06-2X free energy: -1745.787811 a.u.

Cartesian coordinates

ATOM	X	Y	Z
N	-0.232612	-1.693127	-0.208554
C	-1.610679	-2.264679	-0.463365
C	-2.656149	-1.960644	0.586967
H	-1.470459	-3.347929	-0.584687
H	-1.951873	-1.831931	-1.414748
C	-3.982837	-2.612680	0.201537
H	-2.341407	-2.296974	1.589941
H	-2.822842	-0.874708	0.615131
C	-5.104669	-2.194189	1.143043
H	-4.234438	-2.293558	-0.822904
H	-3.876644	-3.711311	0.191882
H	-6.055578	-2.674515	0.874996
H	-4.874146	-2.460397	2.186775
H	-5.252097	-1.104924	1.087043
C	0.351941	-2.195876	1.085143
C	0.407827	-3.705143	1.244181
H	-0.231989	-1.734857	1.896037
H	1.373114	-1.795887	1.120231
C	1.265981	-4.058235	2.459476
H	-0.604656	-4.122592	1.368729
H	0.851699	-4.178684	0.353567
C	1.308507	-5.556775	2.730494
H	2.284344	-3.675640	2.280704
H	0.874543	-3.528067	3.343474
H	1.941123	-5.788451	3.598006
H	0.302019	-5.951895	2.933449

H	1.713687	-6.102037	1.864984
C	0.645085	-2.106502	-1.365246
C	0.437294	-1.311350	-2.647303
H	0.433529	-3.168396	-1.546902
H	1.684035	-2.032978	-1.018118
C	1.152484	-1.983470	-3.817770
H	-0.633724	-1.195892	-2.881395
H	0.829358	-0.289750	-2.525642
C	1.035782	-1.172276	-5.102600
H	2.216243	-2.131607	-3.562347
H	0.730877	-2.990308	-3.973104
H	1.533558	-1.675515	-5.942579
H	-0.019012	-1.018739	-5.371862
H	1.492720	-0.178933	-4.982633
C	-0.320399	-0.188119	-0.124276
C	1.009339	0.541604	0.018058
H	-0.960450	0.036972	0.738083
H	-0.878714	0.143759	-1.010192
C	0.837698	1.978269	-0.475893
H	1.332229	0.550190	1.071348
H	1.822430	0.059403	-0.551759
C	2.050231	2.848638	-0.174813
H	0.635512	1.963026	-1.559686
H	-0.070923	2.408912	-0.023676
H	1.918730	3.872139	-0.554930
H	2.224499	2.909369	0.912657
H	2.963171	2.432801	-0.631242
I	-3.397328	0.748121	-1.955683
C	3.887222	-0.840639	1.504677
C	4.485771	0.387259	1.258892
C	4.970113	0.672211	-0.038776
C	4.820166	-0.358868	-0.996319
C	4.217756	-1.551051	-0.618991
H	3.493764	-1.048212	2.506270
H	4.558505	1.112922	2.066413
H	5.173519	-0.243755	-2.018861
H	4.114953	-2.349918	-1.361663
N	5.539920	1.869895	-0.351474
C	5.690652	2.881729	0.671715

H	6.308747	2.520304	1.509767
H	6.182419	3.759758	0.240079
H	4.714419	3.198834	1.074607
C	5.977200	2.124144	-1.706939
H	5.142969	2.037728	-2.423022
H	6.375664	3.141954	-1.771907
H	6.770567	1.424791	-2.018690
N	3.736341	-1.808955	0.598896
C	-2.499353	0.312648	2.913322
C	-2.768965	1.480207	2.215336
C	-1.754133	2.459014	2.088193
C	-0.527197	2.161324	2.730138
C	-0.372776	0.935601	3.366148
H	-3.293260	-0.434098	3.012986
H	-3.745722	1.613966	1.754048
H	0.307982	2.860031	2.712542
H	0.593031	0.697758	3.827393
N	-1.945280	3.611827	1.391473
C	-3.207298	3.841991	0.708247
H	-4.037021	3.996728	1.420178
H	-3.113227	4.737537	0.084472
H	-3.448078	2.996580	0.044149
C	-0.960006	4.665116	1.474565
H	0.011106	4.350659	1.056649
H	-1.309204	5.528098	0.897868
H	-0.791800	4.988994	2.516527
N	-1.319648	0.000825	3.461907

TBAI-DMAP-3

M06-2X SCF energy: -2128.35653589 a.u.

M06-2X enthalpy: -2127.297969 a.u.

M06-2X free energy: -2127.456747 a.u.

Cartesian coordinates

ATOM	X	Y	Z
N	-0.490515	0.179466	0.078901
C	-1.405666	-0.976551	-0.264044
C	-0.997776	-1.800134	-1.467416
H	-2.407984	-0.540445	-0.380732

H	-1.403077	-1.643210	0.609516
C	-2.015809	-2.914951	-1.699169
H	-0.891745	-1.184144	-2.376636
H	-0.032570	-2.281535	-1.255656
C	-1.549287	-3.880197	-2.781059
H	-2.137207	-3.468586	-0.754048
H	-2.998349	-2.482841	-1.959750
H	-2.289376	-4.671558	-2.963590
H	-1.368526	-3.358947	-3.734670
H	-0.612196	-4.363356	-2.464719
C	-0.322044	1.112963	-1.089937
C	-1.611240	1.591154	-1.734849
H	0.309464	0.590388	-1.823855
H	0.236419	1.976446	-0.706168
C	-1.309914	2.727889	-2.711110
H	-2.093927	0.762556	-2.279570
H	-2.332596	1.937910	-0.977727
C	-2.565916	3.231300	-3.411962
H	-0.825317	3.548959	-2.156823
H	-0.577775	2.377610	-3.458621
H	-2.341306	4.058173	-4.099593
H	-3.040801	2.428205	-3.995797
H	-3.304666	3.590661	-2.679746
C	-1.119172	0.924655	1.232083
C	-1.016388	0.218584	2.578065
H	-2.174275	1.073198	0.960845
H	-0.638146	1.912571	1.272166
C	-1.967015	0.858139	3.588366
H	-1.253641	-0.853009	2.486758
H	0.015013	0.267391	2.962275
C	-1.828421	0.251049	4.979146
H	-1.781548	1.945275	3.632772
H	-3.000987	0.735765	3.226218
H	-2.533819	0.704537	5.688958
H	-2.019472	-0.831507	4.953288
H	-0.811739	0.397123	5.372843
C	0.865283	-0.358934	0.460491
C	1.874377	0.670100	0.948290
H	1.258595	-0.877035	-0.424277

H	0.693083	-1.141815	1.210665
C	2.984749	-0.057783	1.707347
H	2.307232	1.230741	0.102563
H	1.414792	1.415337	1.618219
C	4.138876	0.863293	2.079400
H	2.556243	-0.516087	2.613803
H	3.342905	-0.906393	1.101705
H	4.909459	0.333650	2.658590
H	4.614758	1.287131	1.178936
H	3.781667	1.710698	2.686505
I	0.530182	-3.980266	1.184036
C	2.284033	3.619257	-1.333372
C	3.652212	3.529853	-1.112270
C	4.166087	3.835825	0.168960
C	3.216937	4.235273	1.137789
C	1.873297	4.282472	0.791814
H	1.887010	3.368861	-2.324058
H	4.302981	3.225545	-1.929964
H	3.513468	4.495580	2.151933
H	1.141106	4.583717	1.548481
N	5.496652	3.742749	0.454584
C	6.414873	3.272619	-0.558289
H	6.426815	3.935299	-1.439257
H	7.427162	3.242477	-0.141741
H	6.155804	2.256153	-0.901806
C	5.971597	4.075260	1.779665
H	5.537184	3.412717	2.546874
H	7.060637	3.963827	1.810312
H	5.727497	5.116613	2.046185
N	1.388207	3.979265	-0.412794
C	-5.005587	0.598719	-0.803887
C	-6.372623	0.430768	-0.968156
C	-7.251821	0.892519	0.038715
C	-6.643026	1.509522	1.156315
C	-5.260541	1.616704	1.203412
H	-4.329581	0.240729	-1.588389
H	-6.740523	-0.056018	-1.868688
H	-7.232360	1.900855	1.982810
H	-4.794468	2.095051	2.071070

N	-8.603862	0.751174	-0.062276
C	-9.171969	0.100105	-1.222018
H	-8.813864	-0.937826	-1.322955
H	-10.262371	0.075752	-1.123531
H	-8.924834	0.637944	-2.152177
C	-9.456466	1.231080	1.002292
H	-9.347377	2.317952	1.152411
H	-10.501615	1.027095	0.746699
H	-9.234367	0.730721	1.959529
N	-4.433160	1.174077	0.255153
C	1.950478	-1.895619	-2.887767
C	2.743688	-2.609275	-2.002106
C	3.791177	-1.943101	-1.322202
C	3.945732	-0.568170	-1.623188
C	3.062325	0.036619	-2.509105
H	1.146664	-2.421146	-3.412647
H	2.526091	-3.659696	-1.817978
H	4.720826	0.035478	-1.152212
H	3.169432	1.108233	-2.710345
N	4.598226	-2.584280	-0.432390
C	4.372490	-3.988607	-0.133753
H	4.611041	-4.635274	-0.996737
H	5.013618	-4.278397	0.706067
H	3.326764	-4.156555	0.169255
C	5.783715	-1.919779	0.055672
H	5.535606	-1.024169	0.649406
H	6.338879	-2.607026	0.703021
H	6.448337	-1.606425	-0.769074
N	2.068553	-0.587242	-3.141753

Experimental procedures for the NMR tests

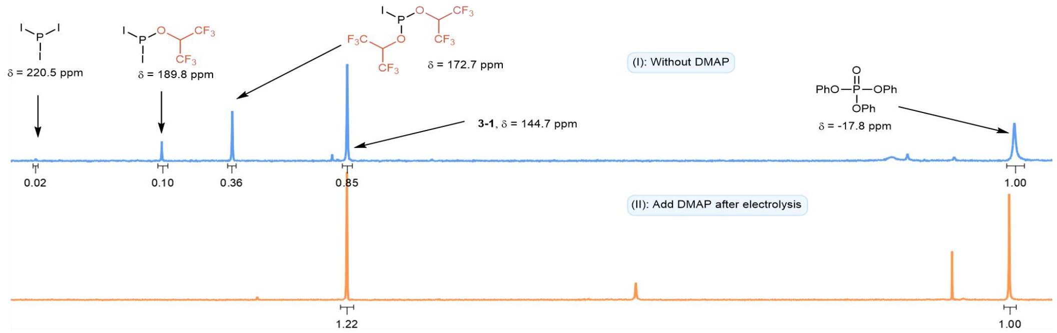

Fig. S33. ^{31}P NMR Chemical shift with/without DMAP

In a 25 mL oven-dried two-necked undivided cell equipped with a stir bar, $^{n}Bu_4NI$ (221.6 mg, 0.6 mmol) was added into the tube. The tube was equipped with carbon cloth (1.5 × 1.5 cm^2) as the anode and Ni foam (2.0 × 1.5 cm^2) as the cathode. This was followed by the addition of P_4 (93.0 mg, 0.75 mmol) and LiCl (50.4 mg, 1.2 mmol) in a glovebox (H_2O and O_2 < 0.1 ppm). Then MeCN (4 mL), $CHCl_3$ (3 mL) and HFIP (2.5 mL, 24 mmol) were added to the tube through a syringe. The mixtures were stirred at a constant current of 100 mA at room temperature for 2.5 h (J = 44.4 mA/cm^2, 3 F/mol). At the end of the reaction, the triphenyl phosphate (326.3 mg, 1.0 mmol) was added as an internal standard and stirred for 5 min and the yield was determined by assure ^{31}P NMR. Then DMAP (366.5 mg, 3.0 mmol) was added into the tube and stirred for 10 min and the yield was determined by assure ^{31}P NMR.

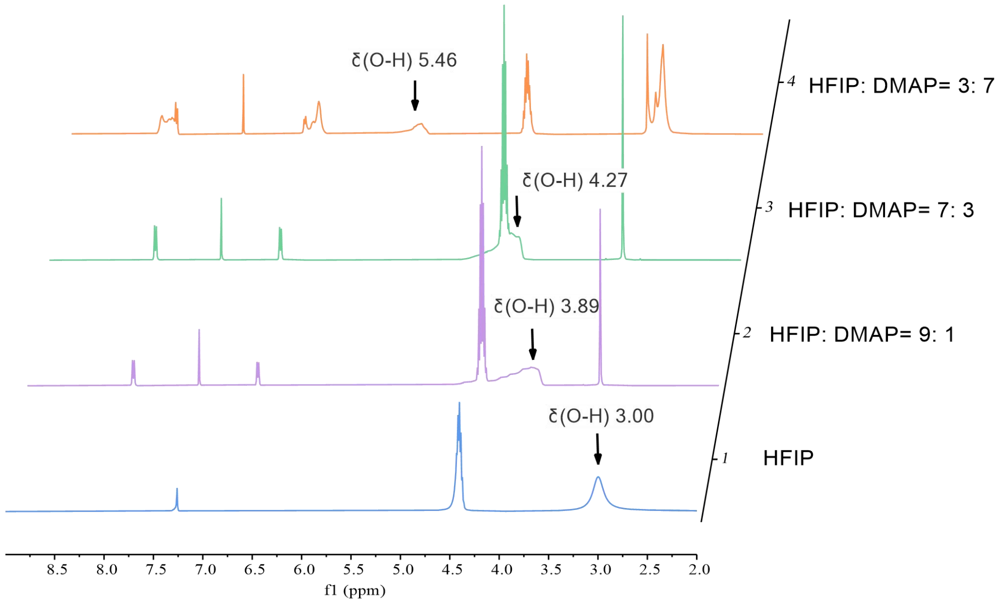

Fig. S34. ^{1}H NMR chemical shift of HFIP (O-H) with DMAP

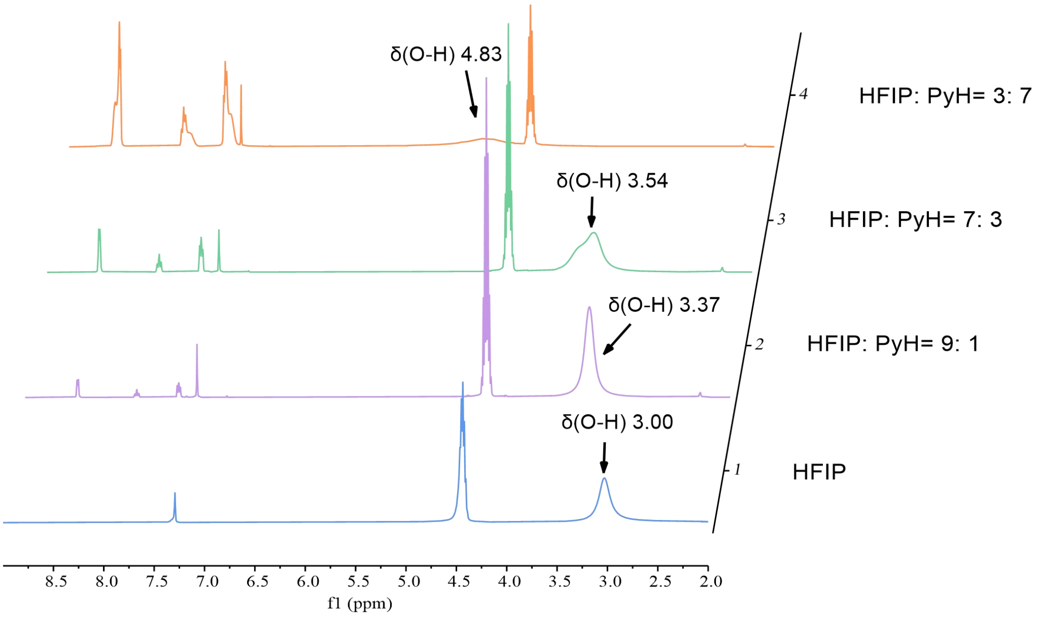

Fig. S35. ^{1}H NMR chemical shift of HFIP (O-H) with Pyridine

The HFIP (588.1 mg, 3.5 mmol) was dissolved in 10 mL $CDCl_3$, which called Solution A. The DMAP (427.6 mg, 3.5 mmol) was dissolved in 10 mL $CDCl_3$, which called Solution B. Different ratios of A and B solution were mixed into an NMR tube, and made ^{1}H NMR test. 1) 1000 μL of A. 2) 900 μL of A, 100 μL of B. 3) 700 μL of A, 300 μL of B. 4) 300 μL of A, 700 μL of B.

The Pyridine (276.9 mg, 3.5 mmol) was used instead of DMAP, and did NMR tests as the same method.

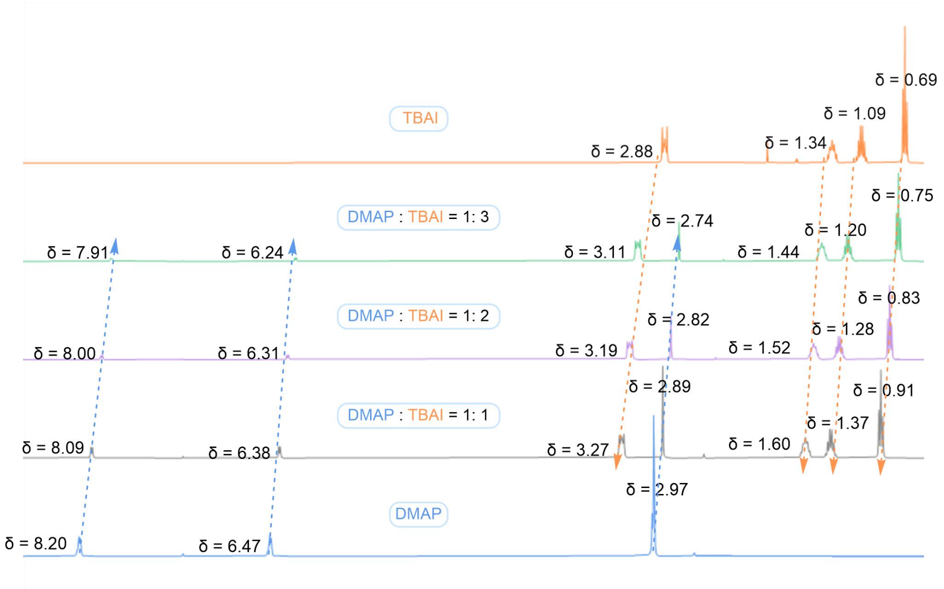

Fig. S36. ^{1}H NMR chemical shift of DMAP ($N\text{-}CH_3$) with TBAI

The DMAP (24.4 mg, 0.2 mmol) was dissolved in 1 mL $CDCl_3$ in an NMR tube, then different equivalents of TBAI was added into the NMR tube, and made ^{1}H NMR test. 1) No TBAI. 2) 1 equiv. TBAI (73.9 mg, 0.2 mmol). 3) 2 equiv. TBAI (147.7 mg, 0.4 mmol). 4) 3 equiv. TBAI (221.6 mg, 0.6 mmol). 5) TBAI (73.9 mg, 0.2 mmol) instead of DMAP.

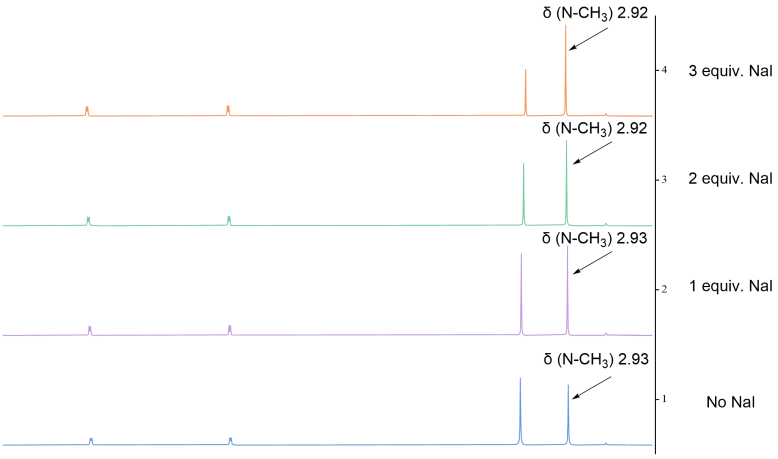

Fig. S37. ^{1}H NMR chemical shift of DMAP (N-CH$_3$) with NaI

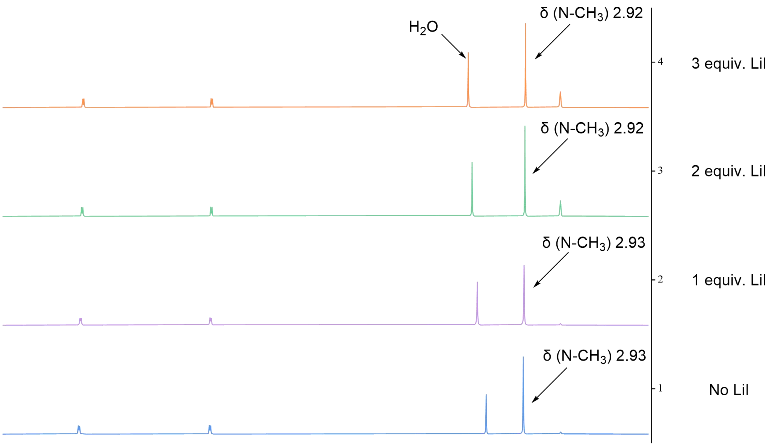

Fig. S38. ^{1}H NMR chemical shift of DMAP (N-CH$_3$) with LiI

The DMAP (24.4 mg, 0.2 mmol) was dissolved in 1 mL DMSO in an NMR tube, then different equivalents of NaI was added into the NMR tube, and made ^{1}H NMR test. 1) No NaI. 2) 1 equiv. NaI (30.0 mg, 0.2 mmol). 3) 2 equiv. NaI (60.0 mg, 0.4 mmol). 4) 3 equiv. NaI (89.9 mg, 0.6 mmol).

LiI was used instead of NaI, and did NMR tests as the same method.

Experimental Procedures for Anode potential monitoring

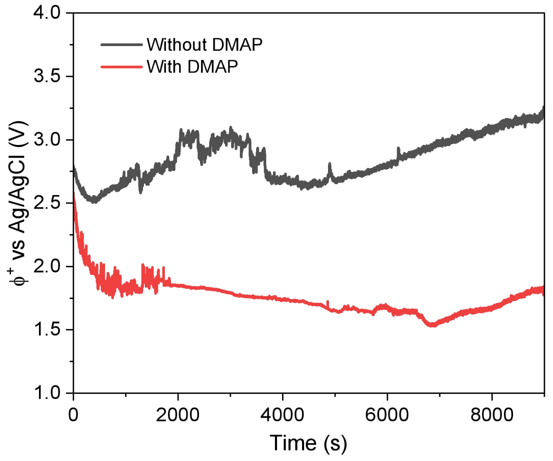

Fig. S39. Anode potential with/without DMAP

In a 10 mL oven-dried two-necked undivided cell equipped with a stir bar, ^{n}Bu$_4$NI (221.6 mg, 0.6 mmol), DMAP (366.5 mg, 3.0 mmol) were added into the beaker. The tube was equipped with carbon cloth (1.5 × 1.5 cm^2) as the anode and Ni foam (2.0 × 1.5 cm^2) as the cathode and Ag/AgCl as the reference electrode. This was followed by the addition of P_4 (93.0 mg, 0.75 mmol), LiCl (50.9 mg, 1.2 mmol) in a glovebox (H_2O and O_2 < 0.1 ppm). Then MeCN (4 mL), $CHCl_3$ (3 mL) and HFIP (2.5 mL, 24 mmol) were added to the tube through a syringe. The mixtures were stirred at a constant current of 100 mA at room temperature for 2.5 h (J = 44.4 mA/cm^2, 3 F/mol). All potentials are referenced against the Ag/AgCl reference electrode.

Detailed Descriptions for Products

The products obtained by using primary alcohol and alkyl Grignard reagent were easily oxidized or hydrolyzed in the air, and it was difficult to separate the pure products during the separation process such as flash chromatography. In order to ensure the purity of these products, we added S_8 (16.0 mg, 0.5 mmol) to the tube at the end of reaction to avoid oxidation and the mixtures were stirred for 30 min. Then the desired products were obtained after purification by flash chromatography on 200-300 silica gel (petroleum: EtOAc = 10: 1).

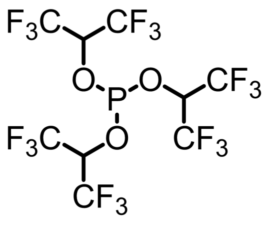

Tris(1,1,1,3,3,3-hexafluoropropan-2-yl) phosphite (3-1): colorless oil was obtained. ^{1}H NMR (400 MHz, Chloroform-*d*) δ 4.91 – 4.79 (dp, *J* = 10.3, 5.2 Hz, 3H). ^{13}C NMR (101 MHz, Chloroform-d) δ 120.12 (q, *J* = 282.2 Hz), 69.84 (td, *J* = 35.6, 8.7 Hz). ^{31}P NMR (162 MHz, Chloroform-*d*) δ 139.70. ^{19}F NMR (377 MHz, Chloroform-*d*) δ -74.22. HRMS (ESI) calcd for $C_9H_4F_{18}O_3P$ $[M+H]^+$: 532.9605 found: 532.9648.

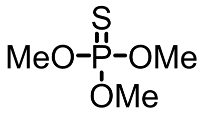

***O,O,O*-trimethylthiophosphate (4):** colorless oil was obtained. ^{1}H NMR (400 MHz, Chloroform-*d*) δ 3.77 – 3.70 (d, *J* = 13.4 Hz, 9H). ^{13}C NMR (101 MHz, Chloroform-*d*) δ 54.58 (d, *J* = 5.5 Hz). ^{31}P NMR (162 MHz, Chloroform-*d*) δ 72.87. HRMS (ESI) calcd for $C_3H_{10}O_3PS$ $[M+H]^+$: 157.0083 found: 157.0083.

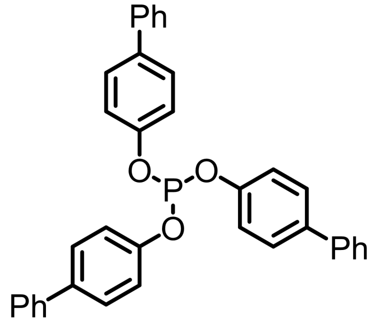

Tri([1,1'-biphenyl]-4-yl) phosphite (5)(*4*): white solid was obtained. ^{1}H NMR (400 MHz, Chloroform-*d*) δ 7.64 – 7.56 (m, 12H), 7.51 – 7.42 (m, 6H), 7.40 – 7.33 (m, 3H), 7.33 – 7.27 (dd, *J* = 8.7, 1.0 Hz, 6H). ^{13}C NMR (101 MHz, Chloroform-*d*) δ 150.97 (d, *J* = 3.3 Hz), 140.31, 137.40, 128.78, 128.42, 127.17, 126.93, 120.95 (d, *J* = 6.9 Hz). ^{31}P NMR (162 MHz, Chloroform-*d*) δ 127.38.

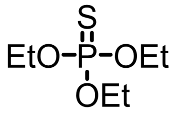

***O,O,O*-triethyl phosphorothioate (6):** colorless oil was obtained. ^{1}H NMR (400 MHz, Chloroform-*d*) δ 4.15 – 4.02 (dq, *J* = 9.5, 7.1 Hz, 6H), 1.35 – 1.25 (td, *J* = 7.1, 0.9 Hz, 9H). ^{13}C NMR

(101 MHz, Chloroform-*d*) δ 64.07 (d, *J* = 5.5 Hz), 15.81 (d, *J* = 7.6 Hz). ^{31}P NMR (162 MHz, Chloroform-*d*) δ 67.53. HRMS (ESI) calcd for $C_6H_{16}O_3PS$ $[M+H]^+$: 199.0552 found: 199.0548.

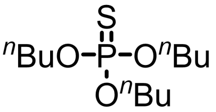

***O,O,O*-tributyl phosphorothioate (7):** colorless oil was obtained. ^{1}H NMR (400 MHz, Chloroform-*d*) δ 4.08 – 3.97 (dt, *J* = 8.7, 6.5 Hz, 6H), 1.73 – 1.58 (dq, *J* = 8.3, 6.6 Hz, 6H), 1.46 – 1.32 (m, 6H), 0.96 – 0.88 (t, *J* = 7.4 Hz, 9H). ^{13}C NMR (101 MHz, Chloroform-*d*) δ ^{13}C NMR (101 MHz, Chloroform-*d*) δ 67.85 (d, *J* = 6.0 Hz), 32.00, 18.68, 13.54. ^{31}P NMR (162 MHz, Chloroform-*d*) δ 68.01. HRMS (ESI) calcd for $C_{12}H_{28}O_3PS$ $[M+H]^+$: 283.1491 found: 283.1490.

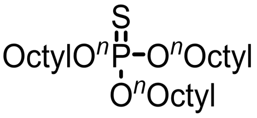

Trioctylthiophosphate (8): colorless oil was obtained. ^{1}H NMR (400 MHz, Chloroform-*d*) δ 4.07 – 3.97 (dt, *J* = 8.8, 6.6 Hz, 6H), 1.71 – 1.59 (m, 6H), 1.49 – 1.10 (m, 30H), 0.91 – 0.83 (m, 9H). ^{13}C NMR (101 MHz, Chloroform-*d*) δ 68.18 (d, *J* = 6.0 Hz), 31.74, 30.04 (d, *J* = 7.4 Hz), 29.14, 29.09, 25.49, 22.59, 14.02. ^{31}P NMR (162 MHz, Chloroform-*d*) δ 68.01. HRMS (ESI) calcd for $C_{24}H_{52}O_3PS$ $[M+H]^+$: 451.3369 found: 451.3365.

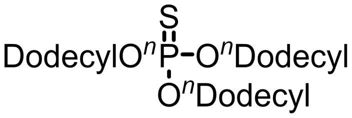

Thiophosphoric acid tris(dodecanyl) ester (9): colorless oil was obtained. ^{1}H NMR (400 MHz, Chloroform-*d*) δ 4.07 – 3.97 (dt, *J* = 9.0, 6.7 Hz, 6H), 1.72 – 1.62 (q, *J* = 7.0 Hz, 6H), 1.41 – 1.17 (d, *J* = 6.8 Hz, 54H), 0.91 – 0.80 (t, *J* = 6.7 Hz, 9H). ^{13}C NMR (101 MHz, Chloroform-*d*) δ 68.22 (d, *J* = 5.9 Hz), 31.91, 30.07 (d, *J* = 7.5 Hz), 29.65, 29.63, 29.58, 29.53, 29.34, 29.17, 25.52, 22.67, 14.09. ^{31}P NMR (162 MHz, Chloroform-*d*) δ 68.01. HRMS (ESI) calcd for $C_{36}H_{76}O_3PS$ $[M+H]^+$: 619.5248 found: 619.5245.

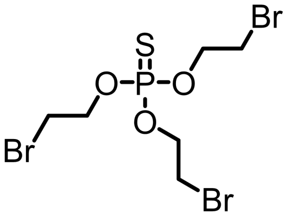

Thiophosphoric acid *O,O,O*-tris-(2-bromo-ethyl ester) (10): colorless oil was obtained. ^{1}H NMR (400 MHz, Chloroform-*d*) δ 4.42 – 4.30 (dt, *J* = 9.8, 6.1 Hz, 6H), 3.58 – 3.50 (t, *J* = 6.1 Hz, 6H). ^{13}C NMR (101 MHz, Chloroform-*d*) δ 67.38 (d, *J* = 5.0 Hz), 29.33 (d, *J* = 8.5 Hz). ^{31}P NMR (162 MHz, Chloroform-*d*) δ 66.83. HRMS (ESI) calcd for $C_6H_{13}Br_3O_3PS$ $[M+H]^+$: 432.7868 found: 432.7874.

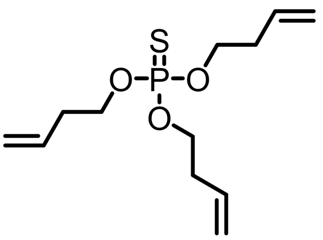

Thiophosphoric acid *O,O,O*-tris-(tribut-3-enyl ester) (11): colorless oil was obtained. ^{1}H NMR (400 MHz, Chloroform-*d*) δ 5.85 – 5.71 (ddt, *J* = 17.0, 10.3, 6.7 Hz, 3H), 5.18 – 5.04 (m, 6H), 4.13 – 4.03 (dt, *J* = 9.2, 6.8 Hz, 6H), 2.47 – 2.38 (qd, *J* = 6.8, 3.4 Hz, 6H). ^{13}C NMR (101 MHz, Chloroform-*d*) δ 133.43, 117.56, 67.10, 34.36 (d, *J* = 7.7 Hz). ^{31}P NMR (162 MHz, Chloroform-*d*) δ 67.77. HRMS (ESI) calcd for $C_{12}H_{22}O_3PS$ $[M+H]^+$: 277.1022 found: 277.1018.

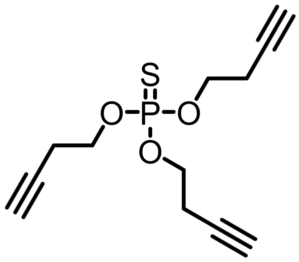

Thiophosphoric acid *O,O,O*-tris-(3-butynyl ester) (12): colorless oil was obtained. ^{1}H NMR (400 MHz, Chloroform-*d*) δ 4.20 – 4.06 (m, 6H), 2.62 – 2.50 (dtt, *J* = 10.2, 6.7, 2.8 Hz, 6H), 2.04 – 1.95 (dq, *J* = 12.1, 2.6 Hz, 3H). ^{13}C NMR (101 MHz, Chloroform-*d*) δ 79.32, 70.36, 65.74 (d, *J* = 5.2 Hz), 20.32 (d, *J* = 8.3 Hz). ^{31}P NMR (162 MHz, Chloroform-*d*) δ 67.18. HRMS (ESI) calcd for $C_{12}H_{16}O_3PS$ $[M+H]^+$: 271.0552 found: 271.0550.

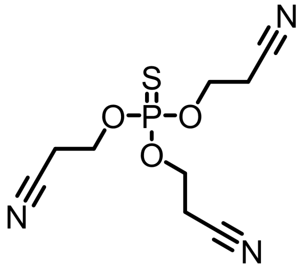

Thiophosphoric acid *O,O,O*-tris-(2-cyanoethyl ester) (13): colorless oil was obtained. ^{1}H NMR (400 MHz, Chloroform-*d*) δ 3.88 – 3.80 (t, *J* = 6.1 Hz, 4H), 2.65 – 2.55 (t, *J* = 6.1 Hz, 4H). ^{13}C NMR (101 MHz, Chloroform-*d*) δ 118.40, 57.51, 21.39. ^{31}P NMR (162 MHz, Chloroform-*d*) δ 68.70. HRMS (ESI) calcd for $C_9H_{13}N_3O_3PS$ $[M+H]^+$: 274.0410 found: 274.0411.

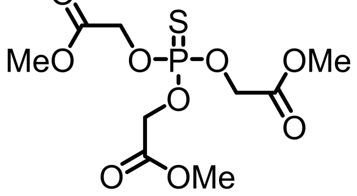

Tris-(glycolic acid methyl ester) phosphite (14): colorless oil was obtained. ^{1}H NMR (400 MHz, Chloroform-*d*) δ 4.76 – 4.59 (m, 6H), 4.12 – 3.66 (m, 9H). ^{13}C NMR (101 MHz, Chloroform-*d*) δ 168.19 (d, *J* = 7.3 Hz), 64.04 (d, *J* = 4.0 Hz), 53.72 (d, *J* = 277.9 Hz). ^{31}P NMR (162 MHz, Chloroform-*d*) δ 71.49. HRMS (ESI) calcd for $C_9H_{16}O_9PS$ $[M+H]^+$: 331.0247 found: 331.0246.

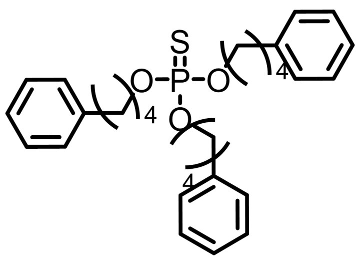

Tris-(4-phenyl-1-butyl) phosphite (15): colorless oil was obtained. ^{1}H NMR (400 MHz, Chloroform-*d*) δ 7.41 – 7.33 (t, *J* = 7.4 Hz, 6H), 7.32 – 7.24 (m, 9H), 4.22 – 4.11 (dt, *J* = 8.5, 5.6 Hz, 6H), 2.79 – 2.69 (t, *J* = 7.0 Hz, 6H), 1.86 – 1.74 (m, *J* = 3.0 Hz, 12H). ^{13}C NMR (101 MHz, Chloroform-*d*) δ 141.80, 128.26, 128.21, 125.71, 67.84 (d, *J* = 5.8 Hz), 35.16, 29.45 (d, *J* = 7.7 Hz), 27.17. ^{31}P NMR (162 MHz, Chloroform-*d*) δ 68.31. HRMS (ESI) calcd for $C_{30}H_{40}O_3PS$ [M+H]$^+$: 511.2430 found: 511.2443.

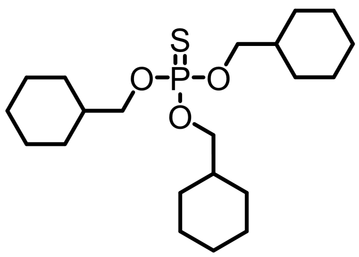

Tris-(cyclohexylmethyl) phosphite (16): colorless oil was obtained. ^{1}H NMR (400 MHz, Chloroform-*d*) δ 3.84 – 3.76 (m, 6H), 1.80 – 1.68 (m, 12H), 1.68 – 1.59 (m, 6H), 1.40 – 1.10 (m, 9H), 1.02 – 0.87 (qd, *J* = 13.3, 12.5, 3.8 Hz, 6H). ^{13}C NMR (101 MHz, Chloroform-*d*) δ 73.02 (d, *J* = 6.3 Hz), 38.13 (d, *J* = 7.6 Hz), 29.26, 26.28, 25.52. ^{31}P NMR (162 MHz, Chloroform-*d*) δ 68.27. HRMS (ESI) calcd for $C_{30}H_{40}O_3PS$ [M+H]$^+$: 403.2430 found: 403.2424.

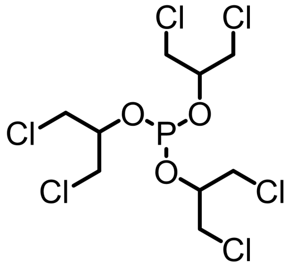

Tris-(1,3-dichlor-isopropyl) phosphite (17): colorless oil was obtained. ^{1}H NMR (400 MHz, Chloroform-*d*) δ 4.66 – 4.54 (dp, *J* = 8.5, 5.2 Hz, 3H), 3.79 – 3.73 (d, *J* = 5.3 Hz, 12H). ^{13}C NMR (101 MHz, Chloroform-*d*) δ 72.54 (d, *J* = 9.6 Hz), 44.45 (d, *J* = 2.8 Hz). ^{31}P NMR (162 MHz, Chloroform-*d*) δ 140.48. HRMS (ESI) calcd for $C_9H_{16}Cl_6O_3P$ [M+H]$^+$: 412.8963 found: 412.8959.

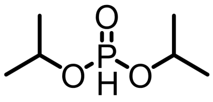

Diisopropyl phosphite (18)(*2*): colorless oil was obtained. ^{1}H NMR (400 MHz, Chloroform-*d*) δ 7.71 – 5.80 (d, *J* = 687.9 Hz, 1H), 4.77 – 4.61 (dhept, *J* = 8.5, 6.2 Hz, 2H), 1.34 – 1.29 (dd, *J* = 6.3, 2.4 Hz, 12H). ^{13}C NMR (101 MHz, Chloroform-*d*) δ 70.78 (d, *J* = 5.6 Hz), 23.93 (d, *J* = 4.7 Hz), 23.73 (d, *J* = 4.8 Hz). ^{31}P NMR (162 MHz, Chloroform-*d*) δ 4.47.

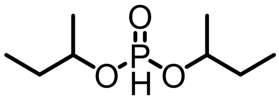

Di-sec-butyl phosphonate (19)(*3*): colorless oil was obtained. ^{1}H NMR (400 MHz, Chloroform-*d*) δ 7.76 – 5.88 (d, *J* = 688.0 Hz, 1H), 4.56 – 4.43 (ttd, *J* = 12.6, 6.3, 2.1 Hz, 2H), 1.75 – 1.51 (m, 4H), 1.36 – 1.29 (m, 6H), 0.98 – 0.88 (td, *J* = 7.4, 1.6 Hz, 6H). ^{13}C NMR (101 MHz, Chloroform-*d*) δ 75.73 – 75.53 (m), 30.53 – 30.30 (m), 21.73 – 21.15 (m), 9.88 – 9.28 (m). ^{31}P NMR (162 MHz, Chloroform-*d*) δ 6.10 – 4.73 (m).

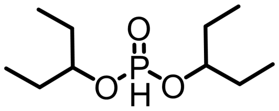

Bis(1-ethylpropyl) phosphonate (20): colorless oil was obtained. ^{1}H NMR (400 MHz, Chloroform-*d*) δ 7.82 – 5.97 (d, *J* = 687.8 Hz, 1H), 4.37 – 4.24 (dp, *J* = 9.5, 6.0 Hz, 2H), 1.67 – 1.55 (m, 8H), 0.96 – 0.87 (td, *J* = 7.4, 1.9 Hz, 12H). ^{13}C NMR (101 MHz, Chloroform-*d*) δ 80.60 (d, *J* = 6.5 Hz), 27.69 (dd, *J* = 27.6, 4.1 Hz), 9.40 (d, *J* = 18.8 Hz). ^{31}P NMR (162 MHz, Chloroform-*d*) δ 6.28. HRMS (ESI) calcd for $C_{10}H_{24}O_3P$ $[M+H]^+$: 223.1458 found: 223.1453.

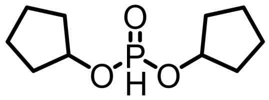

Dicyclopentyl phosphonate (21)(*3*): colorless oil was obtained. ^{1}H NMR (400 MHz, Chloroform-*d*) δ 7.79 – 5.72 (d, *J* = 686.9 Hz, 1H), 4.95 – 4.85 (dp, *J* = 8.3, 4.0 Hz, 2H), 2.03 – 1.68 (dq, *J* = 25.4, 5.8 Hz, 12H), 1.64 – 1.50 (p, *J* = 5.7, 4.3 Hz, 4H). ^{13}C NMR (101 MHz, Chloroform-*d*) δ 79.21 (d, *J* = 6.0 Hz), 33.91 (d, *J* = 4.8 Hz), 22.87 (d, *J* = 1.9 Hz). ^{31}P NMR (162 MHz, Chloroform-*d*) δ 5.30.

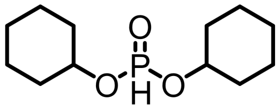

Dicyclohexyl phosphonate (22)(*3*): colorless oil was obtained. ^{1}H NMR (400 MHz, Chloroform-*d*) δ 7.79 – 5.91 (d, *J* = 688.6 Hz, 1H), 4.50 – 4.37 (ddq, *J* = 12.9, 8.8, 3.9 Hz, 2H), 1.99 – 1.87 (m, 4H), 1.81 – 1.68 (dhept, *J* = 12.3, 3.1 Hz, 4H), 1.61 – 1.45 (dtd, *J* = 18.9, 7.9, 6.2, 3.7 Hz, 6H), 1.41 – 1.17 (m, 6H). ^{13}C NMR (101 MHz, Chloroform-*d*) δ 75.68 (d, *J* = 6.0 Hz), 33.50 (d, *J* = 4.5 Hz), 25.03, 23.51. ^{31}P NMR (162 MHz, Chloroform-*d*) δ 4.53.

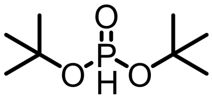

Di-tert-butyl phosphite (23): colorless oil was obtained. ^{1}H NMR (400 MHz, Chloroform-*d*) δ 7.85 – 6.05 (d, *J* = 681.9 Hz, 1H), 1.52 – 1.48 (s, 18H). ^{13}C NMR (101 MHz, Chloroform-*d*) δ 82.84 (d, *J* = 7.3 Hz), 30.31 (d, *J* = 4.6 Hz). ^{31}P NMR (162 MHz, Chloroform-*d*) δ -3.19. HRMS (ESI) calcd for $C_8H_{20}O_3P$ $[M+H]^+$: 195.1145 found: 195.1128.

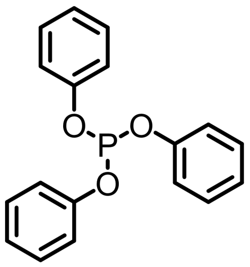

Triphenyl phosphite (24)(*4*): colorless oil was obtained. ^{1}H NMR (400 MHz, Chloroform-*d*) δ 7.42 – 7.34 (m, 6H), 7.26 – 7.15 (m, 9H). ^{13}C NMR (101 MHz, Chloroform-*d*) δ 151.50 (d, *J* = 3.5 Hz), 129.66, 124.21 (d, *J* = 1.3 Hz), 120.66 (d, *J* = 7.0 Hz). ^{31}P NMR (162 MHz, Chloroform-*d*) δ 127.89.

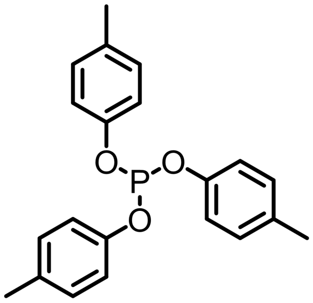

Tri-*p*-tolyl phosphite (25)(*4*): colorless oil was obtained. ^{1}H NMR (400 MHz, Chloroform-*d*) δ 7.21 – 7.16 (m, 6H), 7.15 – 7.10 (m, 6H), 2.40 – 2.36 (s, 9H). ^{13}C NMR (101 MHz, Chloroform-*d*) δ 149.25 (d, *J* = 3.6 Hz), 133.55 (d, *J* = 1.4 Hz), 130.07, 120.43 (d, *J* = 6.7 Hz), 20.65. ^{31}P NMR (162 MHz, Chloroform-*d*) δ 128.47.

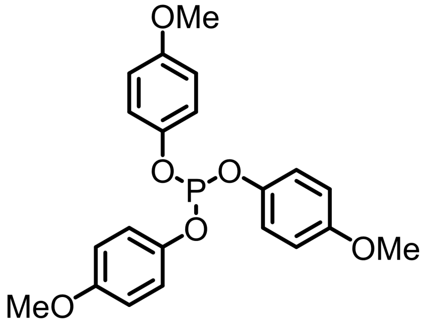

Tris(4-methoxyphenyl) phosphite (26)(*4*): colorless oil was obtained. ^{1}H NMR (400 MHz, Chloroform-*d*) δ 7.11 – 7.02 (m, 6H), 6.89 – 6.80 (m, 6H), 3.80 – 3.76 (s, 9H). ^{13}C NMR (101 MHz, Chloroform-*d*) δ 156.15 (d, *J* = 1.4 Hz), 145.05 (d, *J* = 3.4 Hz), 121.62 (d, *J* = 6.4 Hz), 114.64, 55.57. ^{31}P NMR (162 MHz, Chloroform-*d*) δ 128.91.

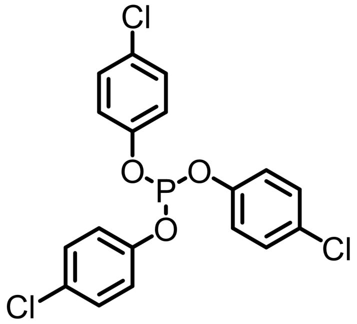

Tris(*p*-methoxyphenyl) phosphite (27)(*5*): colorless oil was obtained. ^{1}H NMR (400 MHz, Chloroform-*d*) δ 7.33 – 7.25 (d, *J* = 8.8 Hz, 6H), 7.08 – 7.02 (d, *J* = 8.4 Hz, 6H). ^{13}C NMR (101 MHz, Chloroform-*d*) δ 149.74 (d, *J* = 2.8 Hz), 129.79, 121.90, 121.83. ^{31}P NMR (162 MHz, Chloroform-*d*) δ 126.89.

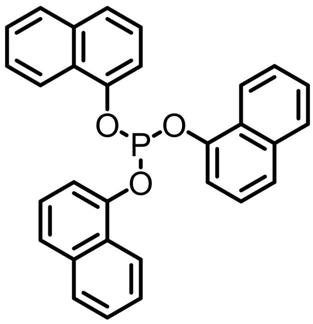

Tri(naphthalen-1-yl) phosphite (28): colorless oil was obtained. ^{1}H NMR (400 MHz, Chloroform-*d*) δ 8.20 – 8.13 (d, *J* = 8.4 Hz, 3H), 7.90 – 7.83 (d, *J* = 8.2 Hz, 3H), 7.71 – 7.64 (d, *J* = 8.0 Hz, 3H), 7.56 – 7.48 (ddd, *J* = 8.2, 6.8, 1.3 Hz, 3H), 7.48 – 7.35 (m, 9H). ^{13}C NMR (101 MHz, Chloroform-*d*) δ 147.72 (d, *J* = 3.0 Hz), 134.87, 127.62, 127.32 (d, *J* = 2.1 Hz), 126.59, 126.06, 125.55, 124.21 (d, *J* = 1.3 Hz), 122.27, 114.99 (d, *J* = 11.9 Hz). ^{31}P NMR (162 MHz, Chloroform-*d*) δ 129.99. HRMS (ESI) calcd for $C_{30}H_{21}O_3P$ $[M+H]^+$: 461.1301 found: 461.1303.

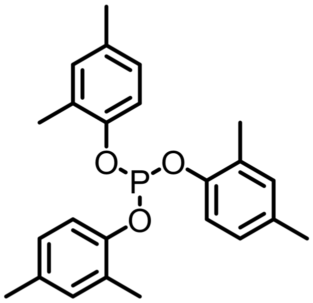

Tris(2.4-dimethylphenyl) phosphite (29): colorless oil was obtained. ^{1}H NMR (400 MHz, Chloroform-*d*) δ 7.11 – 7.04 (dd, *J* = 8.1, 1.1 Hz, 3H), 7.04 – 7.00 (d, *J* = 2.2 Hz, 3H), 6.96 – 6.89 (dd, *J* = 8.2, 2.3 Hz, 3H), 2.32 – 2.28 (s, 9H), 2.23 – 2.19 (s, 9H). ^{13}C NMR (101 MHz, Chloroform-*d*) δ 147.95 (d, *J* = 2.8 Hz), 133.32 (d, *J* = 1.4 Hz), 131.85, 129.48 (d, *J* = 2.6 Hz), 127.12, 120.11 (d, *J* = 10.4 Hz), 20.64, 16.61. ^{31}P NMR (162 MHz, Chloroform-*d*) δ 131.74. HRMS (ESI) calcd for $C_{24}H_{28}O_3P$ $[M+H]^+$: 395.1771 found: 395.1777.

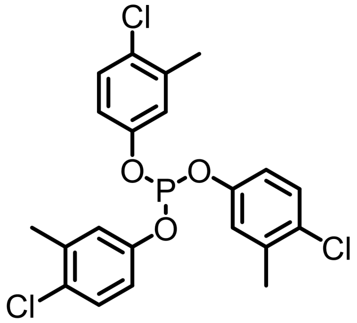

Tris(4-chloro-3-methylphenyl) phosphite (30): colorless oil was obtained. ^{1}H NMR (400 MHz, Chloroform-*d*) δ 7.30 – 7.24 (m, 3H), 7.00 – 6.95 (d, *J* = 2.8 Hz, 3H), 6.93 – 6.85 (dd, *J* = 8.7, 2.9 Hz, 3H), 2.36 – 2.32 (s, 9H). ^{13}C NMR (101 MHz, Chloroform-*d*) δ 149.62 (d, *J* = 2.9 Hz), 137.67, 129.94, 129.86 (d, *J* = 1.7 Hz), 122.89, 119.25, 20.26. ^{31}P NMR (162 MHz, Chloroform-*d*) δ 126.95. HRMS (ESI) calcd for $C_{21}H_{19}Cl_3O_3P$ $[M+H]^+$: 455.0132 found: 455.0140.

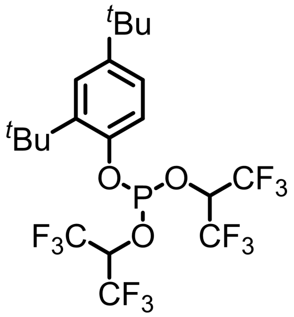

Tris(4-chloro-3-methylphenyl) phosphite (31): colorless oil was obtained. ^{1}H NMR (400 MHz, Chloroform-*d*) δ 7.44 – 7.38 (d, *J* = 2.5 Hz, 1H), 7.20 – 7.13 (dd, *J* = 8.5, 2.5 Hz, 1H), 7.07 – 7.00 (dd, *J* = 8.5, 1.9 Hz, 1H), 5.02 – 4.87 (dp, *J* = 9.1, 5.5 Hz, 2H), 1.33 – 1.29 (s, 12H). ^{13}C NMR (101 MHz, Chloroform-*d*) δ 147.59 (d, *J* = 5.6 Hz), 147.32, 138.85 (d, *J* = 2.8 Hz), 125.01, 123.83, 121.90, 118.51 (d, *J* = 18.0 Hz), 69.65 (td, *J* = 34.7, 9.3 Hz), 37.12, 34.88, 34.57, 31.41, 30.01. ^{31}P NMR (162 MHz, Chloroform-*d*) δ 137.77 (m). HRMS (ESI) calcd for $C_{20}H_{24}F_{12}O_3P$ $[M+H]^+$: 571.1266 found: 571.1277.

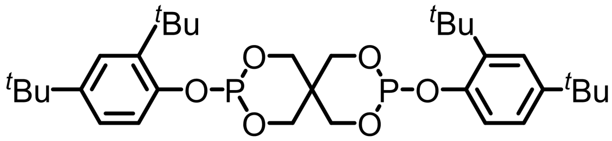

Bis-(2,4-di-tert-butyl-phenyl)-phosphiterythritol diphosphite (32): white solid was obtained. ^{1}H NMR (400 MHz, Chloroform-*d*) δ 7.46 – 7.38 (d, *J* = 2.5 Hz, 1H), 7.22 – 7.14 (dd, *J* = 8.3, 2.5 Hz, 1H), 7.06 – 6.97 (d, *J* = 8.3 Hz, 1H), 4.69 – 4.53 (m, 2H), 4.25 – 4.16 (dd, *J* = 11.4, 2.9 Hz, 1H), 3.56 – 3.43 (m, 1H), 1.47 – 1.43 (s, 9H), 1.35 – 1.30 (s, 9H). ^{13}C NMR (101 MHz, Chloroform-*d*) δ 149.10 (d, *J* = 7.9 Hz), 145.83, 139.14 (d, *J* = 2.5 Hz), 124.43, 123.76, 118.60, 62.72, 62.46, 36.99, 34.92, 34.48, 31.51, 30.18. ^{31}P NMR (162 MHz, Chloroform-*d*) δ 116.53. HRMS (ESI) calcd for $C_{33}H_{51}O_6P_2$ $[M+H]^+$: 605.3155 found: 605.3152.

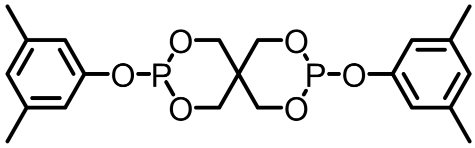

Bis-(3,5-di-methyl-phenyl)-phosphiterythritol diphosphite (33): white solid was obtained. ^{1}H NMR (400 MHz, Chloroform-*d*) δ 6.79 – 6.74 (s, 2H), 6.73 – 6.67 (s, 4H), 4.61 – 4.48 (dd, *J* = 10.9, 3.3 Hz, 4H), 4.25 – 4.17 (dd, *J* = 11.3, 3.0 Hz, 2H), 3.53 – 3.43 (m, 2H), 2.33 – 2.28 (s, 12H). ^{13}C NMR (101 MHz, Chloroform-*d*) δ 152.10 (d, *J* = 7.3 Hz), 139.68, 125.43, 117.37 (d, *J* = 7.9 Hz), 61.98, 37.09 (d, *J* = 4.7 Hz), 21.25. ^{31}P NMR (162 MHz, Chloroform-*d*) δ 117.49. HRMS (ESI) calcd for $C_{21}H_{27}O_6P_2$ $[M+H]^+$: 437.1277 found: 437.1265.

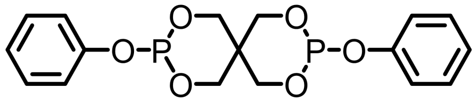

Diphenylpentaerythithol diphosphite (34): colorless oil was obtained. ^{1}H NMR (400 MHz, Chloroform-*d*) δ 7.39 – 7.30 (m, 4H), 7.17 – 7.04 (m, 6H), 4.64 – 4.50 (m, 4H), 4.27 – 4.19 (dd, *J* = 11.3, 2.9 Hz, 2H), 3.56 – 3.45 (ddd, *J* = 11.1, 10.2, 2.3 Hz, 2H). ^{13}C NMR (101 MHz, Chloroform-*d*) δ 152.27 (d, *J* = 7.3 Hz), 129.81, 123.69, 119.74 (d, *J* = 7.8 Hz), 62.00, 37.16. ^{31}P NMR (162 MHz, Chloroform-*d*) δ 117.59. HRMS (ESI) calcd for $C_{17}H_{19}O_6P_2$ $[M+H]^+$: 381.0651 found: 381.0651.

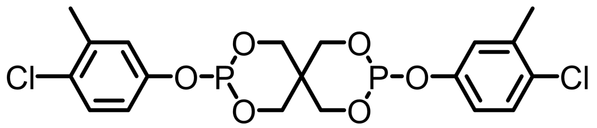

Bis-(4-chloro-3-methylphenyl)-phosphiterythritol diphosphite (35): white solid was obtained. ^{1}H NMR (400 MHz, Chloroform-*d*) δ 7.30 – 7.26 (d, *J* = 8.3 Hz, 2H), 6.98 – 6.93 (d, *J* = 2.9 Hz, 2H), 6.89 – 6.81 (dd, *J* = 8.7, 2.9 Hz, 2H), 4.58 – 4.47 (m, 4H), 4.23 – 4.15 (dd, *J* = 11.3, 2.9 Hz, 2H), 3.54 – 3.44 (m, 2H), 2.38 – 2.33 (s, 6H). ^{13}C NMR (101 MHz, Chloroform-*d*) δ 150.74 (d, *J* = 7.4 Hz), 137.69, 130.03, 129.13, 122.12, 118.37, 62.39, 62.07, 37.13, 20.19. ^{31}P NMR (162 MHz, Chloroform-*d*) δ 117.73. HRMS (ESI) calcd for $C_{19}H_{21}Cl_2O_6P_2$ $[M+H]^+$: 477.0185 found: 477.0182.

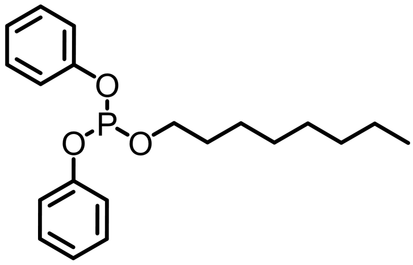

Octyl-diphenyl-phosphite (36): colorless oil was obtained. ^{1}H NMR (400 MHz, Chloroform-*d*) δ 7.38 – 7.27 (m, 4H), 7.19 – 7.07 (m, 6H), 4.20 – 4.10 (q, *J* = 6.6 Hz, 2H), 1.74 – 1.62 (m, 2H), 1.44 – 1.19 (m, 10H), 0.92 – 0.84 (t, *J* = 6.7 Hz, 3H). ^{13}C NMR (101 MHz, Chloroform-*d*) δ 152.22, 129.64, 123.67, 120.23 (d, *J* = 8.2 Hz), 62.80, 31.77, 30.73, 29.20, 29.17, 25.78, 22.64, 14.09. ^{31}P NMR (162 MHz, Chloroform-*d*) δ 128.87. HRMS (ESI) calcd for $C_{20}H_{28}O_3P$ $[M+H]^+$: 347.1771 found: 347.1736.

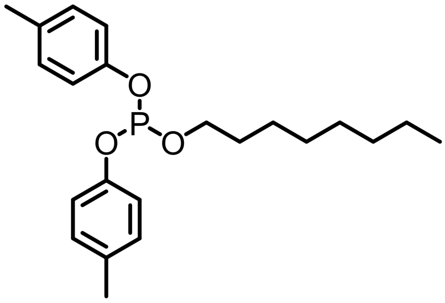

Bis(4-methylphenyl) octyl phosphite (37): white solid was obtained. ^{1}H NMR (400 MHz, Chloroform-*d*) δ 7.15 – 7.07 (d, *J* = 8.4 Hz, 4H), 7.06 – 6.95 (m, 4H), 4.17 – 4.08 (q, *J* = 6.6 Hz, 2H), 2.34 – 2.29 (s, 6H), 1.73 – 1.62 (m, 2H), 1.46 – 1.18 (dd, *J* = 7.6, 3.3 Hz, 10H), 0.93 – 0.85 (t, *J* = 6.7 Hz, 3H). ^{13}C NMR (101 MHz, Chloroform-*d*) δ 149.89 (d, *J* = 6.3 Hz), 133.08 (d, *J* = 1.3 Hz), 130.07, 119.98, 62.59, 31.77, 30.73 (d, *J* = 3.9 Hz), 29.22, 29.18, 25.78, 22.65, 20.68, 14.11. ^{31}P NMR (162 MHz, Chloroform-*d*) δ 129.35. HRMS (ESI) calcd for $C_{22}H_{32}O_3P$ $[M+H]^+$: 375.2084 found: 375.2083.

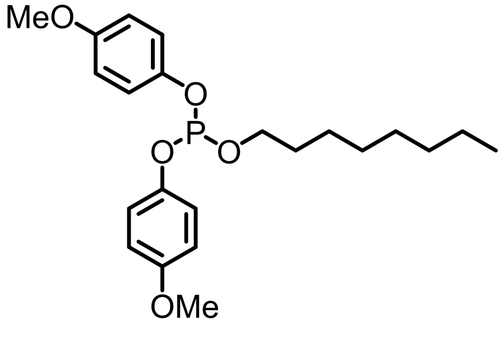

Bis(4-methoxyphenol) octyl phosphite (38): yellow oil was obtained. ^{1}H NMR (400 MHz, Chloroform-*d*) δ 7.07 – 6.96 (m, 4H), 6.88 – 6.79 (m, 4H), 4.17 – 4.07 (q, *J* = 6.7 Hz, 2H), 3.80 – 3.76 (s, 6H), 1.73 – 1.64 (m, 2H), 1.45 – 1.26 (m, 10H), 0.93 – 0.85 (m, 3H). ^{13}C NMR (101 MHz, Chloroform-*d*) δ 155.81 (d, *J* = 1.3 Hz), 145.65 (d, *J* = 6.0 Hz), 121.23 (d, *J* = 7.0 Hz), 114.59, 62.67 (d, *J* = 1.4 Hz), 55.55, 31.76, 30.77 (d, *J* = 4.0 Hz), 29.20, 29.17, 25.78, 22.63, 14.08. ^{31}P NMR (162 MHz, Chloroform-*d*) δ 130.08. HRMS (ESI) calcd for $C_{22}H_{32}O_5P$ [M+H]$^+$: 407.1982 found: 407.2003.

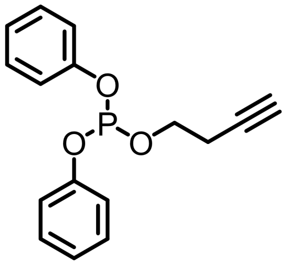

Phosphorous acid diphenyl ester prop-3-ynyl ester (39): white solid was obtained. ^{1}H NMR (400 MHz, Chloroform-*d*) δ 7.37 – 7.27 (dd, *J* = 8.9, 7.0 Hz, 4H), 7.18 – 7.06 (m, 6H), 4.32 – 4.22 (q, *J* = 6.9 Hz, 2H), 2.64 – 2.55 (td, *J* = 7.0, 2.7 Hz, 2H), 2.05 – 1.99 (t, *J* = 2.6 Hz, 1H). ^{13}C NMR (101 MHz, Chloroform-*d*) δ 152.00 (d, *J* = 6.5 Hz), 129.71, 123.88 (d, *J* = 1.3 Hz), 120.22 (d, *J* = 7.7 Hz), 80.32, 70.04, 60.35, 21.09 (d, *J* = 3.8 Hz). ^{31}P NMR (162 MHz, Chloroform-*d*) δ 128.09. HRMS (ESI) calcd for $C_{16}H_{16}O_3P$ [M+H]$^+$: 287.0832 found: 287.0825.

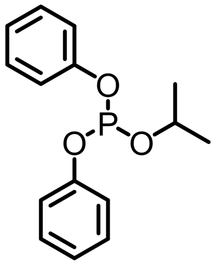

Isopropyl diphenyl phosphite (40): colorless oil was obtained. ^{1}H NMR (400 MHz, Chloroform-*d*) δ 7.39 – 7.27 (m, 4H), 7.15 – 7.06 (m, 6H), 5.04 – 4.88 (dhept, *J* = 9.1, 6.2 Hz, 1H), 1.38 – 1.32 (d, *J* = 6.2 Hz, 6H). ^{13}C NMR (101 MHz, Chloroform-*d*) δ 152.32 (d, *J* = 6.2 Hz), 129.60, 123.58 (d, *J* = 1.4 Hz), 120.17, 67.93 (d, *J* = 2.8 Hz), 24.58 (d, *J* = 2.6 Hz). ^{31}P NMR (162 MHz, Chloroform-*d*) δ 130.19. HRMS (ESI) calcd for $C_{15}H_{18}O_3P$ [M+H]$^+$: 277.0988 found: 277.0981.

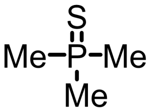

Trimethylphosphane sulfide (41)(*6*): white solid was obtained. ^{1}H NMR (400 MHz, Chloroform-*d*) δ 1.80 – 1.72 (d, *J* = 13.1 Hz, 9H). ^{13}C NMR (101 MHz, Chloroform-*d*) δ 22.54 (d, *J* = 55.7 Hz). ^{31}P NMR (162 MHz, Chloroform-*d*) δ 30.55.

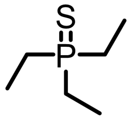

Triethylphosphine sulfide (42)(*7*): white solid was obtained. ^{1}H NMR (400 MHz, Chloroform-*d*) δ 1.87 – 1.74 (dq, *J* = 11.4, 7.6 Hz, 6H), 1.22 – 1.09 (dt, *J* = 18.3, 7.6 Hz, 9H). ^{13}C NMR (101 MHz, Chloroform-*d*) δ 22.81 (d, *J* = 51.7 Hz), 6.37 (d, *J* = 4.7 Hz). ^{31}P NMR (162 MHz, Chloroform-*d*) δ 54.85.

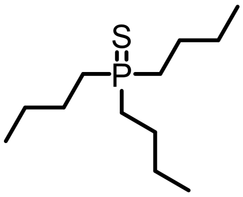

Tri-*n*-butylphosphine sulfide (43)(*8*): colorless oil was obtained. ^{1}H NMR (400 MHz, Chloroform-*d*) δ 1.84 – 1.73 (m, 6H), 1.65 – 1.48 (m, 6H), 1.48 – 1.33 (m, 6H), 0.97 – 0.89 (t, *J* = 7.3 Hz, 9H). ^{13}C NMR (101 MHz, Chloroform-*d*) δ 30.54 (d, *J* = 50.5 Hz), 24.42 (d, *J* = 3.9 Hz), 23.95 (d, *J* = 15.5 Hz), 13.63. ^{31}P NMR (162 MHz, Chloroform-*d*) δ 48.79.

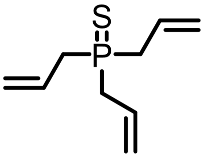

Triallylphosphine sulfide (44)(*7*): colorless oil was obtained. ^{1}H NMR (400 MHz, Chloroform-*d*) δ 5.96 – 5.80 (ddtd, *J* = 17.5, 10.2, 7.5, 5.4 Hz, 3H), 5.36 – 5.14 (m, 6H), 2.77 – 2.67 (ddt, *J* = 14.2, 7.5, 1.2 Hz, 6H). ^{13}C NMR (101 MHz, Chloroform-*d*) δ 127.64 (d, *J* = 8.8 Hz), 121.01 (d, *J* = 12.3 Hz), 36.01 (d, *J* = 48.9 Hz). ^{31}P NMR (162 MHz, Chloroform-*d*) δ 42.52.

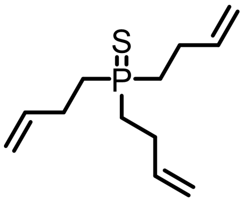

Tri(3-butenyl) phosphine sulfide (45)(*7*): colorless oil was obtained. ^{1}H NMR (400 MHz, Chloroform-*d*) δ 5.90 – 5.75 (ddt, *J* = 16.7, 10.2, 6.4 Hz, 3H), 5.17 – 4.93 (m, 6H), 2.44 – 2.29 (m, 6H), 1.97 – 1.84 (m, 6H). ^{13}C NMR (101 MHz, Chloroform-*d*) δ 136.82 (d, *J* = 15.4 Hz), 115.68, 30.02 (d, *J* = 49.6 Hz), 26.40 (d, *J* = 2.9 Hz). ^{31}P NMR (162 MHz, Chloroform-*d*) δ 48.78.

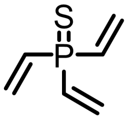

Trivinylphosphine sulfide (46)(*7*): colorless oil was obtained. ^{1}H NMR (400 MHz, Chloroform-*d*) δ 6.46 – 6.27 (m, 6H), 6.28 – 6.09 (m, 3H). ^{13}C NMR (101 MHz, Chloroform-*d*) δ 133.53 (d, *J* = 1.7 Hz), 130.22 (d, *J* = 81.5 Hz). ^{31}P NMR (162 MHz, Chloroform-*d*) δ 29.17.

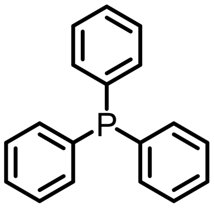

Triphenylphosphine (47)(*9*): white solid was obtained. ^{1}H NMR (400 MHz, Chloroform-*d*) δ 7.45 – 6.78 (m, 15H) ^{13}C NMR (101 MHz, Chloroform-*d*) δ 137.11 (d, *J* = 10.6 Hz), 133.71 (d, *J* = 19.4 Hz), 128.69, 128.47 (d, *J* = 6.9 Hz). ^{31}P NMR (162 MHz, Chloroform-*d*) δ -5.57.

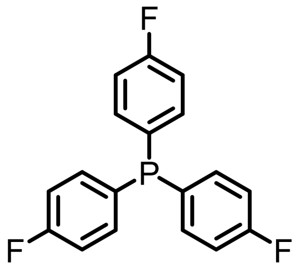

Tri(4-fluorophenyl) phosphine (48)(*10*): white solid was obtained. ^{1}H NMR (400 MHz, Chloroform-*d*) δ 7.35 – 7.23 (m, 6H), 7.14 – 7.04 (t, *J* = 8.6 Hz, 6H). ^{13}C NMR (101 MHz, Chloroform-*d*) δ 163.43 (d, *J* = 249.5 Hz), 135.54 (d, *J* = 8.0 Hz), 132.46 (dd, *J* = 10.9, 3.5 Hz), 116.04. ^{31}P NMR (162 MHz, Chloroform-*d*) δ -9.12. ^{19}F NMR (377 MHz, Chloroform-*d*) δ -111.90.

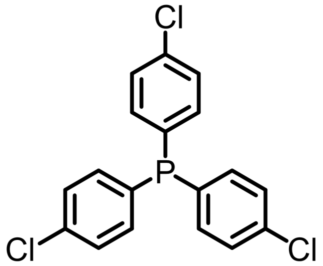

Tri(4-chlorophenyl) phosphine (49)(*10*): white solid was obtained. ^{1}H NMR (400 MHz, Chloroform-*d*) δ 7.37 – 7.29 (m, 6H), 7.26 – 7.15 (m, 6H). ^{13}C NMR (101 MHz, Chloroform-*d*) δ 135.51, 134.88 (d, *J* = 5.2 Hz), 134.72 (d, *J* = 3.3 Hz), 128.93. ^{31}P NMR (162 MHz, Chloroform-*d*) δ -8.63.

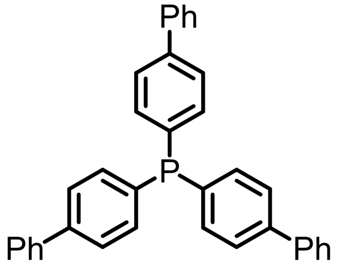

Tri([1,1'-biphenyl]-4-yl) phosphane (50): white solid was obtained. ^{1}H NMR (400 MHz, Chloroform-*d*) δ 7.69 – 7.60 (tt, *J* = 7.1, 2.0 Hz, 12H), 7.54 – 7.43 (dt, *J* = 10.9, 7.7 Hz, 12H), 7.43 – 7.34 (m, 3H). ^{13}C NMR (101 MHz, Chloroform-*d*) δ 141.55, 141.18, 140.45, 135.98 (d, *J* = 10.7 Hz), 134.19 (d, *J* = 19.6 Hz), 128.77 (d, *J* = 9.9 Hz), 127.56, 127.41 – 126.97 (m). ^{31}P NMR (162 MHz, Chloroform-*d*) δ -7.57. HRMS (ESI) calcd for $C_{36}H_{28}P$ $[M+H]^{+}$: 491.1923 found: 491.1909.

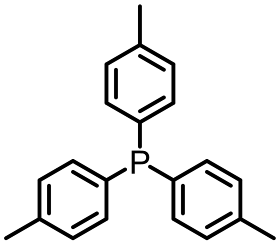

Tri(4-methylphenyl) phosphine (51)(*11*): white solid was obtained. ^{1}H NMR (400 MHz, Chloroform-*d*) δ 7.25 – 7.18 (dd, *J* = 8.7, 6.8 Hz, 6H), 7.18 – 7.12 (m, 6H), 2.38 – 2.33 (s, 9H). ^{13}C NMR (101 MHz, Chloroform-*d*) δ 138.48, 134.04 (d, *J* = 9.1 Hz), 133.59 (d, *J* = 19.4 Hz), 126.76, 21.28. ^{31}P NMR (162 MHz, Chloroform-*d*) δ -8.05.

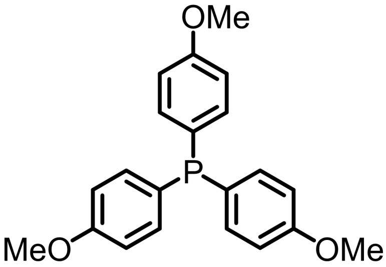

Tri(4-methoxyphenyl) phosphine (52)(*10*): white solid was obtained. ^{1}H NMR (400 MHz, Chloroform-*d*) δ 7.27 – 7.18 (dd, *J* = 8.8, 7.2 Hz, 6H), 6.92 – 6.85 (m, 6H), 3.82 – 3.78 (s, 9H). ^{13}C NMR (101 MHz, Chloroform-*d*) δ 160.00, 134.89 (d, *J* = 20.7 Hz), 128.74 (d, *J* = 7.3 Hz), 114.06 (d, *J* = 7.7 Hz), 55.13. ^{31}P NMR (162 MHz, Chloroform-*d*) δ -10.34.

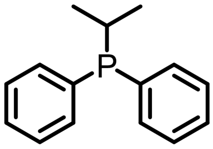

Isopropyldiphenylphosphine (53): white solid was obtained. ^{1}H NMR (400 MHz, Chloroform-*d*) δ 7.54 – 7.44 (ddt, *J* = 7.1, 5.2, 2.3 Hz, 4H), 7.38 – 7.26 (d, *J* = 5.3 Hz, 6H), 2.50 – 2.35 (m, *J* = 6.9 Hz, 1H), 1.13 – 1.02 (dd, *J* = 15.4, 6.9 Hz, 6H). ^{13}C NMR (101 MHz, Chloroform-*d*) δ 137.43, 133.52 (d, *J* = 18.9 Hz), 128.66, 128.27 (d, *J* = 7.0 Hz), 25.00 (d, *J* = 7.7 Hz), 19.64 (d, *J* = 17.8 Hz). ^{31}P NMR (162 MHz, Chloroform-*d*) δ 1.28. HRMS (ESI) calcd for $C_{15}H_{18}P$ $[M+H]^+$: 229.1141 found: 229.1138.

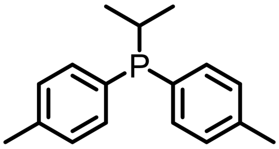

Isopropylbis(4-methylphenyl) phosphine (54): white solid was obtained. ^{1}H NMR (400 MHz, Chloroform-*d*) δ 7.44 – 7.35 (t, *J* = 7.6 Hz, 4H), 7.18 – 7.11 (d, *J* = 7.6 Hz, 4H), 2.48 – 2.36 (m, 1H), 2.36 – 2.30 (s, 6H), 1.11 – 1.01 (dd, *J* = 15.4, 6.9 Hz, 6H). ^{13}C NMR (101 MHz, Chloroform-*d*) δ 138.65, 133.53, 133.34, 129.09 (d, *J* = 7.3 Hz), 25.03 (d, *J* = 6.4 Hz), 21.27, 19.60 (d, *J* = 17.7 Hz). ^{31}P NMR (162 MHz, Chloroform-*d*) δ -0.54. HRMS (ESI) calcd for $C_{17}H_{22}P$ $[M+H]^+$: 257.1403 found: 257.1399.

References

1. J. M. Slattery et al. Filling a Niche in "Ligand Space" with Bulky, Electron-Poor Phosphorus(III) Alkoxides. *Chem. Eur. J.* **25**, 2262-2271 (2019).
2. G. Keglevich et al. Continuous Flow Esterification of a H-Phosphinic Acid, and Transesterification of H-Phosphinates and H-Phosphonates under Microwave Conditions. *Molecules* **25**, 719-733 (2020).
3. Y. F. Zhao et al. Direct Synthesis of Dialkylphosphites from White Phosphorus. *Adv. Synth. Catal.* **364**, 2916-2921 (2022).
4. G. Tang et al. Diphenyl Diselenide-Catalyzed Synthesis of Triaryl Phosphites and Triaryl Phosphates from White Phosphorus. *Org. Lett.* **23**, 5158-5163 (2021).
5. C.-Y. Ho et al. Nitrile assisted, Brønsted acid catalyzed regio and stereoselective diarylphosphonylation of allyl silyl ethers. Org. Biomol. Chem. 8, 3480-3487 (2010).
6. M. J. Rose et al. Iron Hydride Detection and Intramolecular Hydride Transfer in a Synthetic Model of Mono-Iron Hydrogenase with a CNS Chelate. *Inorg. Chem.* **55**, 386-389 (2016).
7. G. Baccolini et al. Highly Atom-Economic One-Pot Formation of Three Different C−P Bonds: General Synthesis of Acyclic Tertiary Phosphine Sulfides. *J. Org. Chem.* **70**, 4774-4777 (2005).
8. J. L. Gustafson et al. A Practical Lewis Base Catalyzed Electrophilic Chlorination of Arenes and Heterocycles. *Org. Lett.* **17**, 1042-1045 (2015).
9. G. Keglevich et al. The Deoxygenation of Phosphine Oxides under Green Chemical Conditions. *Heteroat. Chem.* **26**, 199-205 (2015).
10. L. Li et al. Tertiary Amine-Mediated Reductions of Phosphine Oxides to Phosphines. *Org. Lett.* **25**, 5236-5241 (2023).
11. Z. Q. Wang et al. Palladium-Catalyzed C-P(III) Bond Formation by Coupling ArBr/ArOTf with Acylphosphines. *J. Org. Chem.* **86**, 8987-8996 (2021).
12. S. Westenhoff et al. Solvent-Dependent Structural Dynamics in the Ultrafast Photodissociation Reaction of Triiodide Observed with Time-Resolved X-ray Solution Scattering. *J. Am. Chem. Soc.* **145**, 15754-15765 (2023).

13 M. J. Frisch, G. W. Trucks, H. B. Schlegel, G. E. Scuseria, M. A. Robb, J. R. Cheeseman, G. Scalmani, V. Barone, B. Mennucci, G. A. Petersson, H. Nakatsuji, M. Caricato; X. Li; H. P. Hratchian, A. F. Izmaylov, J. Bloino, G. Zheng, J. L. Sonnenberg, M. Hada, M. Ehara, K. Toyota, R. Fukuda, J. Hasegawa, M. Ishida, T. Nakajima, Y. Honda, O. Kitao, H. Nakai, T. Vreven, J. A., Jr. Montgomery, J. E. Peralta, F. Ogliaro, M. Bearpark, J. J. Heyd, E. Brothers, K. N. Kudin, V. N. Staroverov, R. Kobayashi, J. Normand, K. Raghavachari, A. Rendell, J. C. Burant, S. S. Iyengar, J. Tomasi, M. Cossi, N. Rega, N. J. Millam, M. Klene, J. E. J. B. Knox, Cross, V. Bakken, C. Adamo, J. Jaramillo, R. Gomperts, R. E. Stratmann, O. Yazyev, A. J. Austin, R. Cammi, C. Pomelli, J. W. Ochterski, R. L. Martin, K. Morokuma, V. G. Zakrzewski, G. A. Voth, P. Salvador, J. J. Dannenberg, S. Dapprich, A. D. Daniels, O. Farkas, J. B. Foresman, J. V. Ortiz, J. Cioslowski, D. J. Fox, Gaussian 16, Revision C.01; Gaussian, Inc.: Wallingford, CT, (2016).

14 a) R. Valero et al. Performance of the M06 family of exchange-correlation functionals for predicting magnetic coupling in organic and inorganic molecules. *J. Chem. Phys.* **128**,

114103 (2008). b) Y. Zhao et al. The M06 suite of density functionals for main group thermochemistry, thermochemical kinetics, noncovalent interactions, excited states, and transition elements: two new functionals and systematic testing of four M06-class functionals and 12 other functionals. *Chem. Acc.* **120**, 215-241 (2008). c) Y. Zhao et al. Density Functionals with Broad Applicability in Chemistry. *Acc. Chem. Res.* **41**, 157-167 (2008).

15 C. J. Cramer et al. Universal Solvation Model Based on Solute Electron Density and on a Continuum Model of the Solvent Defined by the Bulk Dielectric Constant and Atomic Surface Tensions. *J. Phys. Chem. B.* **113**, 6378-6396 (2009).

16 T. Lu et al. Multiwfn: A multifunctional wavefunction analyzer. *Journal of Computational Chemistry* **33**, 580–592 (2012),

17 S. Manzetti et al. Wavefunction and reactivity study of benzo[a]pyrene diol epoxide and its enantiomeric forms. *Struct Chem* **25**, 1521-1533 (2014),

18 K. Schulten et al. VMD: Visual molecular dynamics. *J. Mol. Graphics,* **14**, 33-38 (1996),

19 Legault, C. Y. CYLView, 1.0b; Université de Sherbrooke: Canada, http://www.cylview.org (2009).

Copies of product NMR spectra

3-1

^{1}H NMR

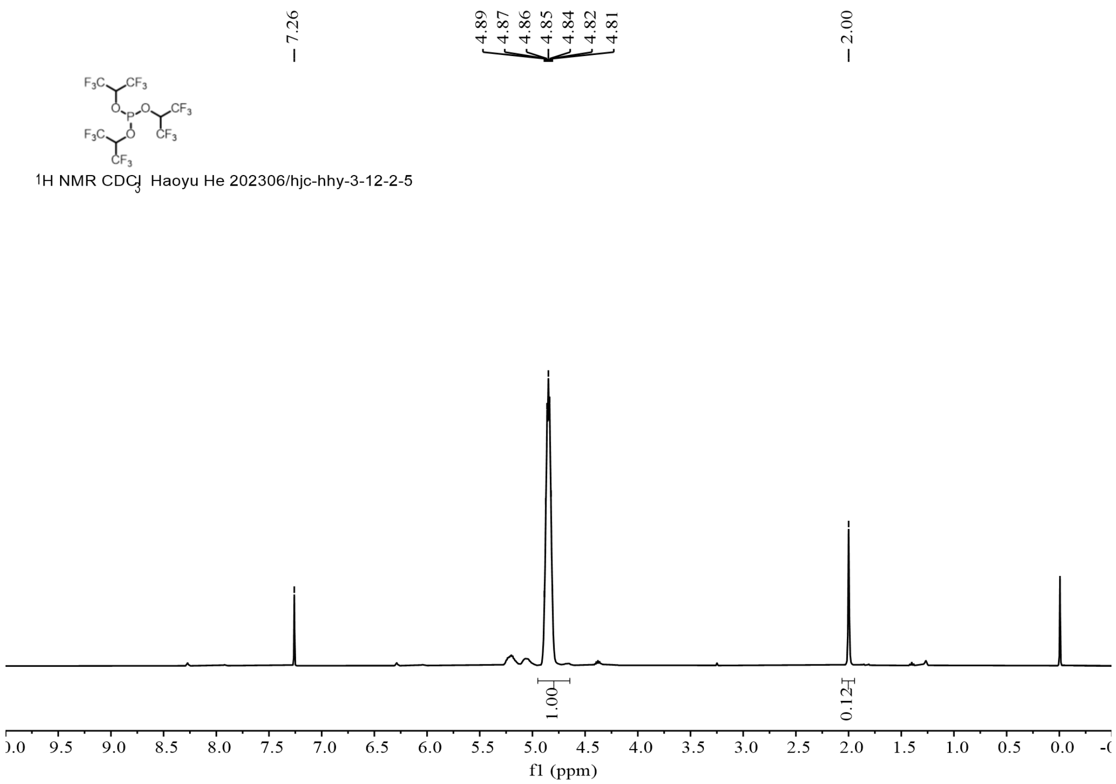

^{13}C NMR

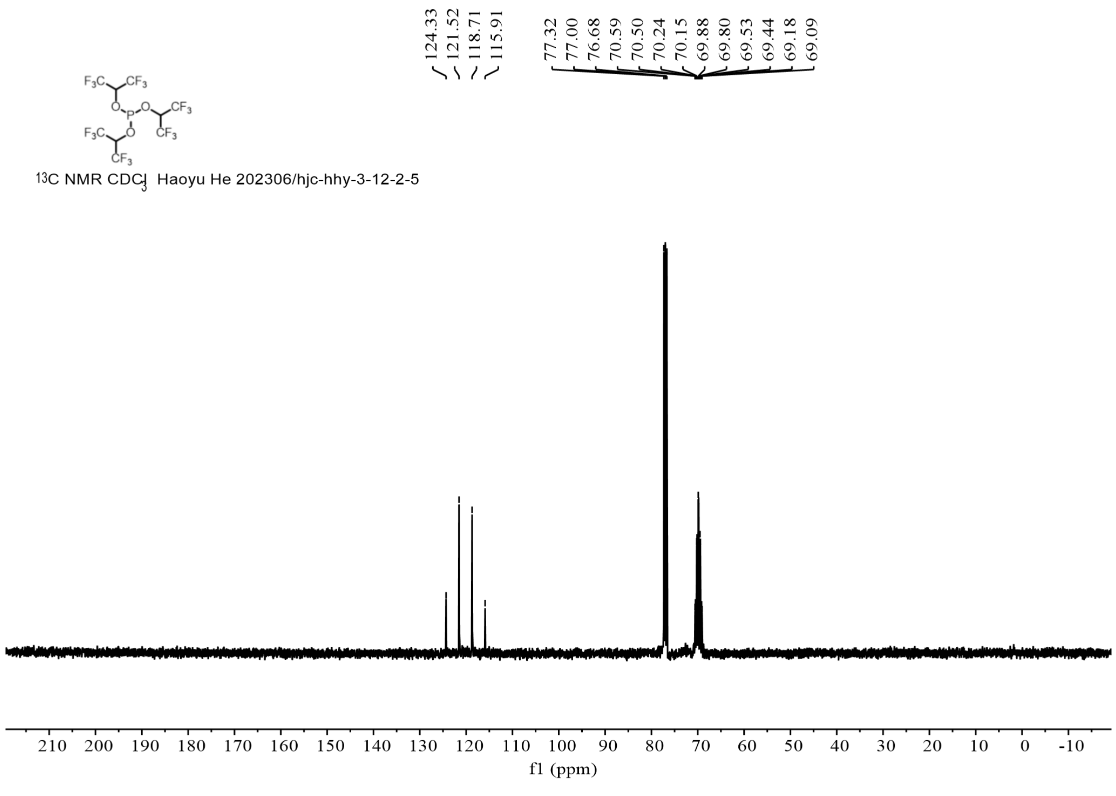

^{31}P NMR

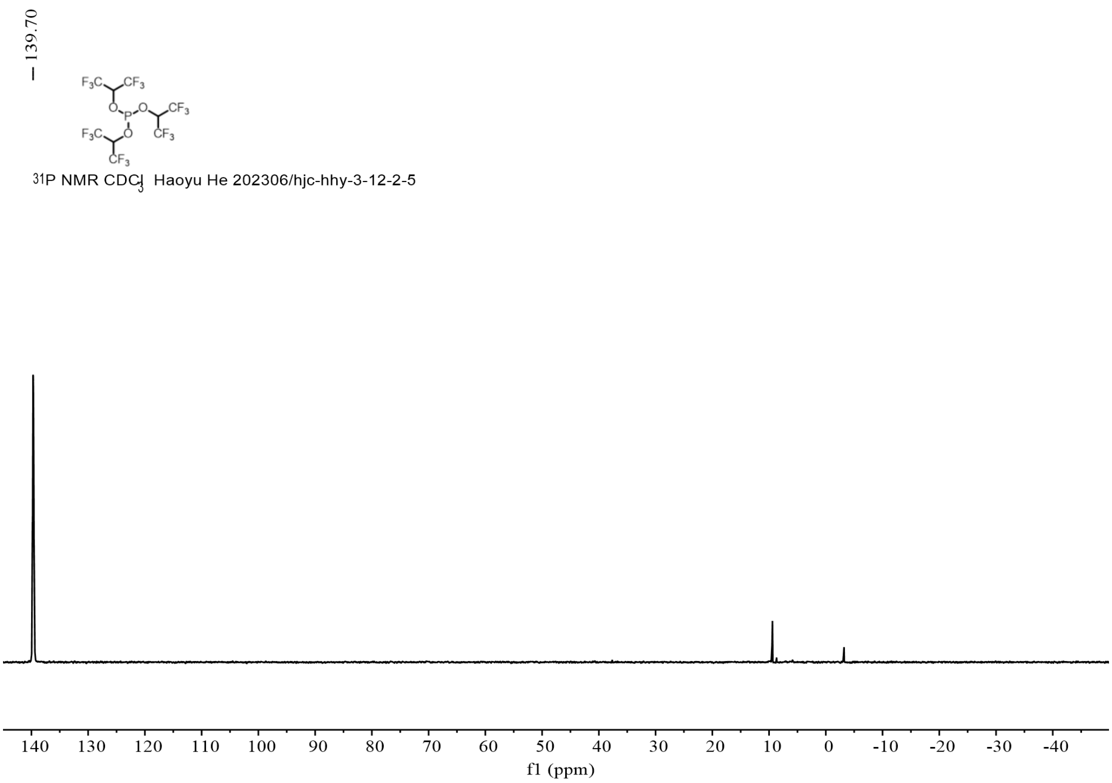

^{19}F NMR

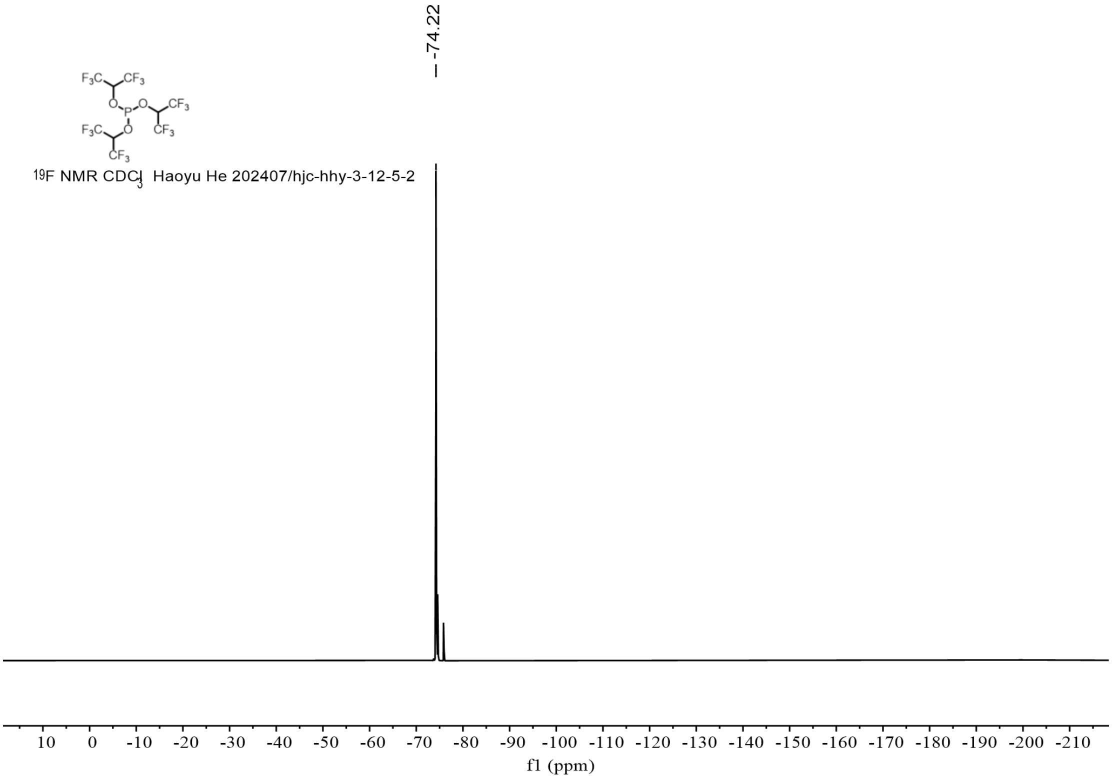

4

^{1}H NMR

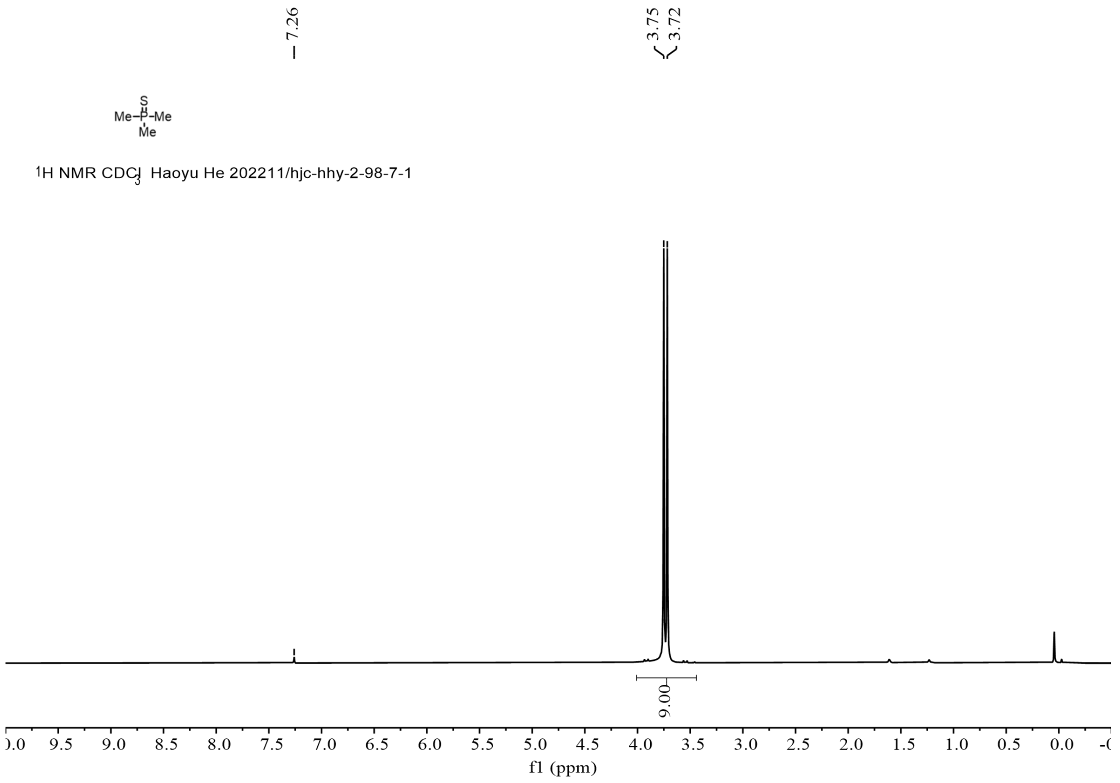

^{13}C NMR

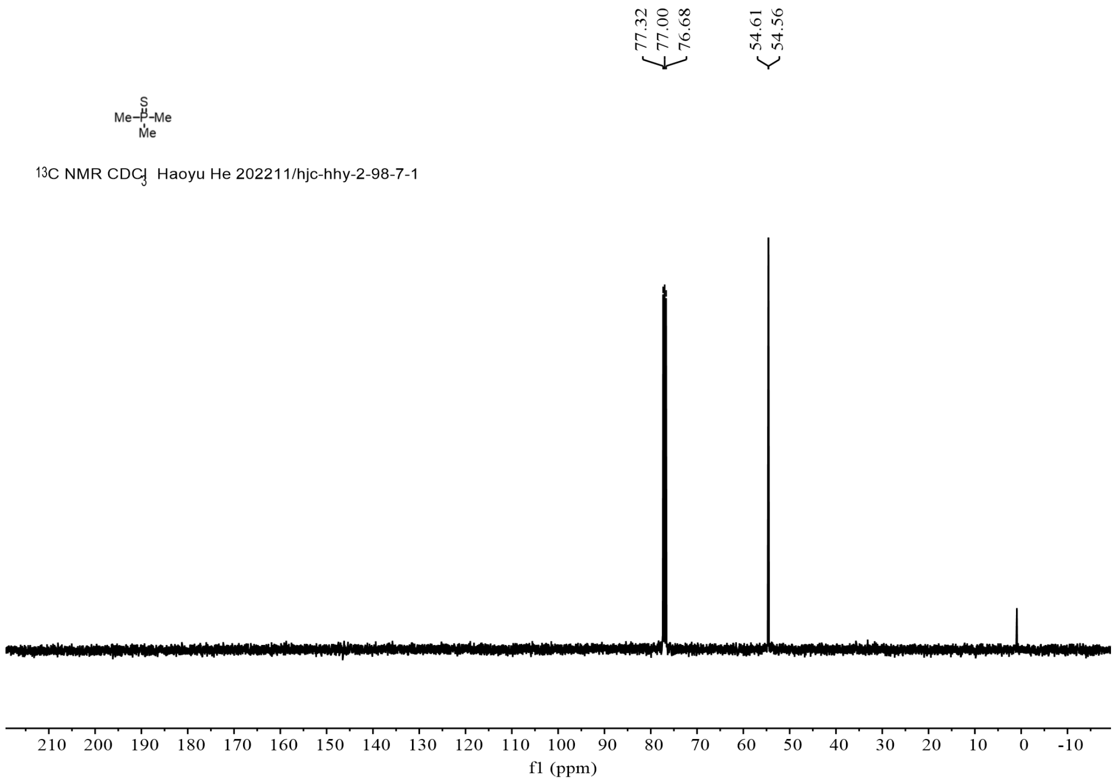

^{31}P NMR

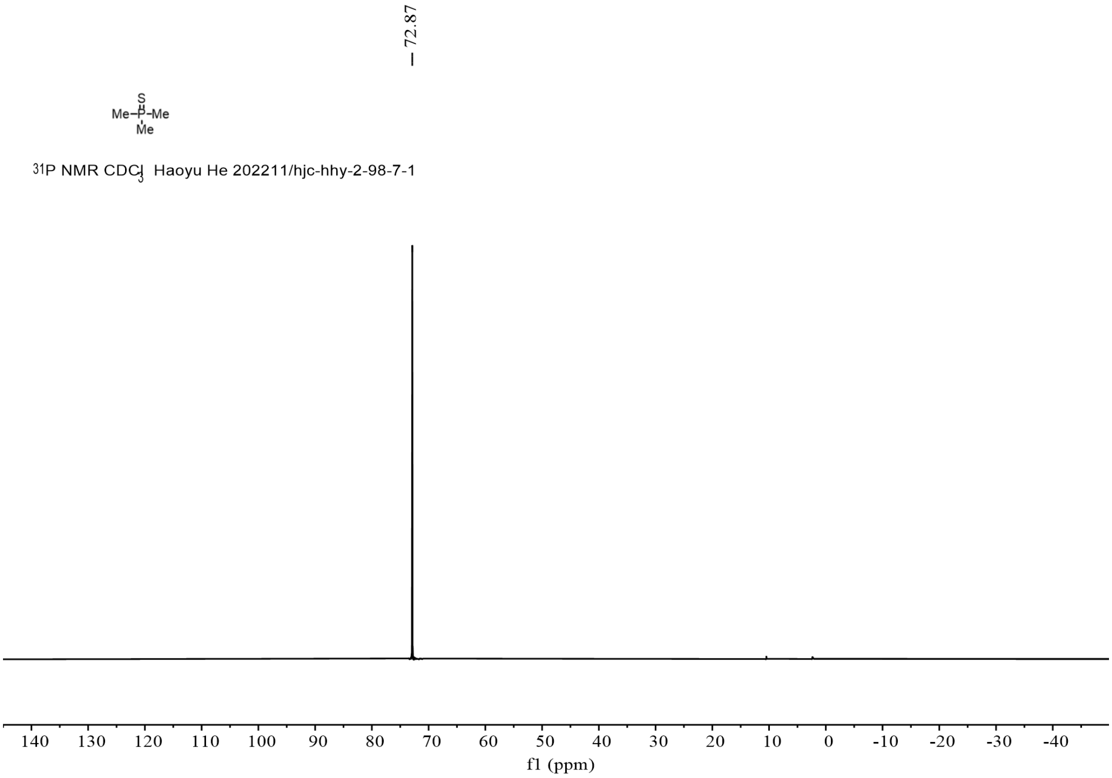

72.87
S
Me–P–Me
Me
31P NMR CDCl3 Haoyu He 202211/hjc-hhy-2-98-7-1
140
130
120
110
100
90
80
70
60
50
40
30
20
10
0
-10
-20
-30
-40
f1 (ppm)

5

^{1}H NMR

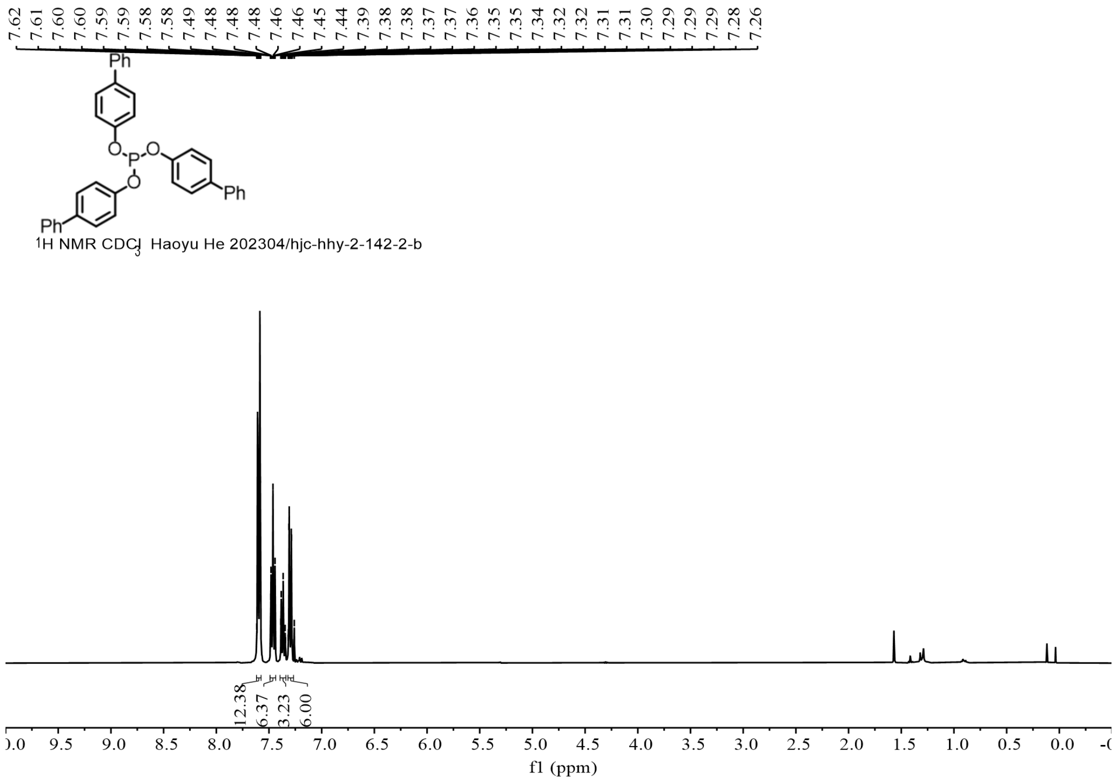

^{13}C NMR

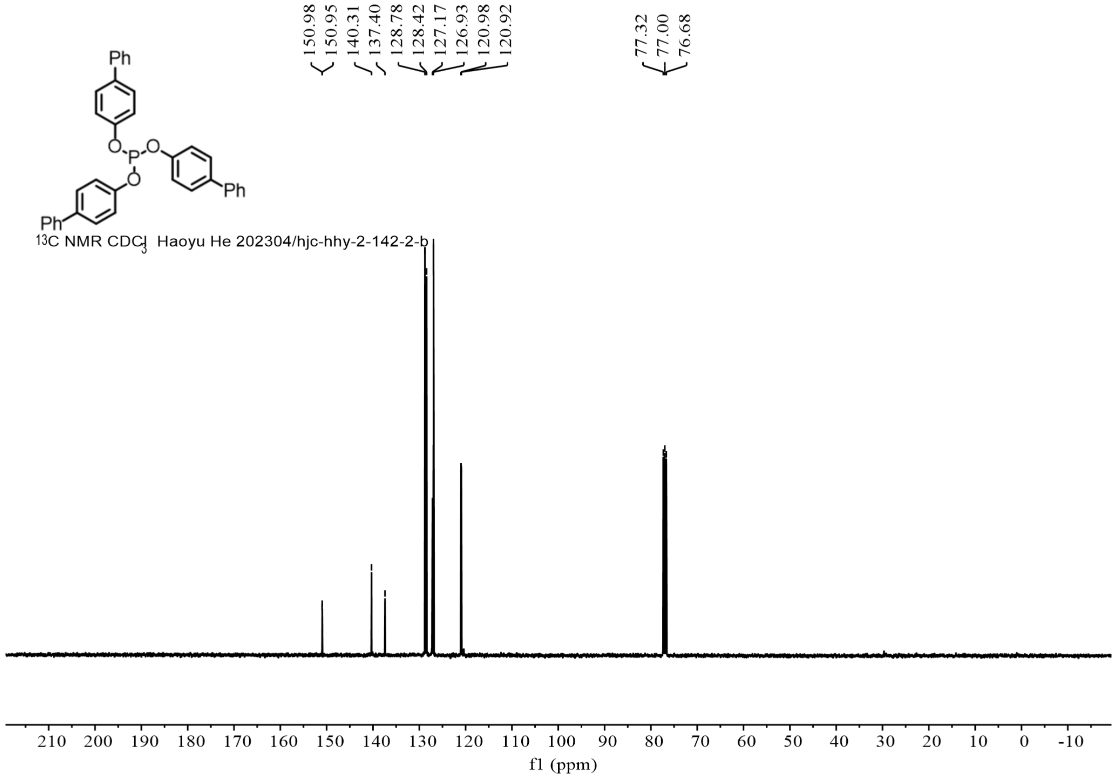

^{31}P NMR

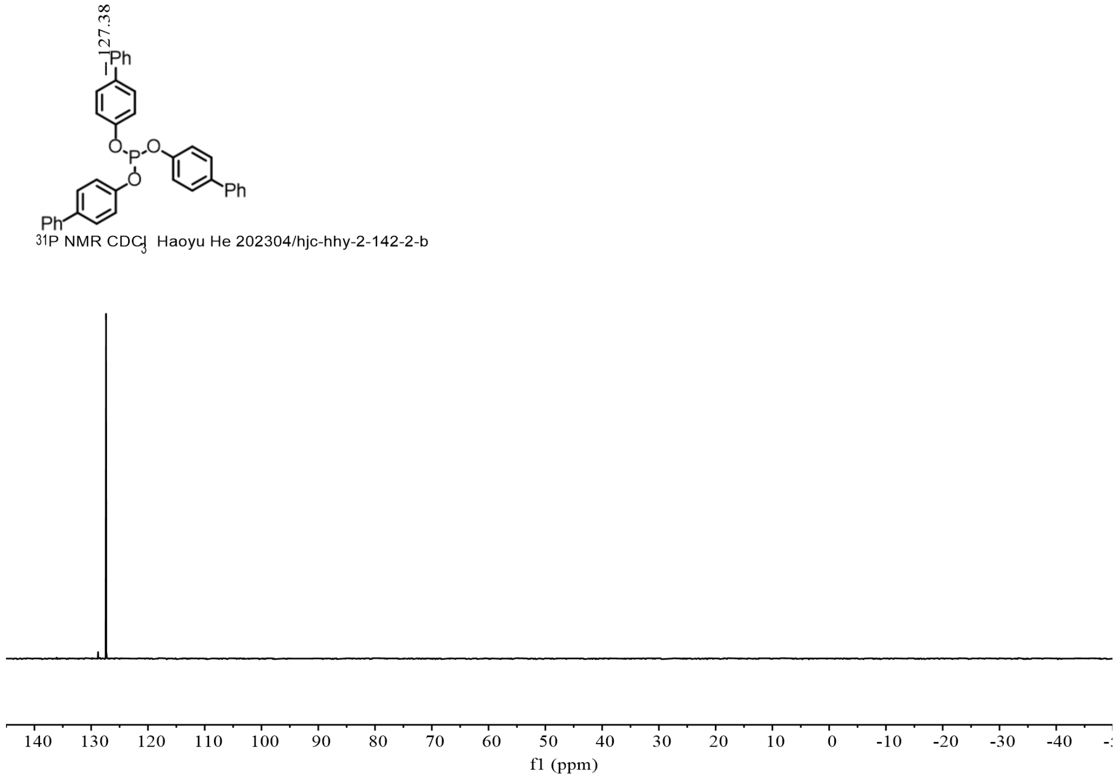

127.38
Ph
O
P
O
O
Ph
Ph
31P NMR CDCl3 Haoyu He 202304/hjc-hhy-2-142-2-b
140
130
120
110
100
90
80
70
60
50
40
30
20
10
0
-10
-20
-30
-40
f1 (ppm)

6

^{1}H NMR

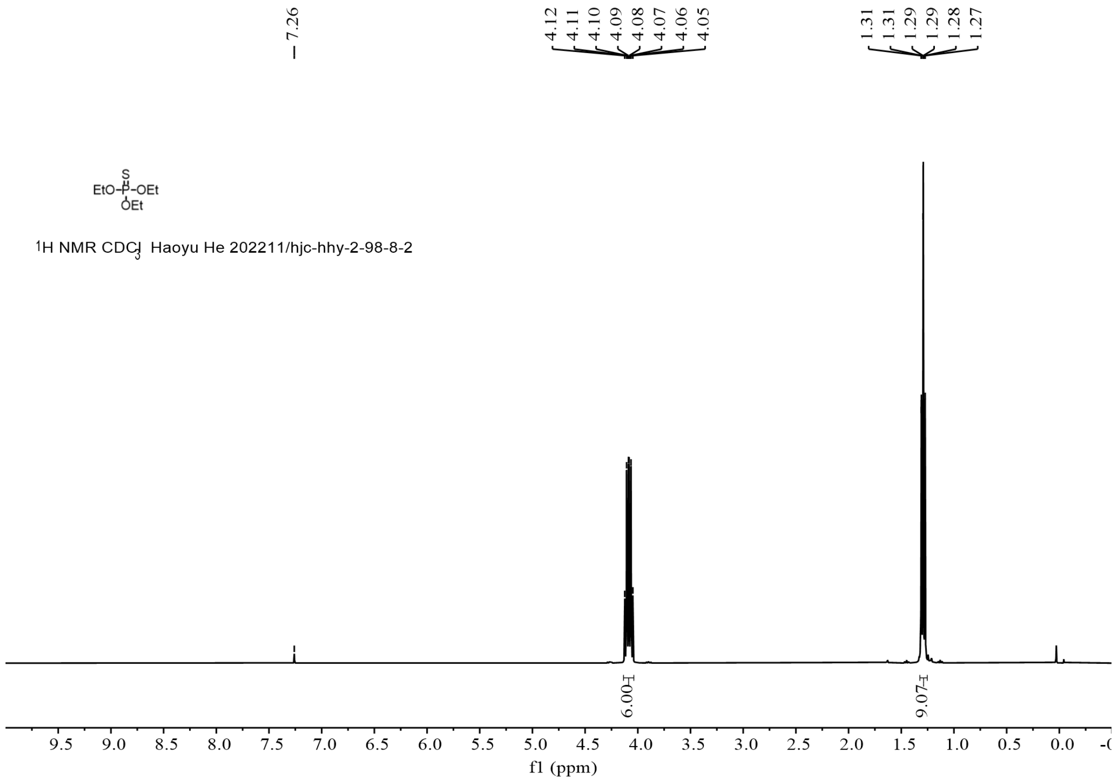

^{13}C NMR

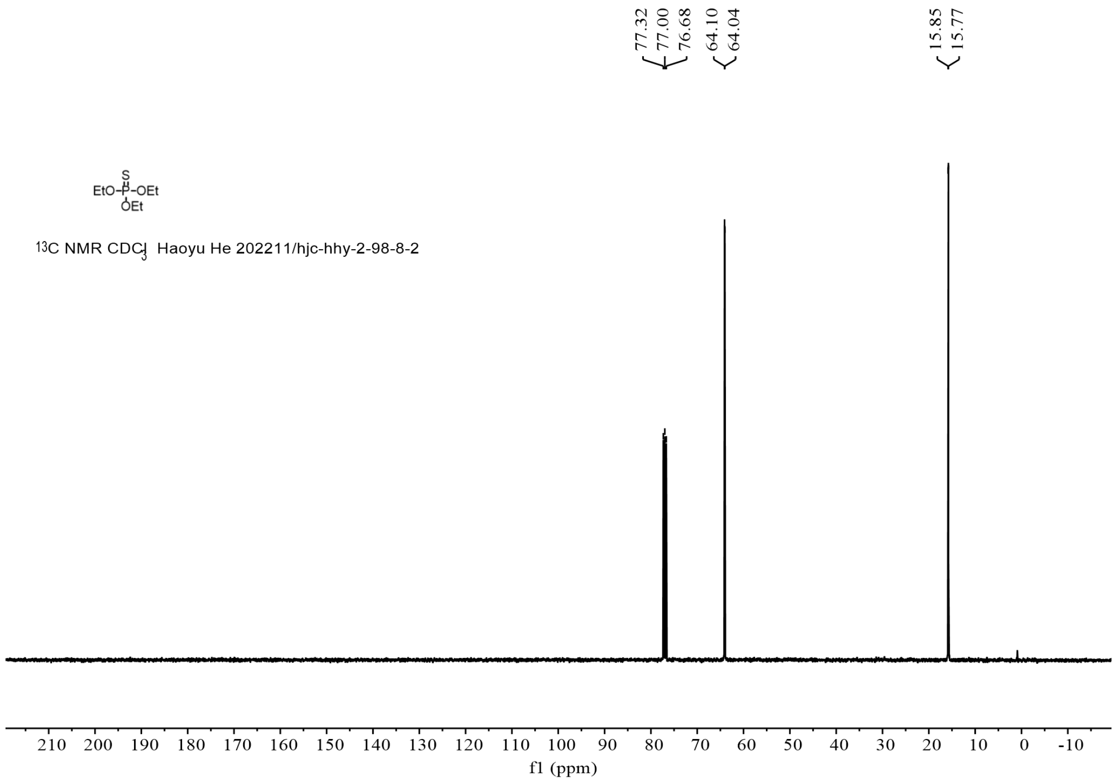

^{31}P NMR

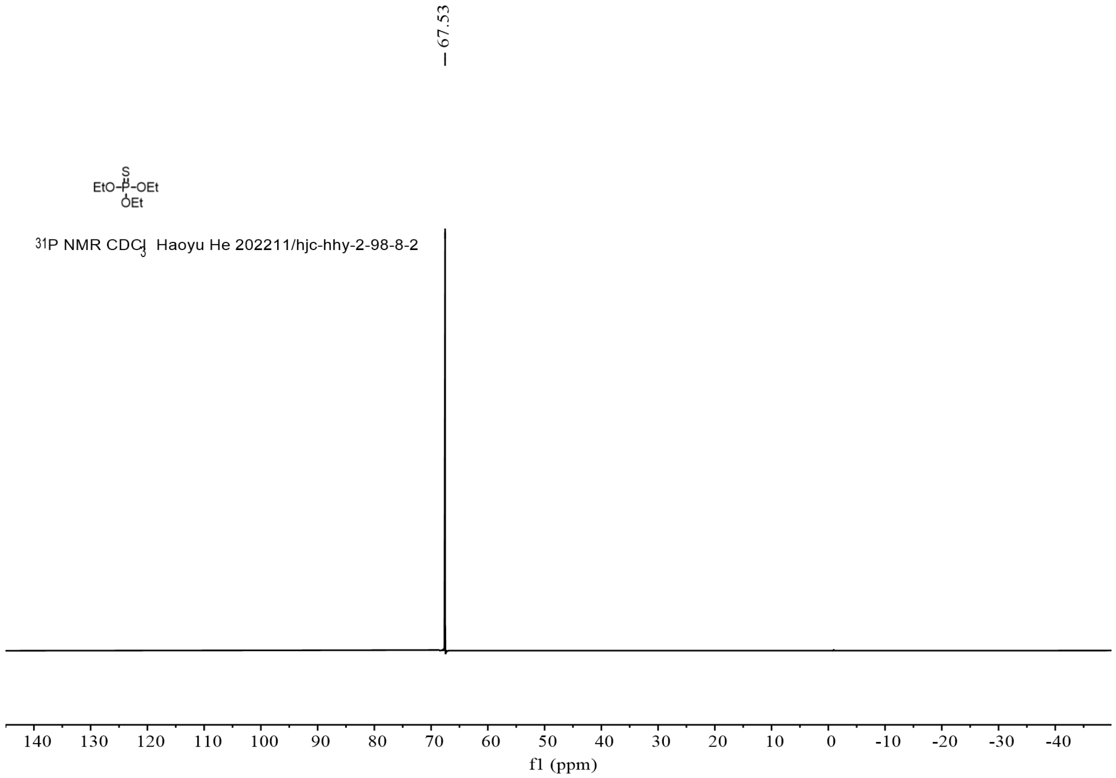

67.53
EtO-P-OEt
S
OEt
31P NMR CDCl3 Haoyu He 202211/hjc-hhy-2-98-8-2
140 130 120 110 100 90 80 70 60 50 40 30 20 10 0 -10 -20 -30 -40
f1 (ppm)

7

^{1}H NMR

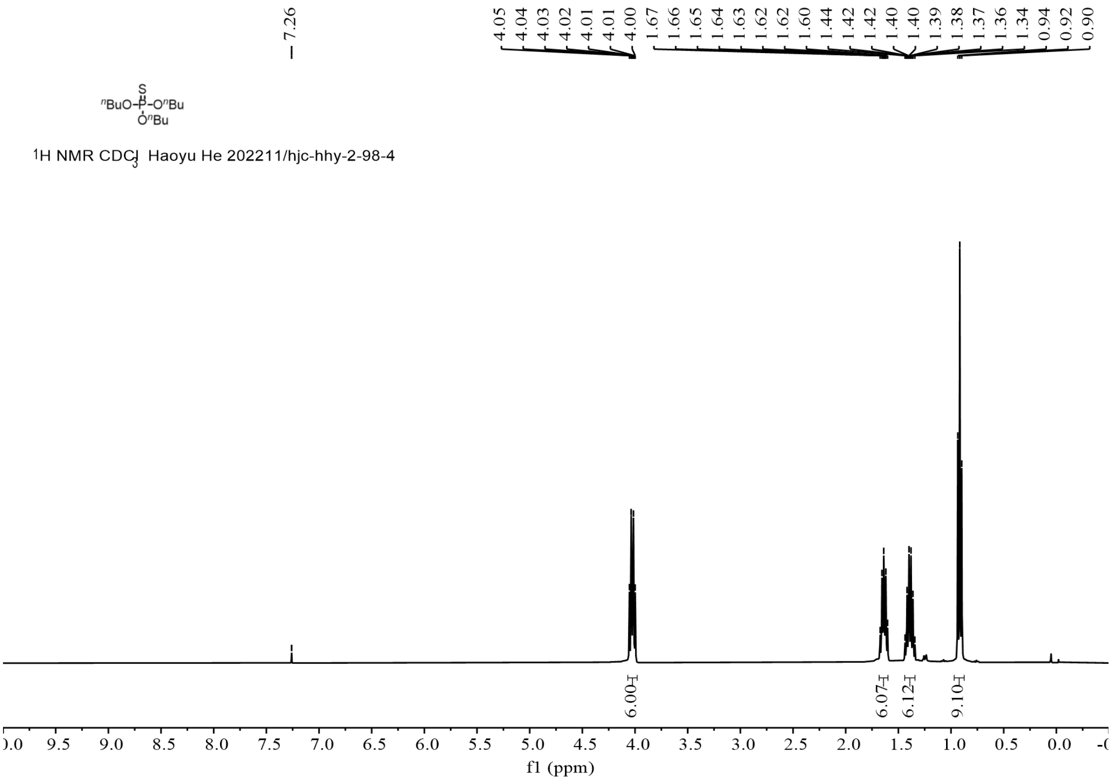

^{13}C NMR

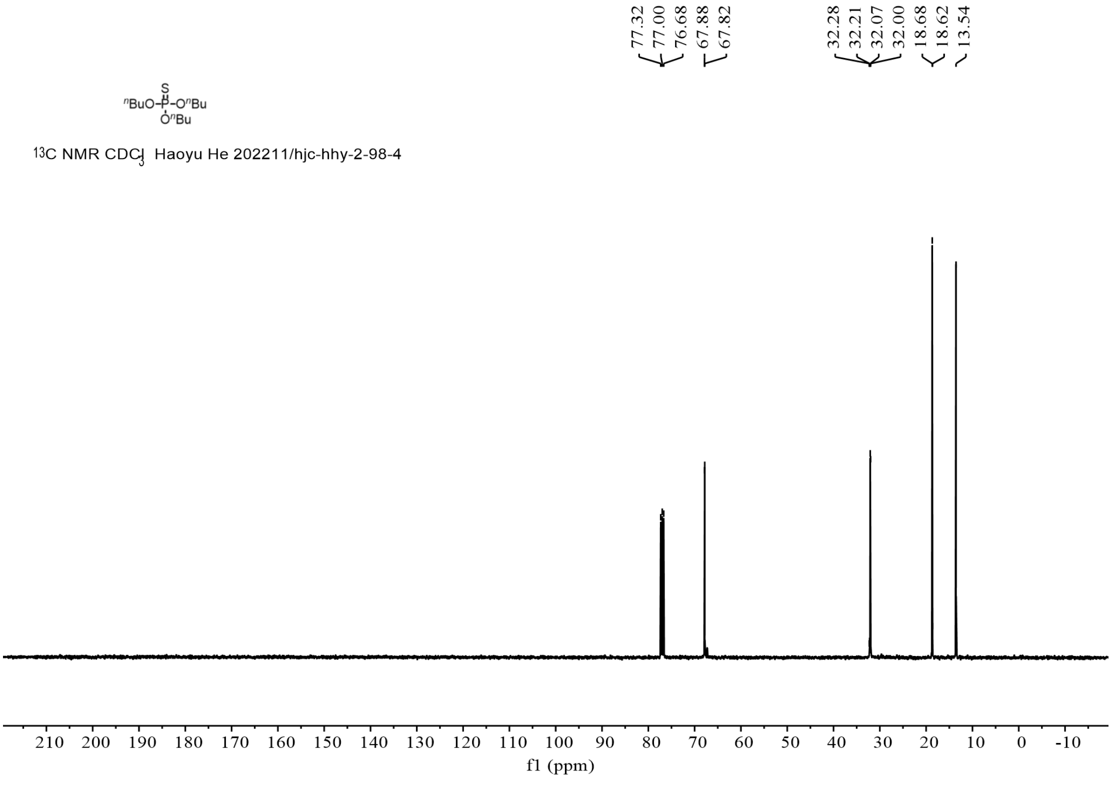

^{31}P NMR

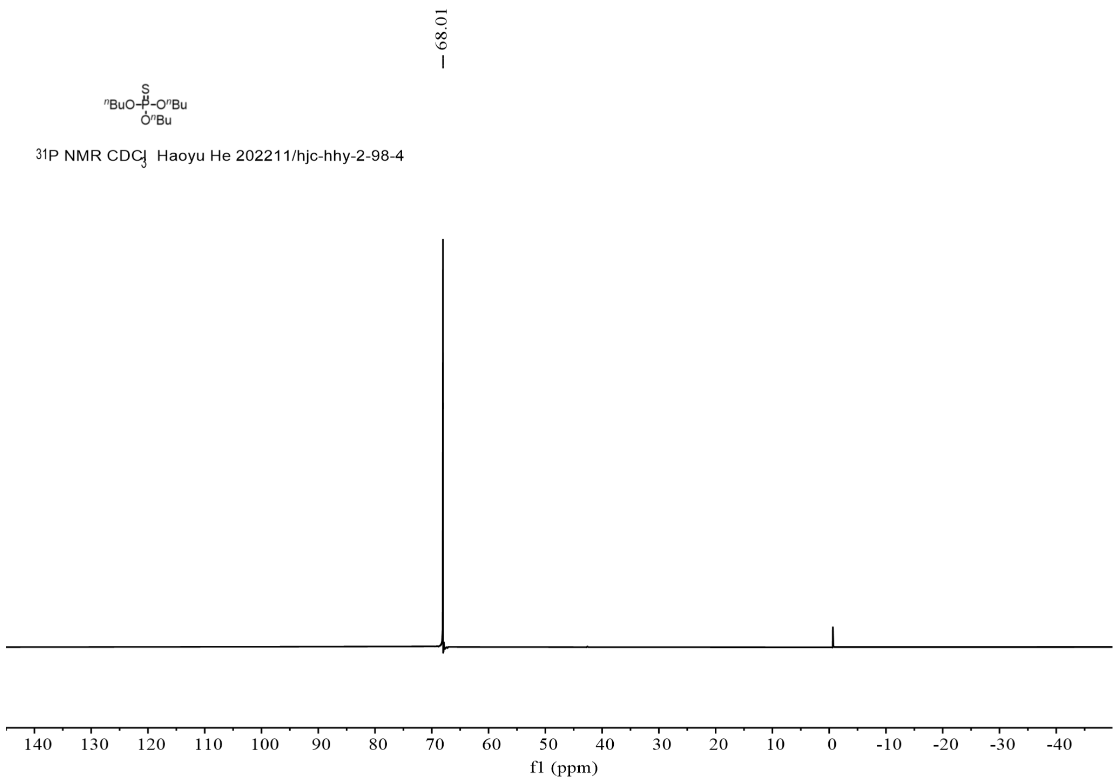
68.01
S
nBuO-P-OnBu
OnBu
31P NMR CDCl3 Haoyu He 202211/hjc-hhy-2-98-4
140 130 120 110 100 90 80 70 60 50 40 30 20 10 0 -10 -20 -30 -40
f1 (ppm)

8

^{1}H NMR

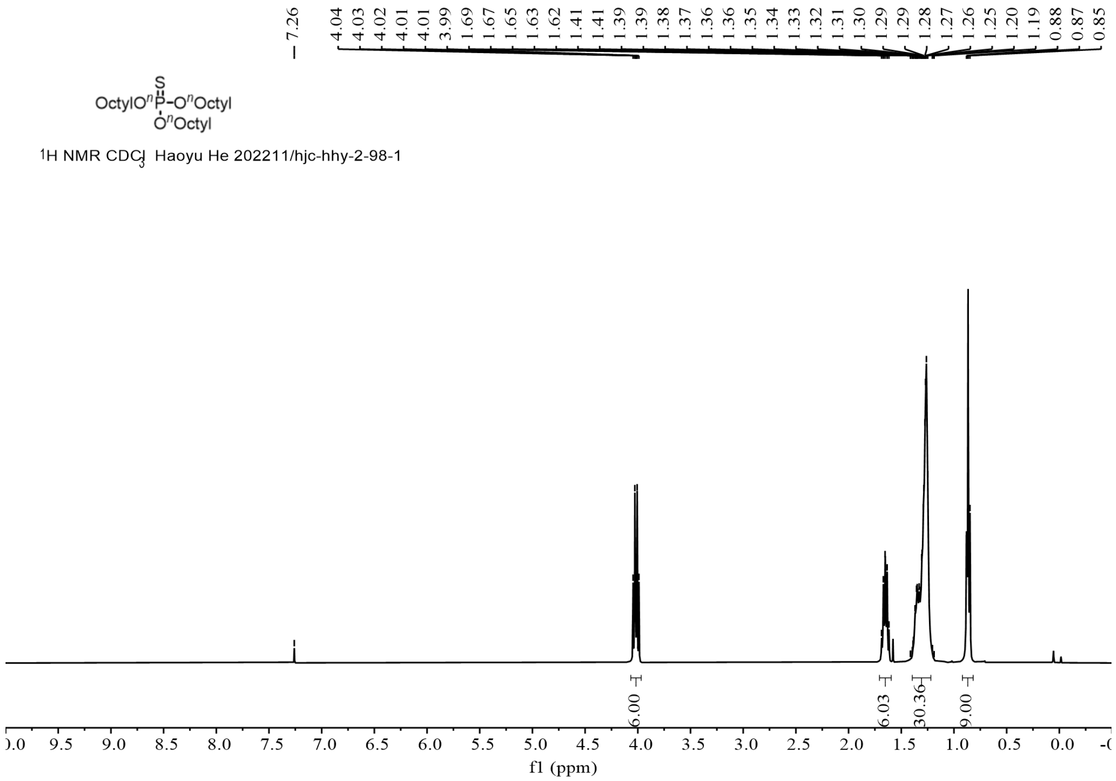

^{13}C NMR

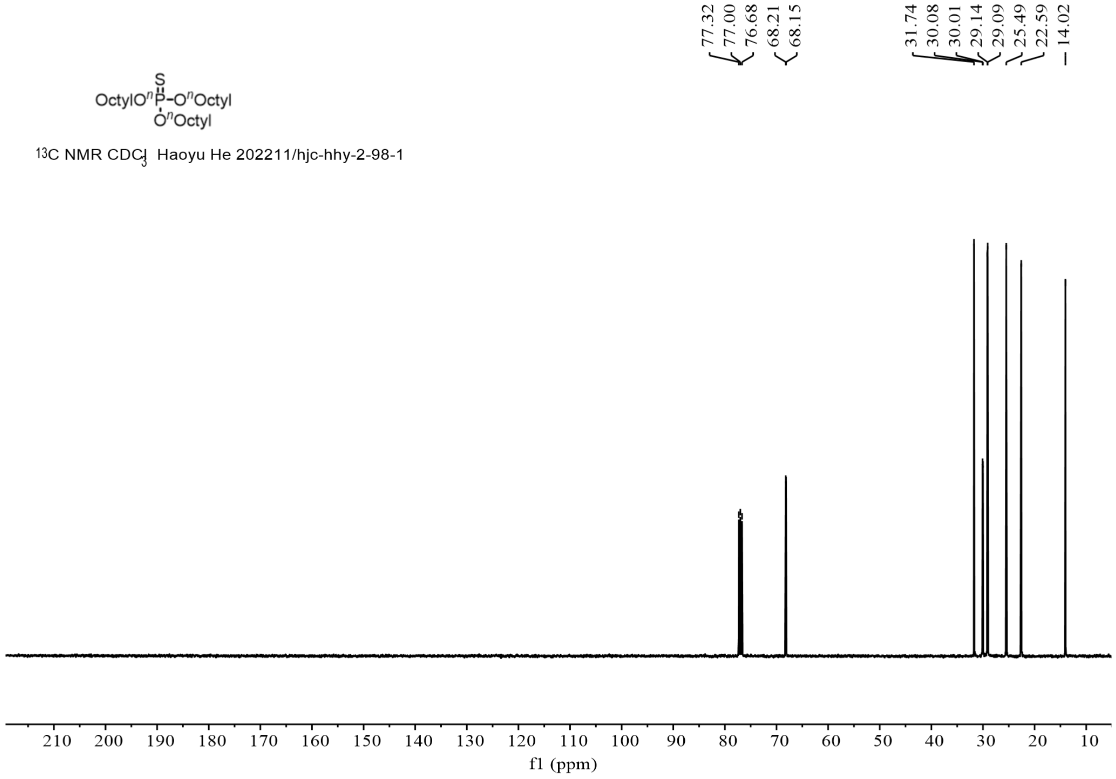

^{31}P NMR

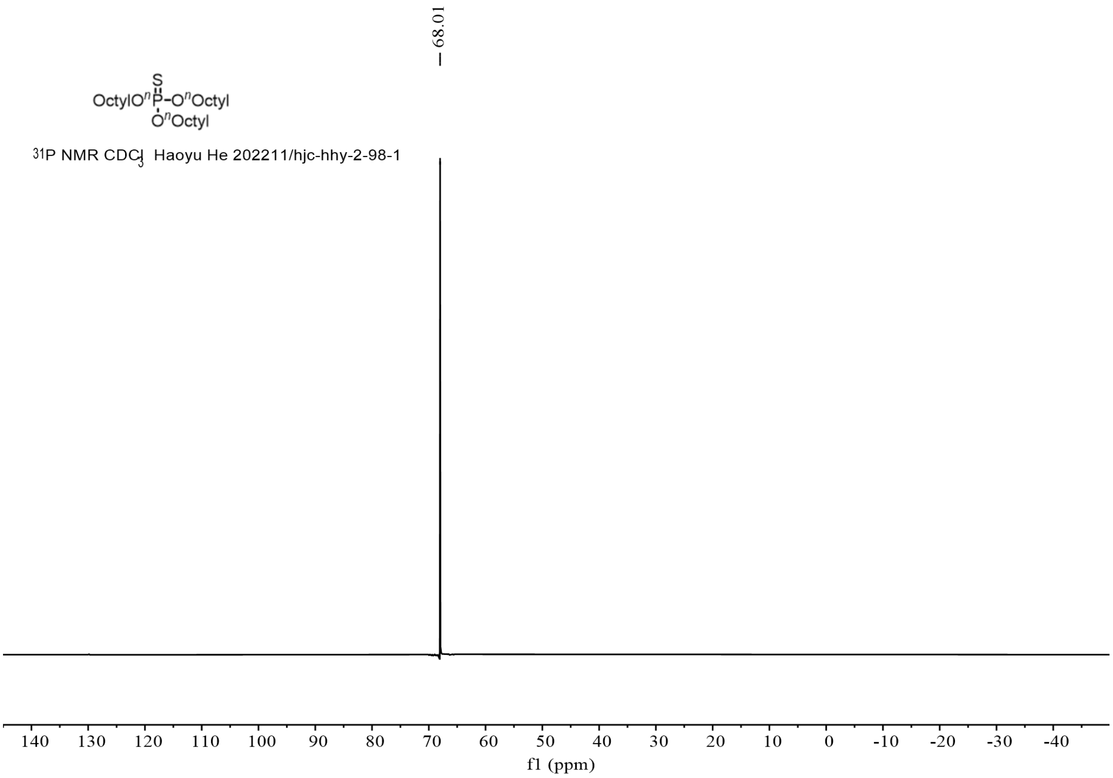
68.01
S
OctylO^nP-O^nOctyl
O^nOctyl
31P NMR CDCl3 Haoyu He 202211/hjc-hhy-2-98-1
140 130 120 110 100 90 80 70 60 50 40 30 20 10 0 -10 -20 -30 -40
f1 (ppm)

9

^{1}H NMR

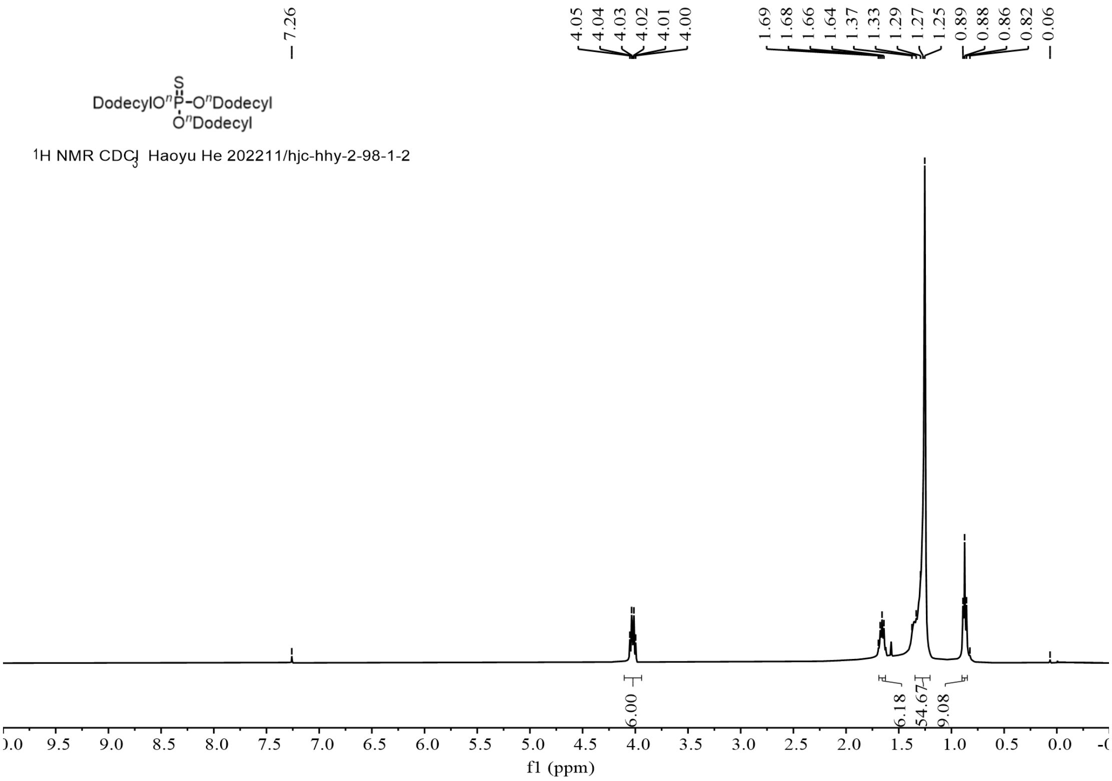

^{13}C NMR

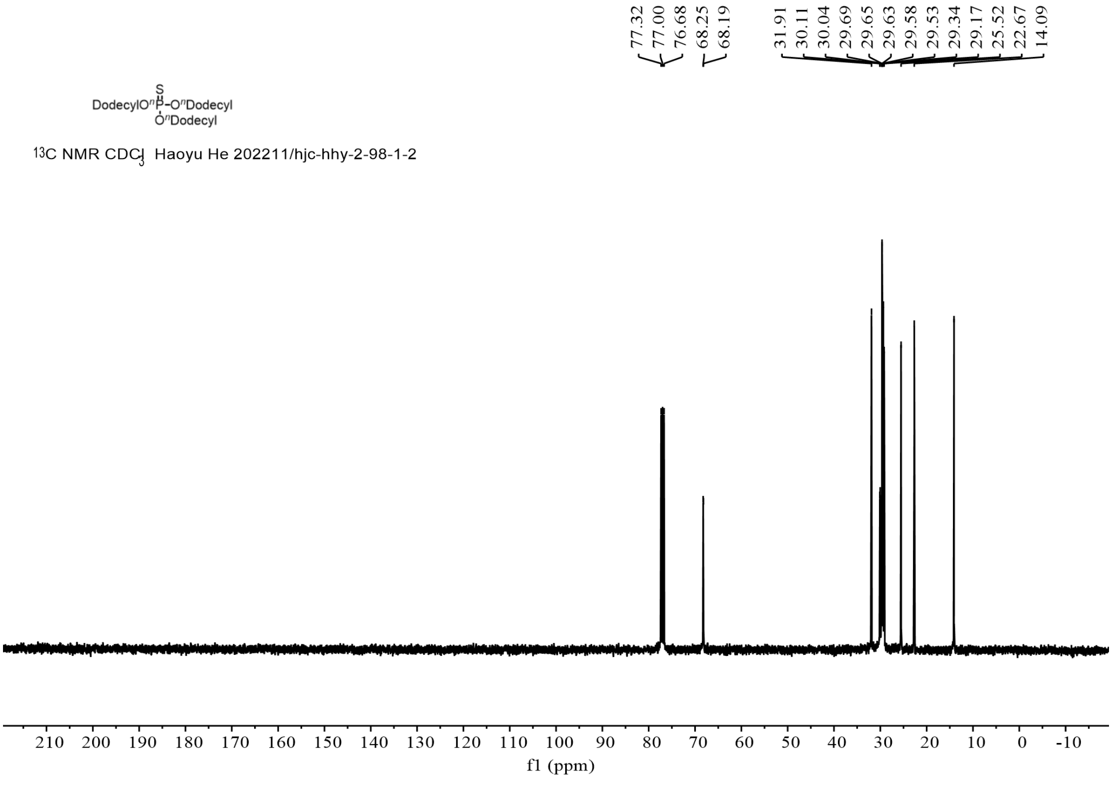

^{31}P NMR

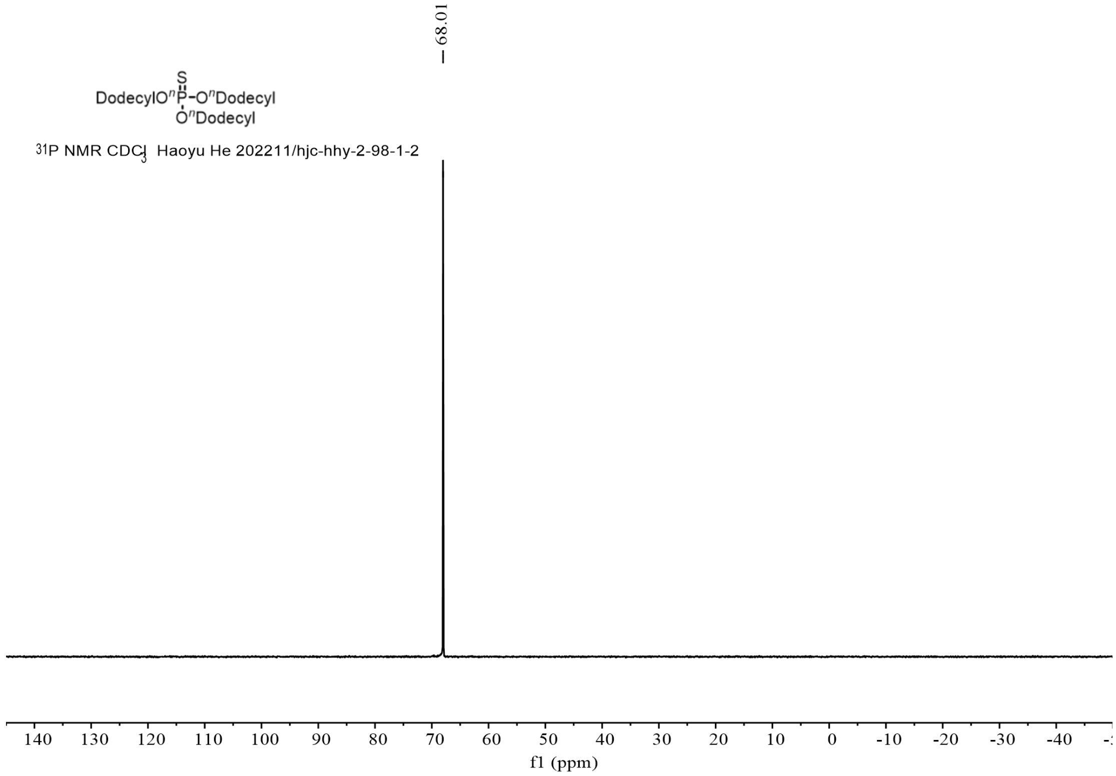
68.01
S
DodecylO^{n}P-O^{n}Dodecyl
O^{n}Dodecyl
31P NMR CDCl3 Haoyu He 202211/hjc-hhy-2-98-1-2
140 130 120 110 100 90 80 70 60 50 40 30 20 10 0 -10 -20 -30 -40
f1 (ppm)

10

^{1}H NMR

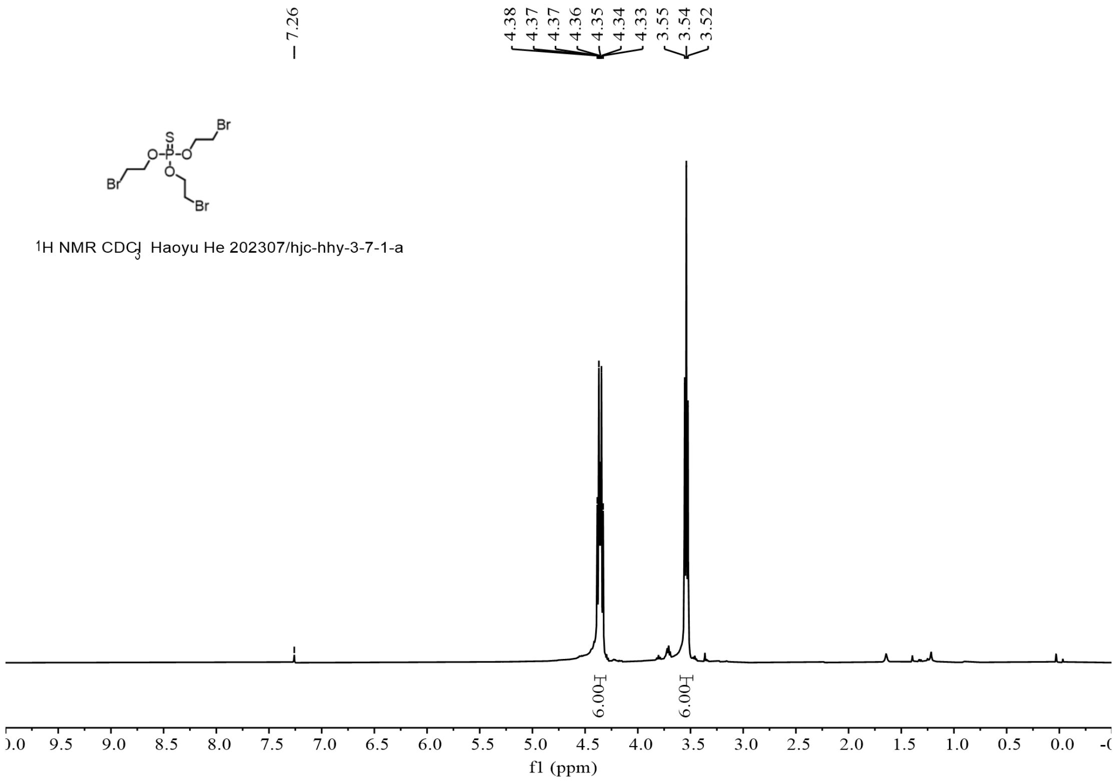

^{13}C NMR

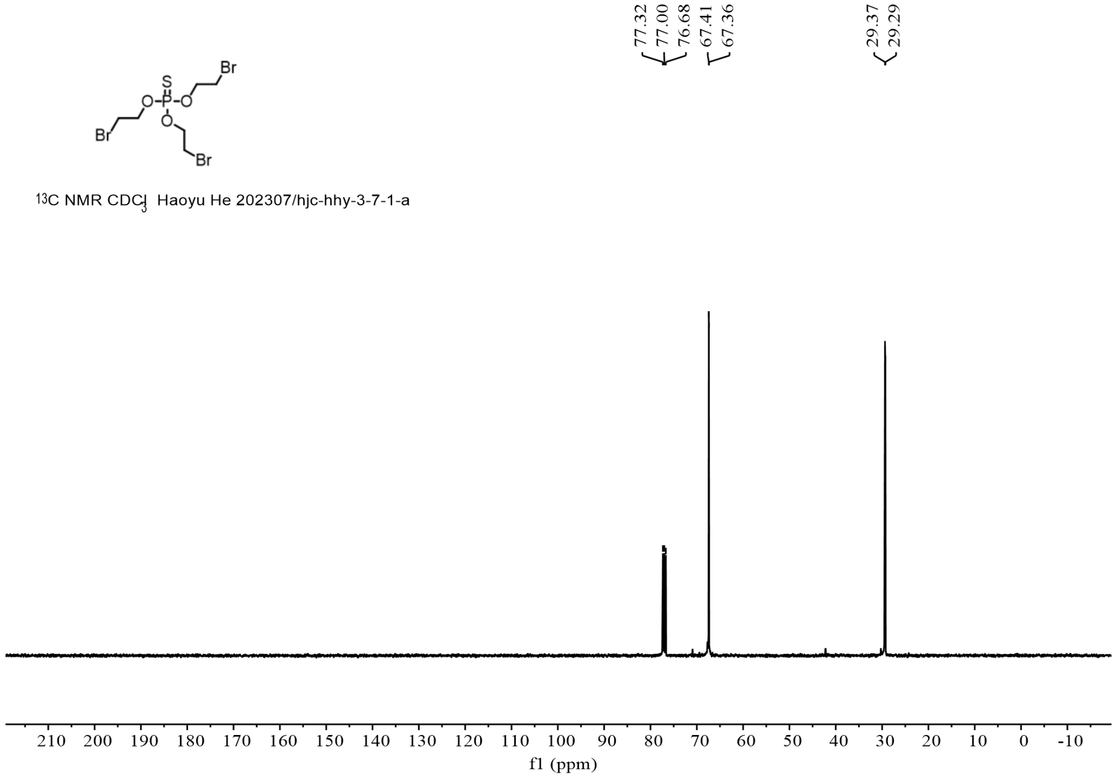

^{31}P NMR

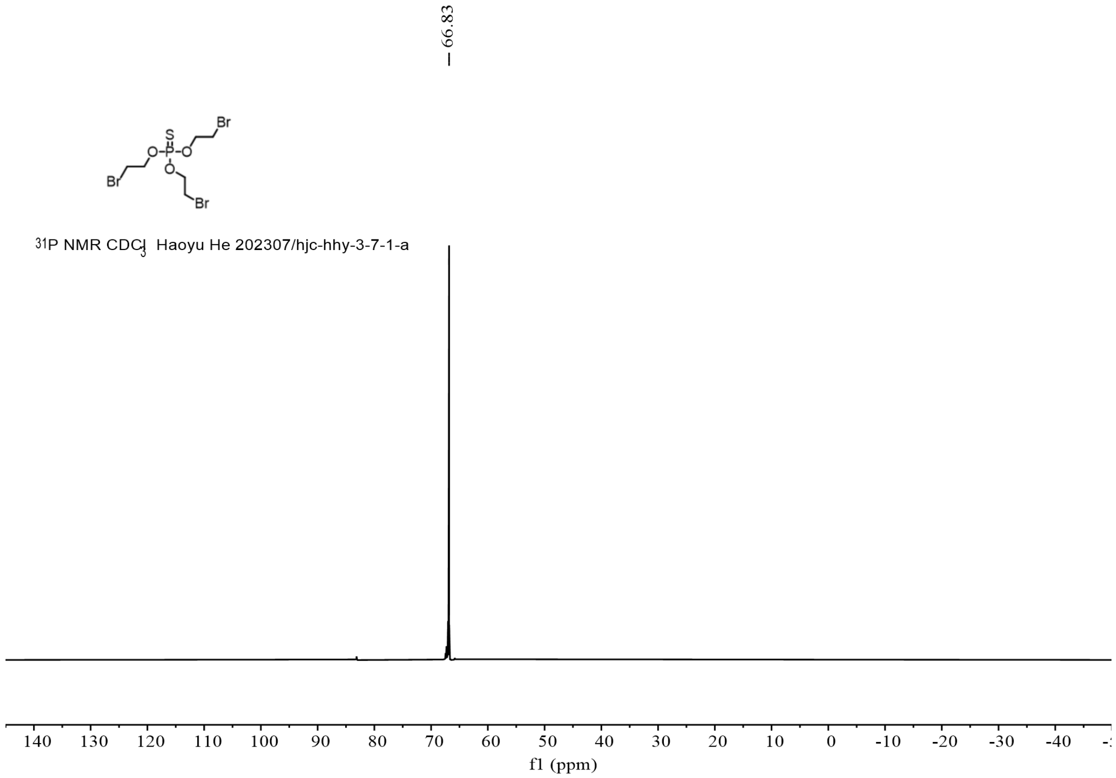

66.83
31P NMR CDCl3 Haoyu He 202307/hjc-hhy-3-7-1-a
140 130 120 110 100 90 80 70 60 50 40 30 20 10 0 -10 -20 -30 -40
f1 (ppm)

11

^{1}H NMR

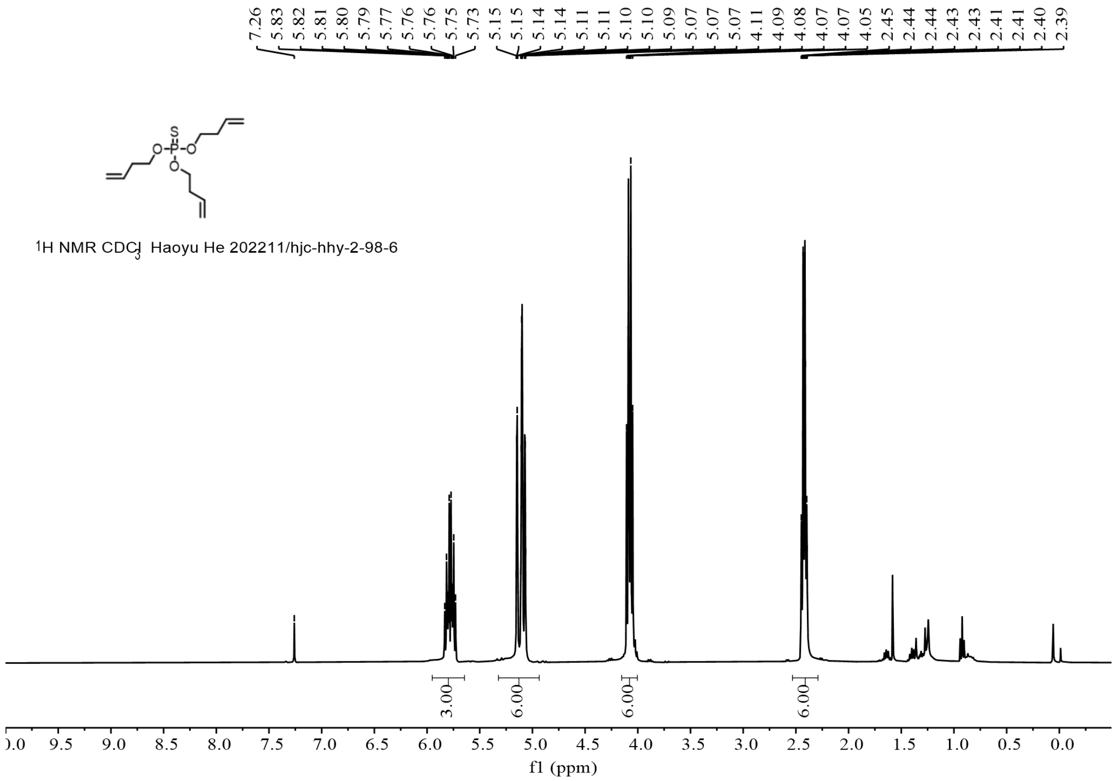

^{13}C NMR

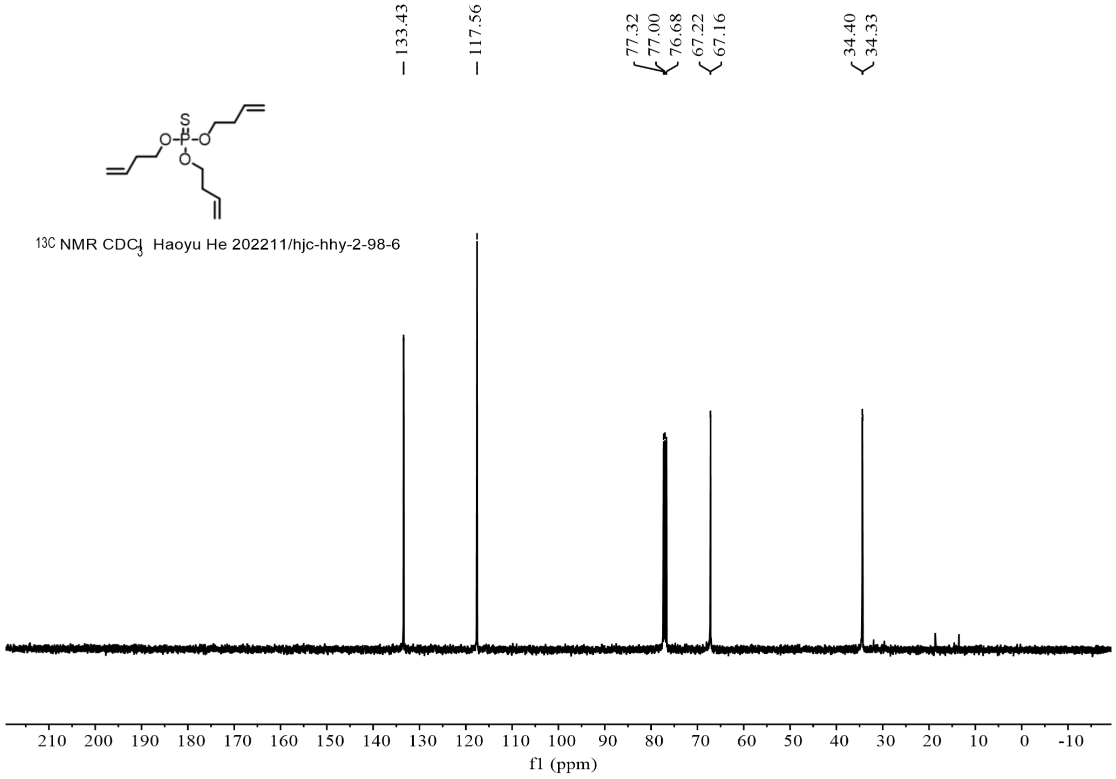

^{31}P NMR

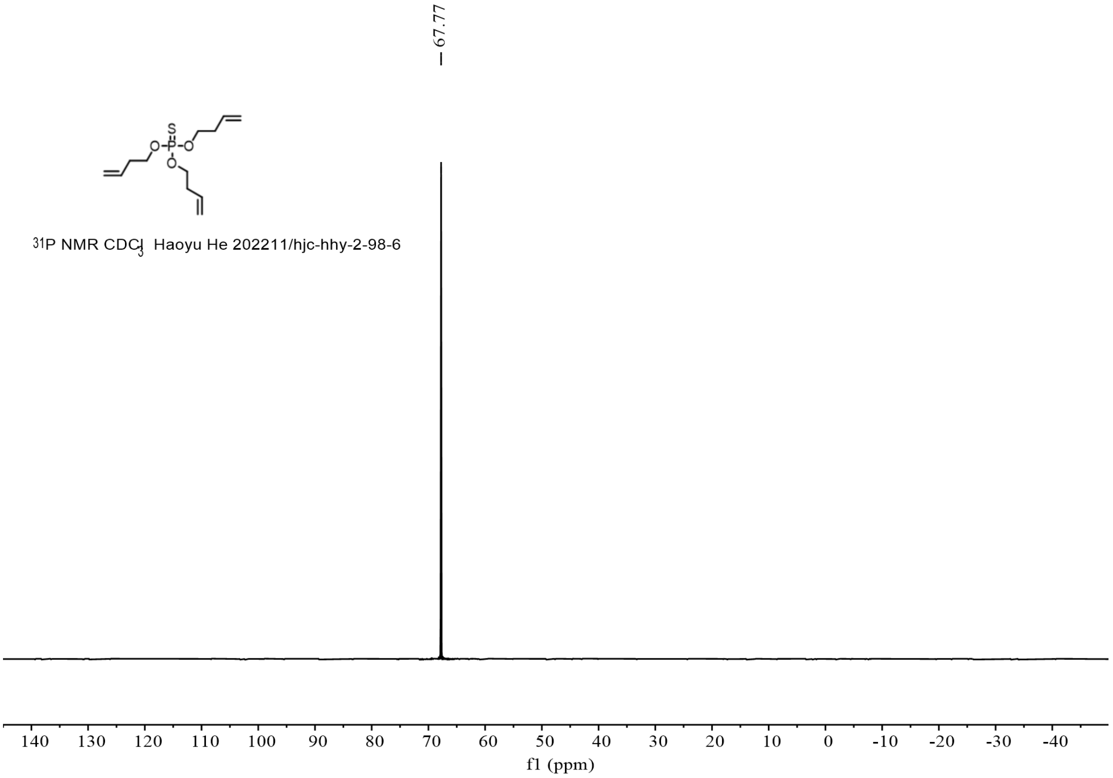

67.77
31P NMR CDCl3 Haoyu He 202211/hjc-hhy-2-98-6
140
130
120
110
100
90
80
70
60
50
40
30
20
10
0
-10
-20
-30
-40
f1 (ppm)

12

^{1}H NMR

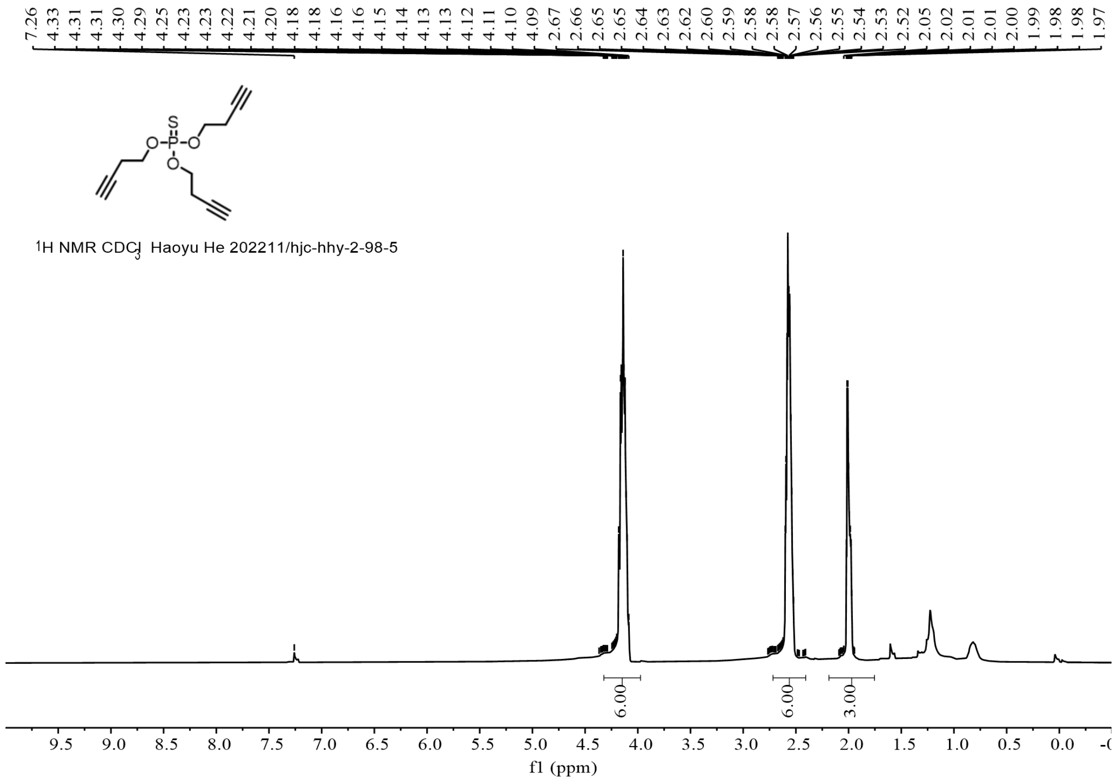

^{13}C NMR

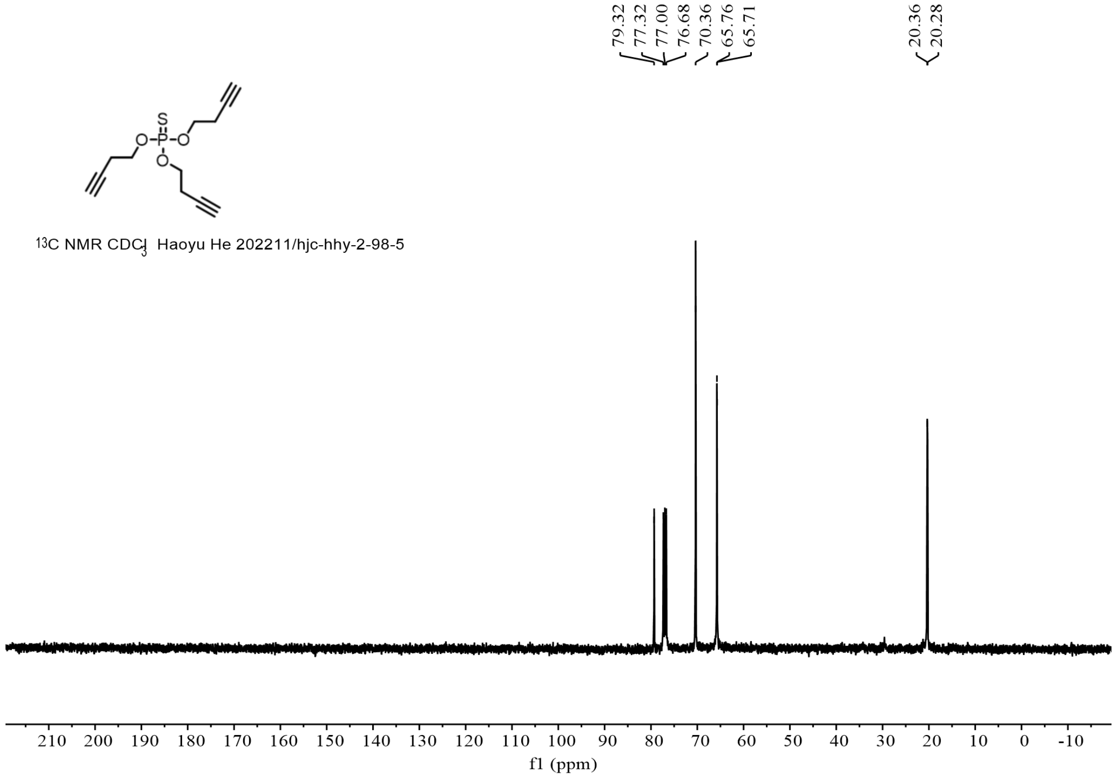

^{31}P NMR

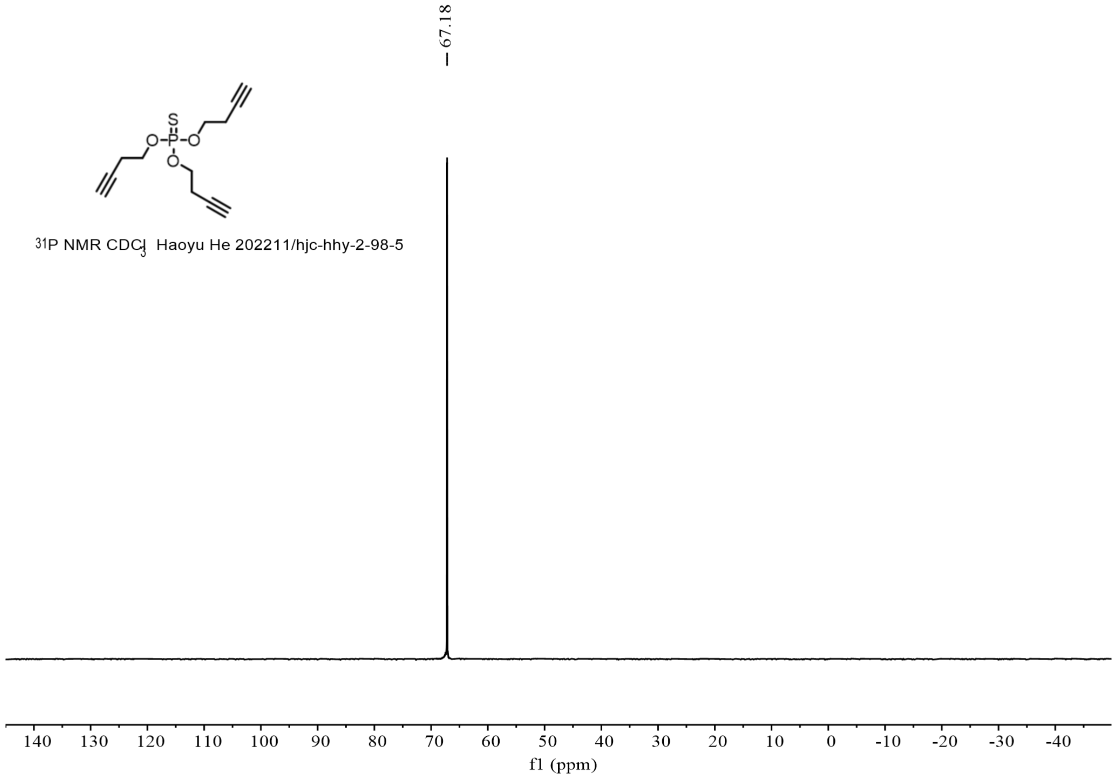

67.18
31P NMR CDCl3 Haoyu He 202211/hjc-hhy-2-98-5
140 130 120 110 100 90 80 70 60 50 40 30 20 10 0 -10 -20 -30 -40
f1 (ppm)

13

^{1}H NMR

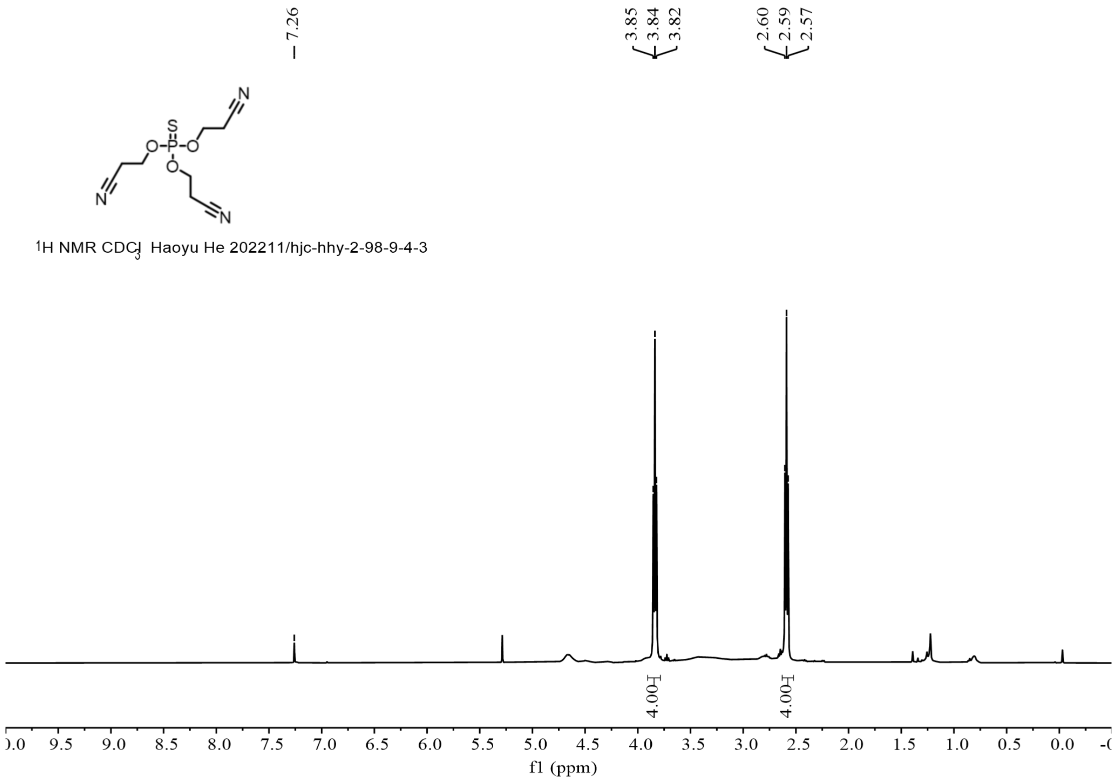

^{13}C NMR

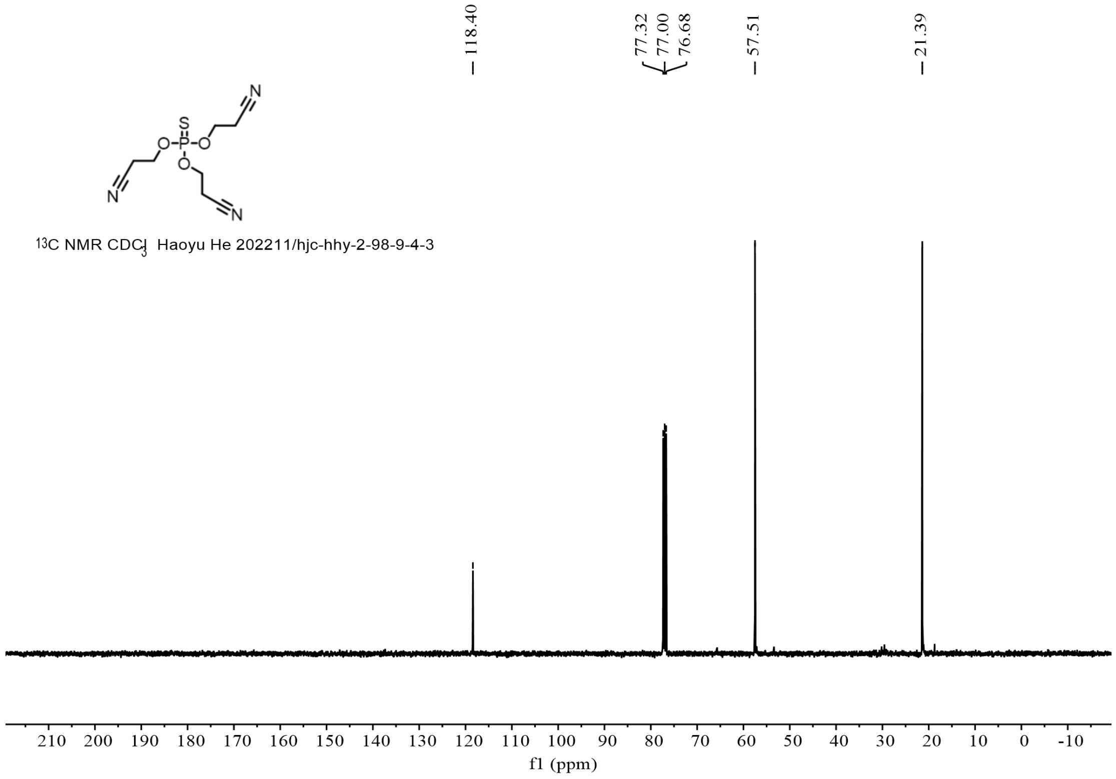

^{31}P NMR

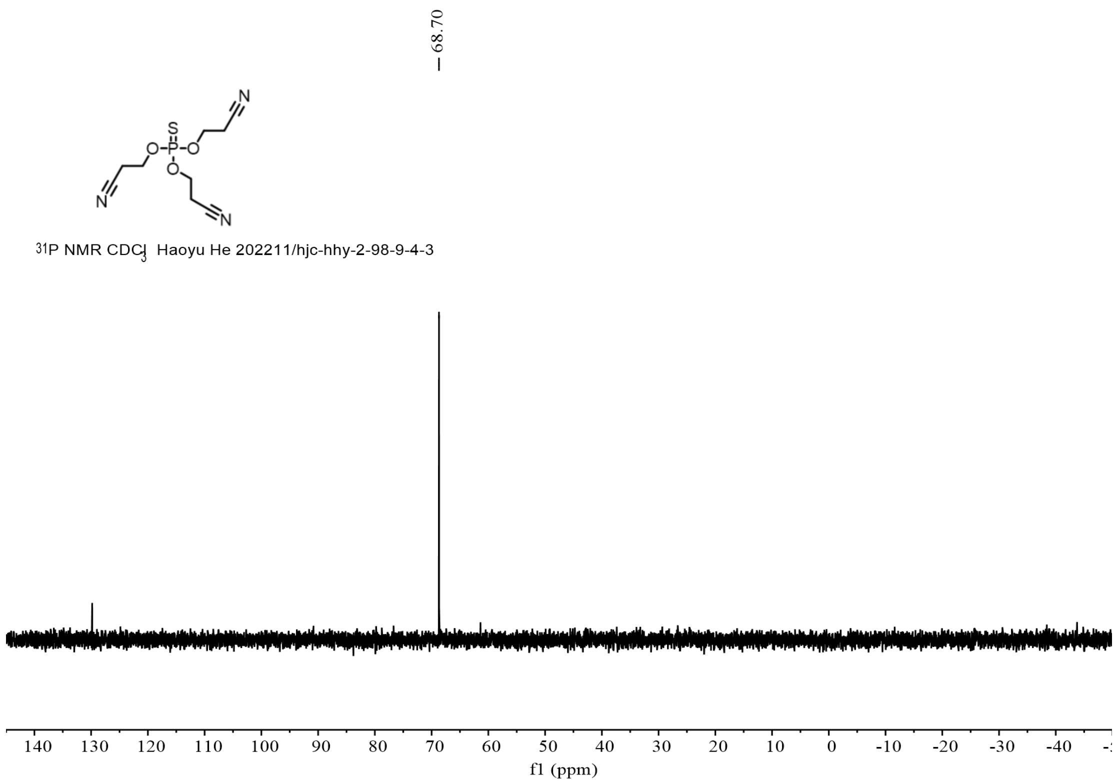

68.70
S
O
P
N
31P NMR CDCl3 Haoyu He 202211/hjc-hhy-2-98-9-4-3
140
130
120
110
100
90
80
70
60
50
40
30
20
10
0
-10
-20
-30
-40
f1 (ppm)

14

^{1}H NMR

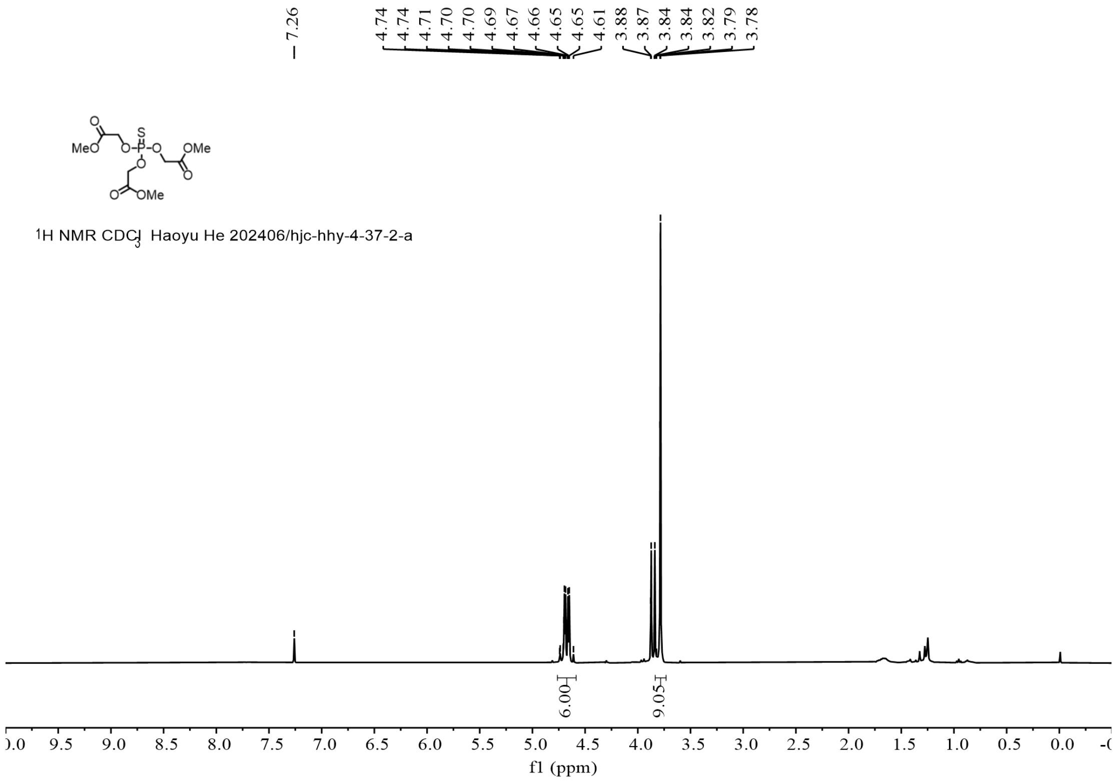

^{13}C NMR

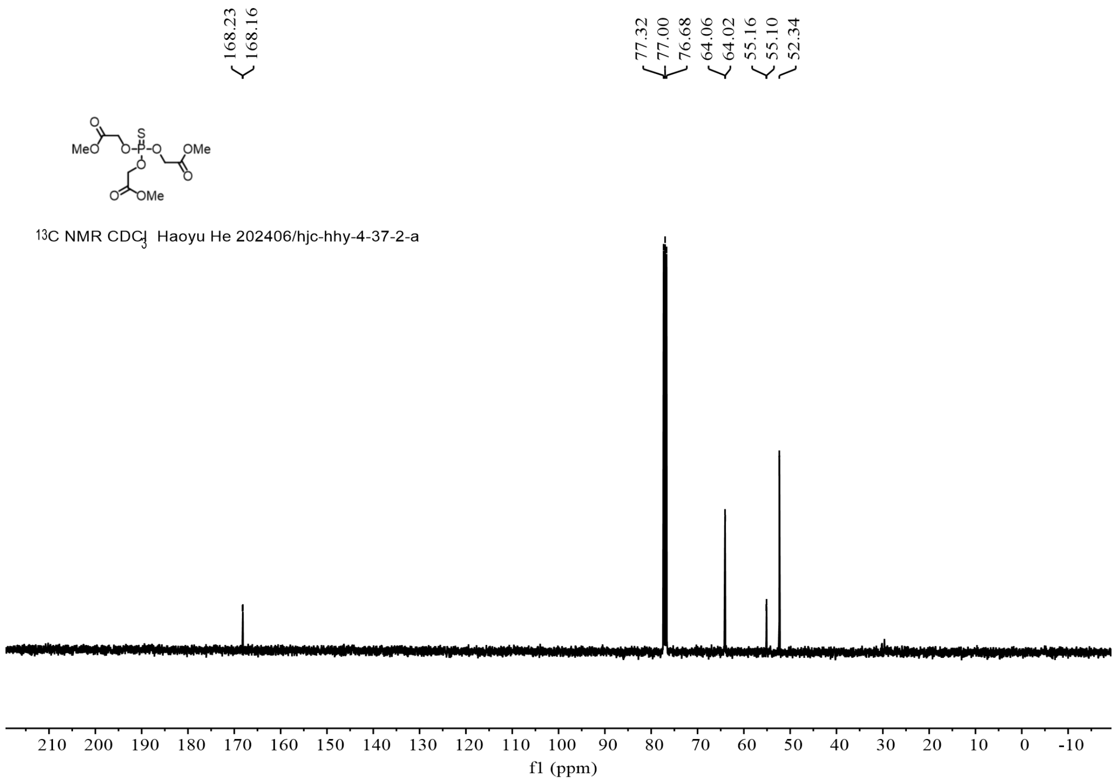

^{31}P NMR

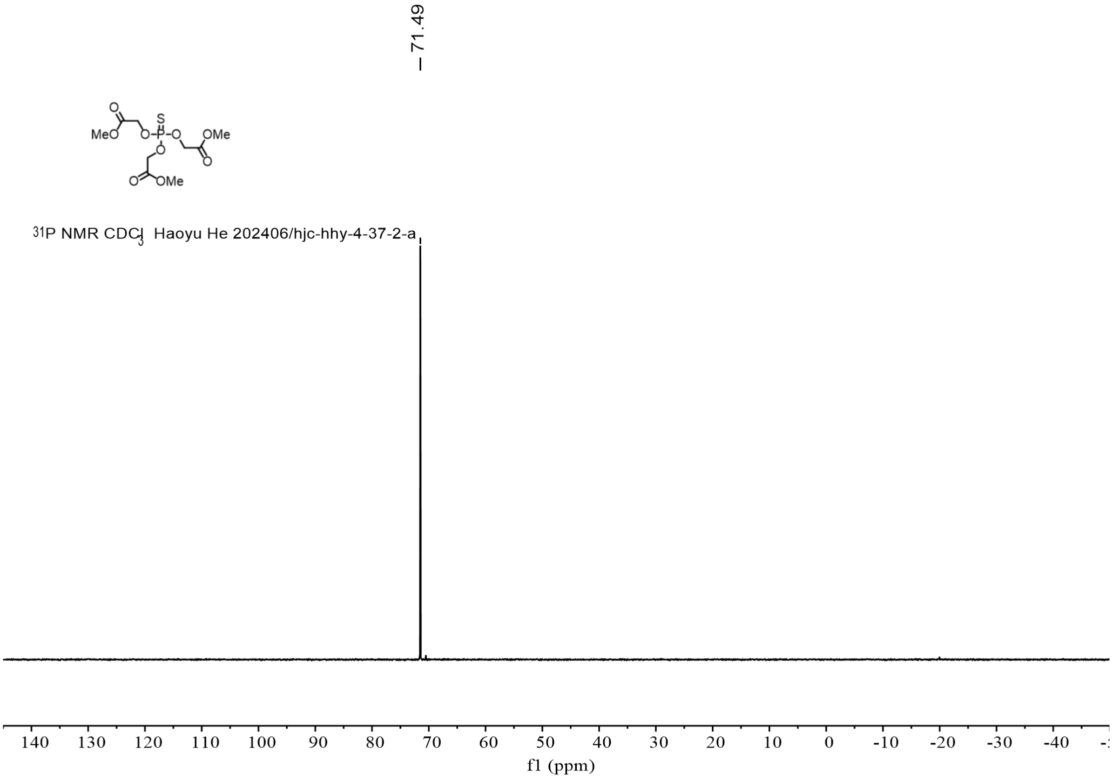

71.49
MeO O P O OMe
S
O
O OMe
31P NMR CDCl3 Haoyu He 202406/hjc-hhy-4-37-2-a
140 130 120 110 100 90 80 70 60 50 40 30 20 10 0 -10 -20 -30 -40
f1 (ppm)

15

^{1}H NMR

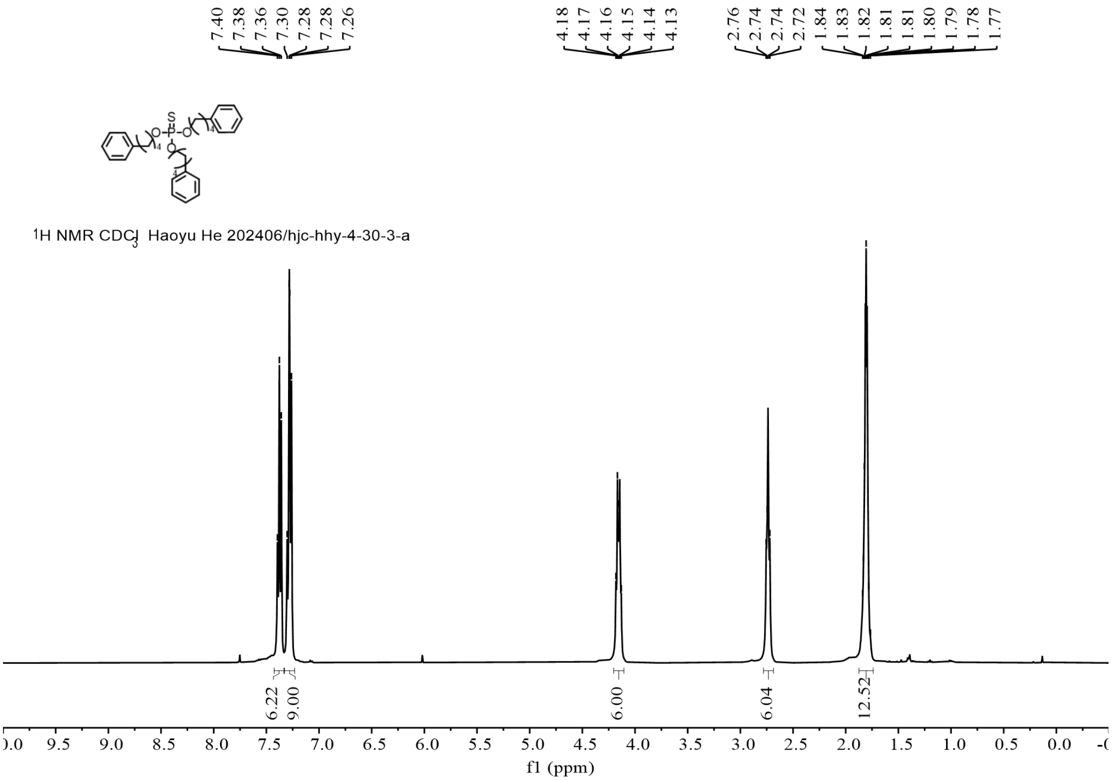

^{13}C NMR

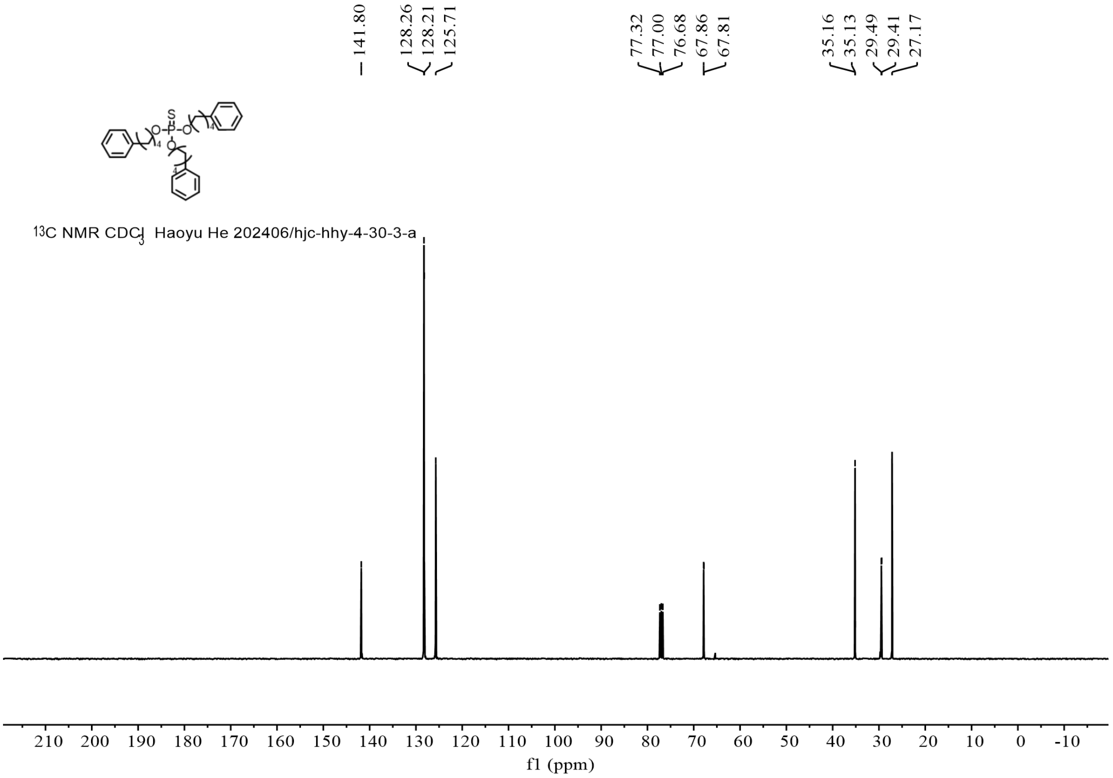

^{31}P NMR

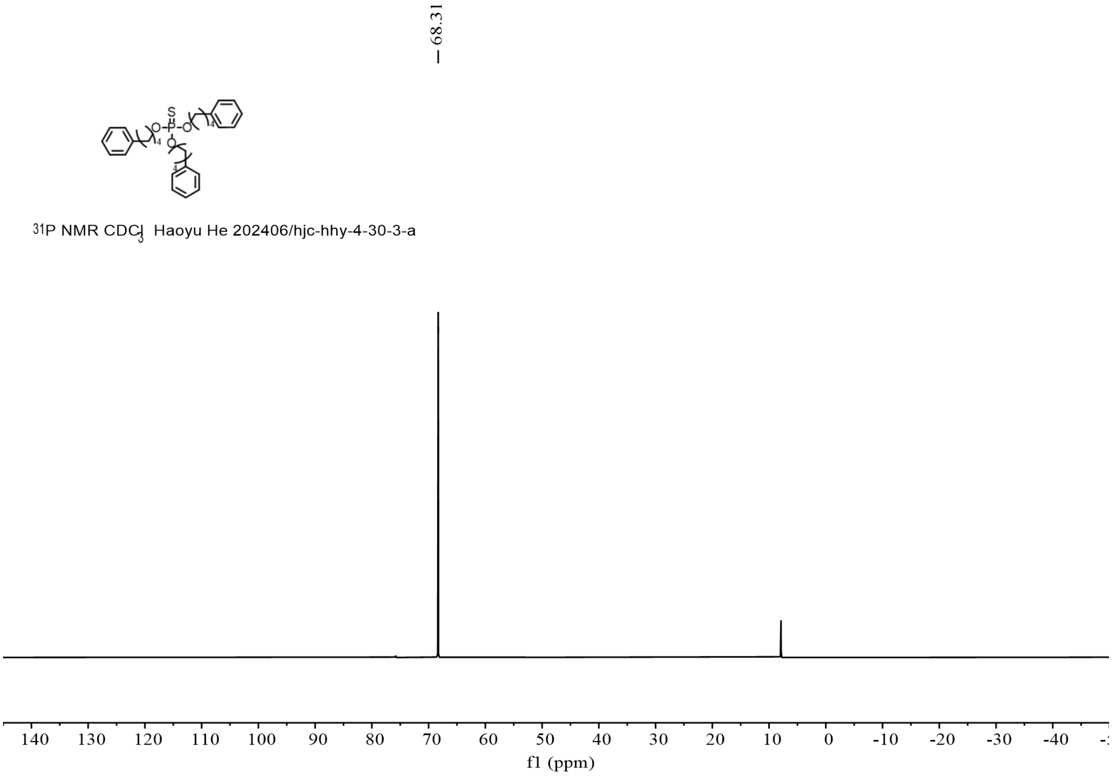
68.31
S
P
O
4
31P NMR CDCl3 Haoyu He 202406/hjc-hhy-4-30-3-a
140 130 120 110 100 90 80 70 60 50 40 30 20 10 0 -10 -20 -30 -40
f1 (ppm)

16

^{1}H NMR

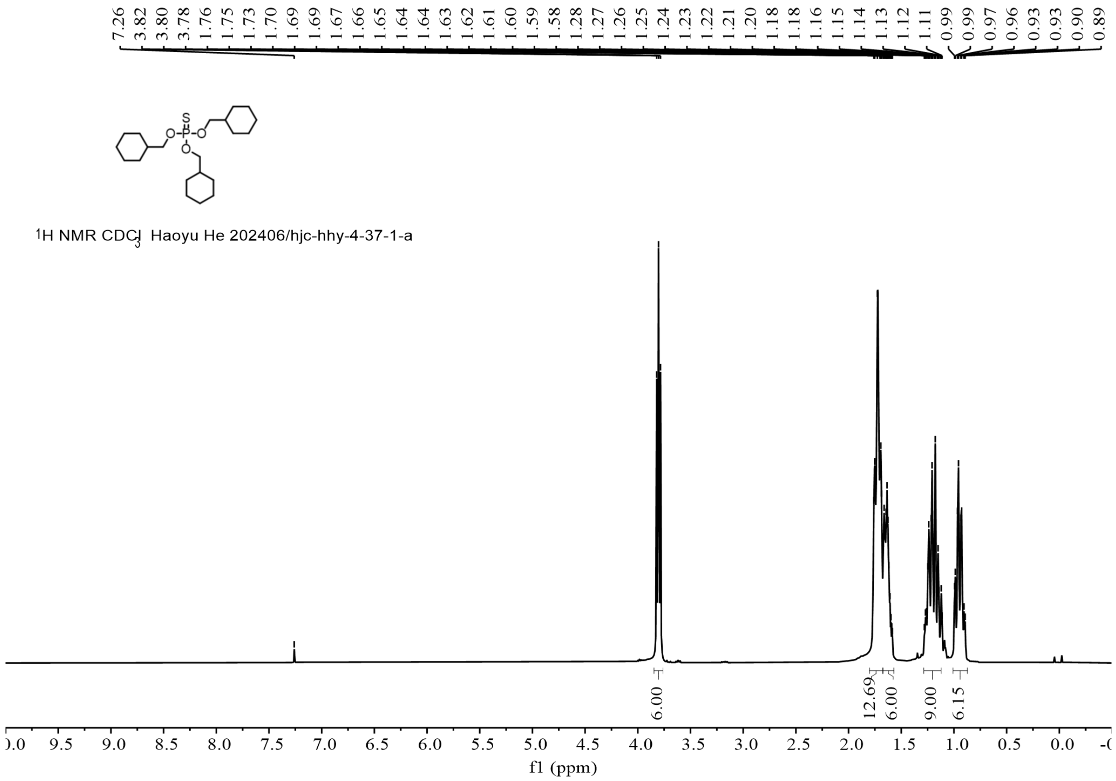

^{13}C NMR

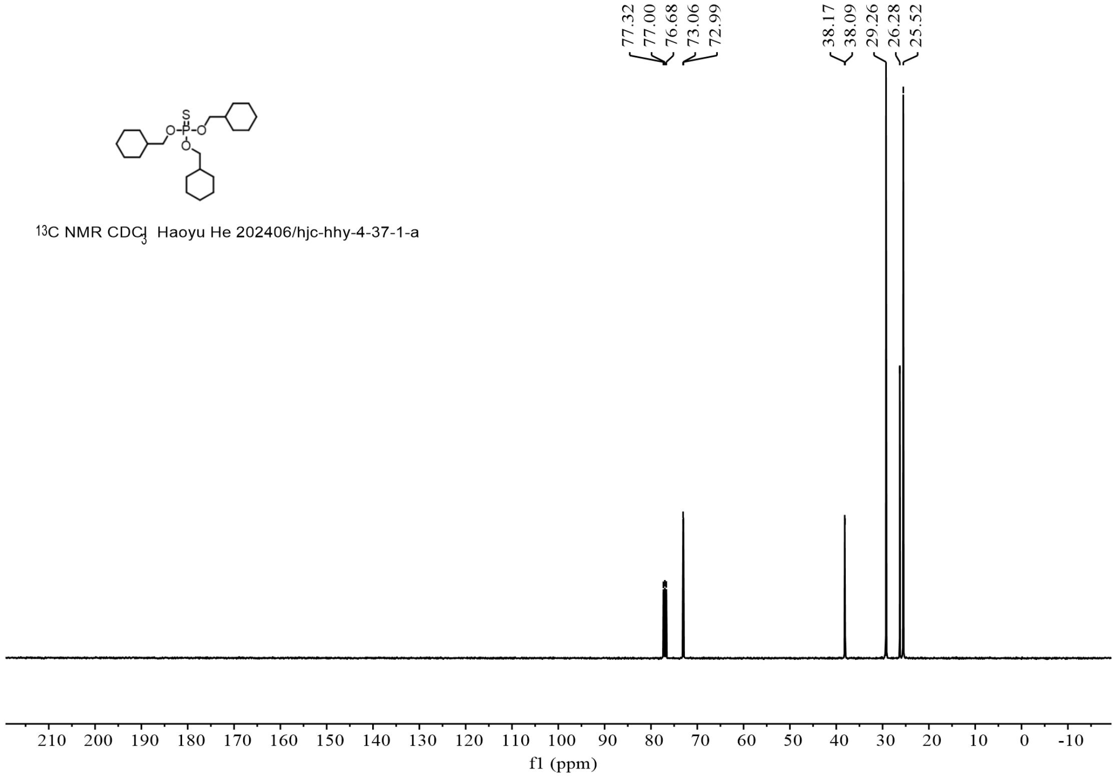

^{31}P NMR

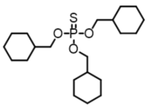

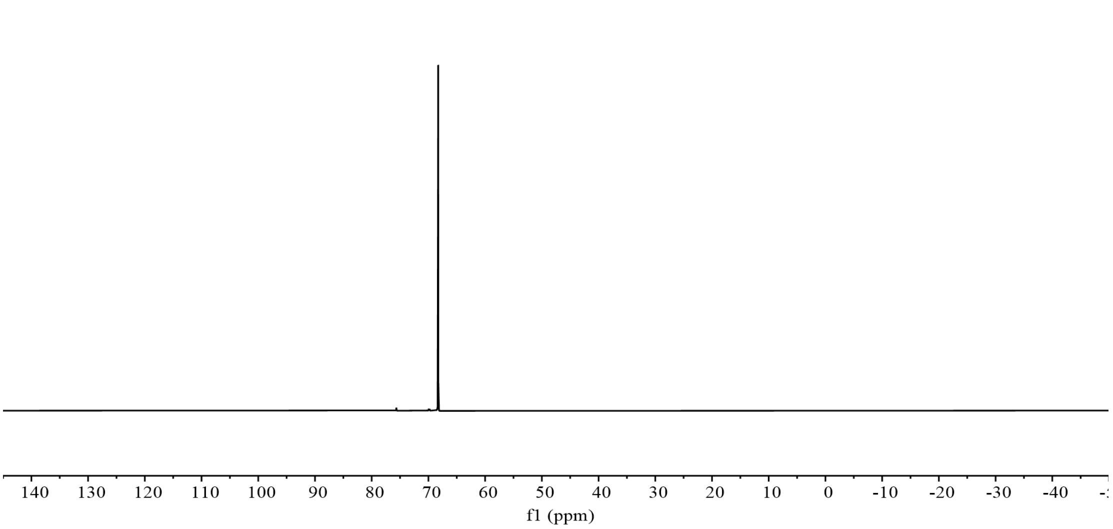
68.27
31P NMR CDCl3 Haoyu He 202406/hjc-hhy-4-37-1-a
140
130
120
110
100
90
80
70
60
50
40
30
20
10
0
-10
-20
-30
-40
f1 (ppm)

17

^{1}H NMR

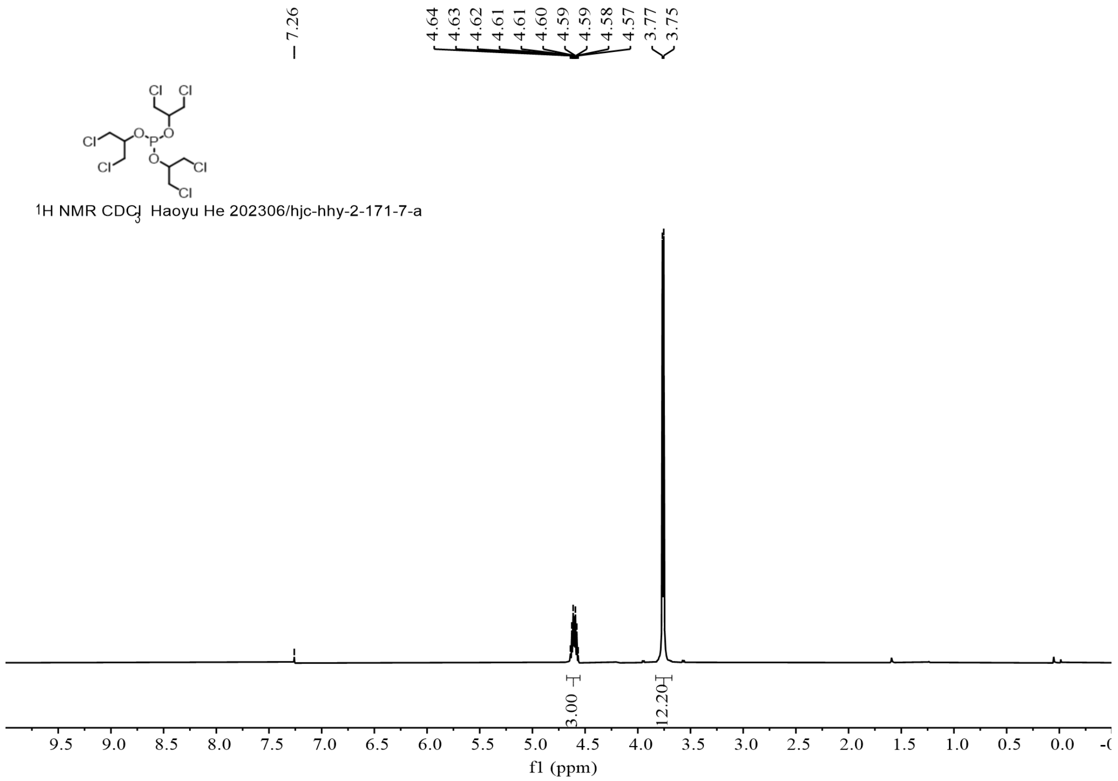

^{13}C NMR

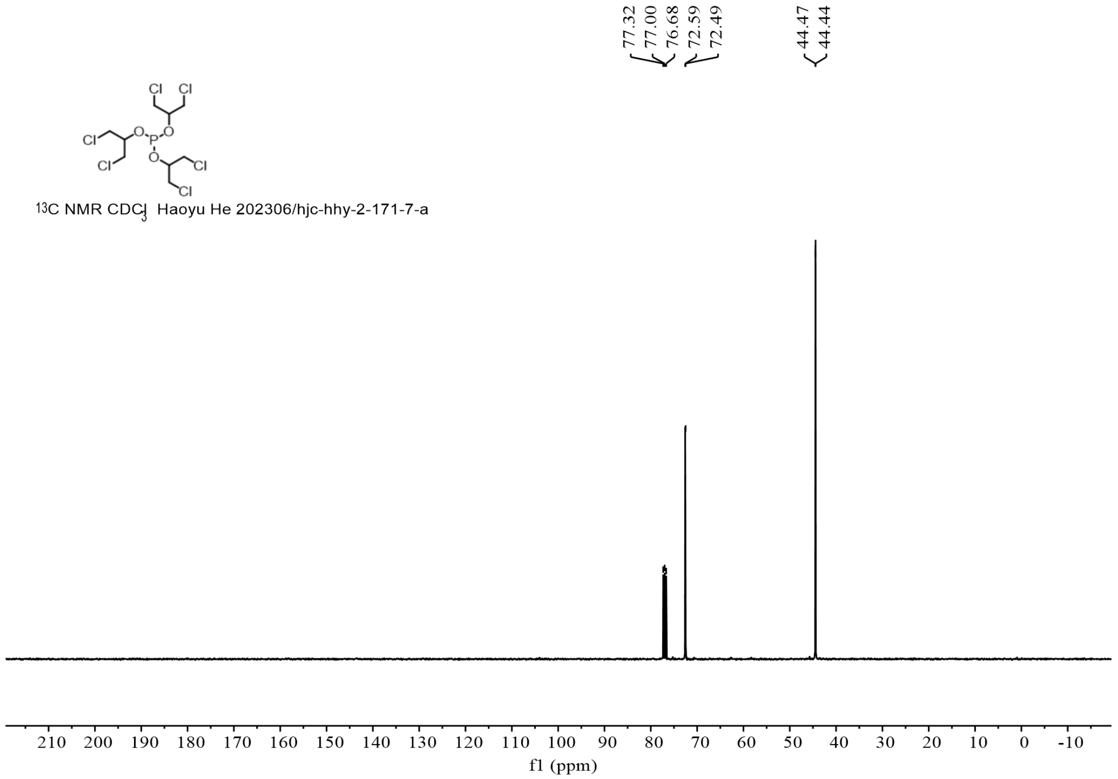

^{31}P NMR

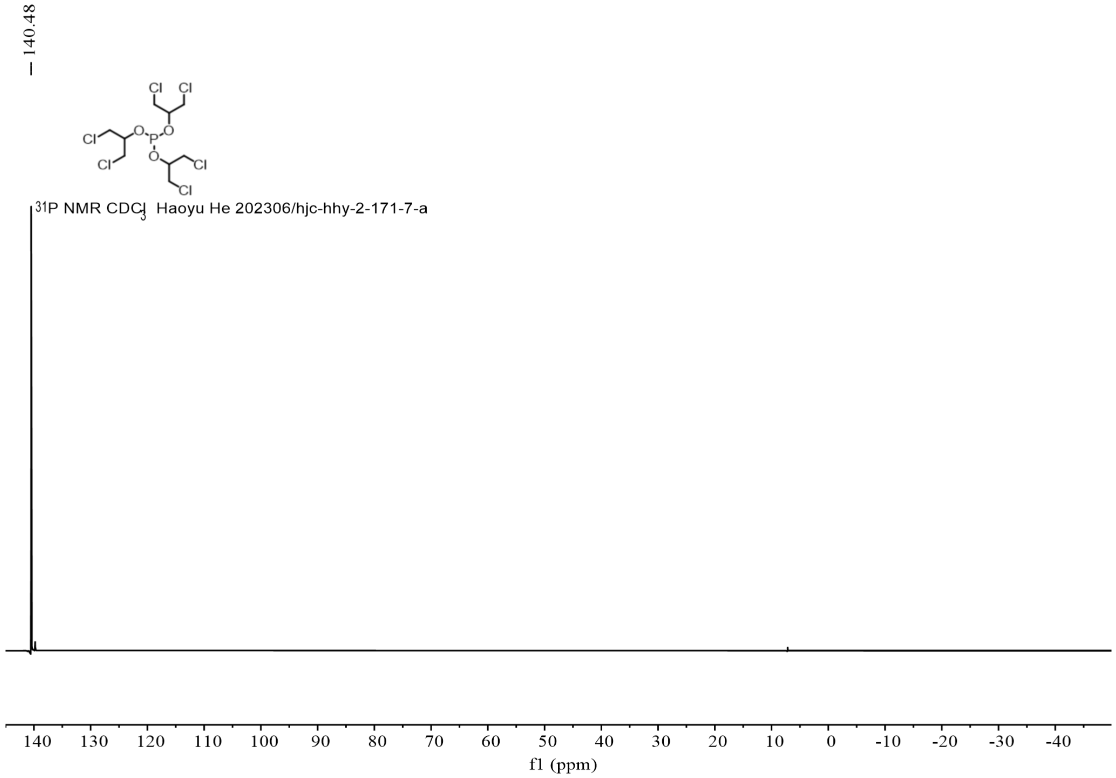

— 140.48
Cl
Cl
O
P
Cl
Cl
Cl
Cl
^{31}P NMR CDCl$_3$ Haoyu He 202306/hjc-hhy-2-171-7-a
140 130 120 110 100 90 80 70 60 50 40 30 20 10 0 -10 -20 -30 -40
f1 (ppm)

18

^{1}H NMR

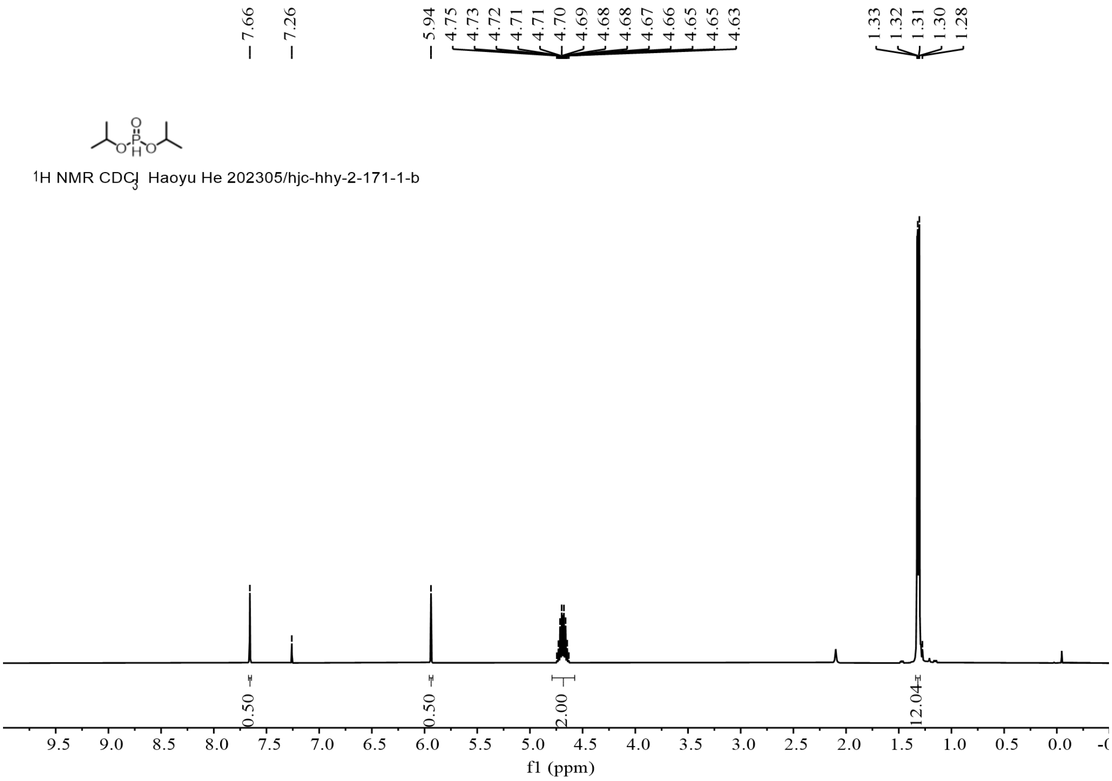

^{13}C NMR

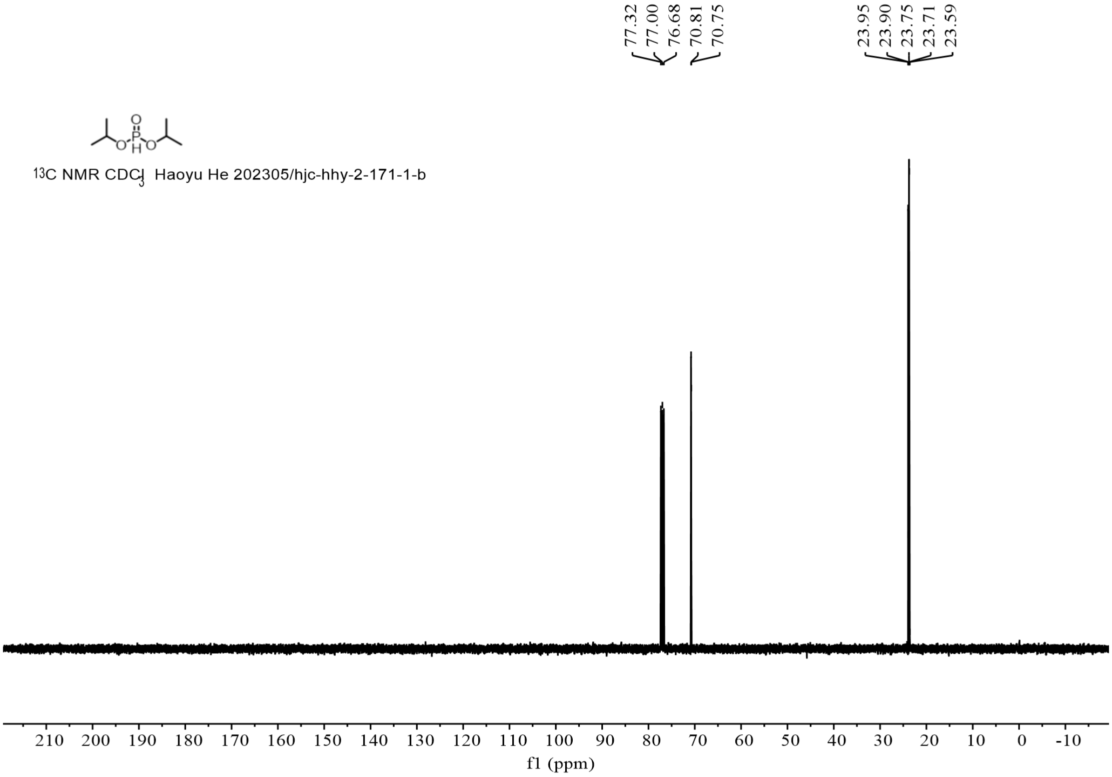

^{31}P NMR

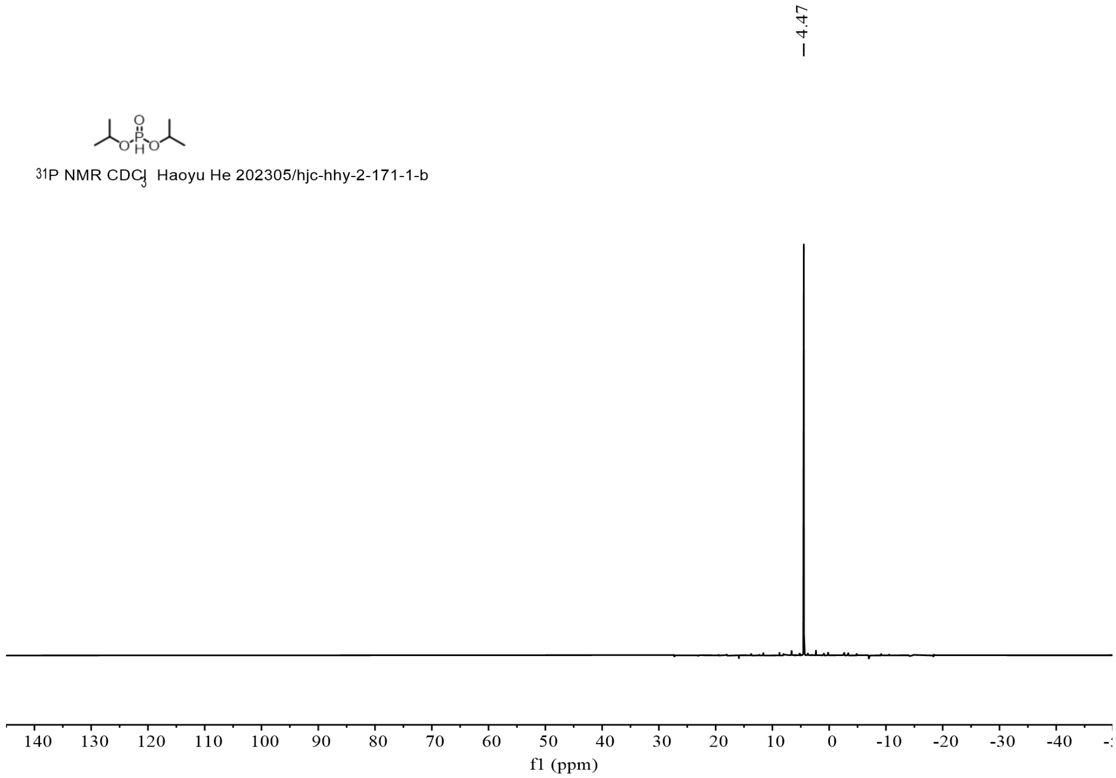

4.47
31P NMR CDCl3 Haoyu He 202305/hjc-hhy-2-171-1-b
140
130
120
110
100
90
80
70
60
50
40
30
20
10
0
-10
-20
-30
-40
f1 (ppm)

19

^{1}H NMR

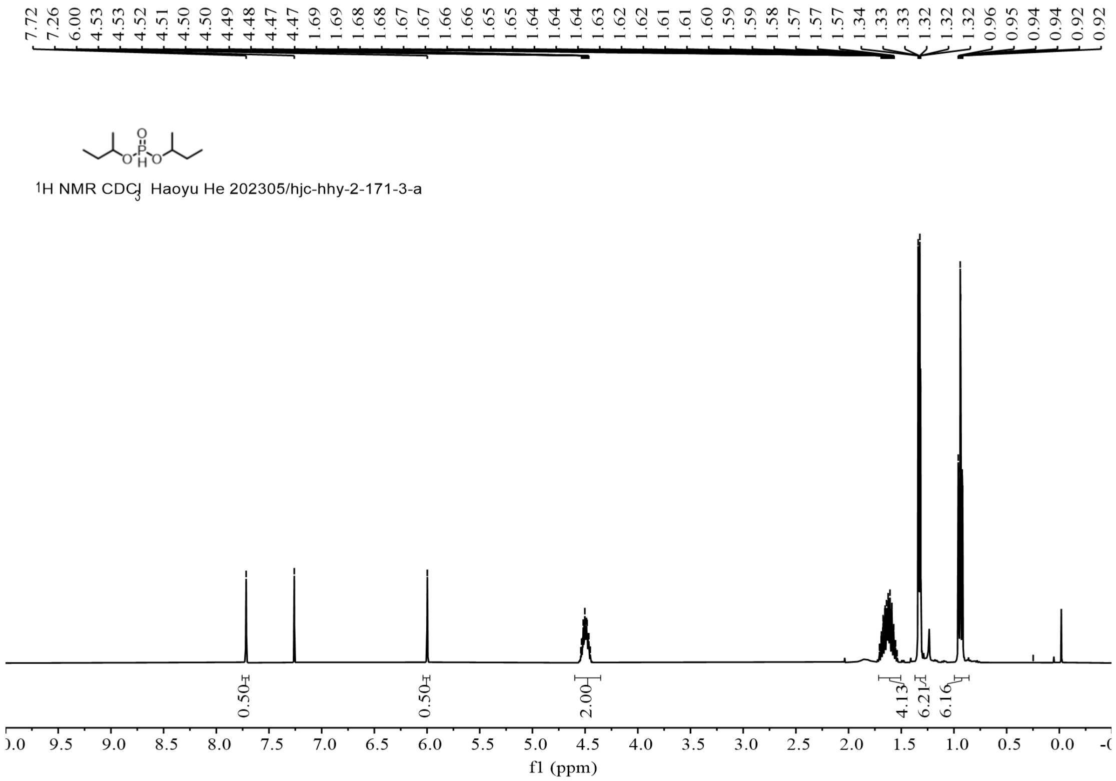

^{13}C NMR

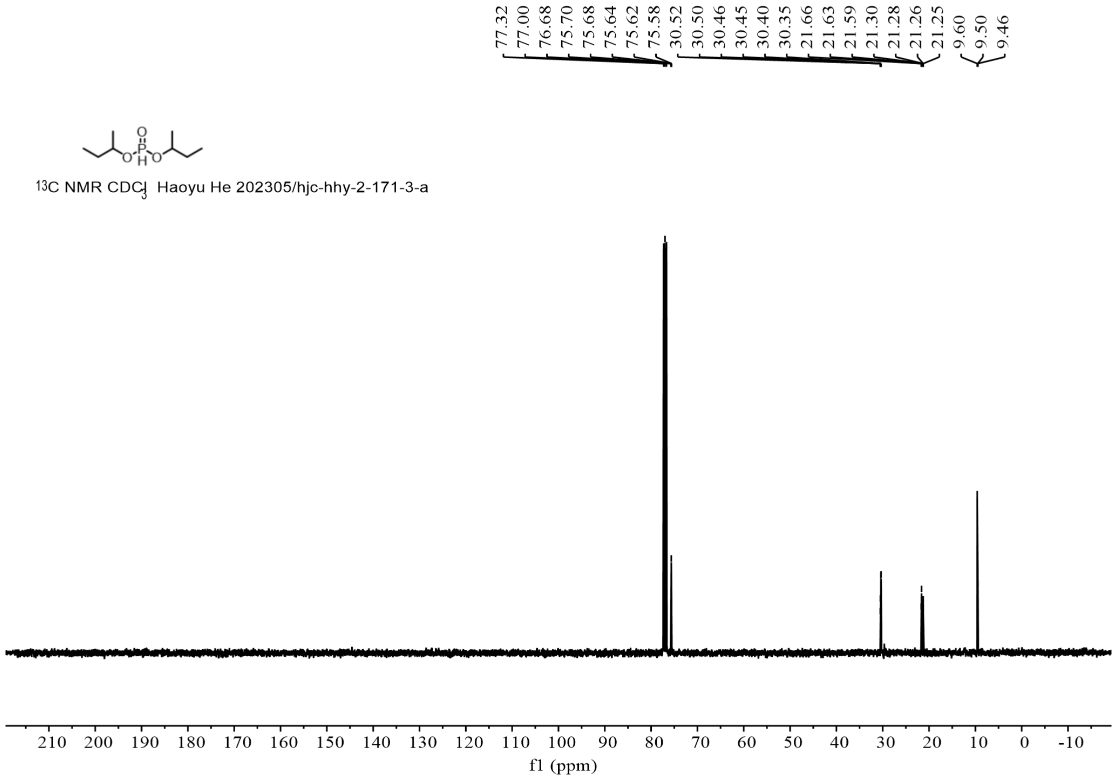

^{31}P NMR

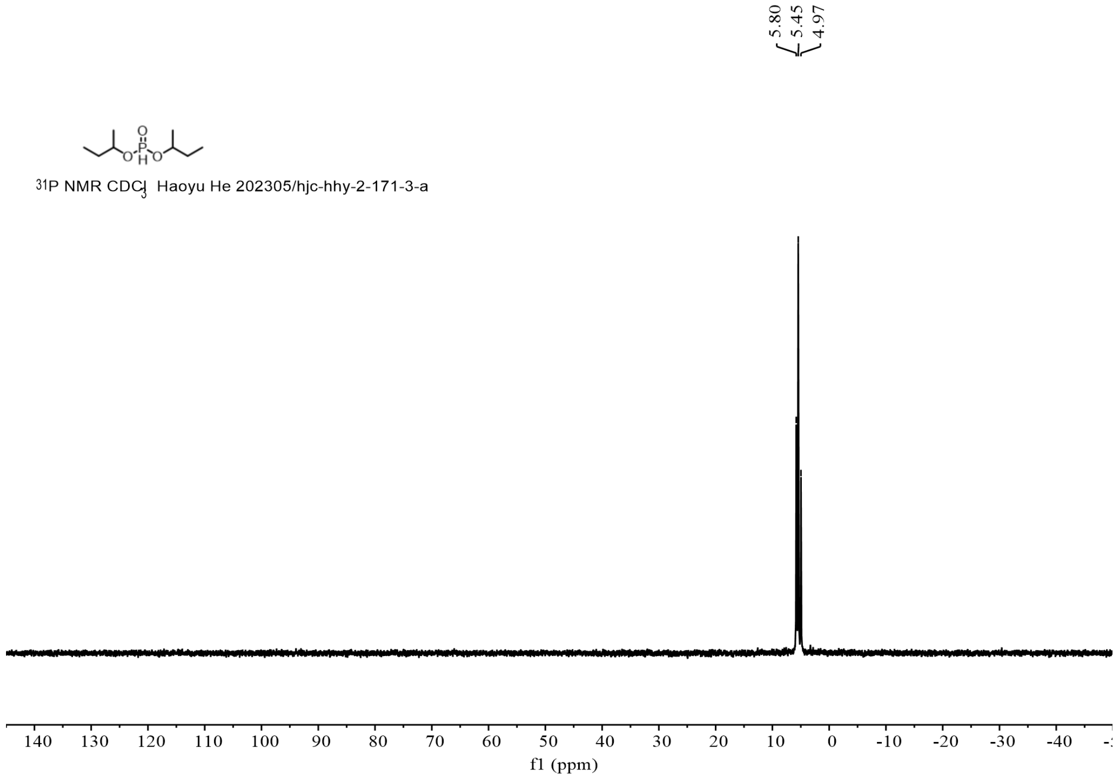

5.80
5.45
4.97
31P NMR CDCl3 Haoyu He 202305/hjc-hhy-2-171-3-a
140
130
120
110
100
90
80
70
60
50
40
30
20
10
0
-10
-20
-30
-40
f1 (ppm)

20

^{1}H NMR

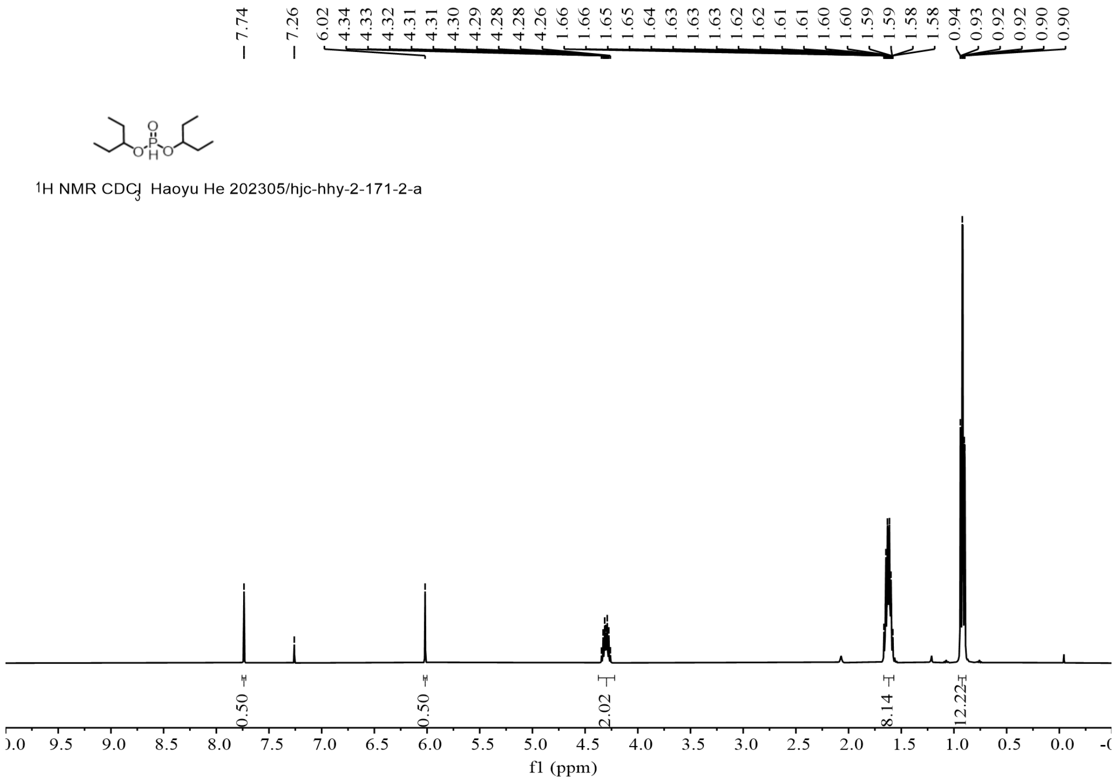

^{13}C NMR

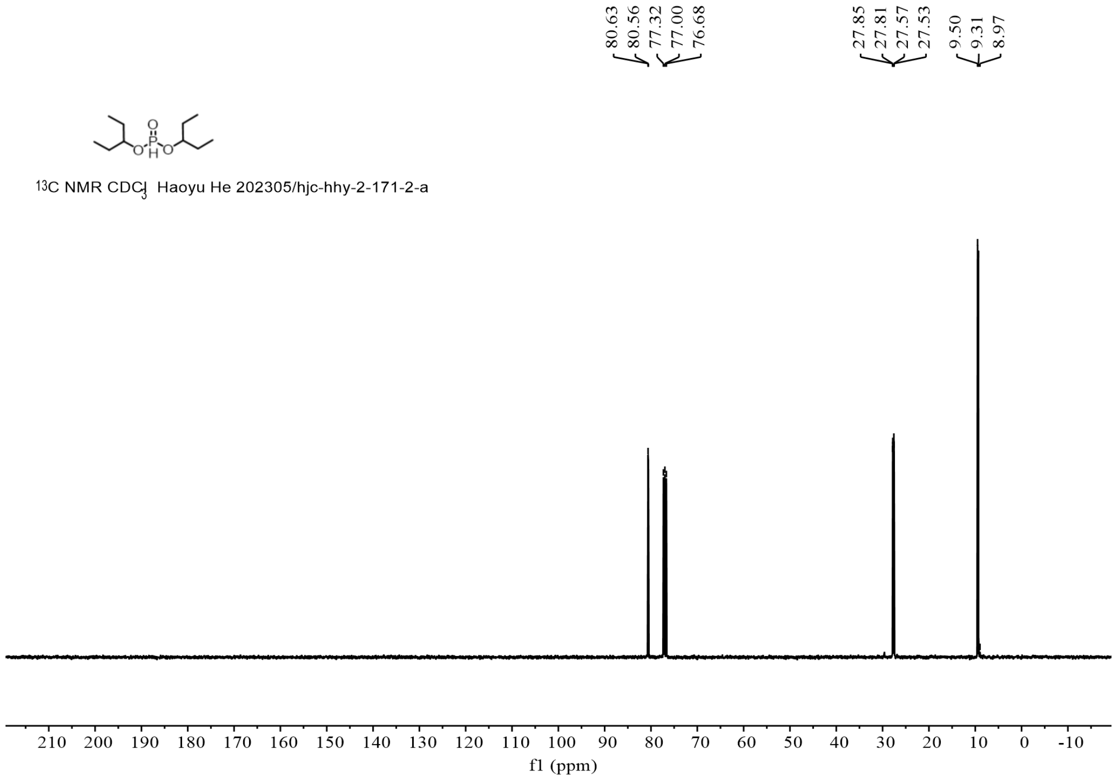

^{31}P NMR

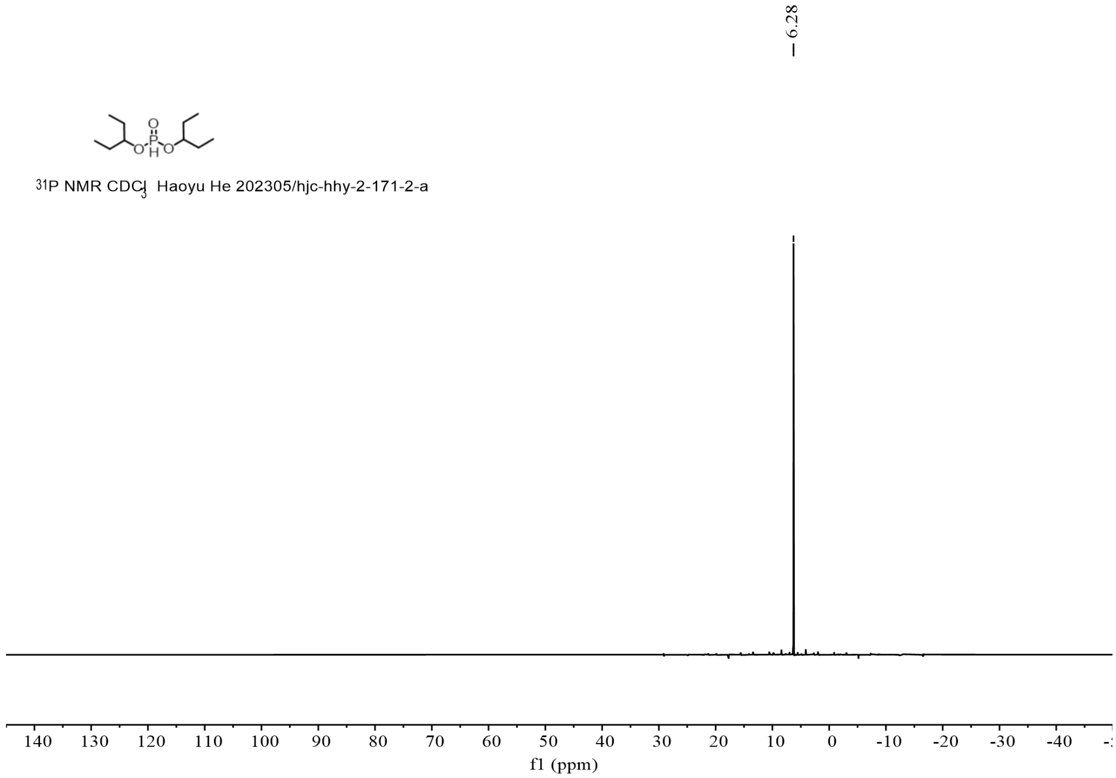

6.28
31P NMR CDCl3 Haoyu He 202305/hjc-hhy-2-171-2-a
140
130
120
110
100
90
80
70
60
50
40
30
20
10
0
-10
-20
-30
-40
f1 (ppm)

21

^{1}H NMR

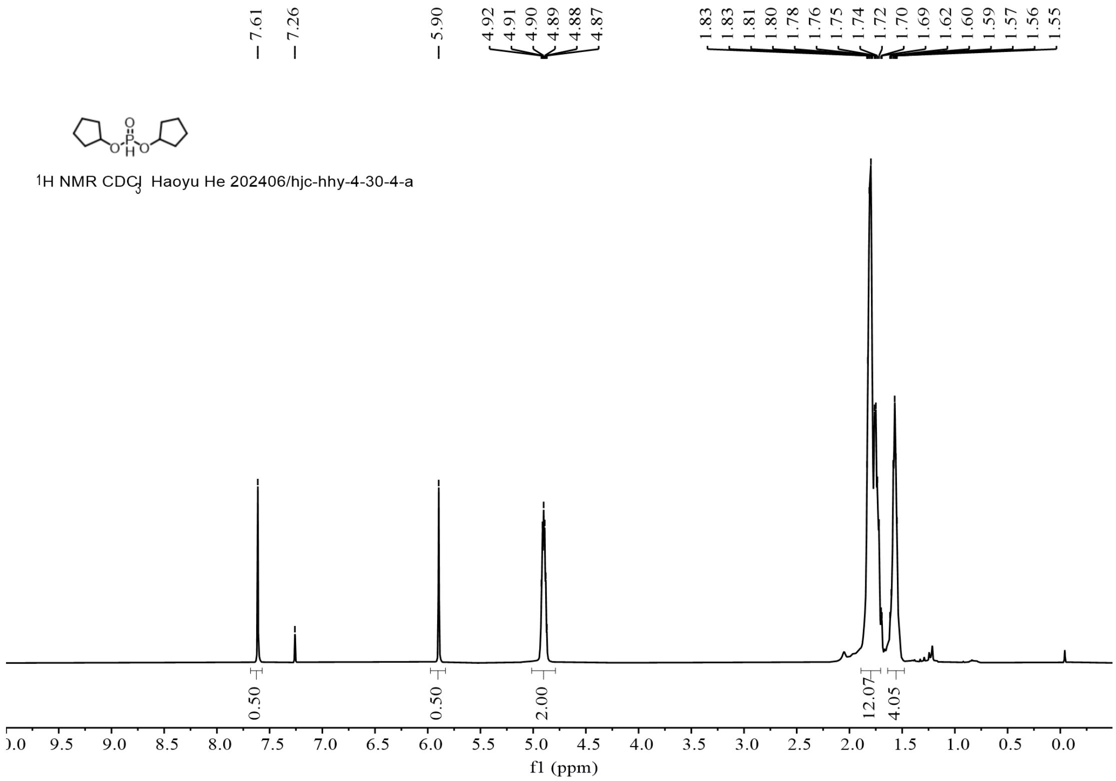

^{13}C NMR

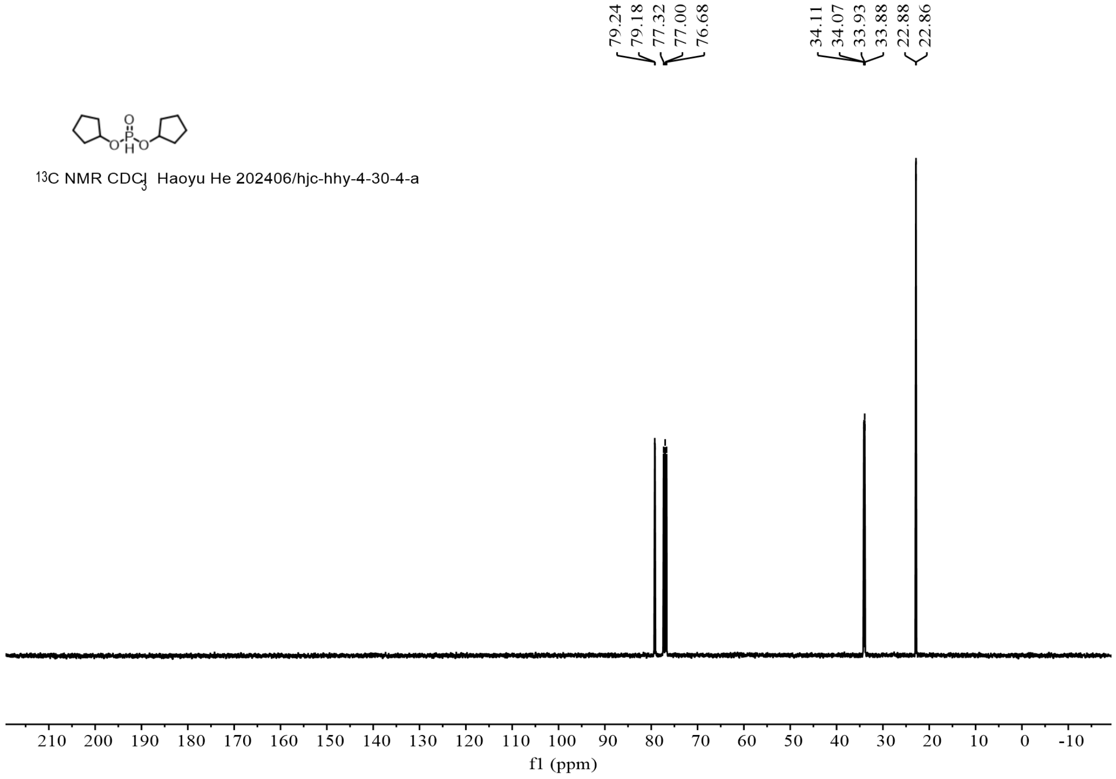

^{31}P NMR

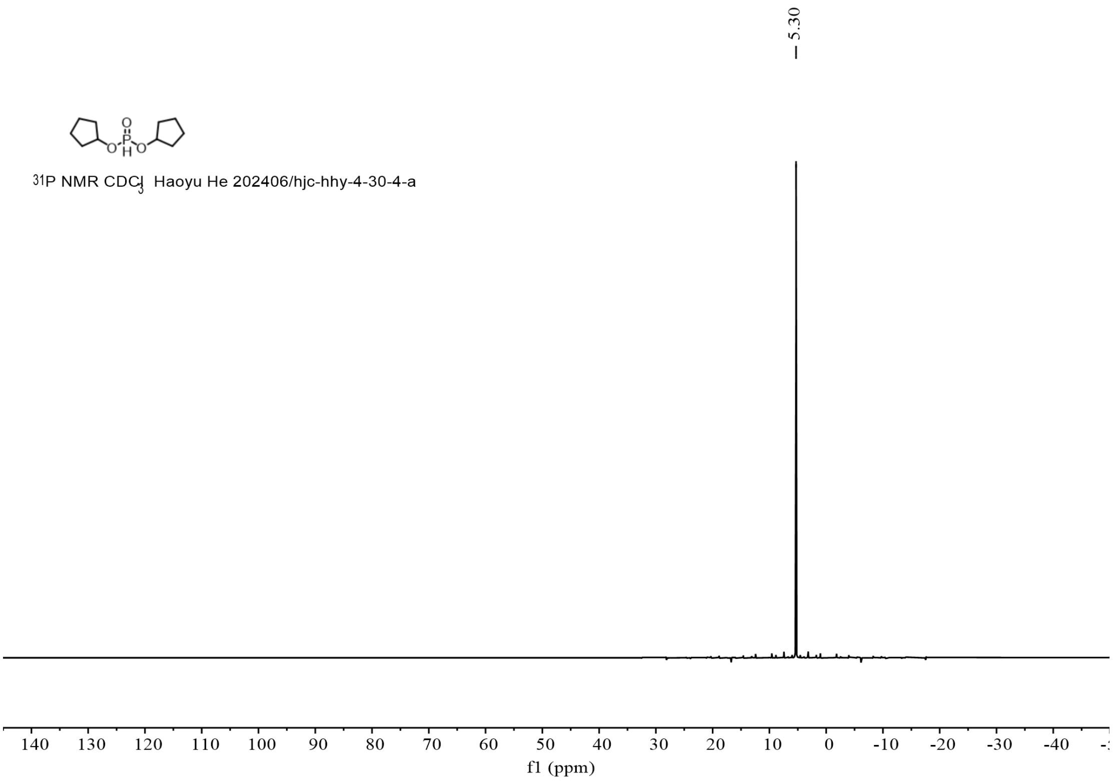
5.30
31P NMR CDCl3 Haoyu He 202406/hjc-hhy-4-30-4-a
140
130
120
110
100
90
80
70
60
50
40
30
20
10
0
-10
-20
-30
-40
f1 (ppm)

22

^{1}H NMR

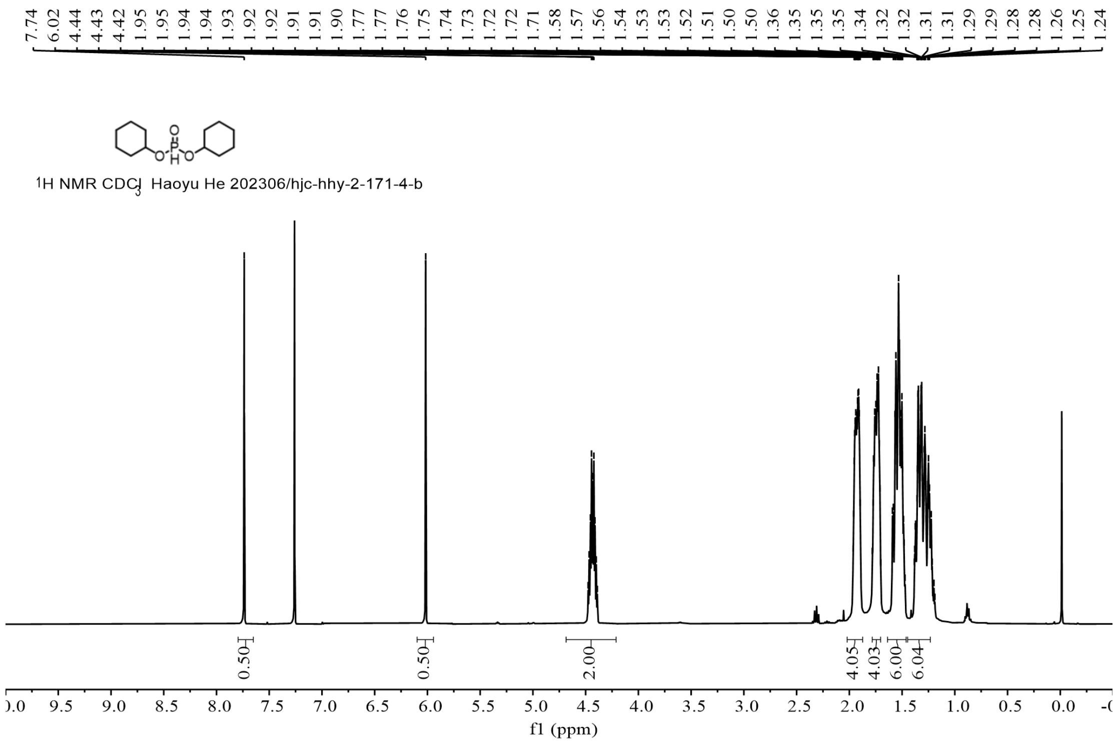

^{13}C NMR

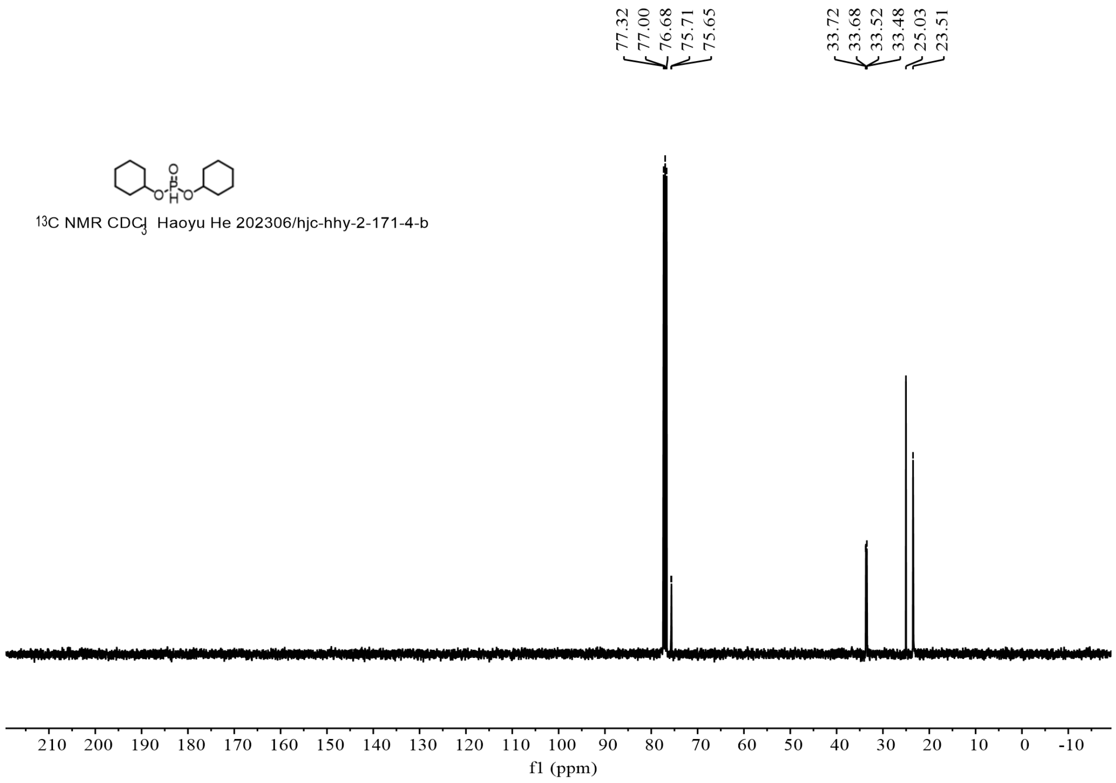

^{31}P NMR

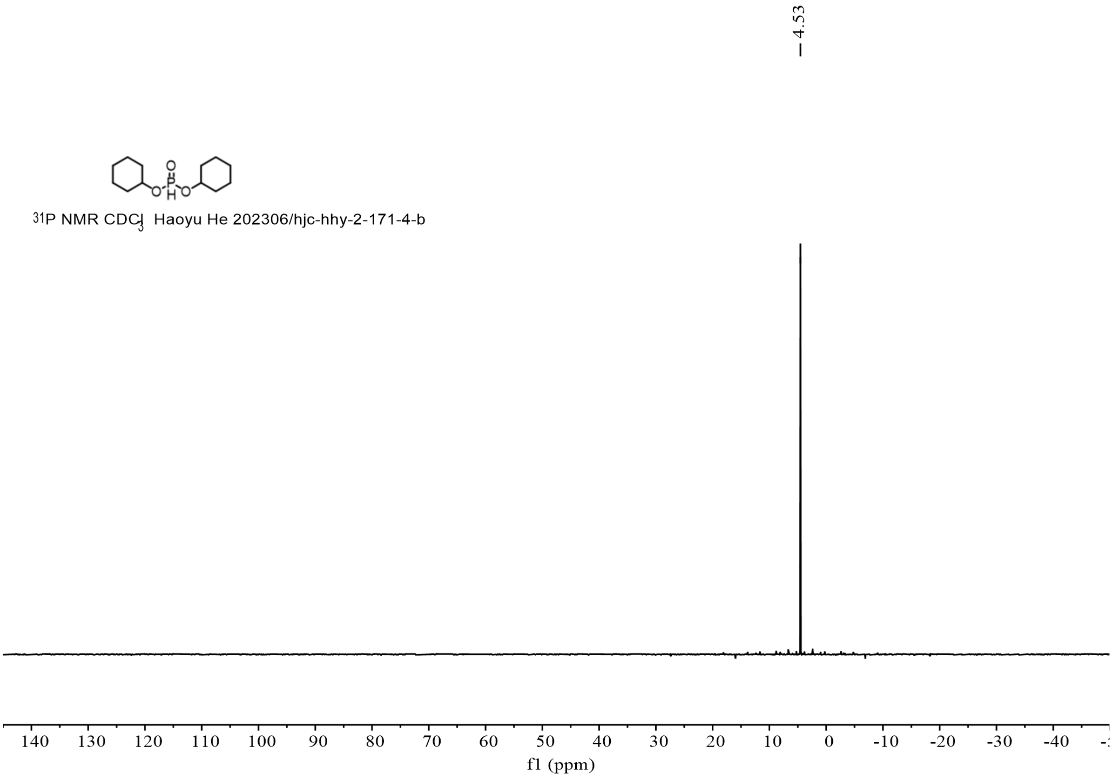

4.53
31P NMR CDCl3 Haoyu He 202306/hjc-hhy-2-171-4-b
140 130 120 110 100 90 80 70 60 50 40 30 20 10 0 -10 -20 -30 -40
f1 (ppm)

23

^{1}H NMR

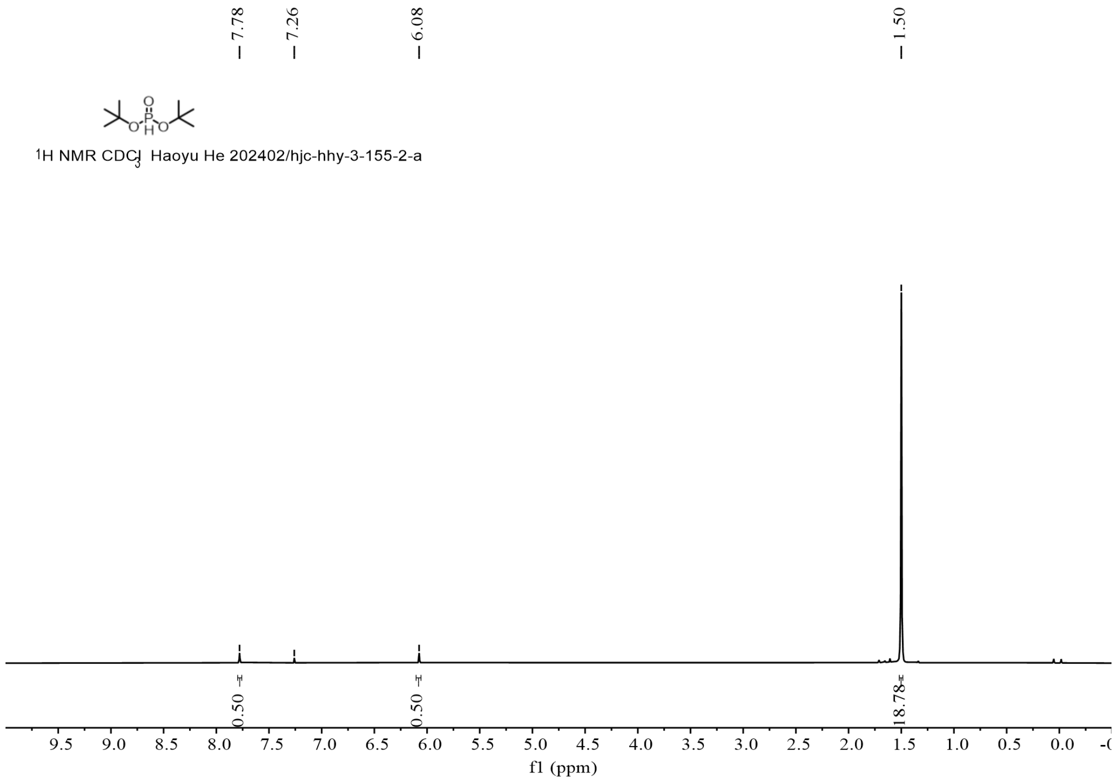

^{13}C NMR

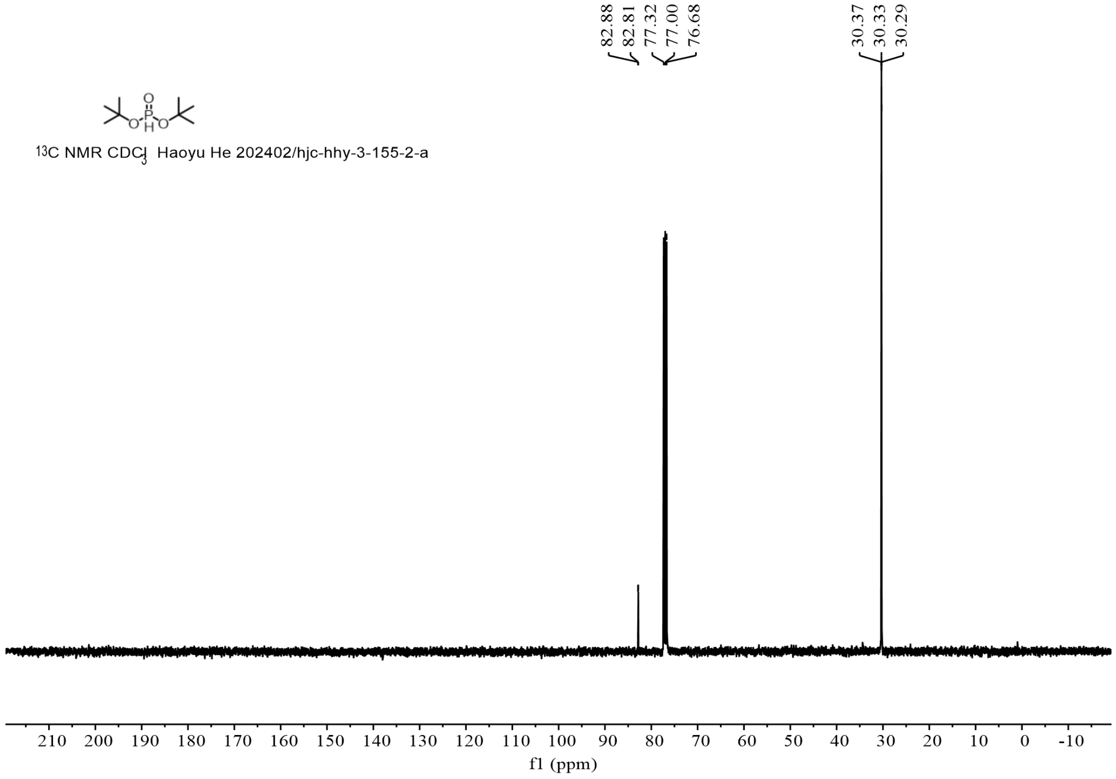

^{31}P NMR

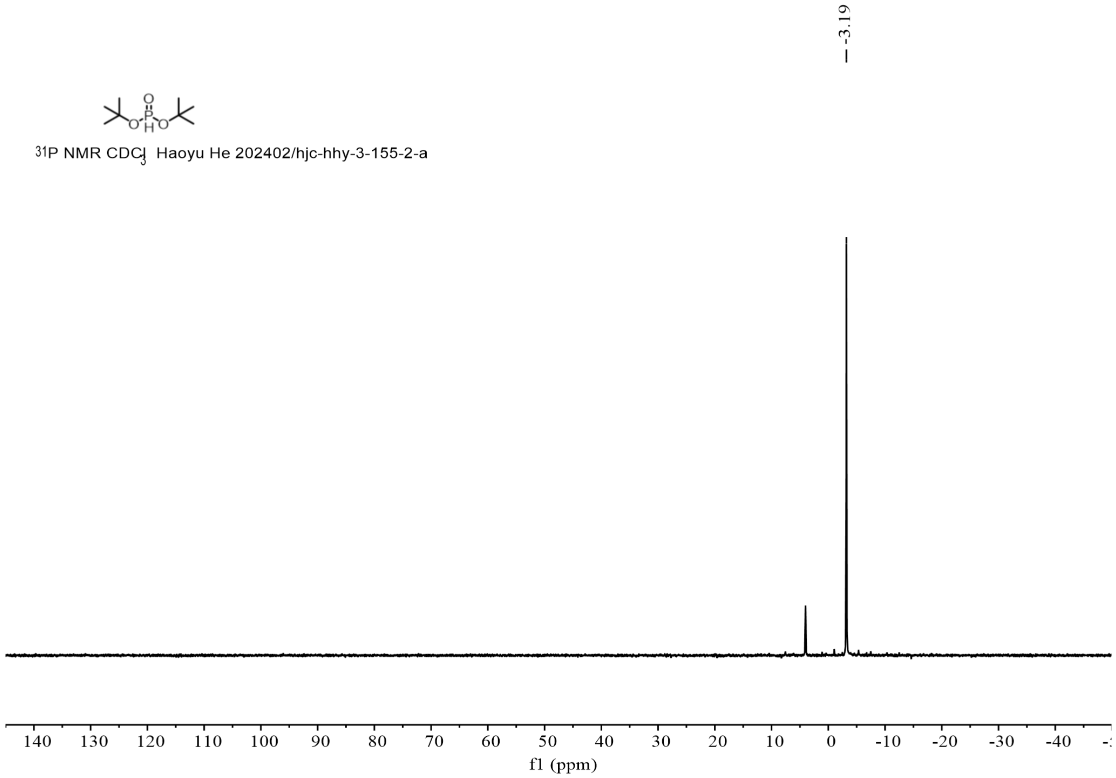

-3.19
31P NMR CDCl3 Haoyu He 202402/hjc-hhy-3-155-2-a
140 130 120 110 100 90 80 70 60 50 40 30 20 10 0 -10 -20 -30 -40
f1 (ppm)

24

^{1}H NMR

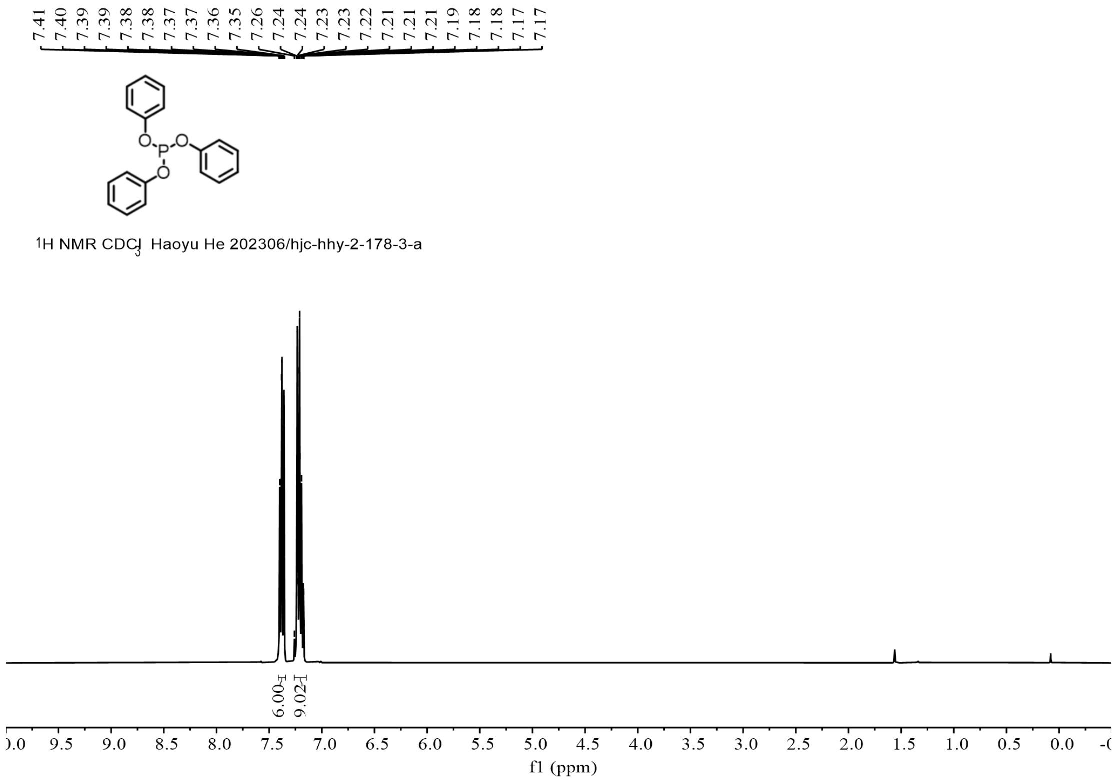

^{13}C NMR

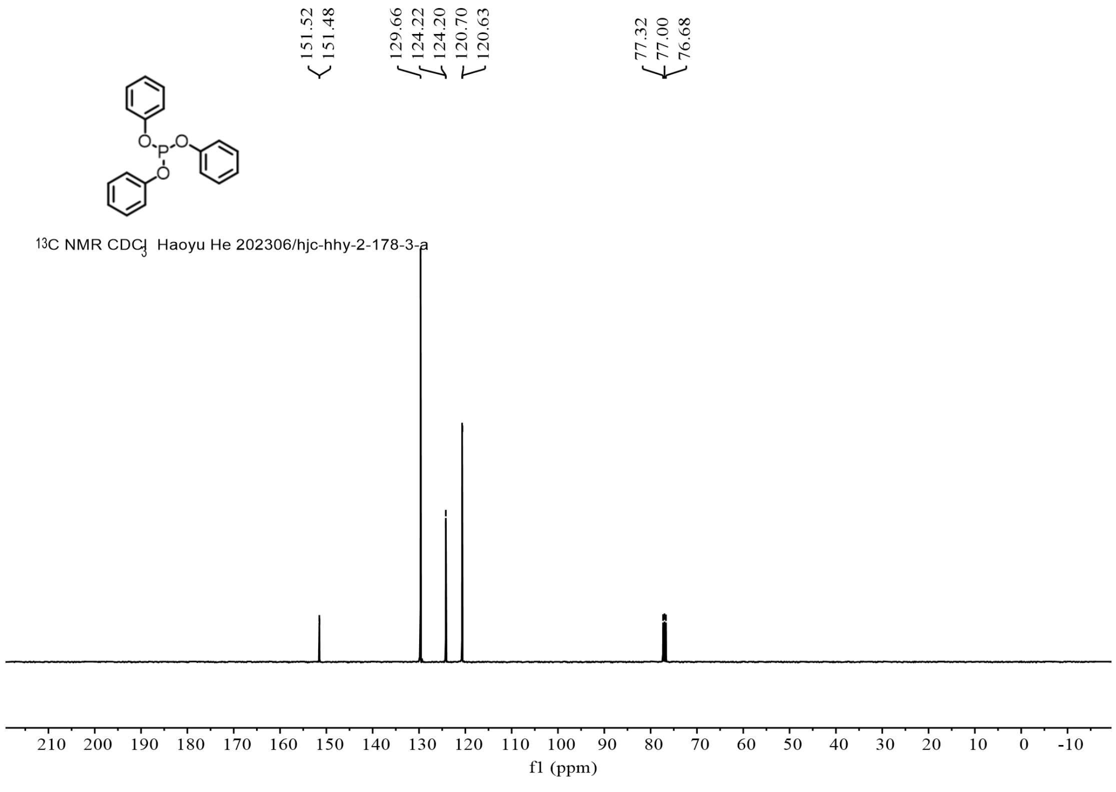

^{31}P NMR

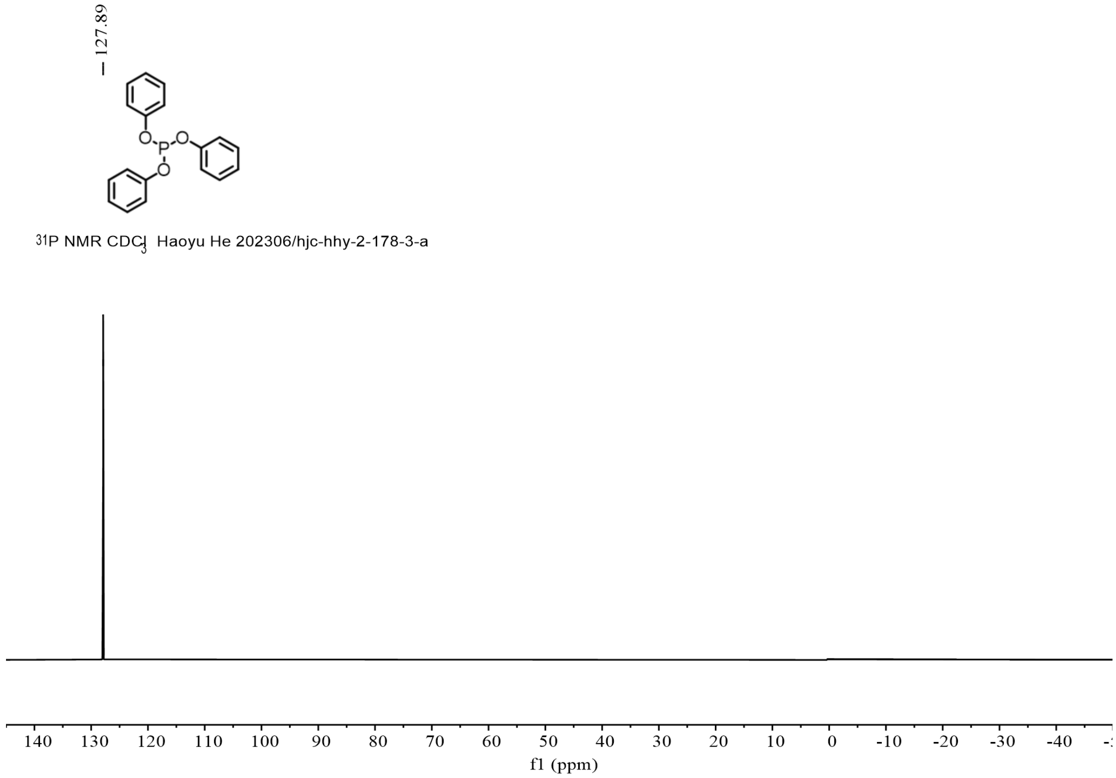
127.89
O
P
O
O
31P NMR CDCl3 Haoyu He 202306/hjc-hhy-2-178-3-a
140
130
120
110
100
90
80
70
60
50
40
30
20
10
0
-10
-20
-30
-40
f1 (ppm)

25

^{1}H NMR

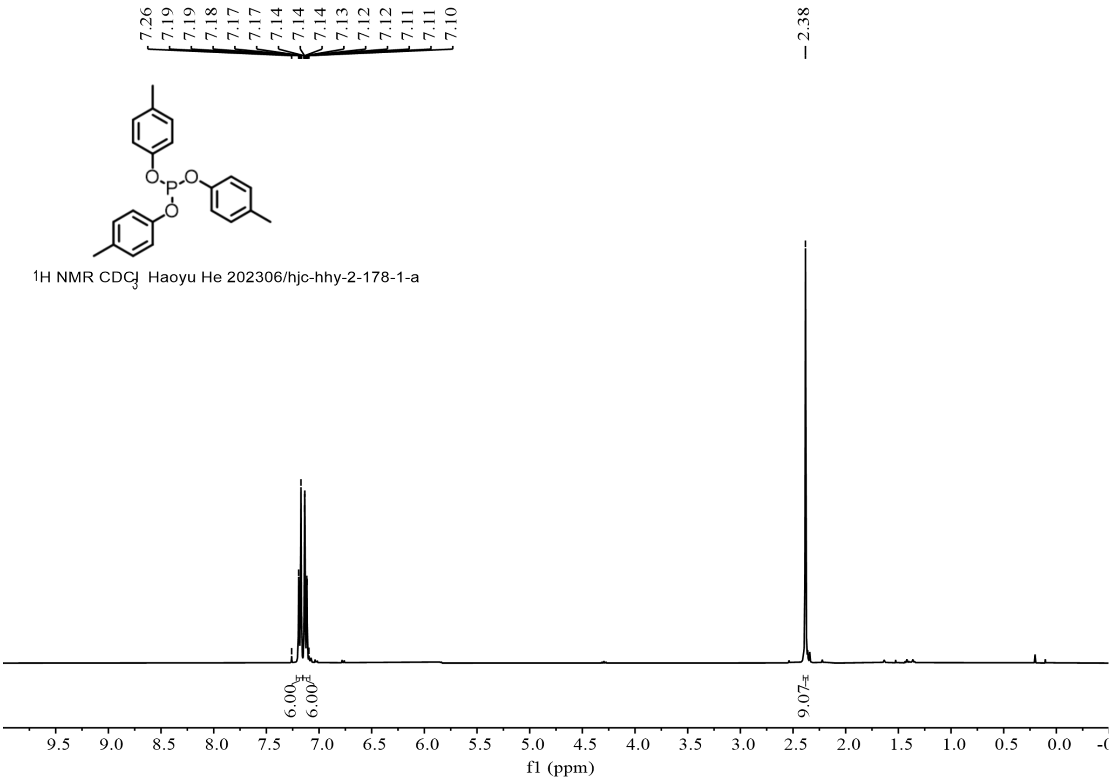

^{13}C NMR

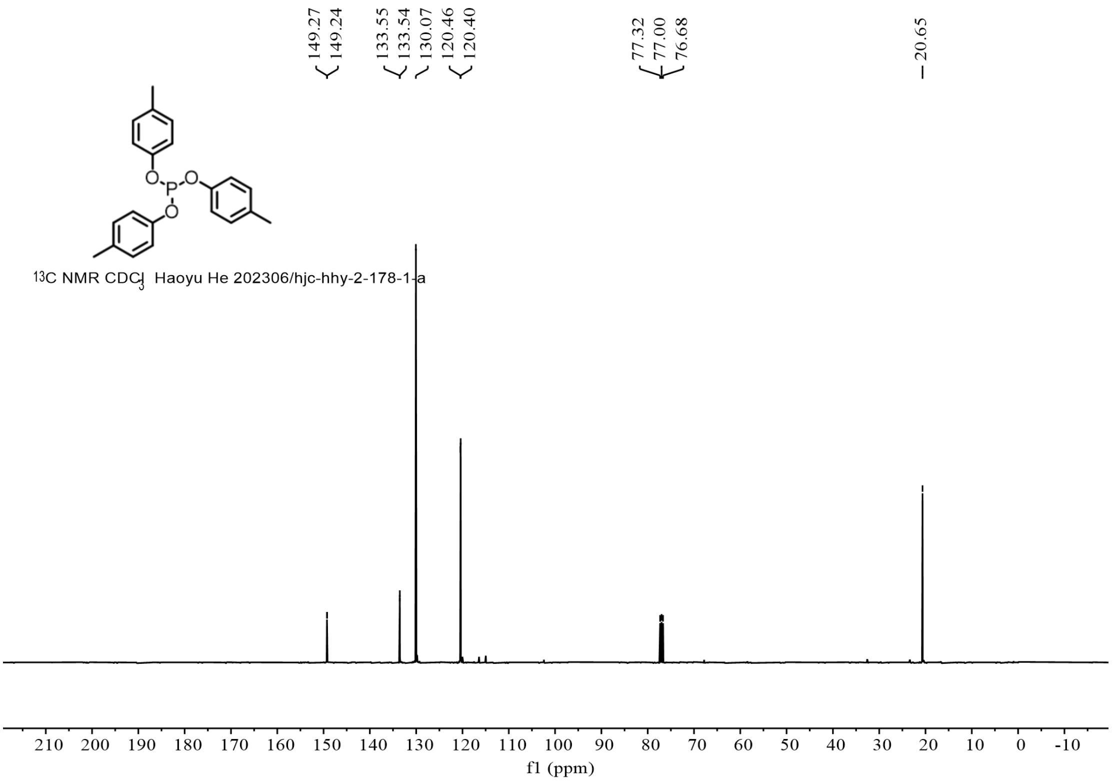

^{31}P NMR

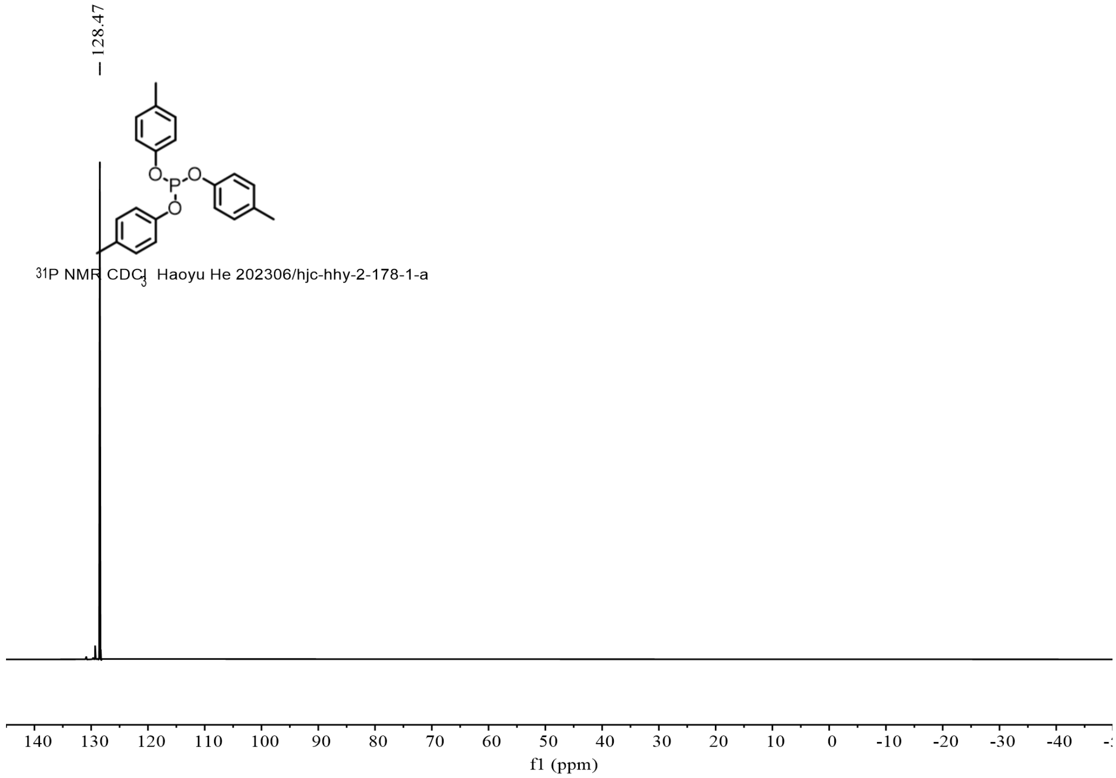

128.47
31P NMR CDCl3 Haoyu He 202306/hjc-hhy-2-178-1-a
140 130 120 110 100 90 80 70 60 50 40 30 20 10 0 -10 -20 -30 -40
f1 (ppm)

26

^{1}H NMR

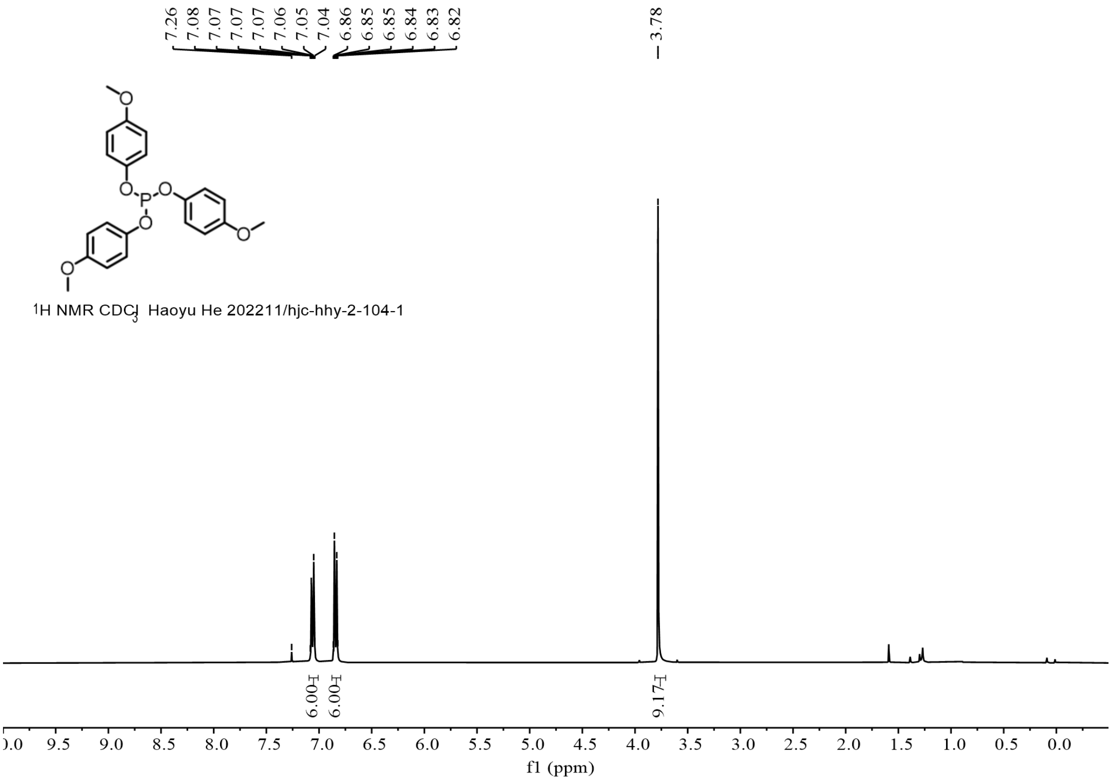

^{13}C NMR

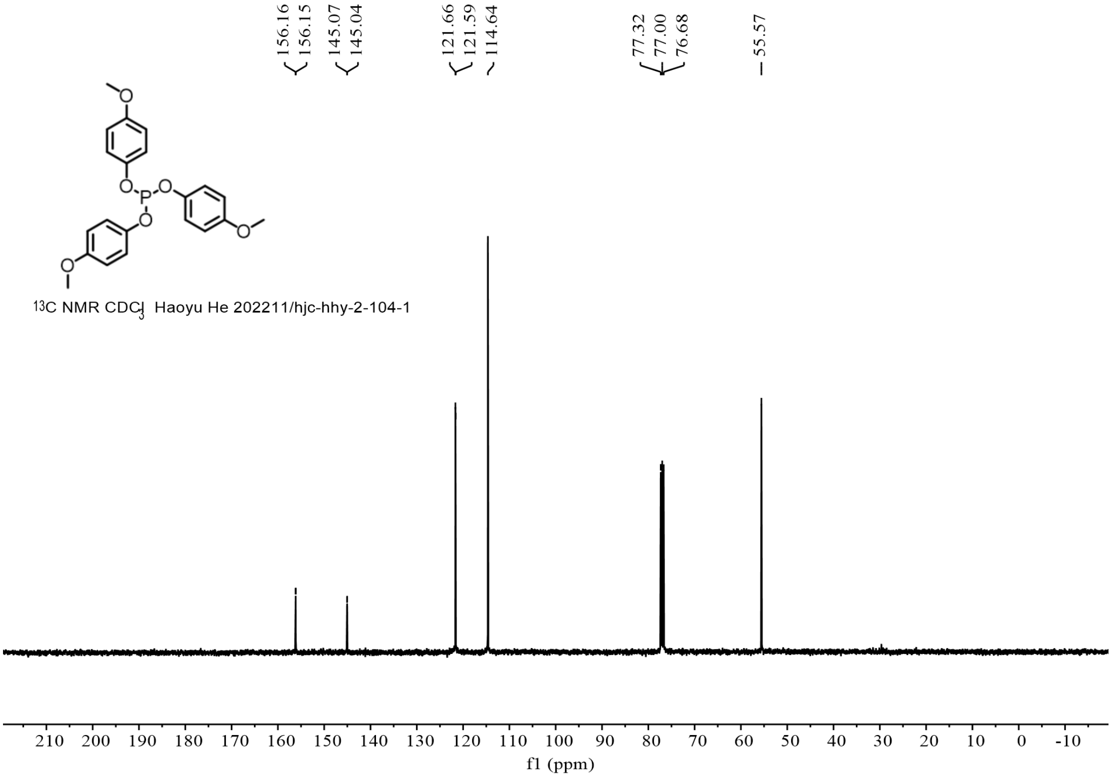

^{31}P NMR

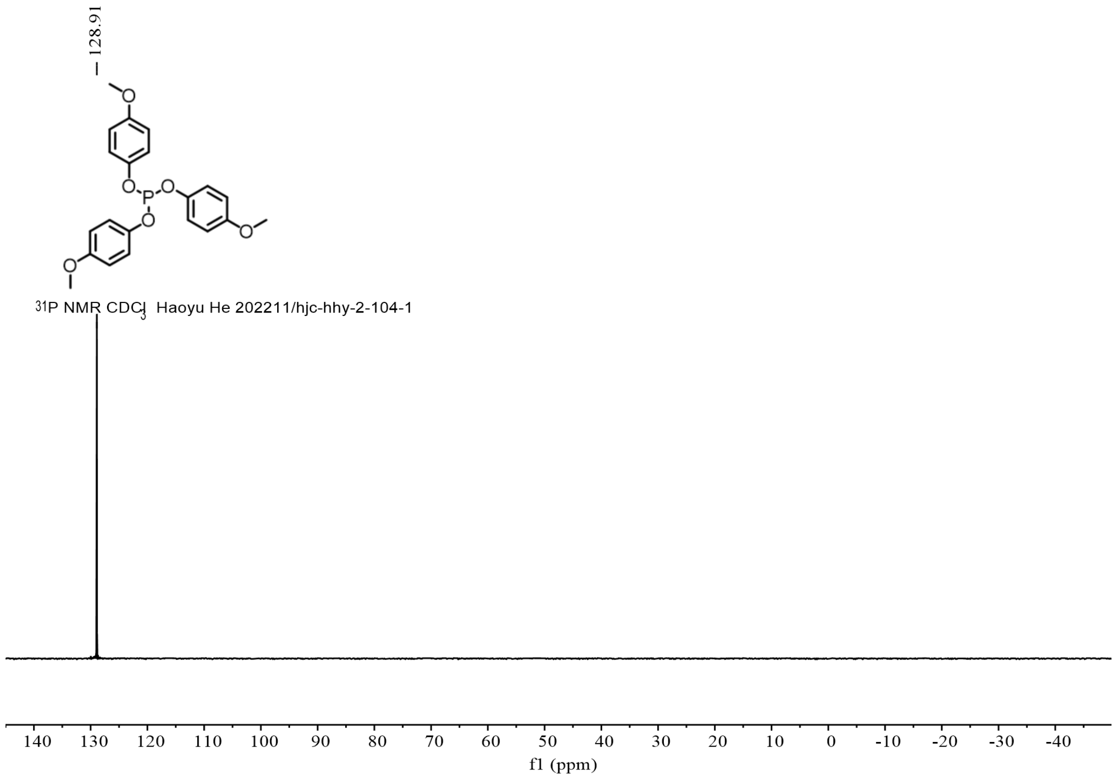

128.91
31P NMR CDCl3 Haoyu He 202211/hjc-hhy-2-104-1
140 130 120 110 100 90 80 70 60 50 40 30 20 10 0 -10 -20 -30 -40
f1 (ppm)

27

^{1}H NMR

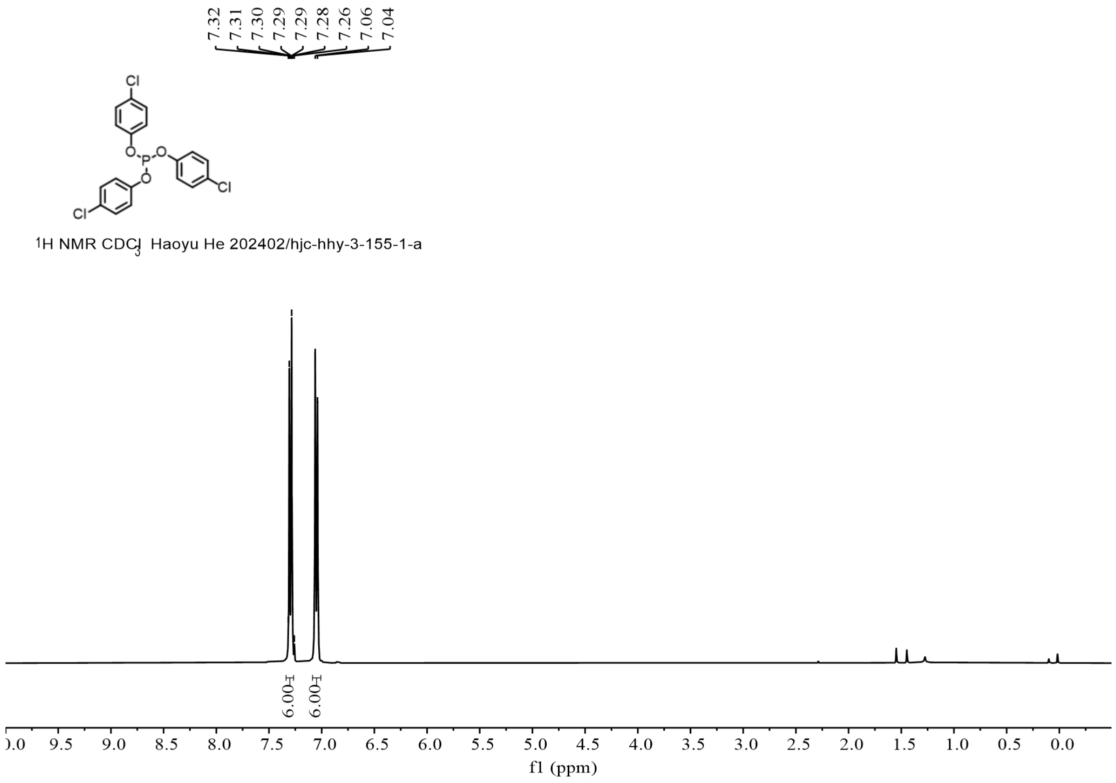

^{13}C NMR

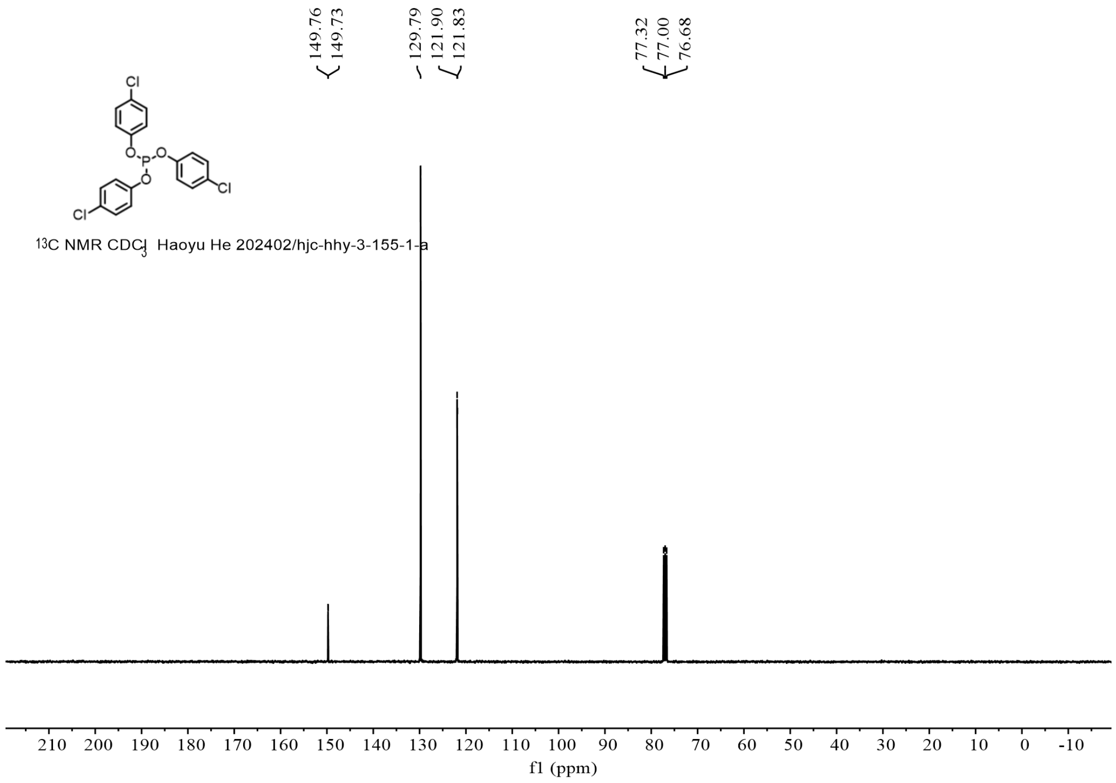

^{31}P NMR

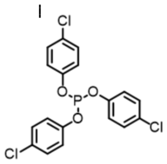

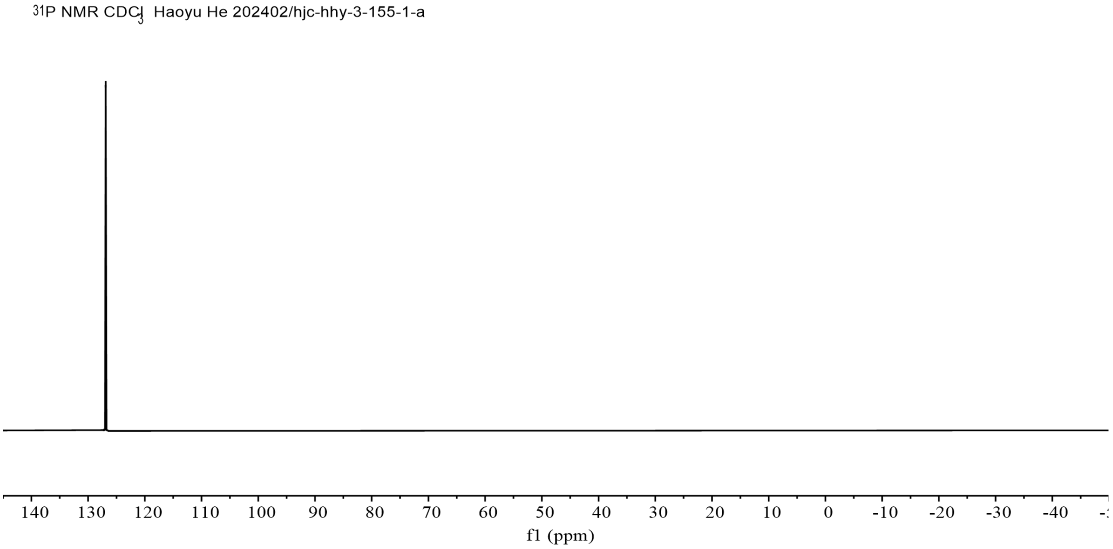
126.89
31P NMR CDCl3 Haoyu He 202402/hjc-hhy-3-155-1-a
140 130 120 110 100 90 80 70 60 50 40 30 20 10 0 -10 -20 -30 -40
f1 (ppm)

28

^{1}H NMR

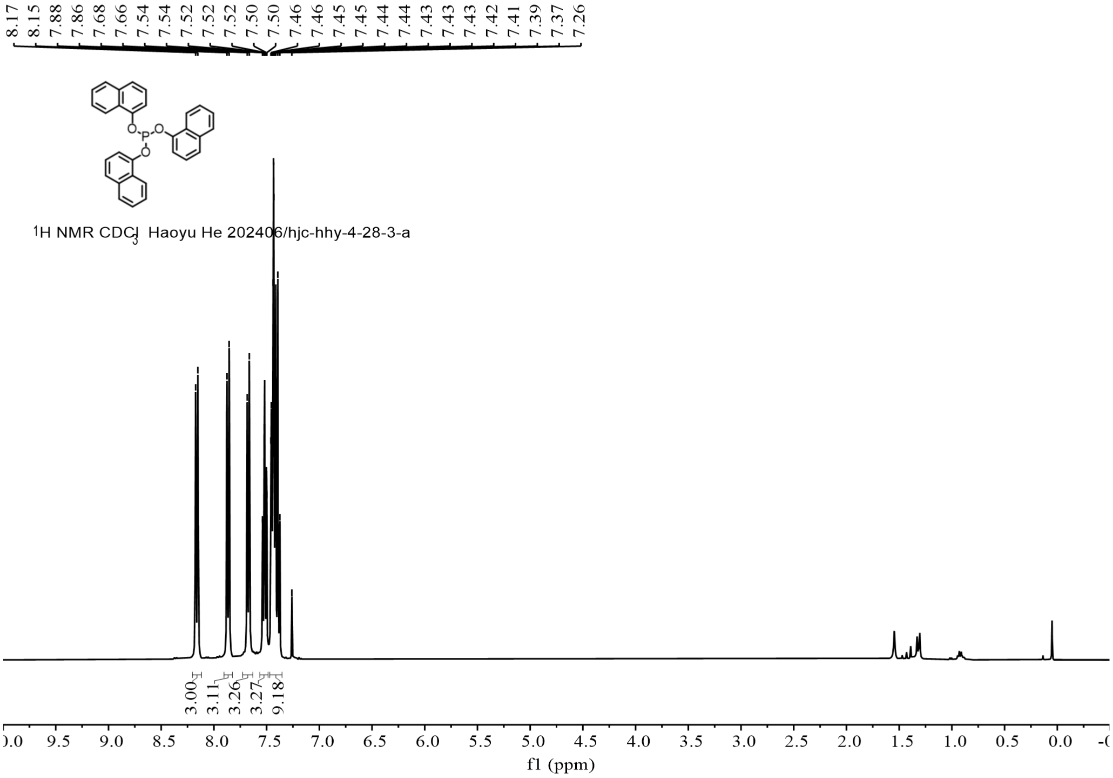

^{13}C NMR

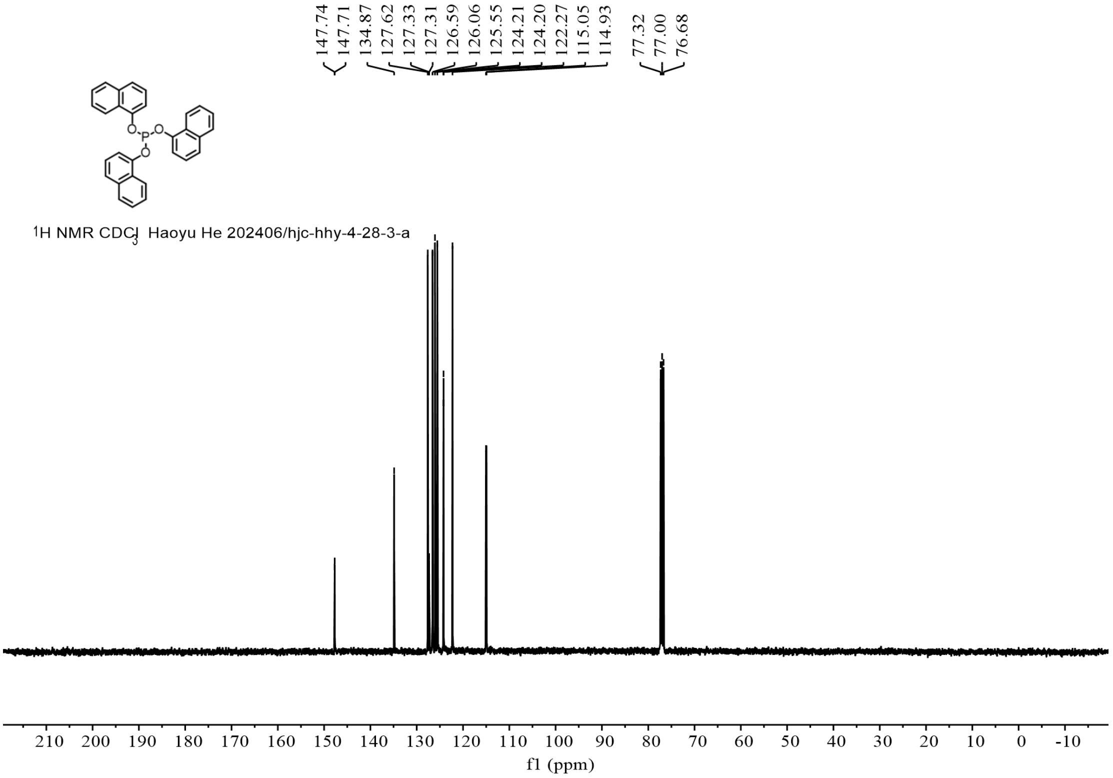

^{31}P NMR

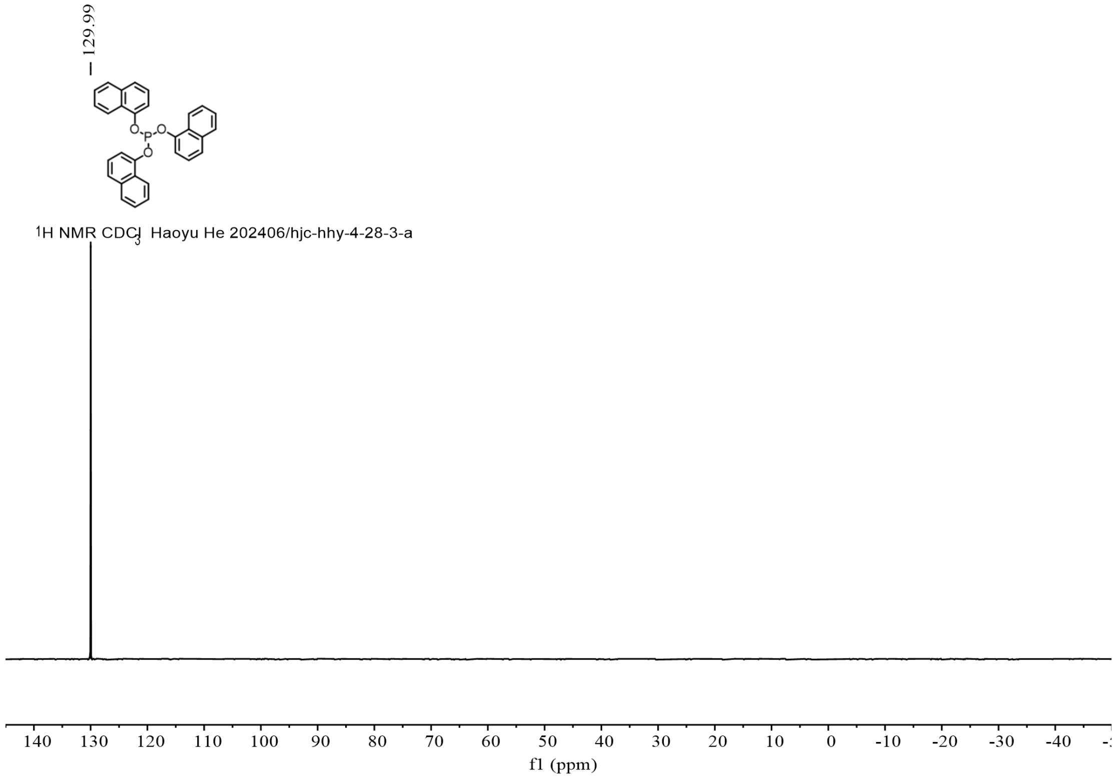

129.99
O
P
O
O
1H NMR CDCl3 Haoyu He 202406/hjc-hhy-4-28-3-a
140 130 120 110 100 90 80 70 60 50 40 30 20 10 0 -10 -20 -30 -40
f1 (ppm)

29

^{1}H NMR

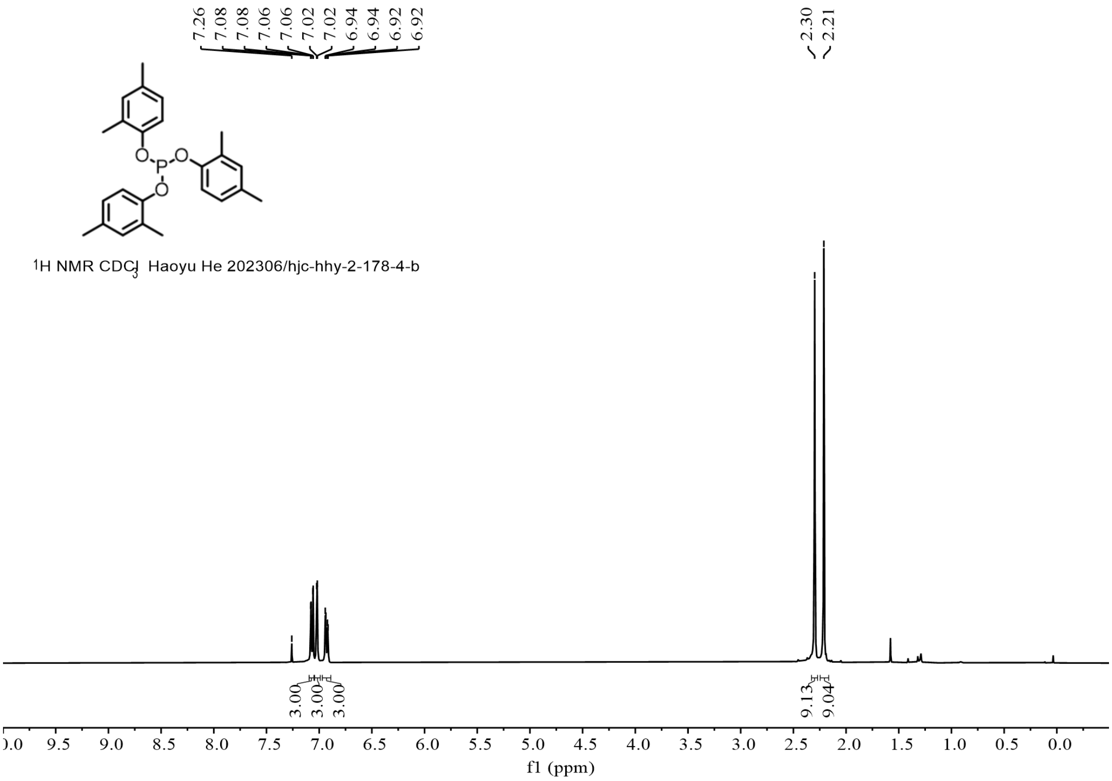

^{13}C NMR

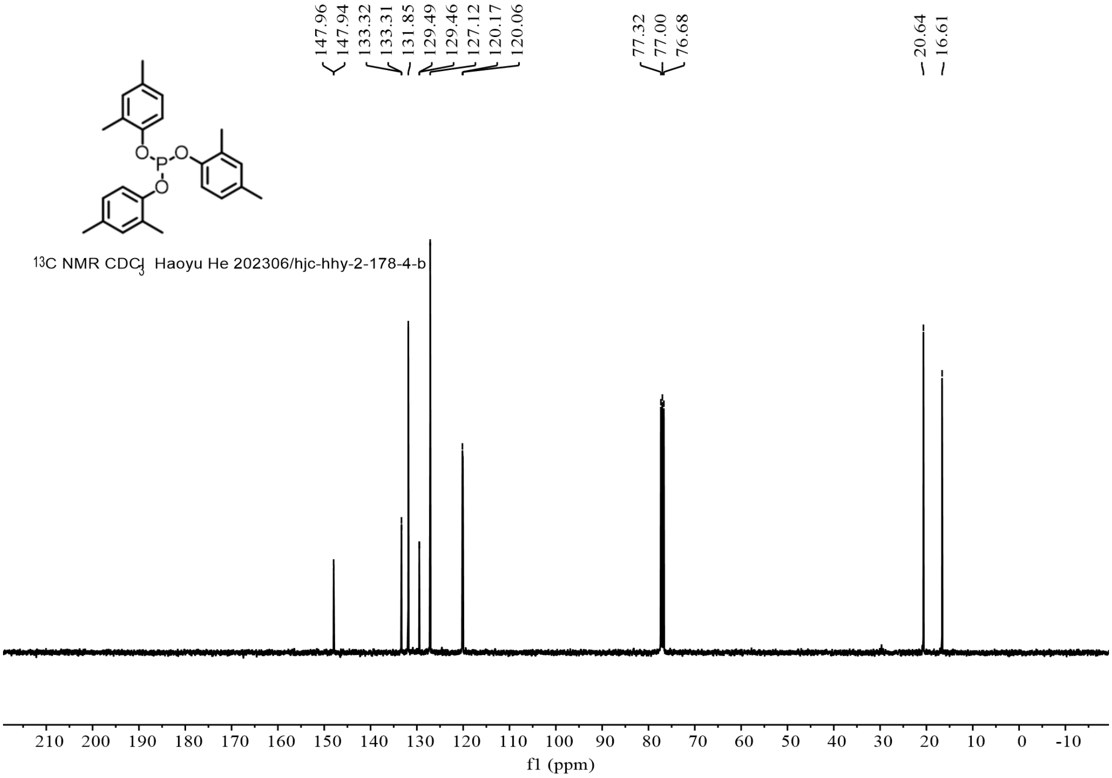

^{31}P NMR

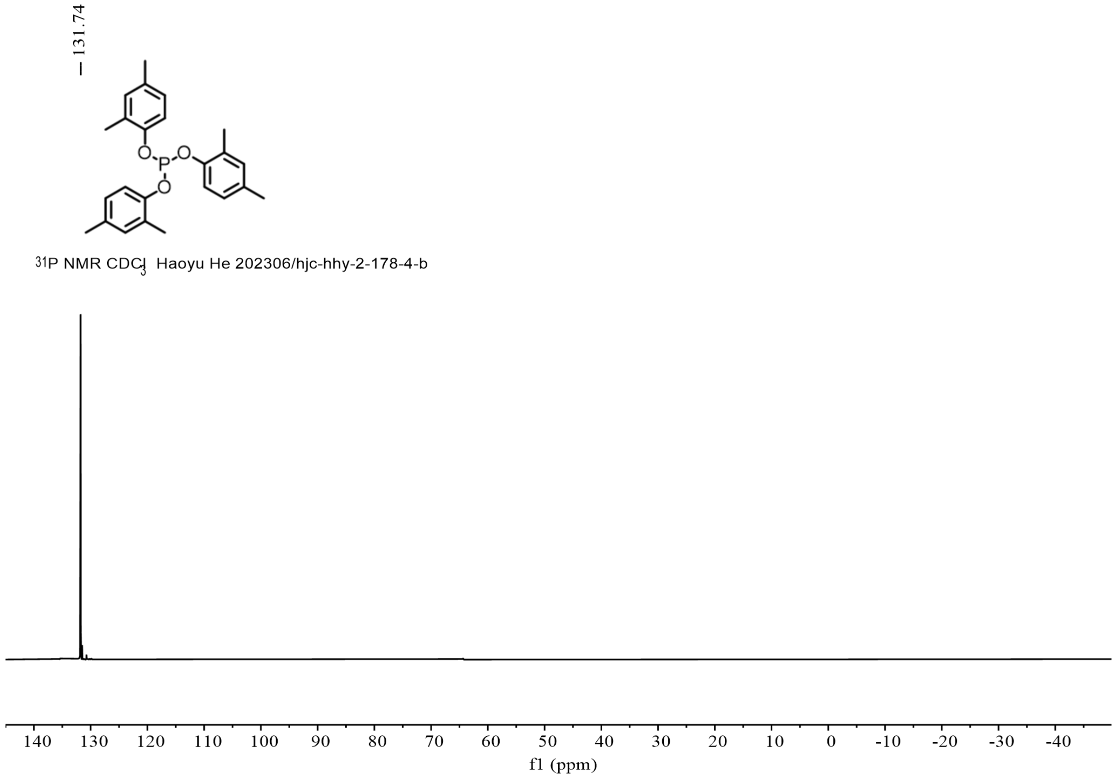

131.74
31P NMR CDCl3 Haoyu He 202306/hjc-hhy-2-178-4-b
140 130 120 110 100 90 80 70 60 50 40 30 20 10 0 -10 -20 -30 -40
f1 (ppm)

30

^{1}H NMR

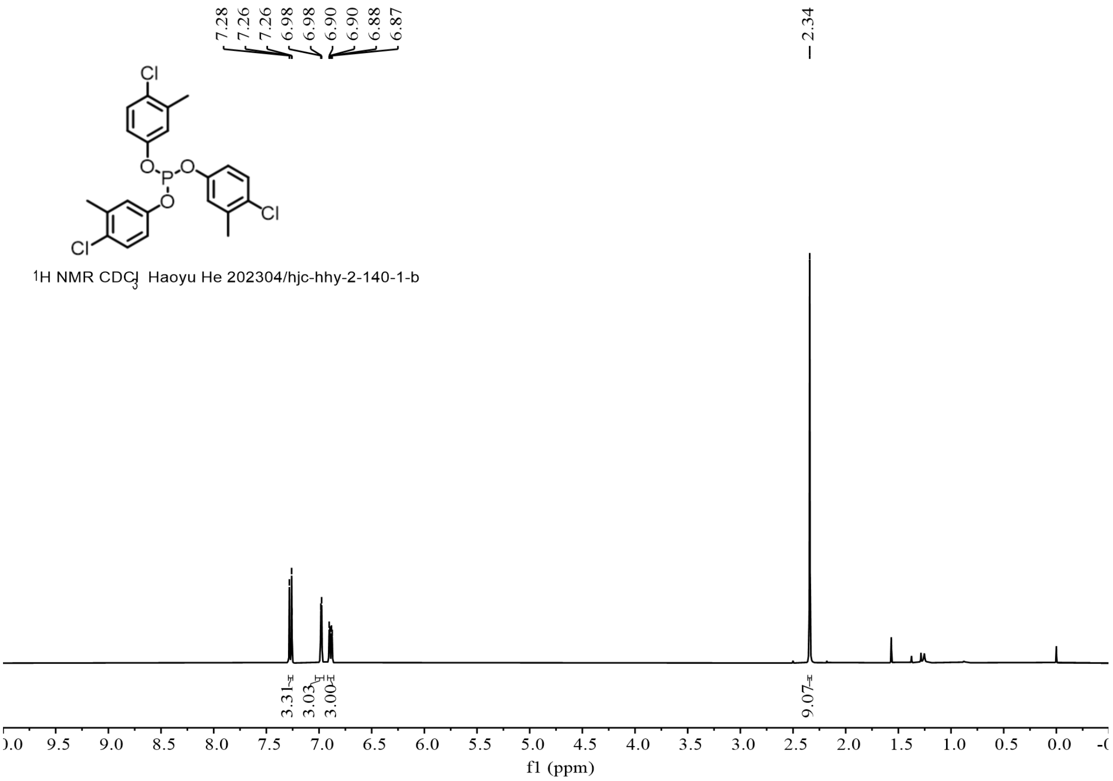

^{13}C NMR

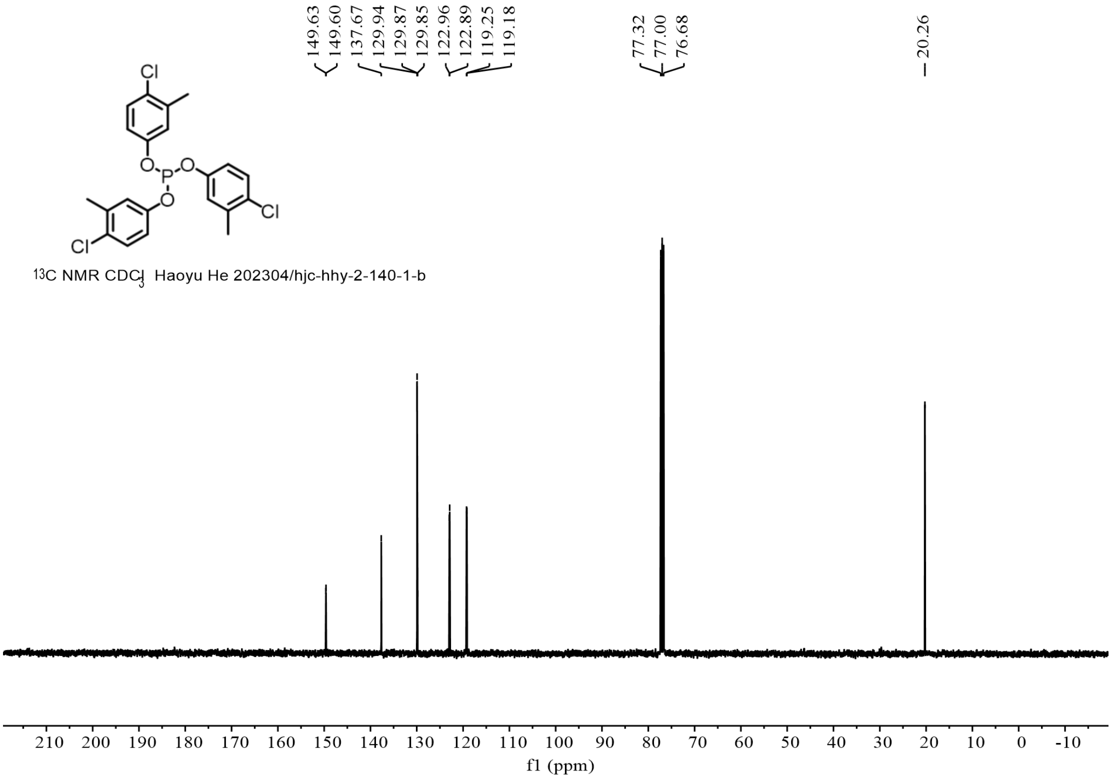

^{31}P NMR

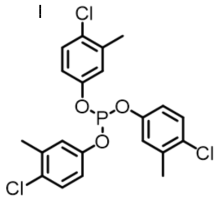

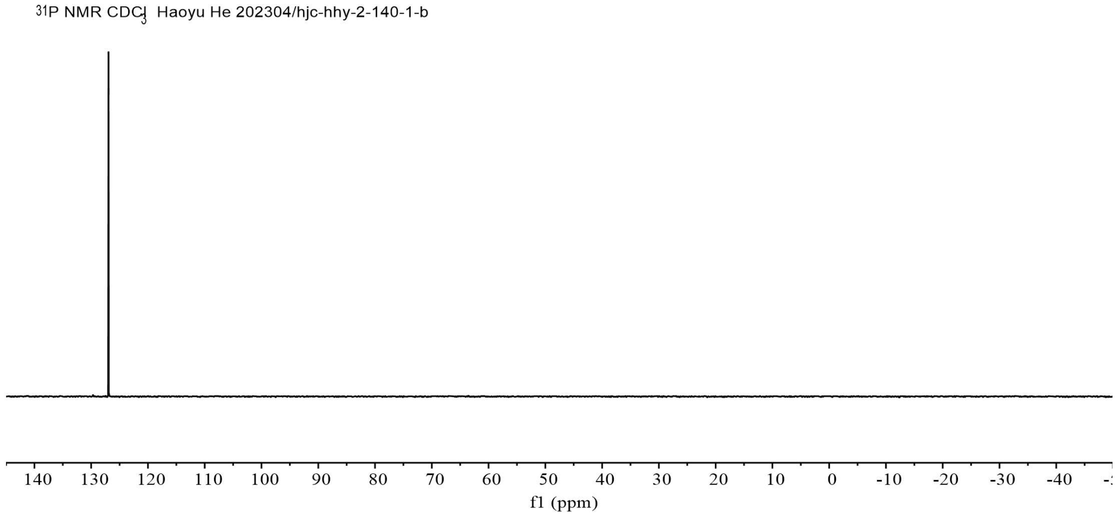

126.95
31P NMR CDCl3 Haoyu He 202304/hjc-hhy-2-140-1-b
140 130 120 110 100 90 80 70 60 50 40 30 20 10 0 -10 -20 -30 -40
f1 (ppm)

31

^{1}H NMR

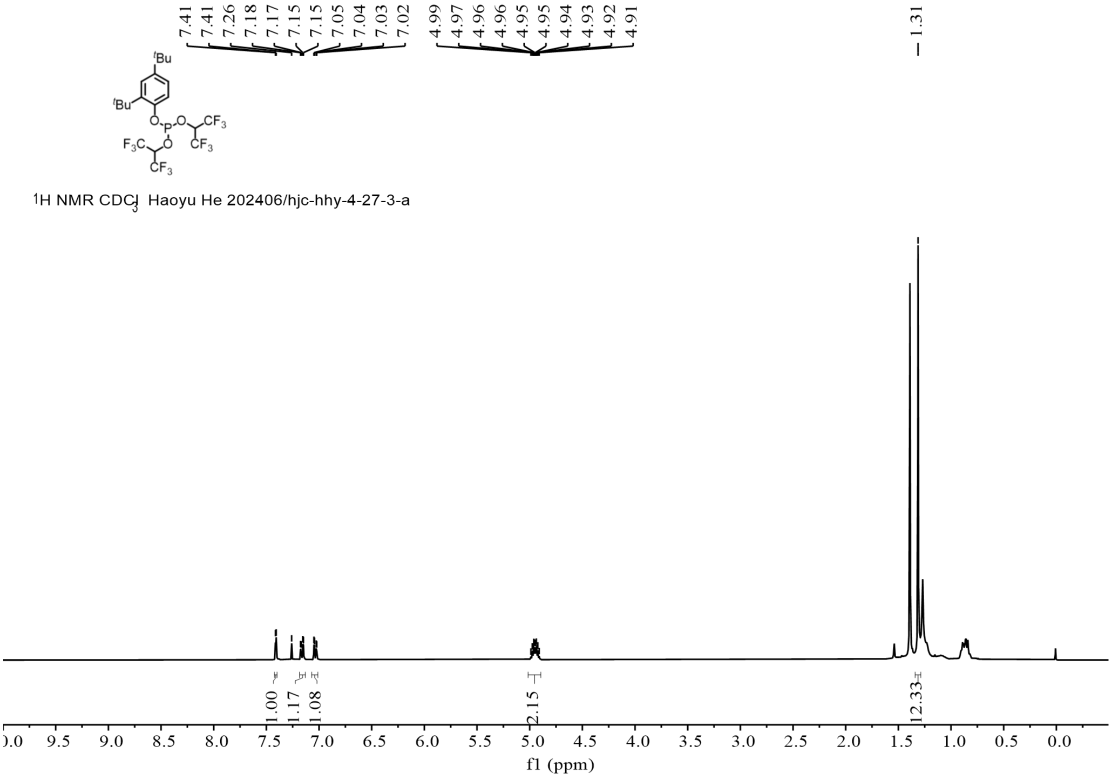

^{13}C NMR

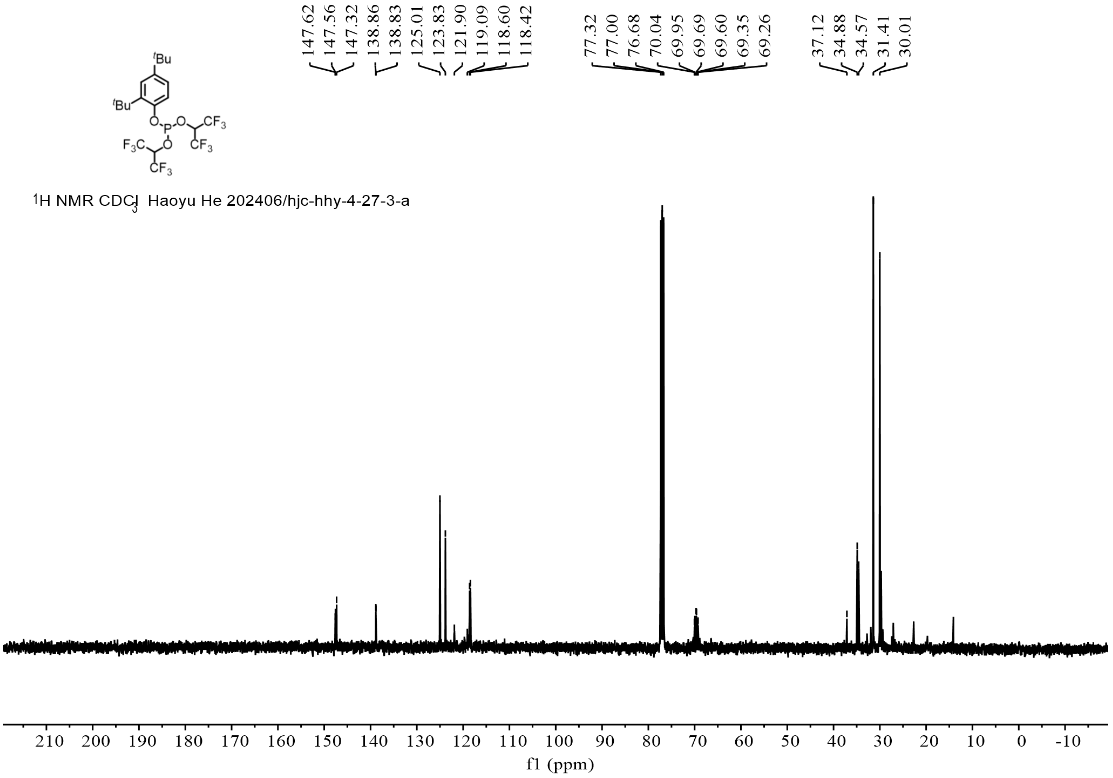

^{31}P NMR

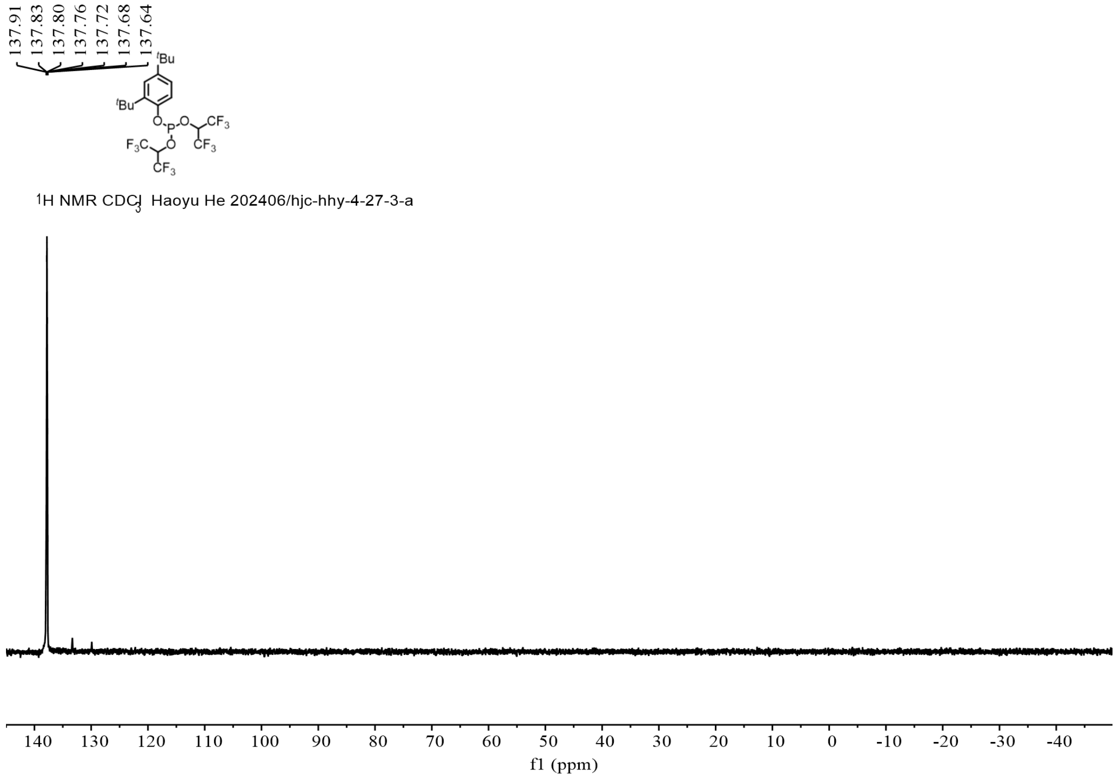
137.91
137.83
137.80
137.76
137.72
137.68
137.64
tBu
tBu
O
P
O
CF3
CF3
F3C
O
CF3
1H NMR CDCl3 Haoyu He 202406/hjc-hhy-4-27-3-a
140
130
120
110
100
90
80
70
60
50
40
30
20
10
0
-10
-20
-30
-40
f1 (ppm)

32

^{1}H NMR

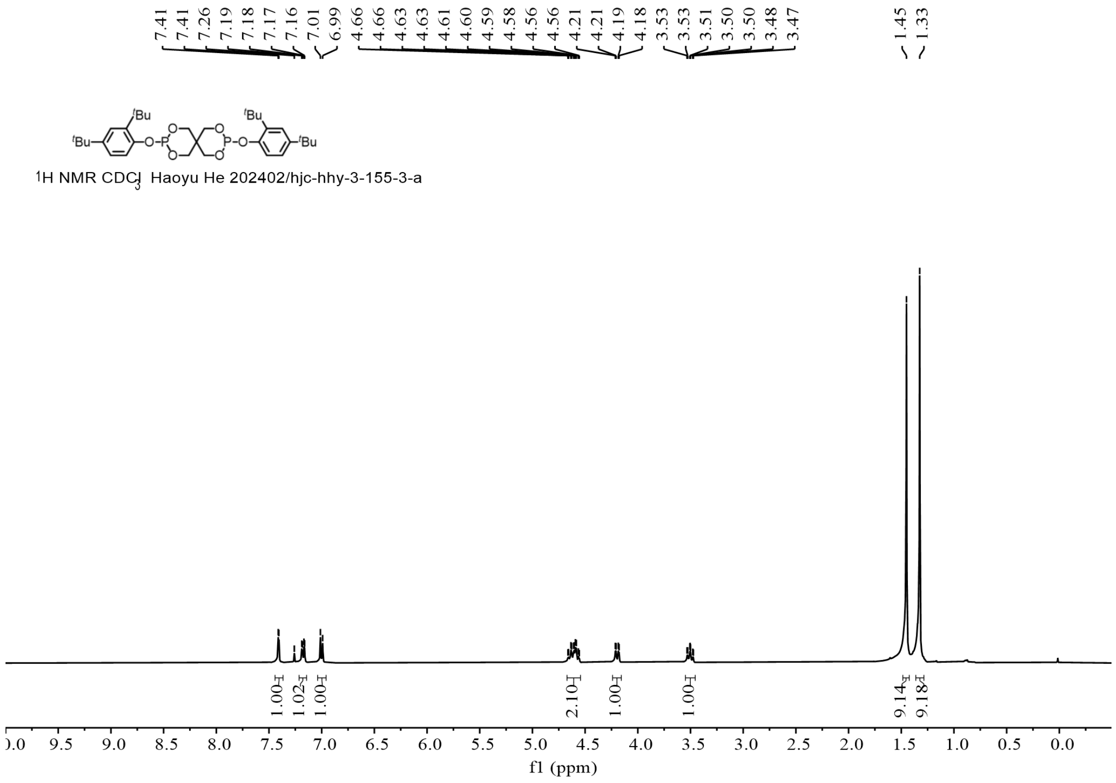

^{13}C NMR

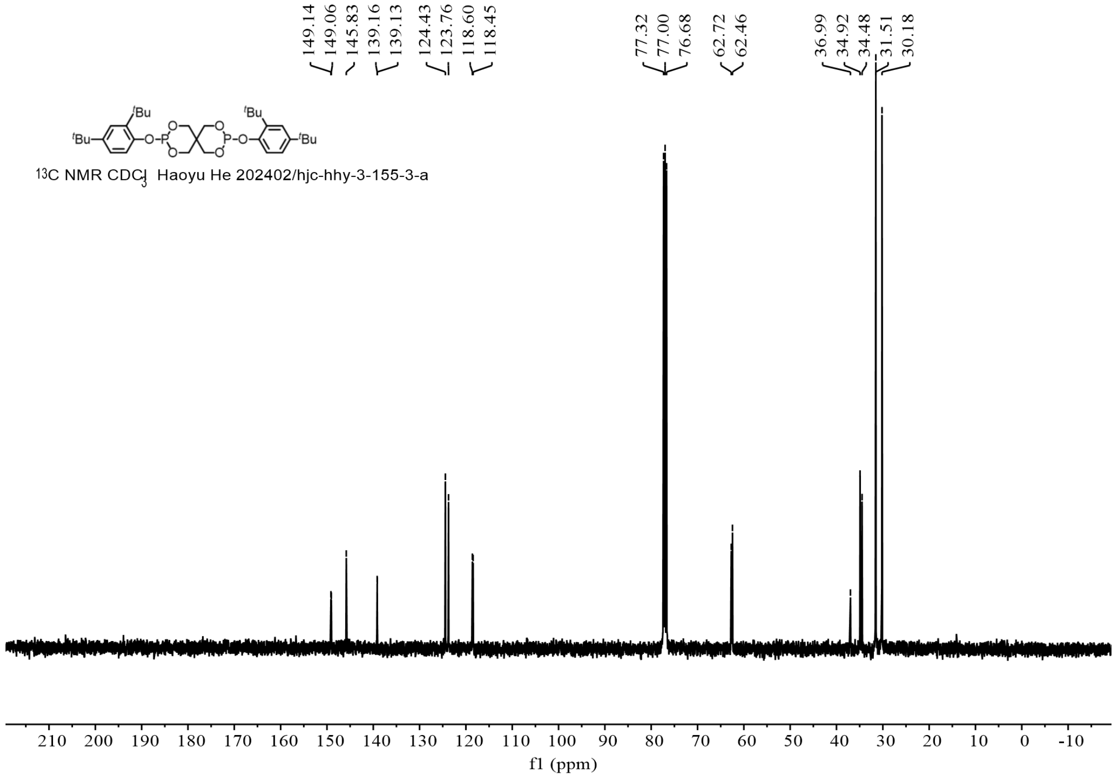

31P NMR

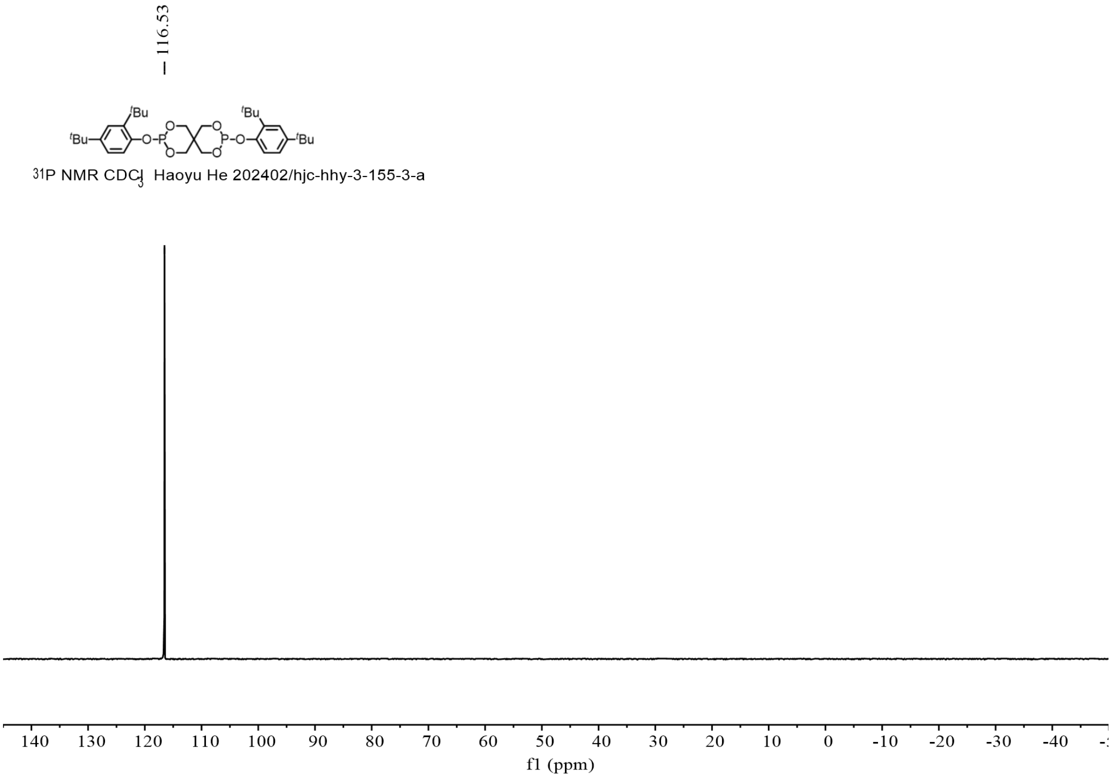

116.53
tBu
tBu
tBu
tBu
31P NMR CDCl3 Haoyu He 202402/hjc-hhy-3-155-3-a
140 130 120 110 100 90 80 70 60 50 40 30 20 10 0 -10 -20 -30 -40
f1 (ppm)

33

^{1}H NMR

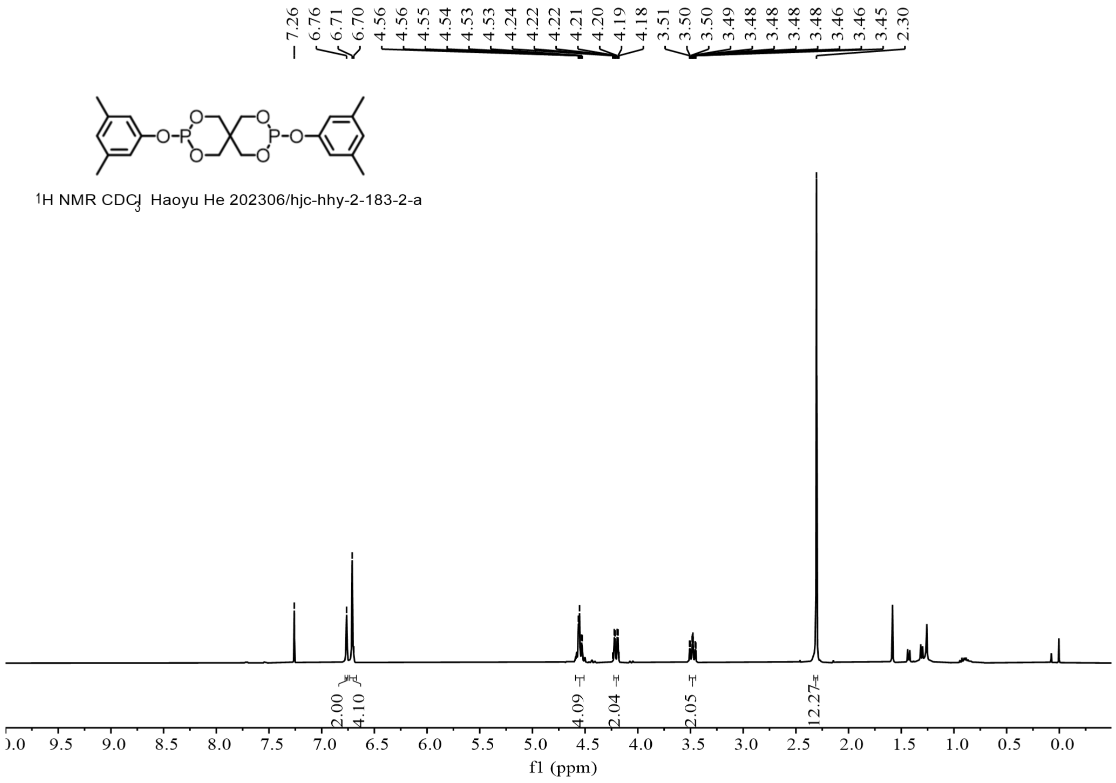

^{13}C NMR

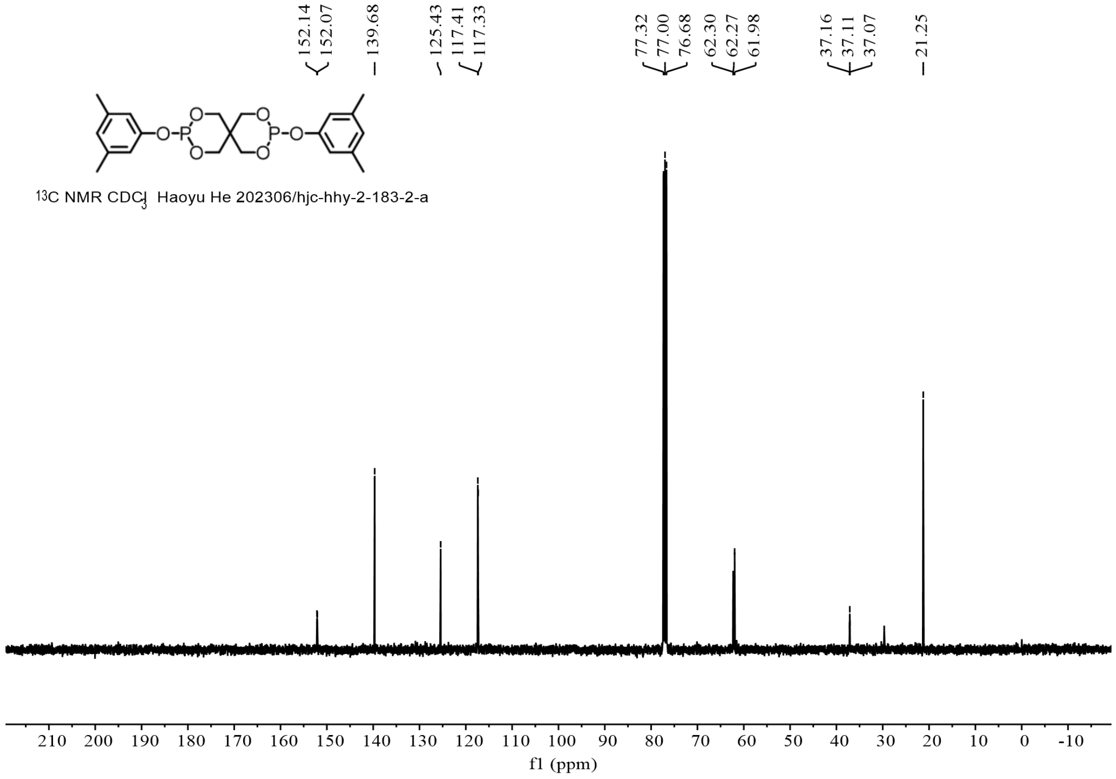

^{31}P NMR

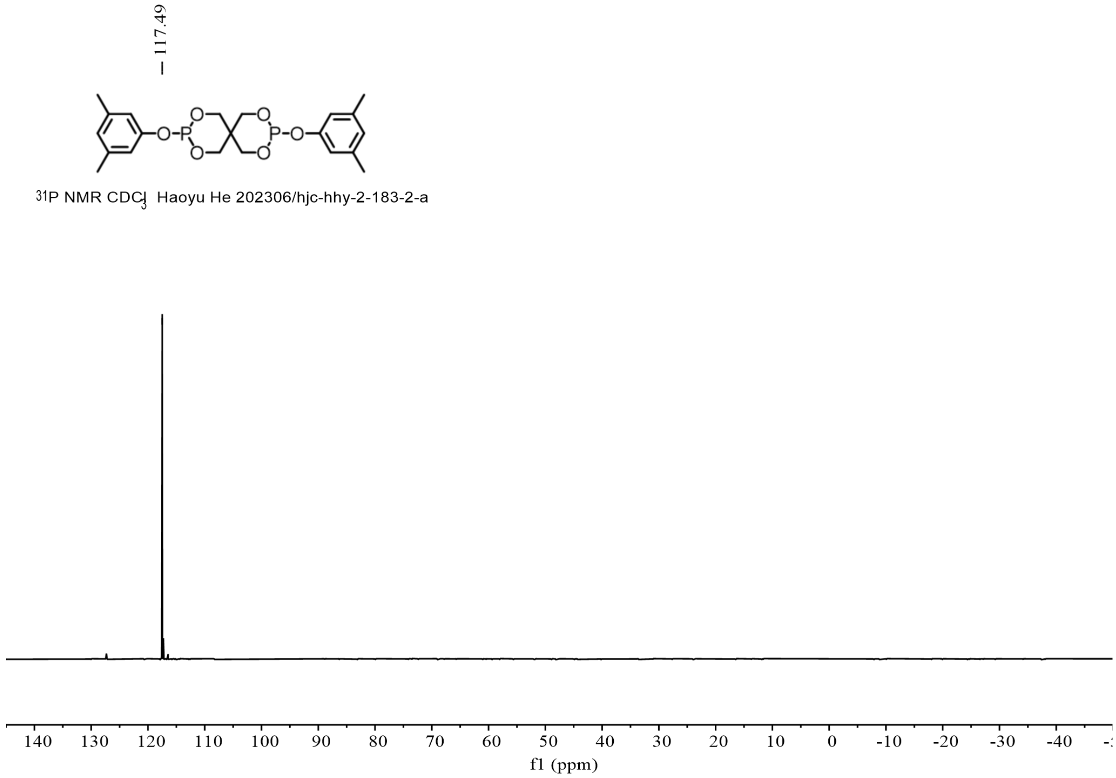

117.49
31P NMR CDCl3 Haoyu He 202306/hjc-hhy-2-183-2-a
140
130
120
110
100
90
80
70
60
50
40
30
20
10
0
-10
-20
-30
-40
f1 (ppm)

34

^{1}H NMR

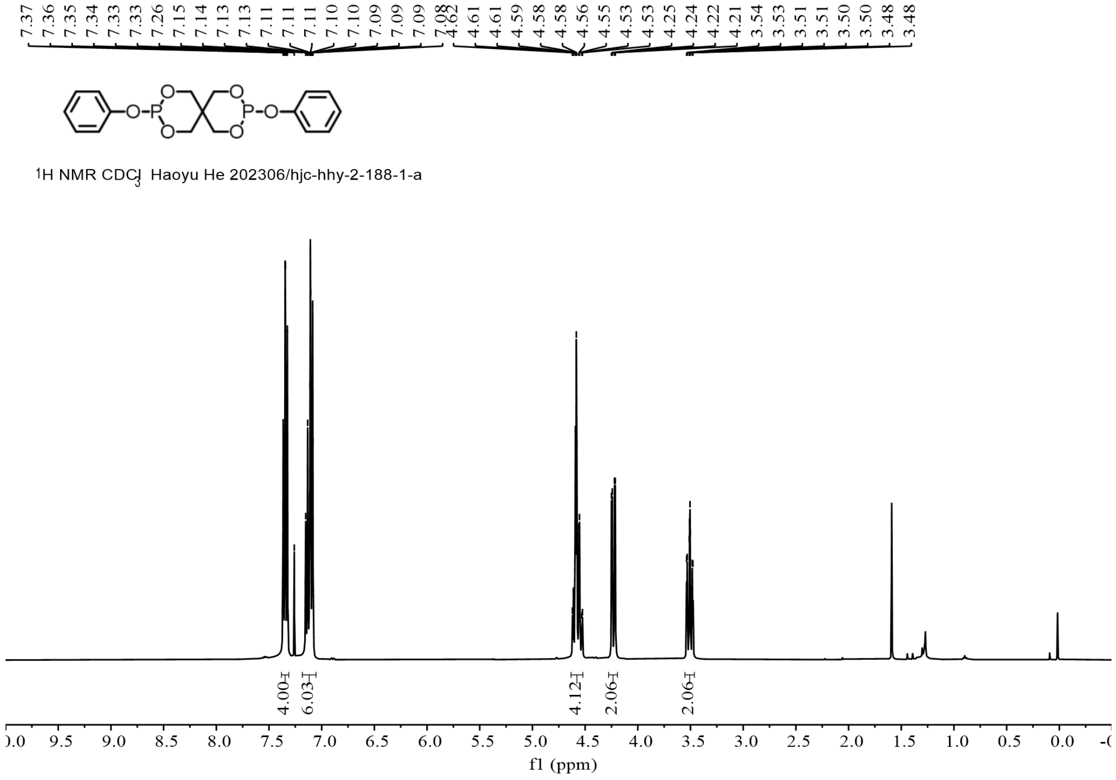

^{13}C NMR

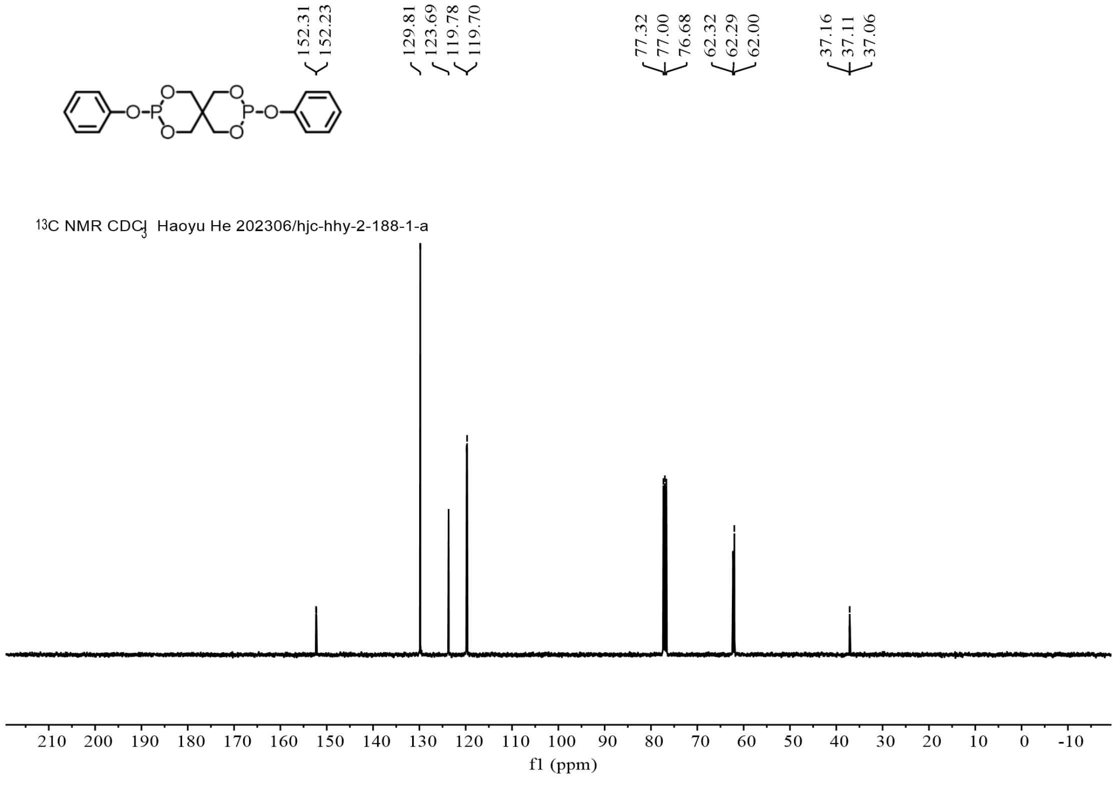

^{31}P NMR

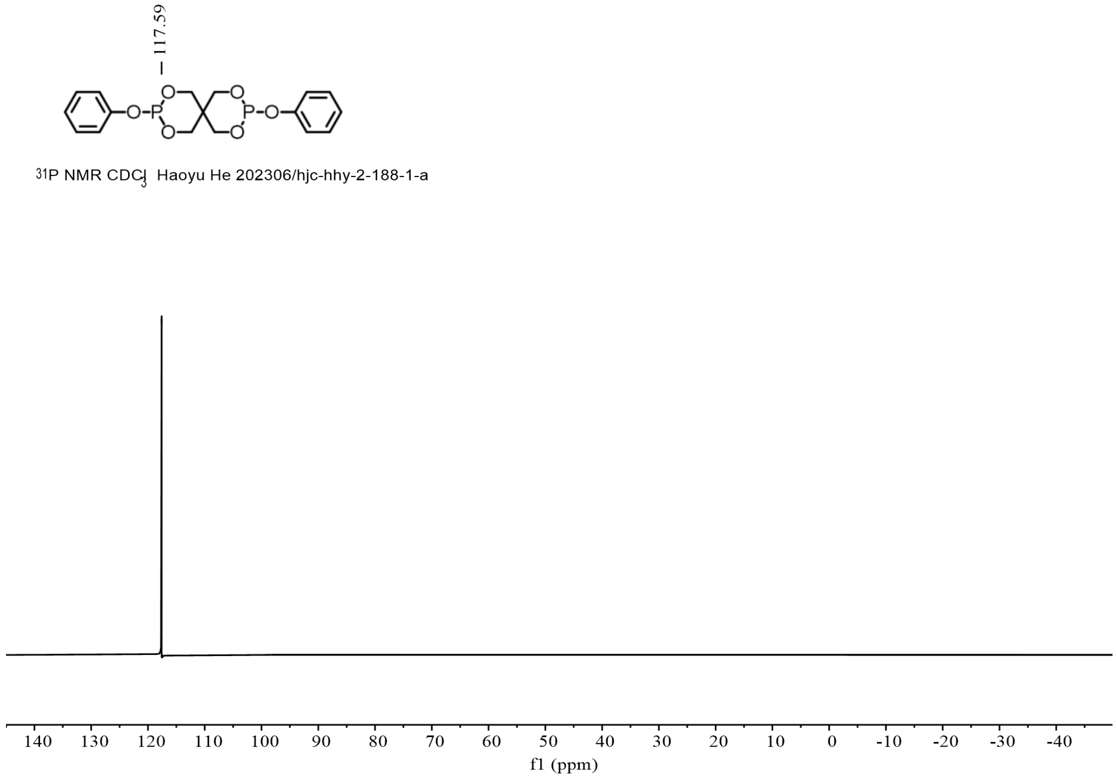

117.59
31P NMR CDCl3 Haoyu He 202306/hjc-hhy-2-188-1-a
140 130 120 110 100 90 80 70 60 50 40 30 20 10 0 -10 -20 -30 -40
f1 (ppm)

35

^{1}H NMR

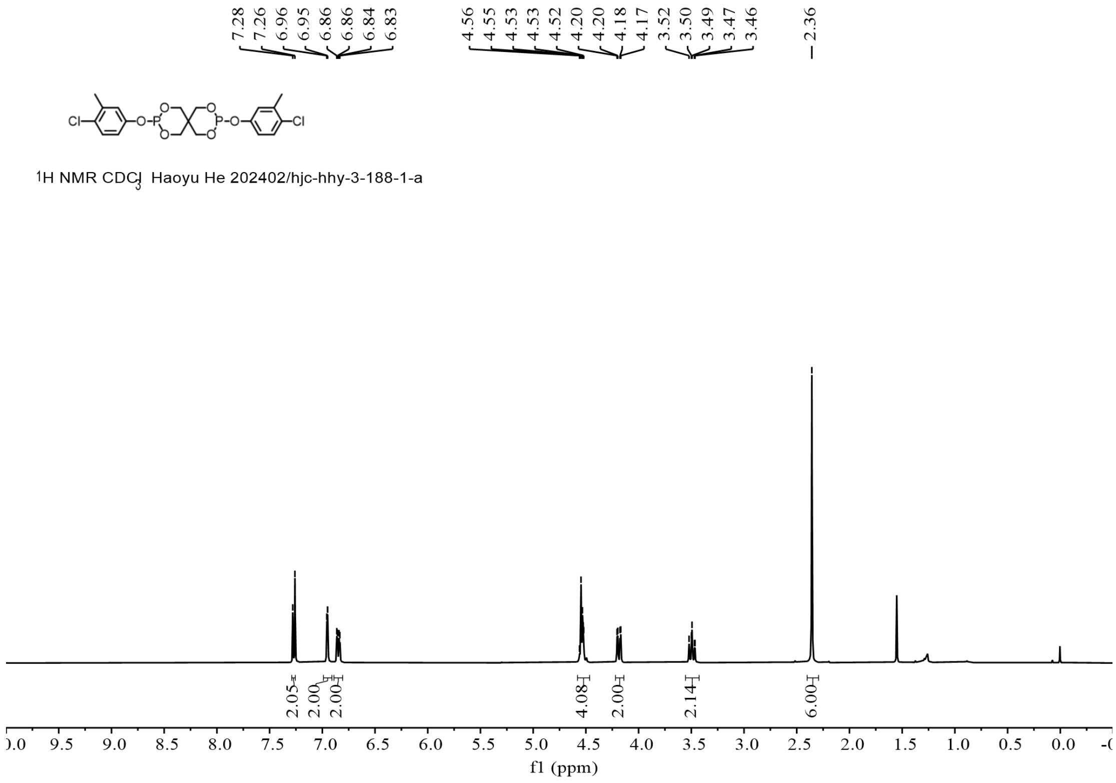

^{13}C NMR

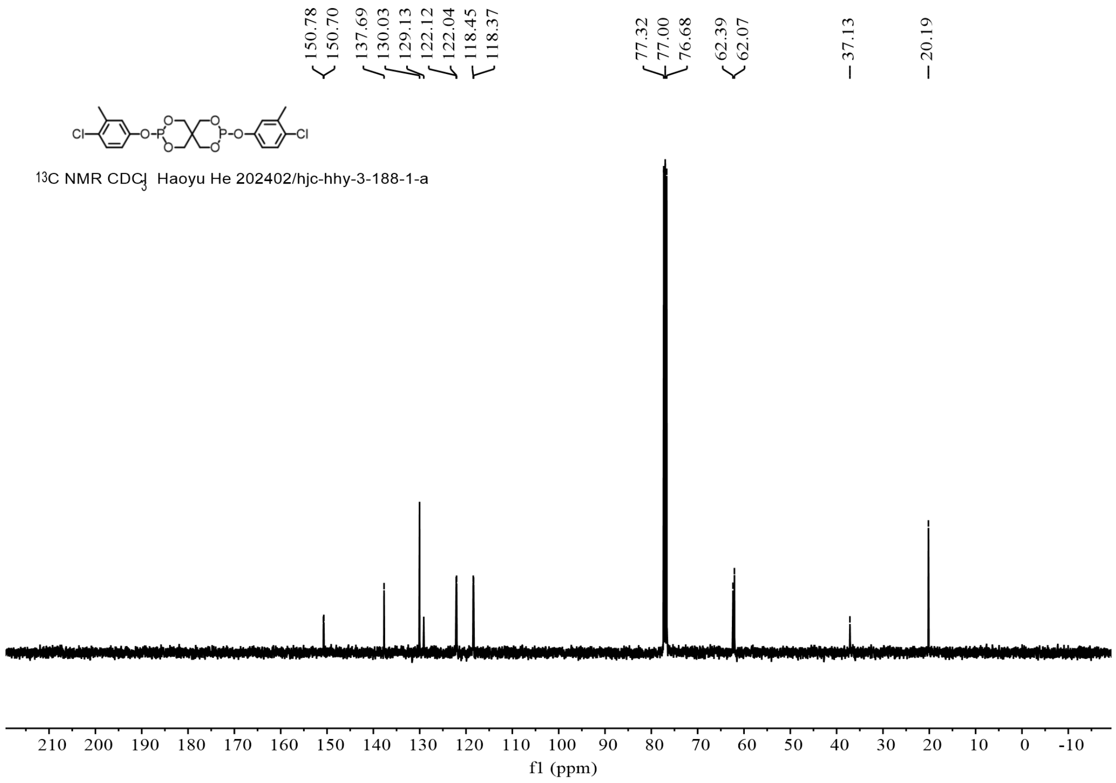

^{31}P NMR

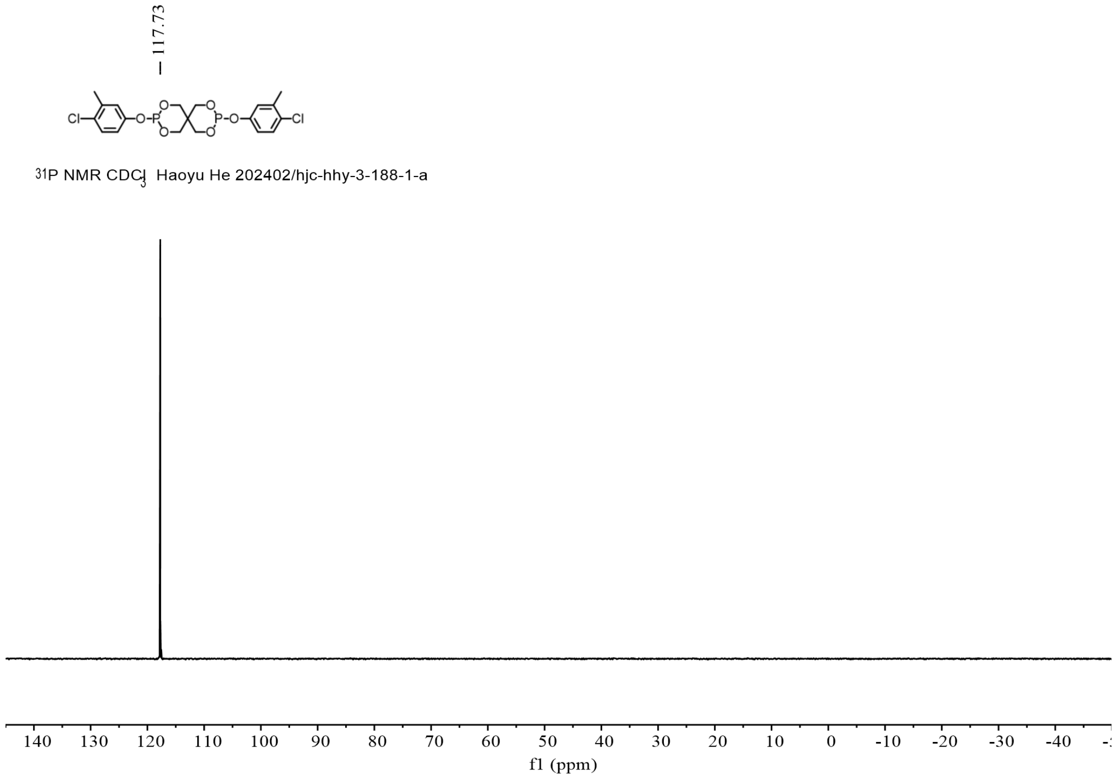
117.73
Cl
Cl
31P NMR CDCl3 Haoyu He 202402/hjc-hhy-3-188-1-a
140
130
120
110
100
90
80
70
60
50
40
30
20
10
0
-10
-20
-30
-40
f1 (ppm)

36

^{1}H NMR

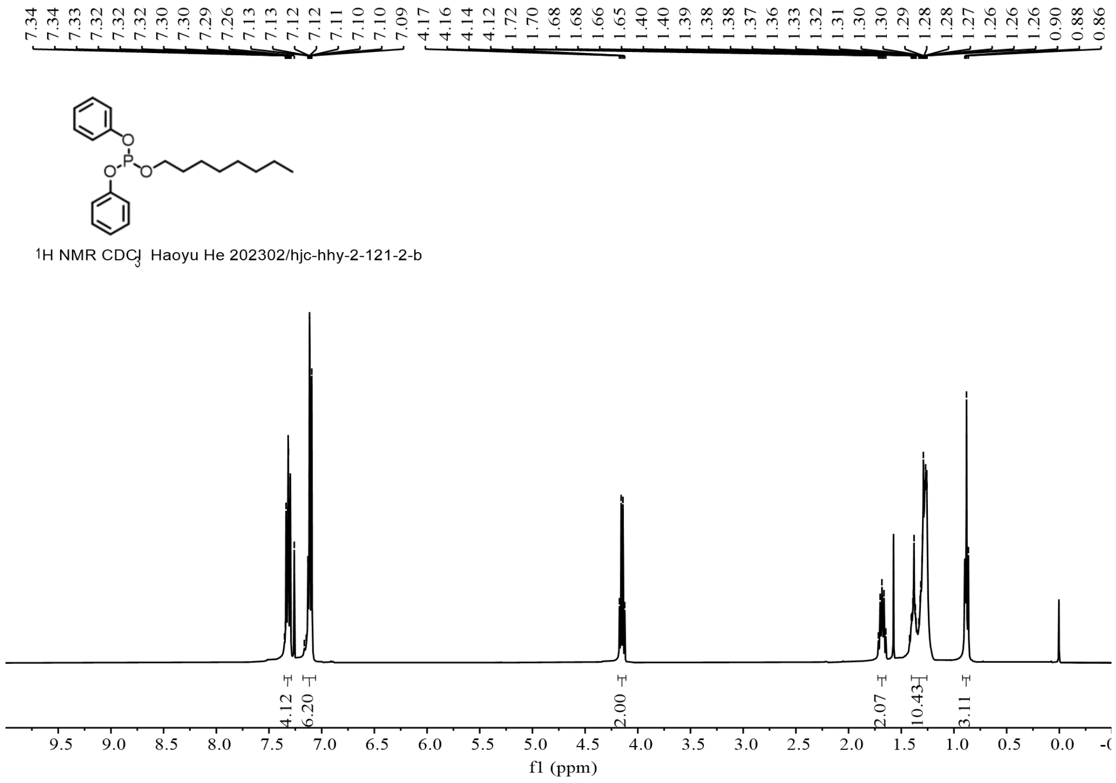

^{13}C NMR

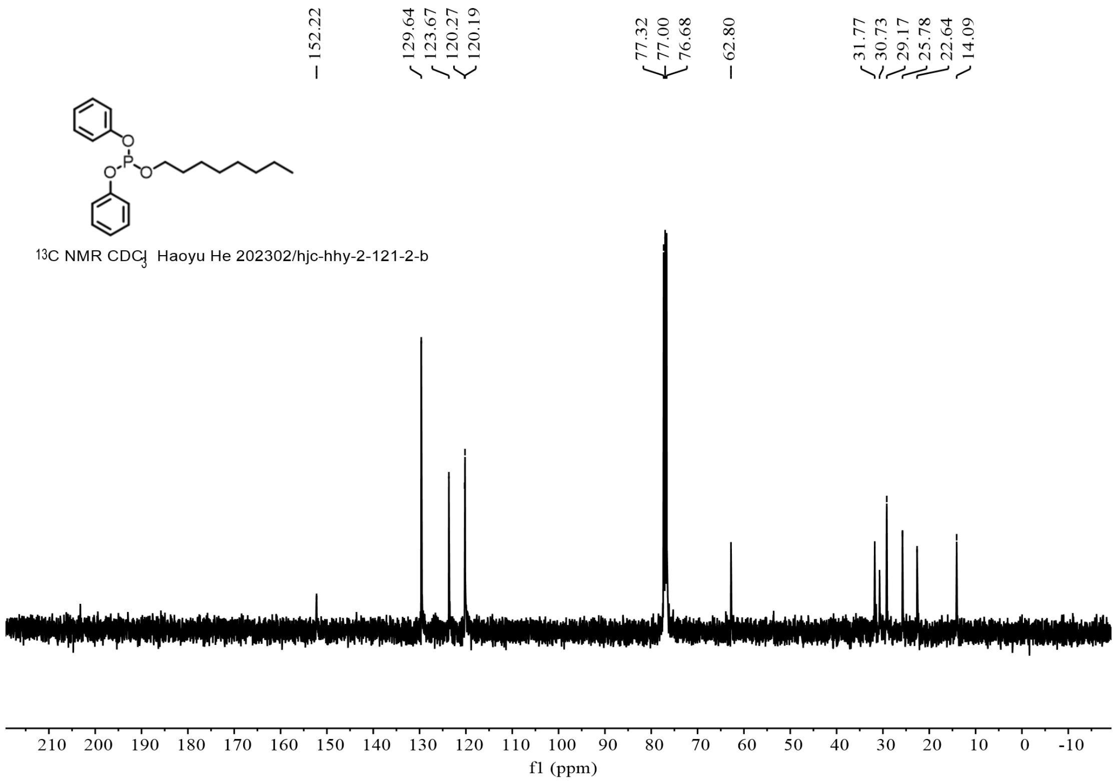

^{31}P NMR

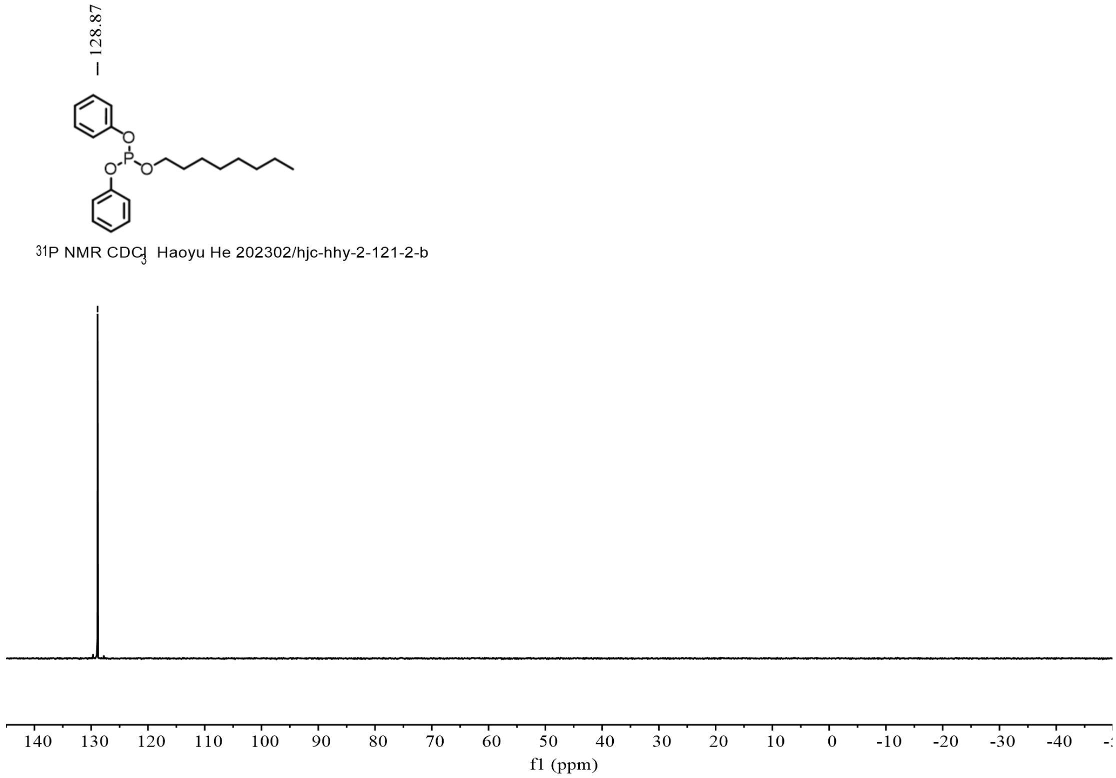

128.87
31P NMR CDCl3 Haoyu He 202302/hjc-hhy-2-121-2-b
140
130
120
110
100
90
80
70
60
50
40
30
20
10
0
-10
-20
-30
-40
f1 (ppm)

37

^{1}H NMR

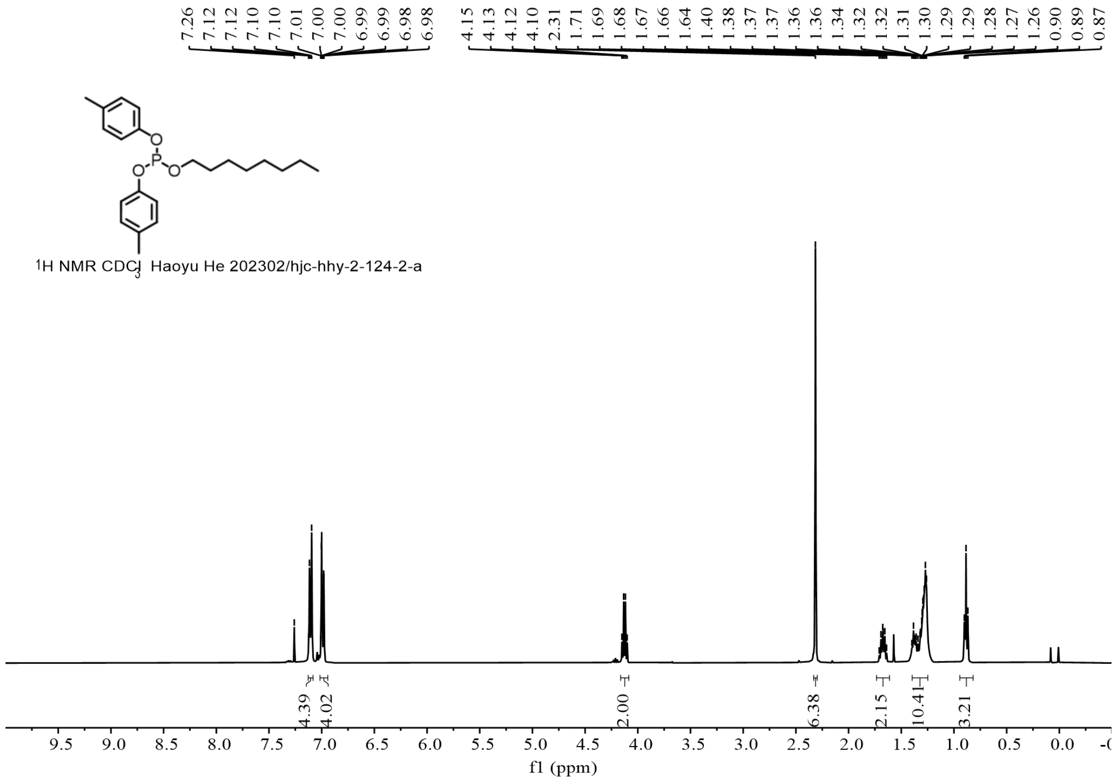

^{13}C NMR

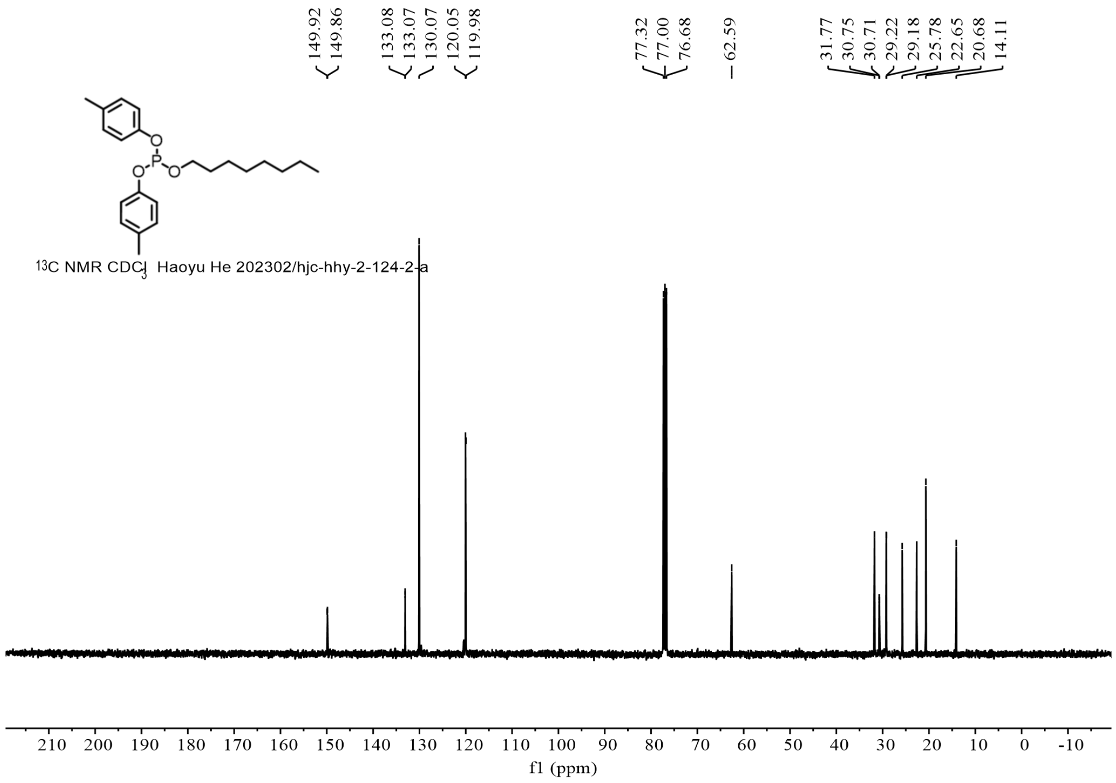

^{31}P NMR

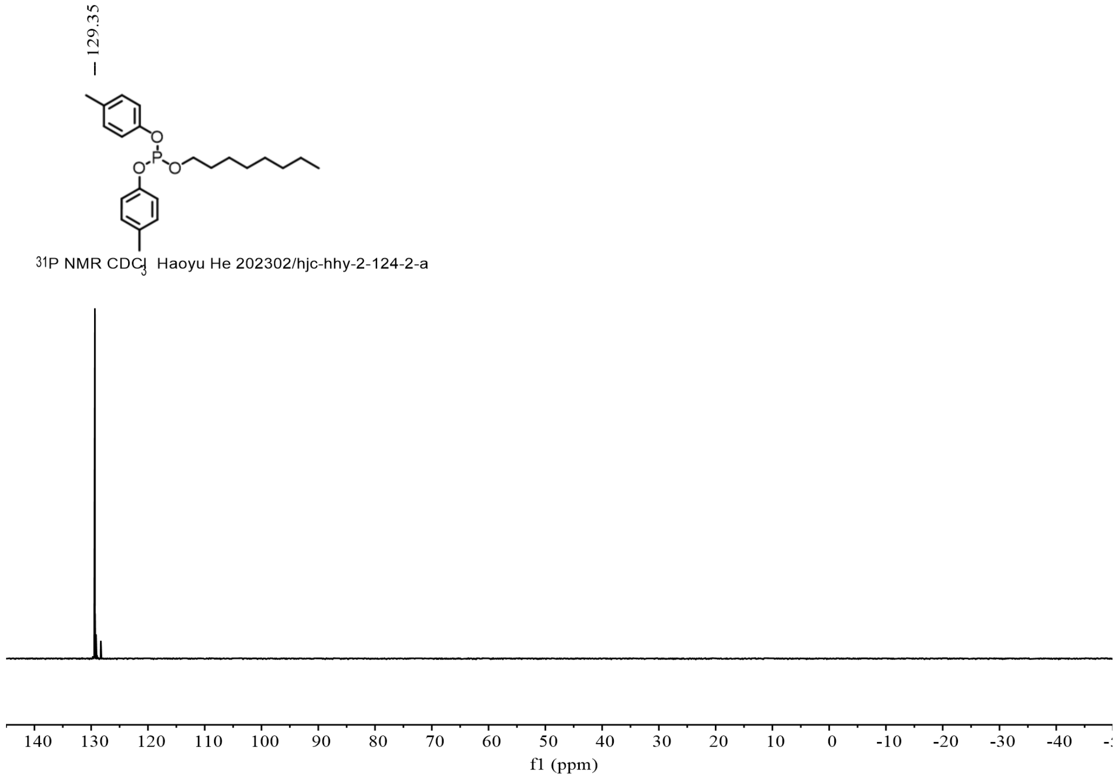
129.35
31P NMR CDCl3 Haoyu He 202302/hjc-hhy-2-124-2-a
140 130 120 110 100 90 80 70 60 50 40 30 20 10 0 -10 -20 -30 -40
f1 (ppm)

38

^{1}H NMR

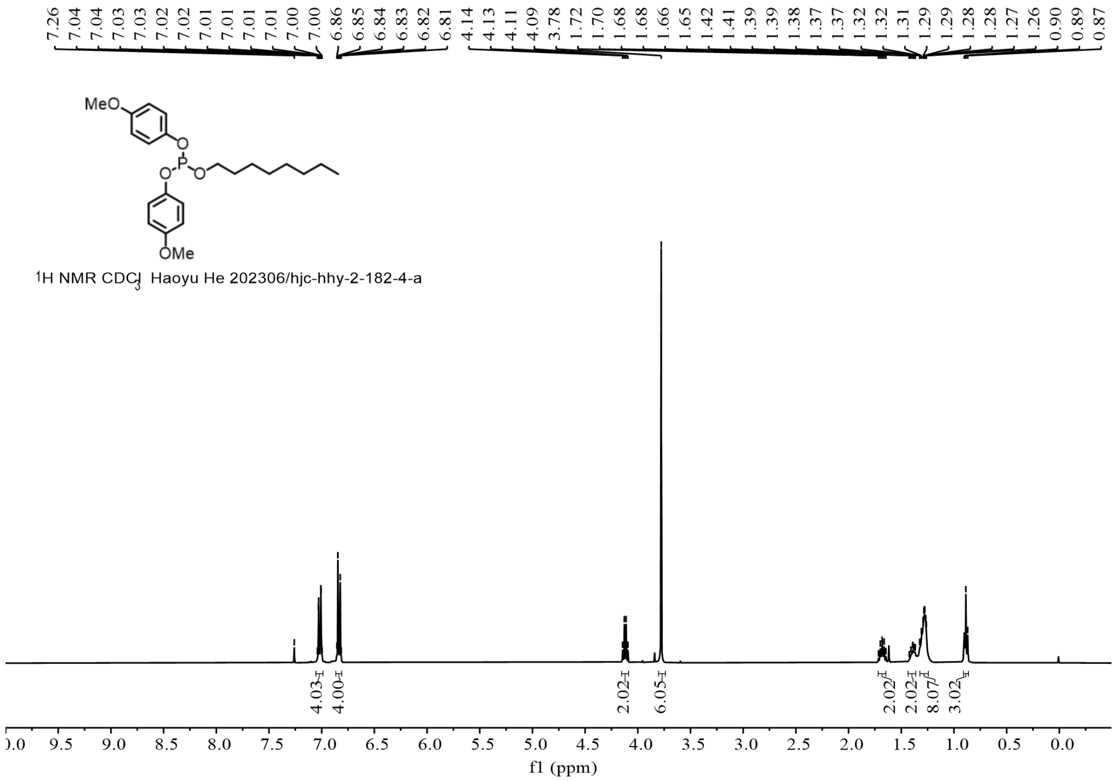

^{13}C NMR

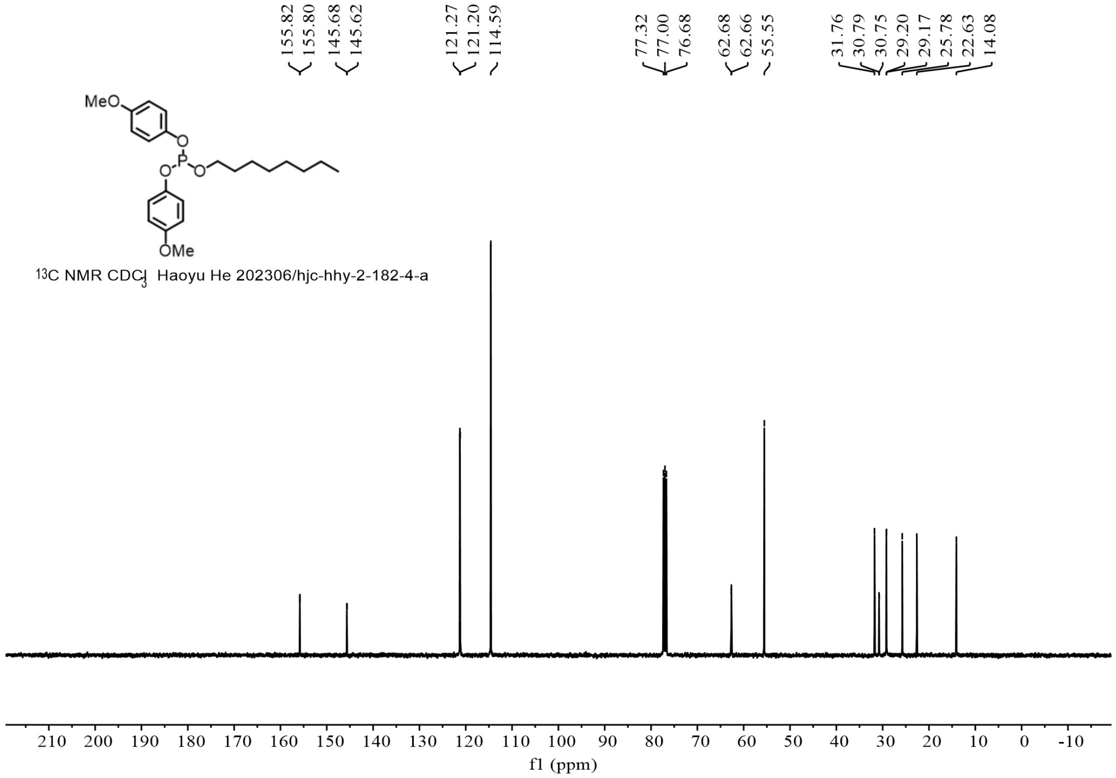

^{31}P NMR

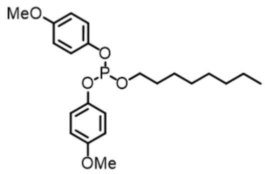

MeO
O
P
O
O
OMe

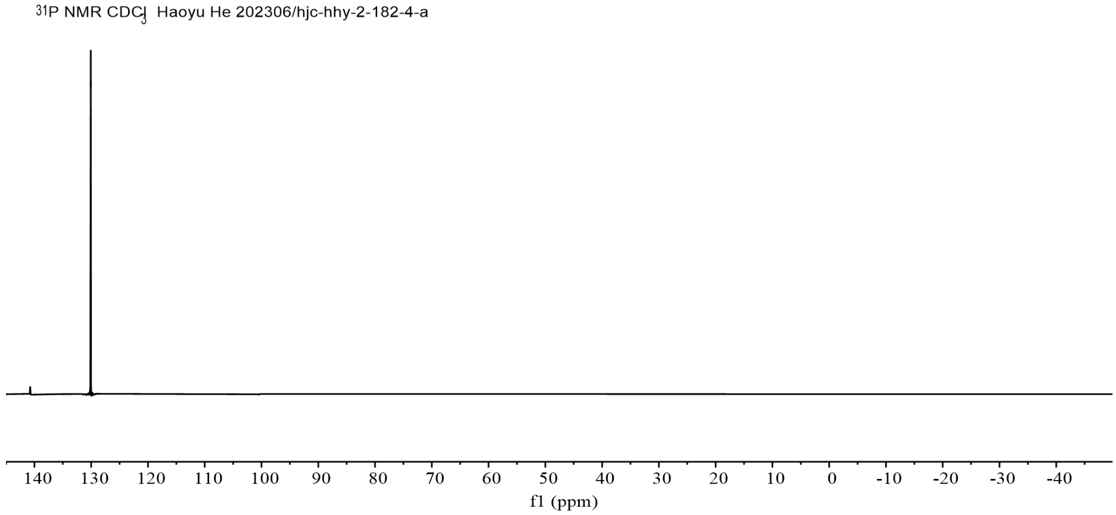

130.08
31P NMR CDCl3 Haoyu He 202306/hjc-hhy-2-182-4-a
140
130
120
110
100
90
80
70
60
50
40
30
20
10
0
-10
-20
-30
-40
f1 (ppm)

39

^{1}H NMR

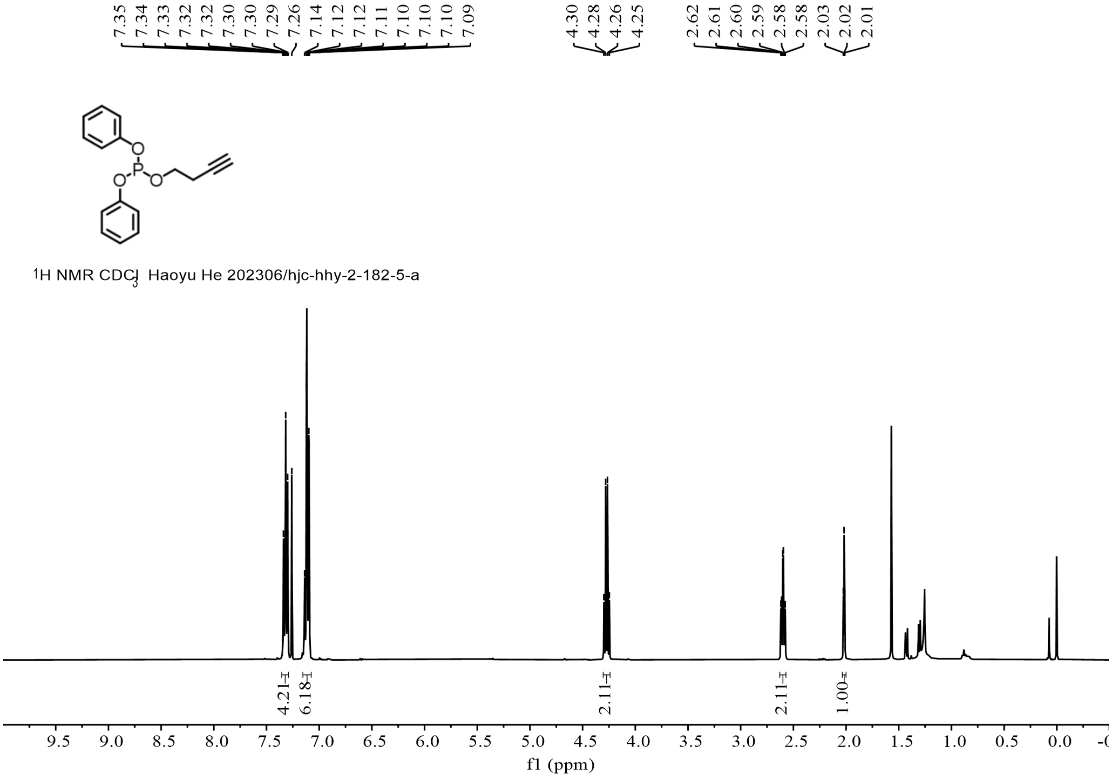

^{13}C NMR

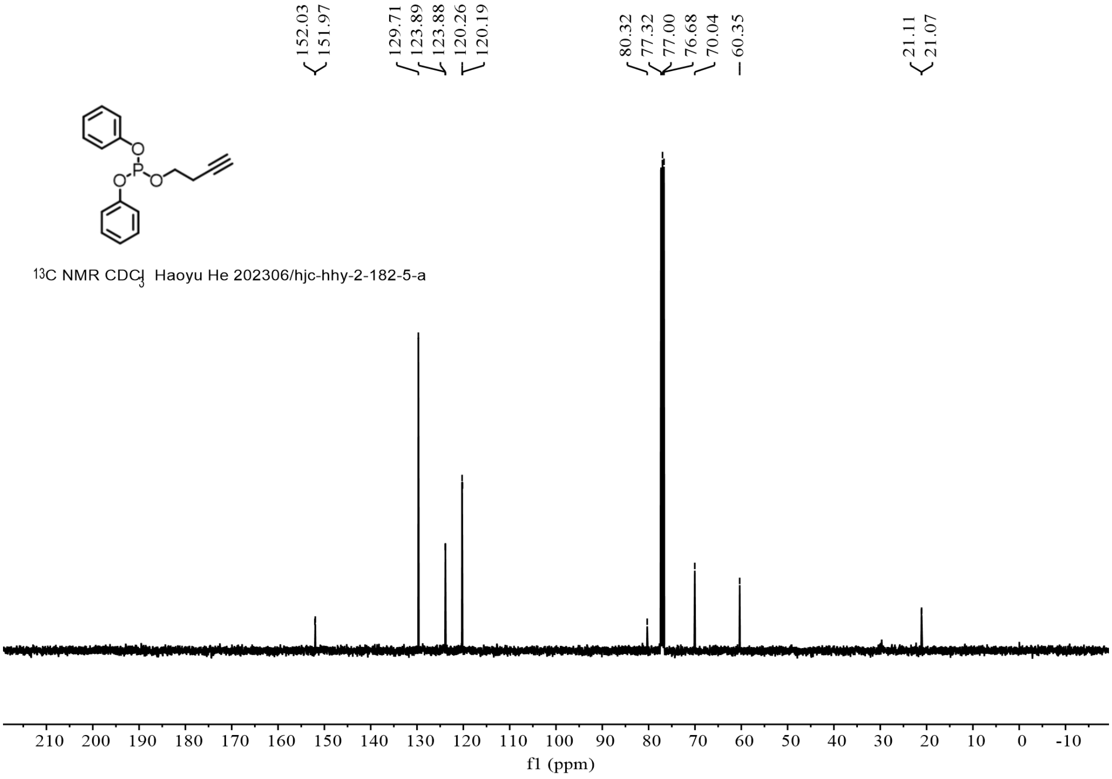

^{31}P NMR

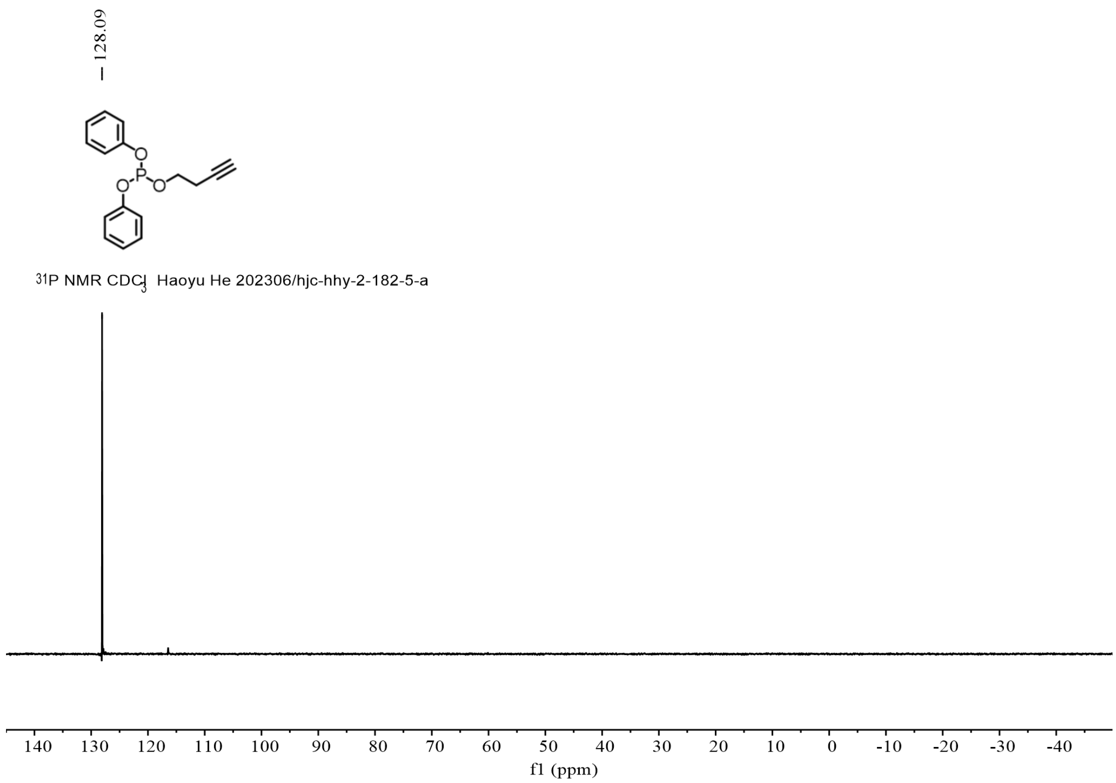

128.09
31P NMR CDCl3 Haoyu He 202306/hjc-hhy-2-182-5-a
140 130 120 110 100 90 80 70 60 50 40 30 20 10 0 -10 -20 -30 -40
f1 (ppm)

40

^{1}H NMR

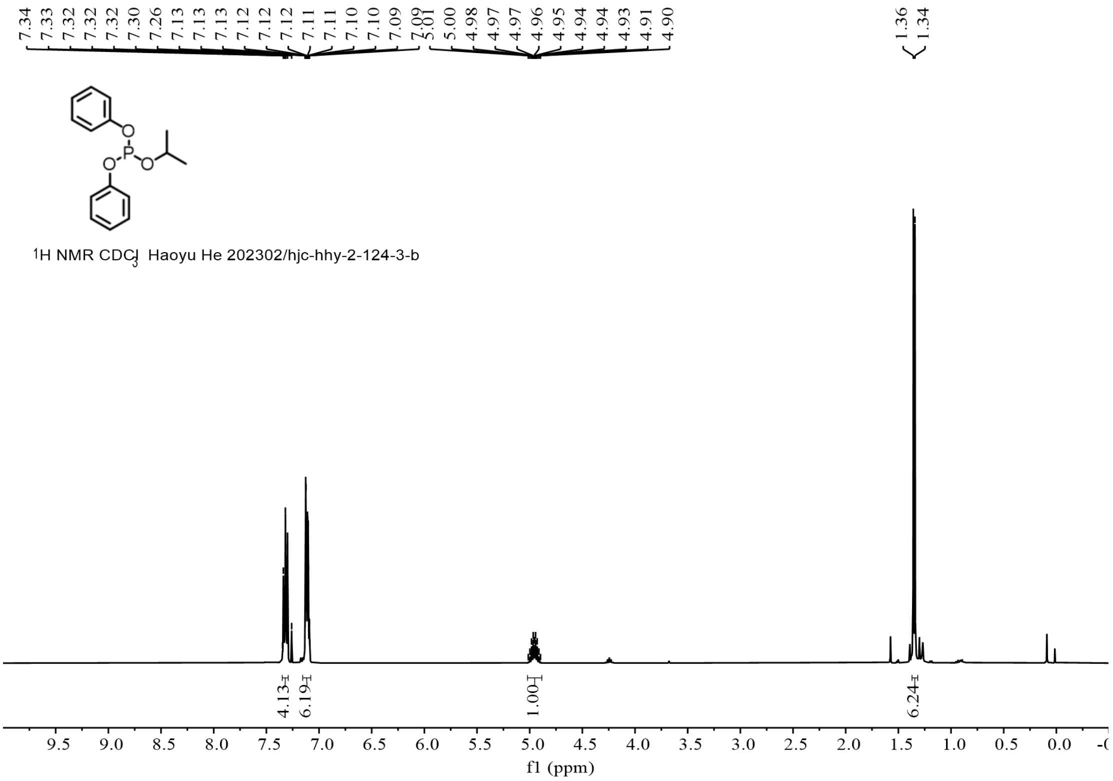

^{13}C NMR

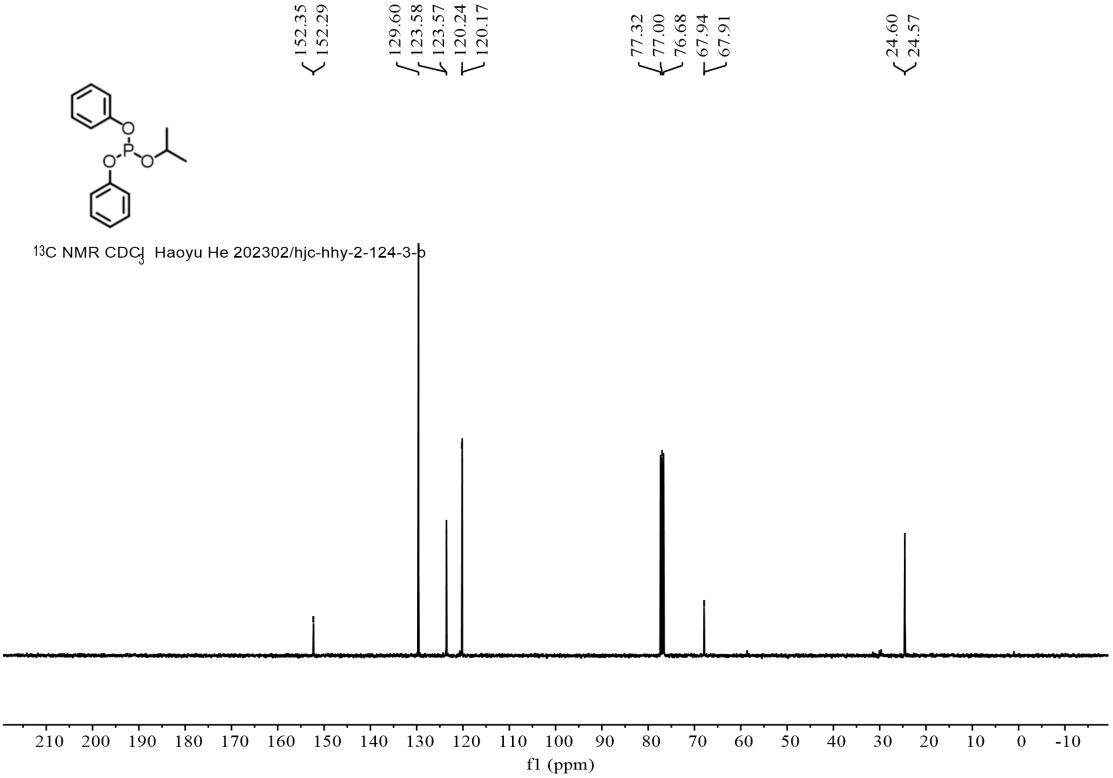

^{31}P NMR

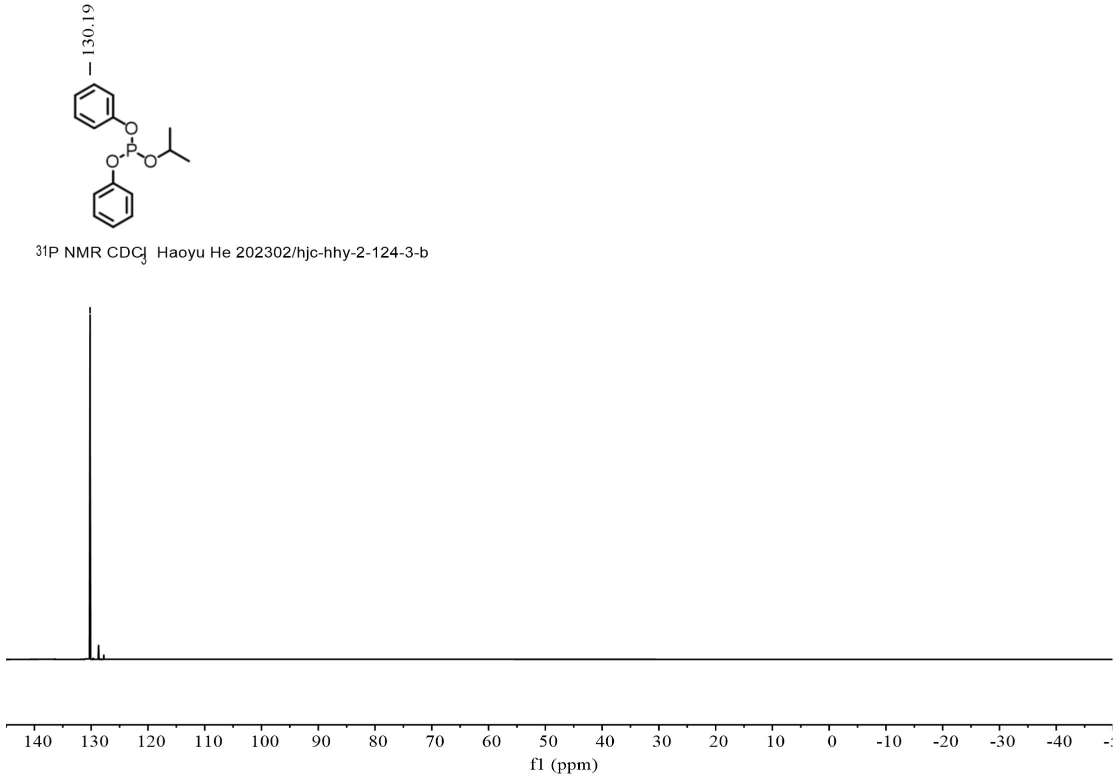

130.19
31P NMR CDCl3 Haoyu He 202302/hjc-hhy-2-124-3-b
140 130 120 110 100 90 80 70 60 50 40 30 20 10 0 -10 -20 -30 -40
f1 (ppm)

41

^{1}H NMR

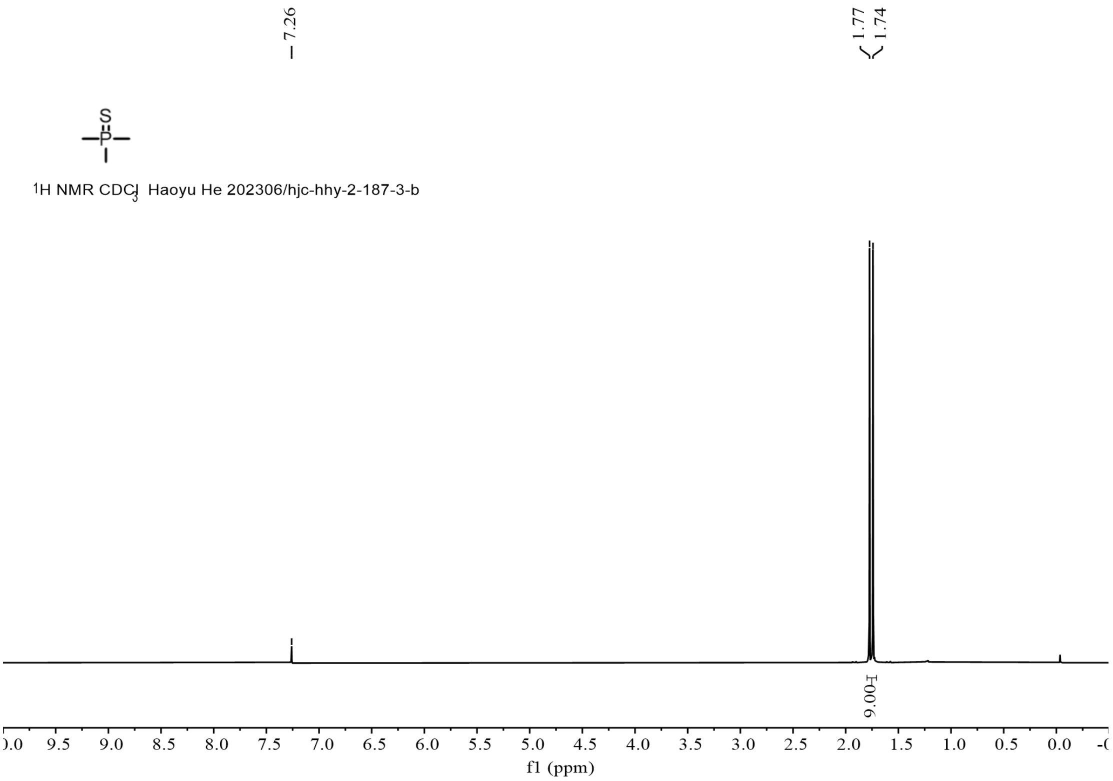

^{13}C NMR

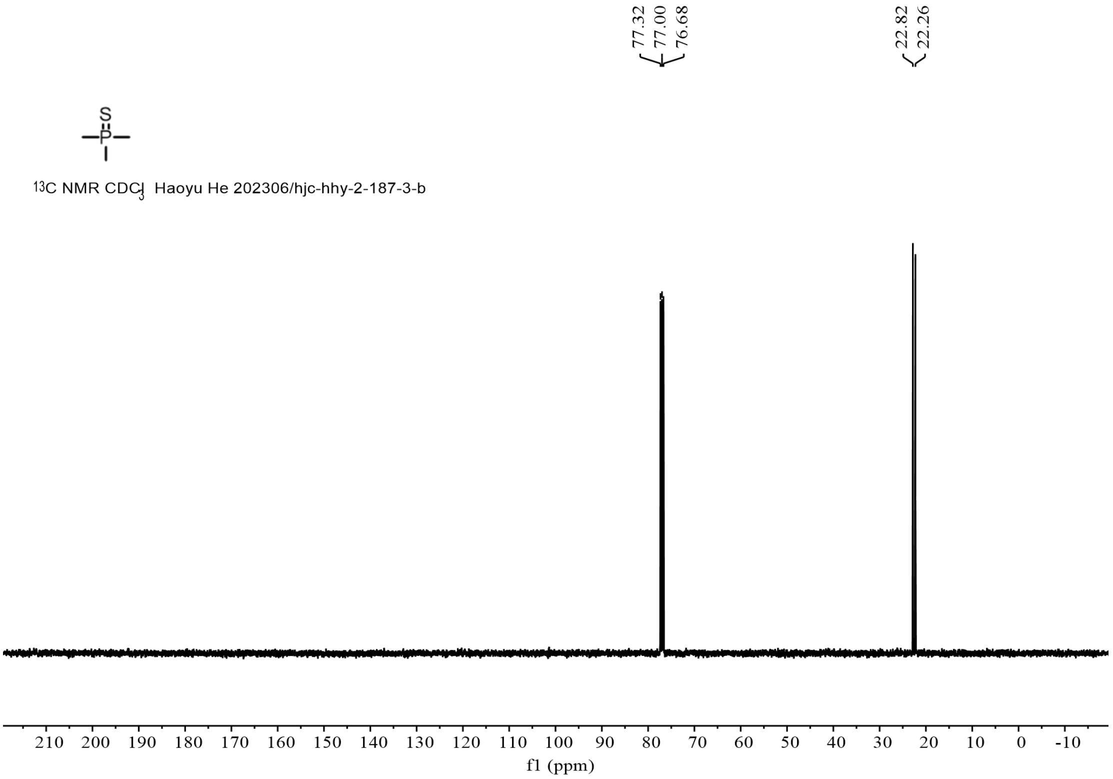

^{31}P NMR

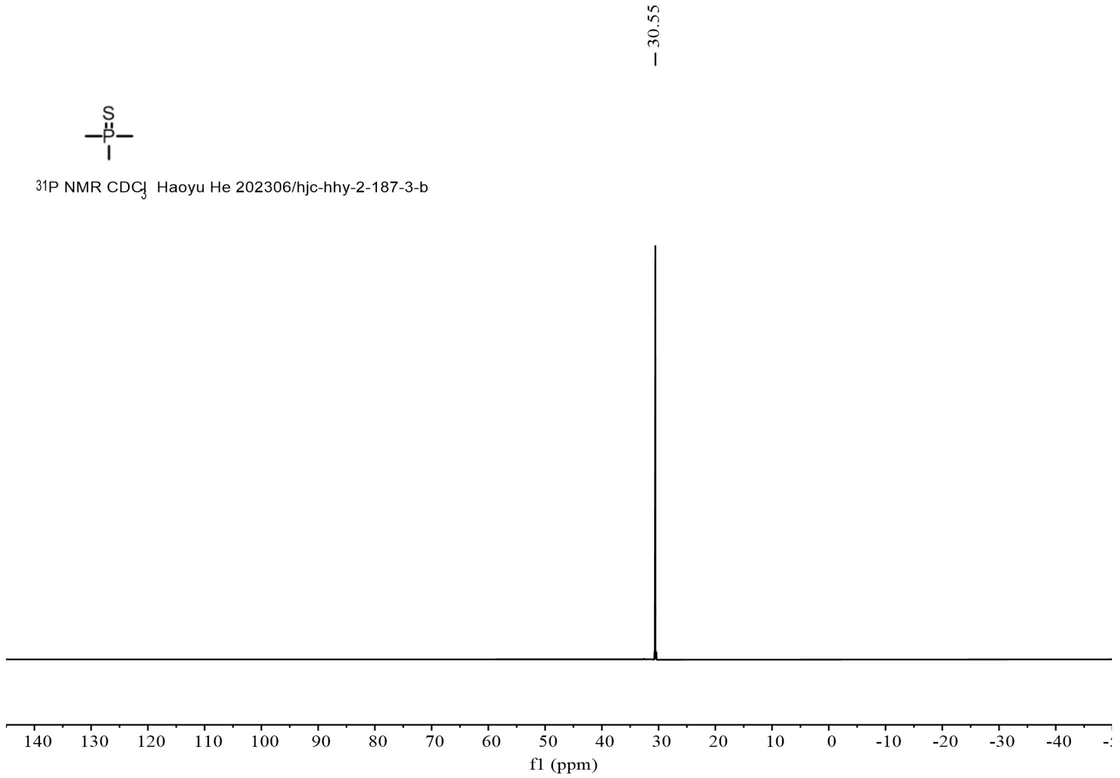

30.55
S
P
31P NMR CDCl3 Haoyu He 202306/hjc-hhy-2-187-3-b
140
130
120
110
100
90
80
70
60
50
40
30
20
10
0
-10
-20
-30
-40
f1 (ppm)

42

^{1}H NMR

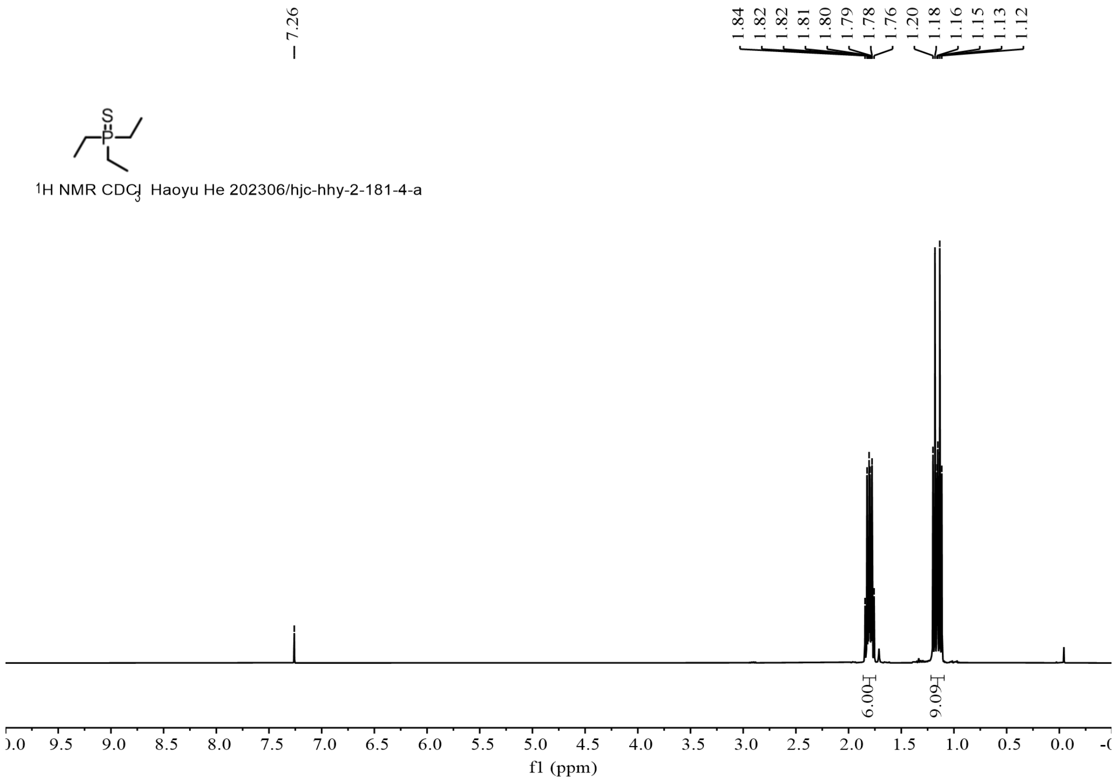

^{13}C NMR

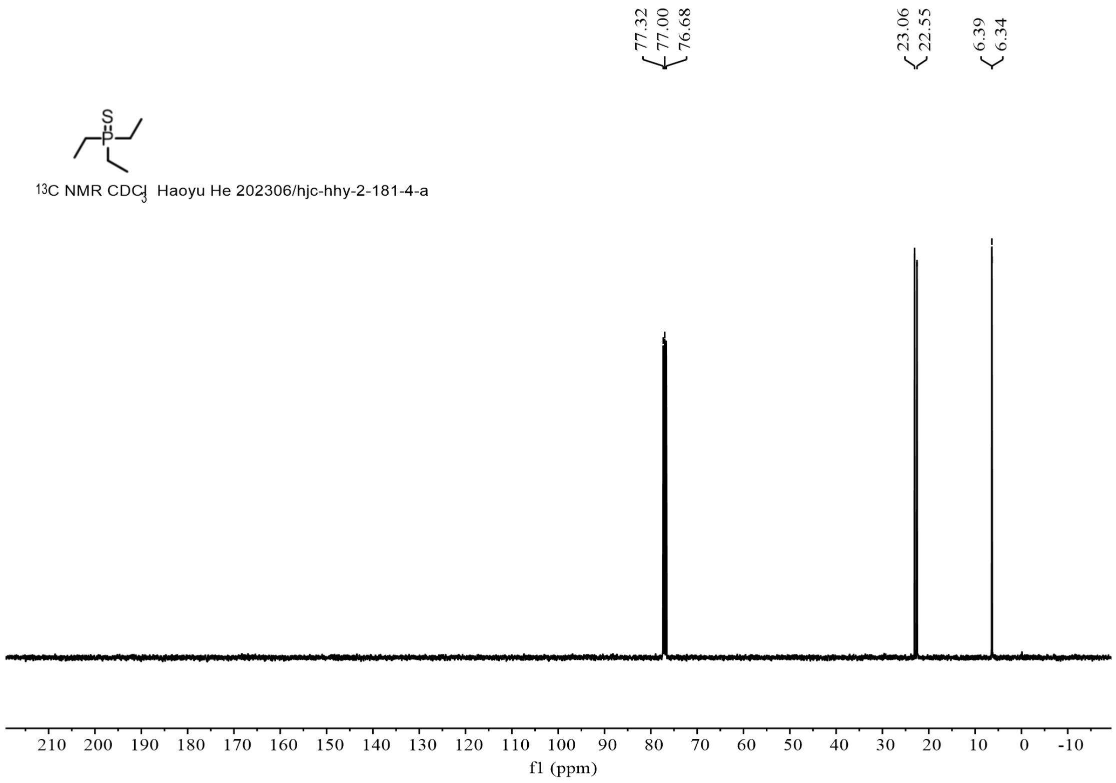

^{31}P NMR

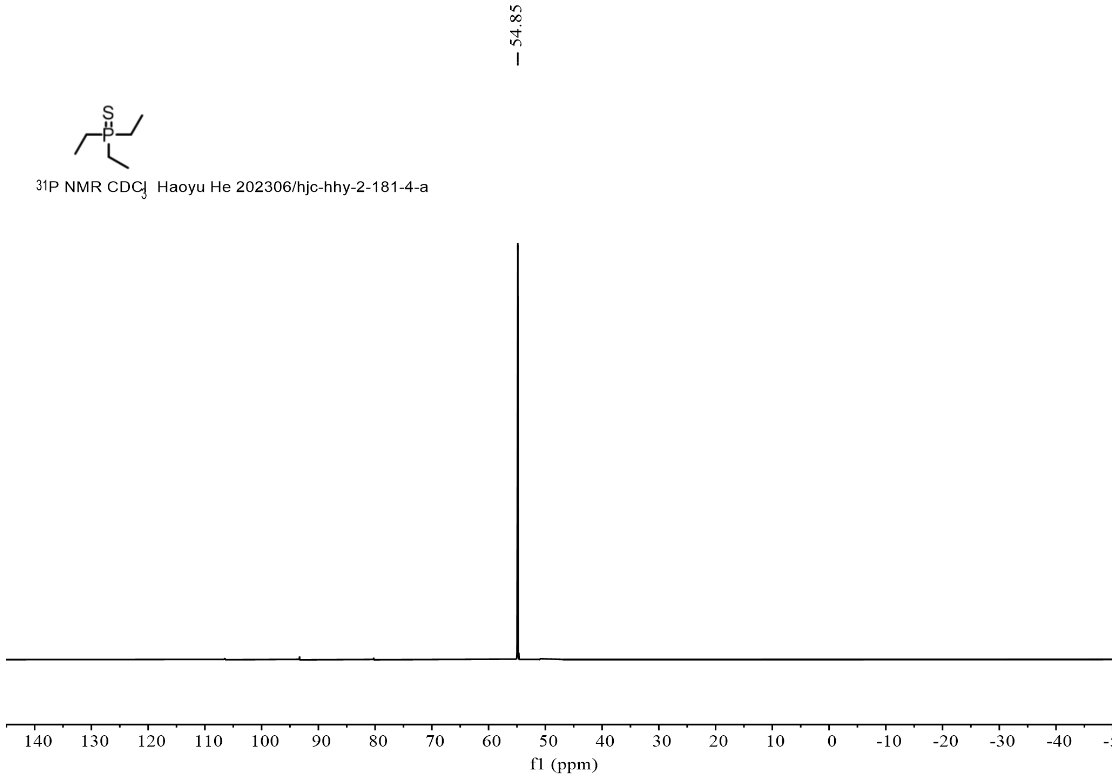

54.85
31P NMR CDCl3 Haoyu He 202306/hjc-hhy-2-181-4-a
140
130
120
110
100
90
80
70
60
50
40
30
20
10
0
-10
-20
-30
-40
f1 (ppm)

43

^{1}H NMR

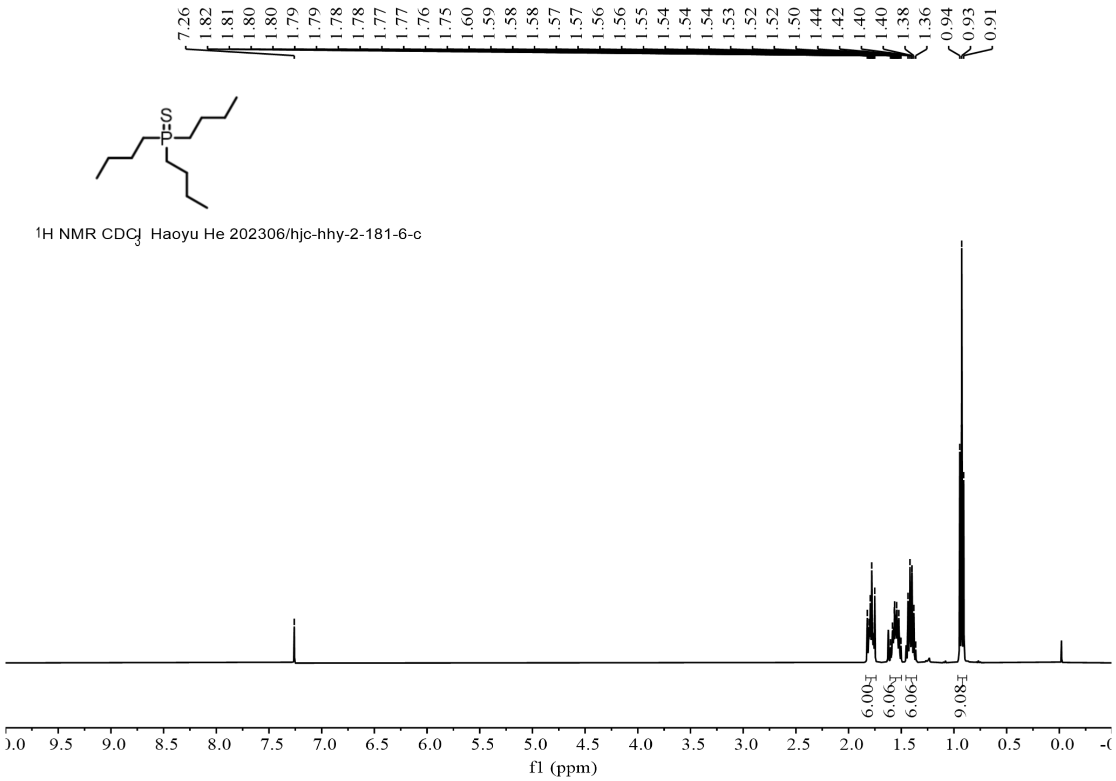

^{13}C NMR

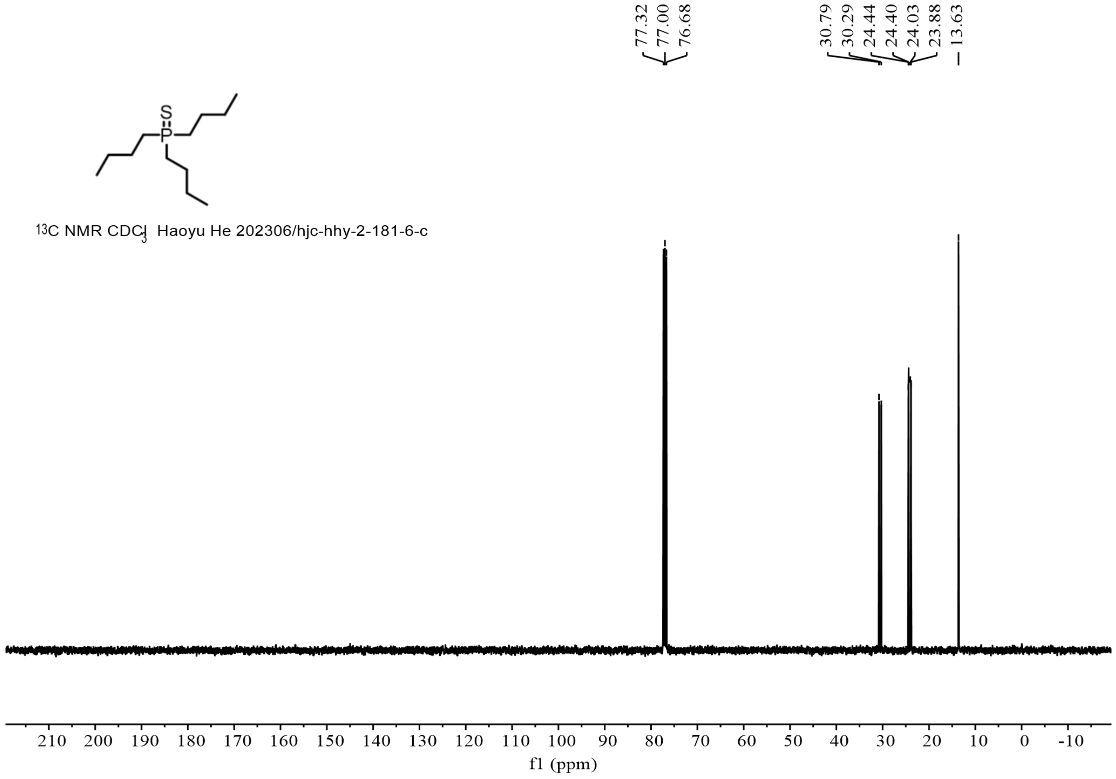

^{31}P NMR

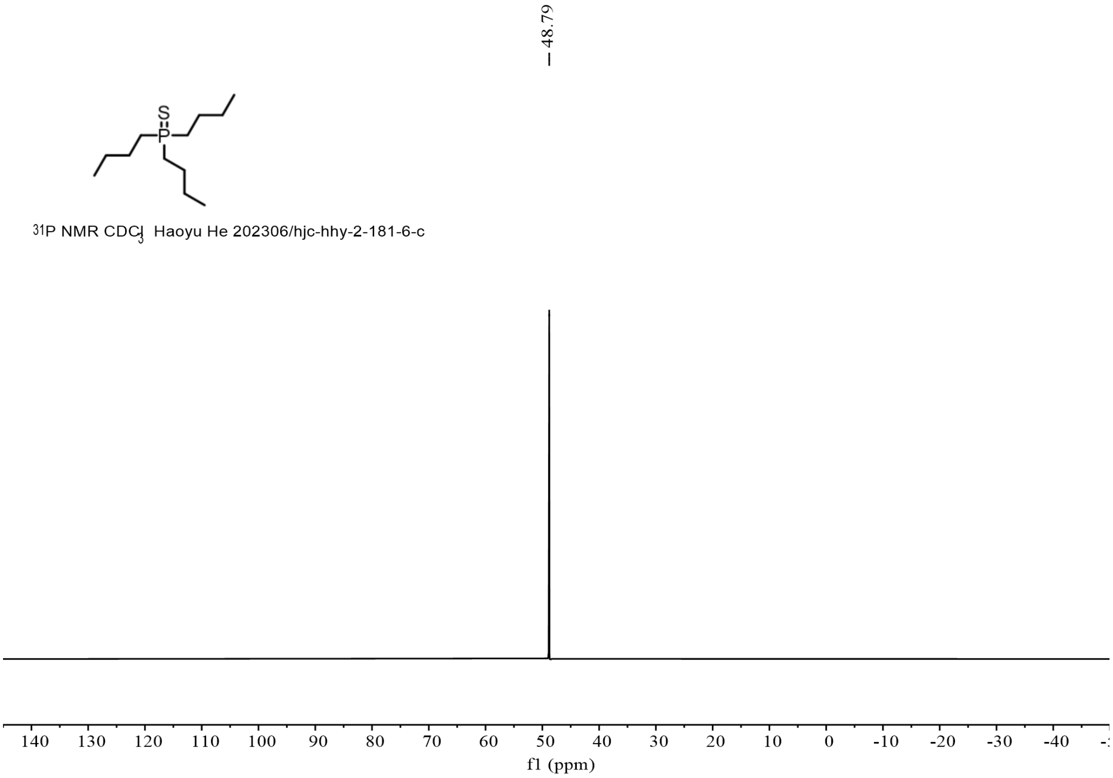
48.79
31P NMR CDCl3 Haoyu He 202306/hjc-hhy-2-181-6-c
140 130 120 110 100 90 80 70 60 50 40 30 20 10 0 -10 -20 -30 -40
f1 (ppm)

44

^{1}H NMR

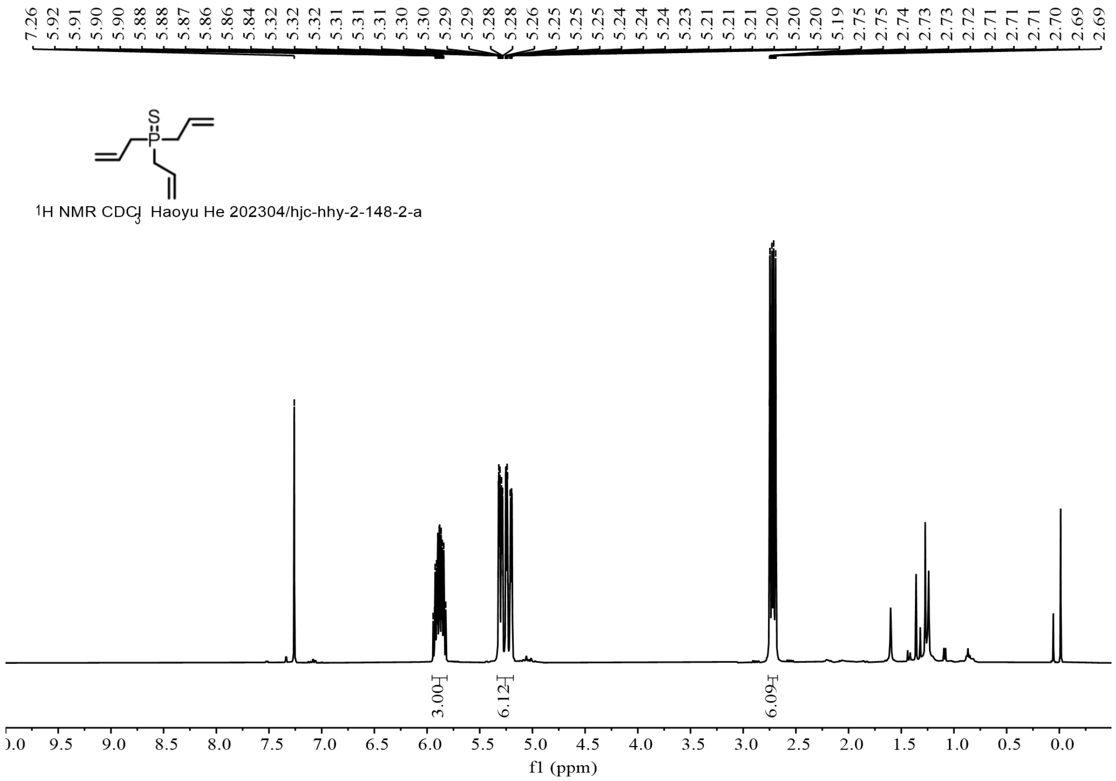

^{13}C NMR

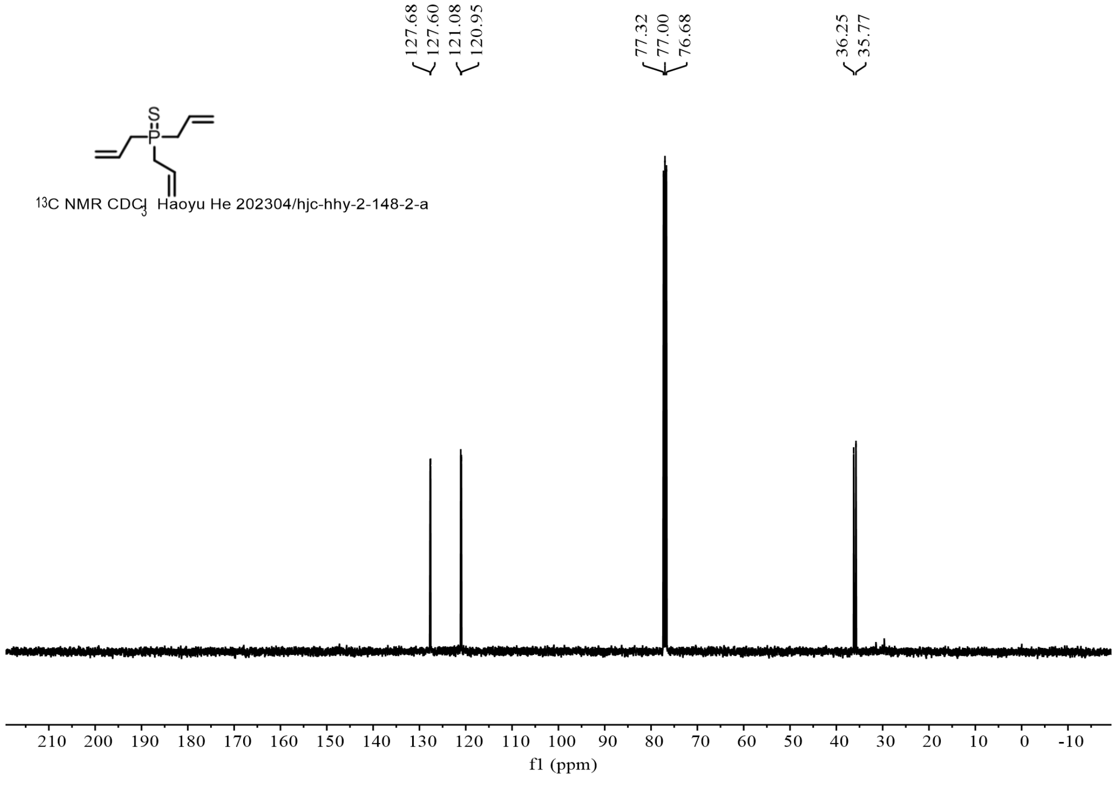

^{31}P NMR

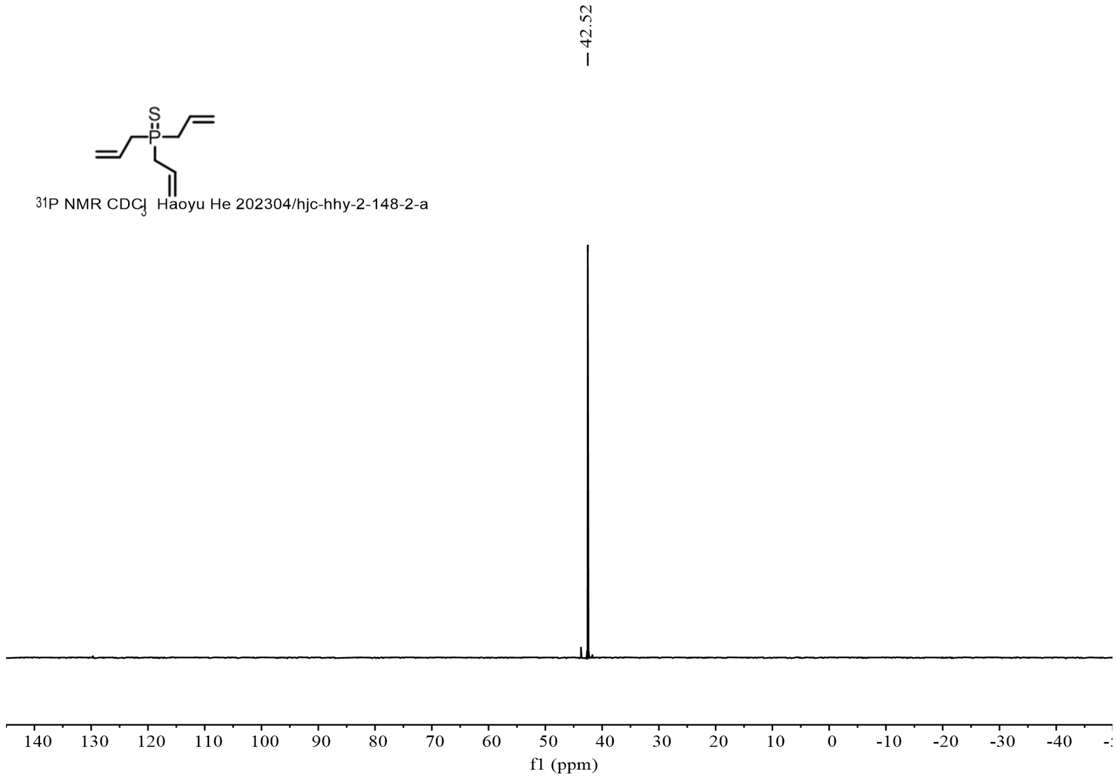

42.52
S
P
31P NMR CDCl3 Haoyu He 202304/hjc-hhy-2-148-2-a
140
130
120
110
100
90
80
70
60
50
40
30
20
10
0
-10
-20
-30
-40
f1 (ppm)

45

^{1}H NMR

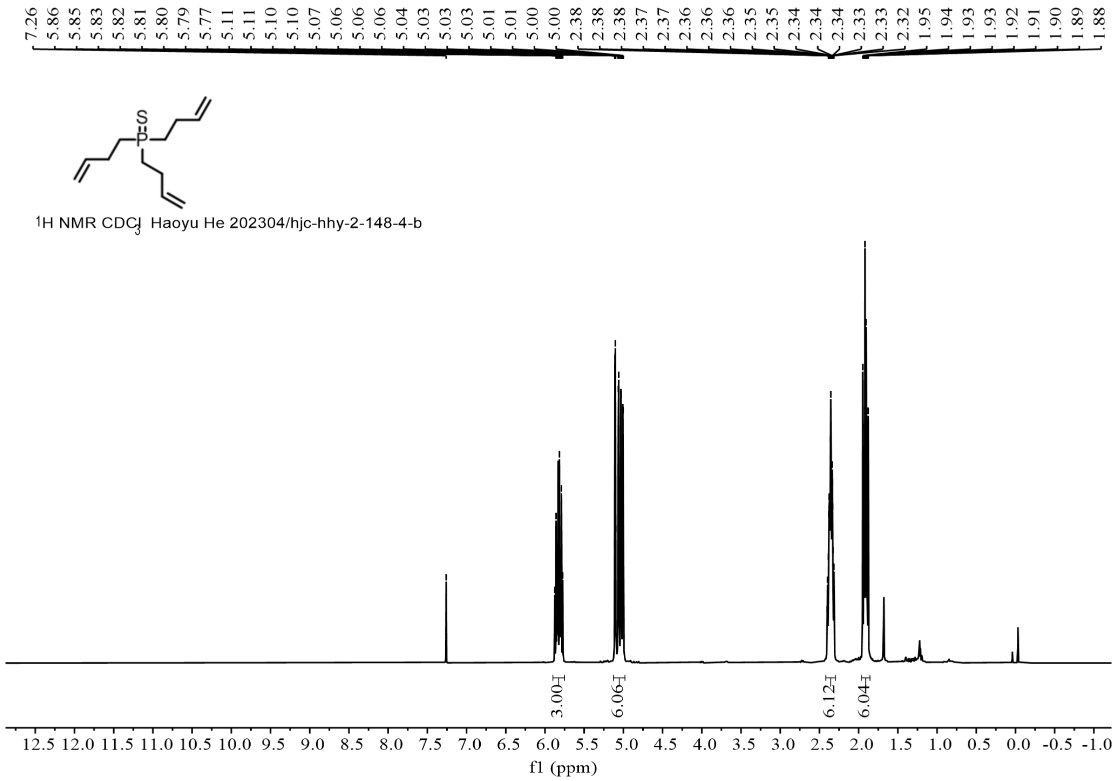

^{13}C NMR

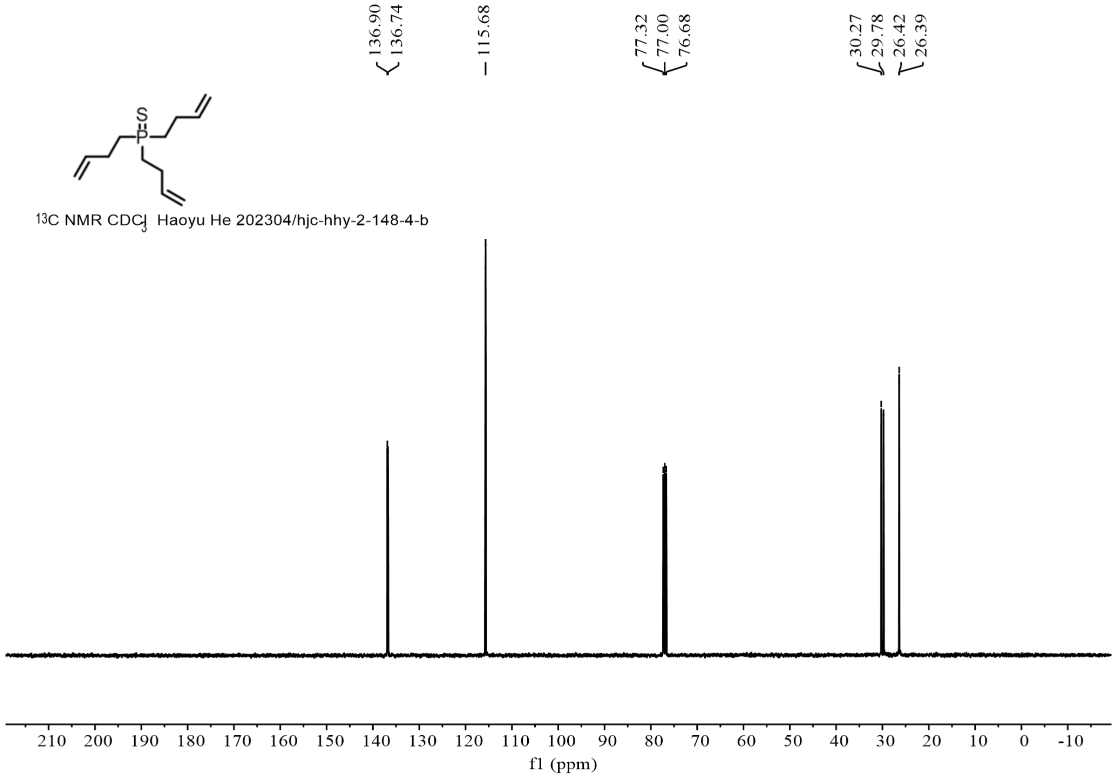

^{31}P NMR

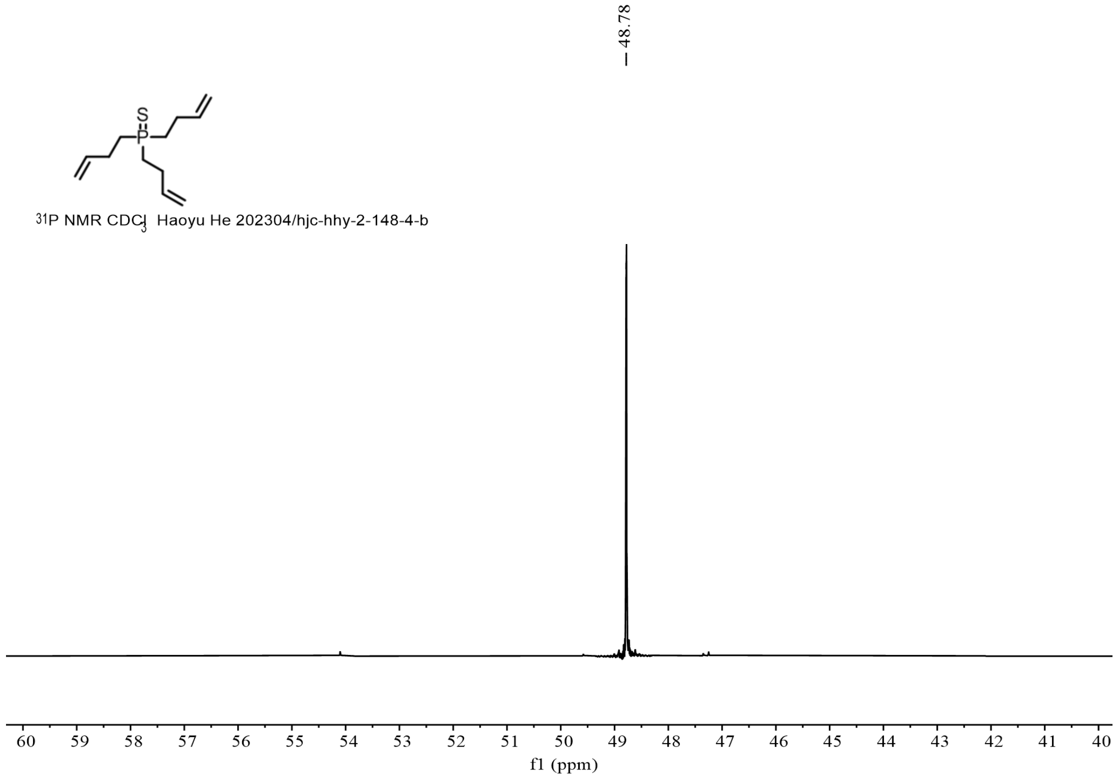
— 48.78
S
P
31P NMR CDCl3 Haoyu He 202304/hjc-hhy-2-148-4-b
60
59
58
57
56
55
54
53
52
51
50
49
48
47
46
45
44
43
42
41
40
f1 (ppm)

46

^{1}H NMR

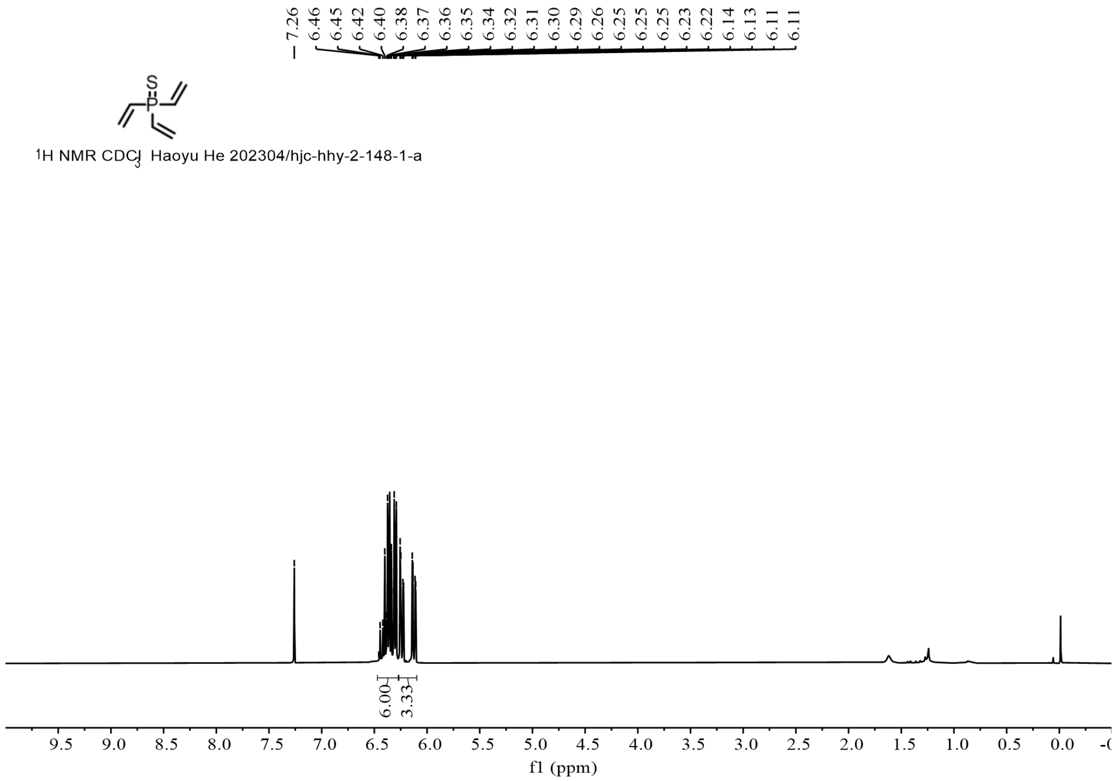

^{13}C NMR

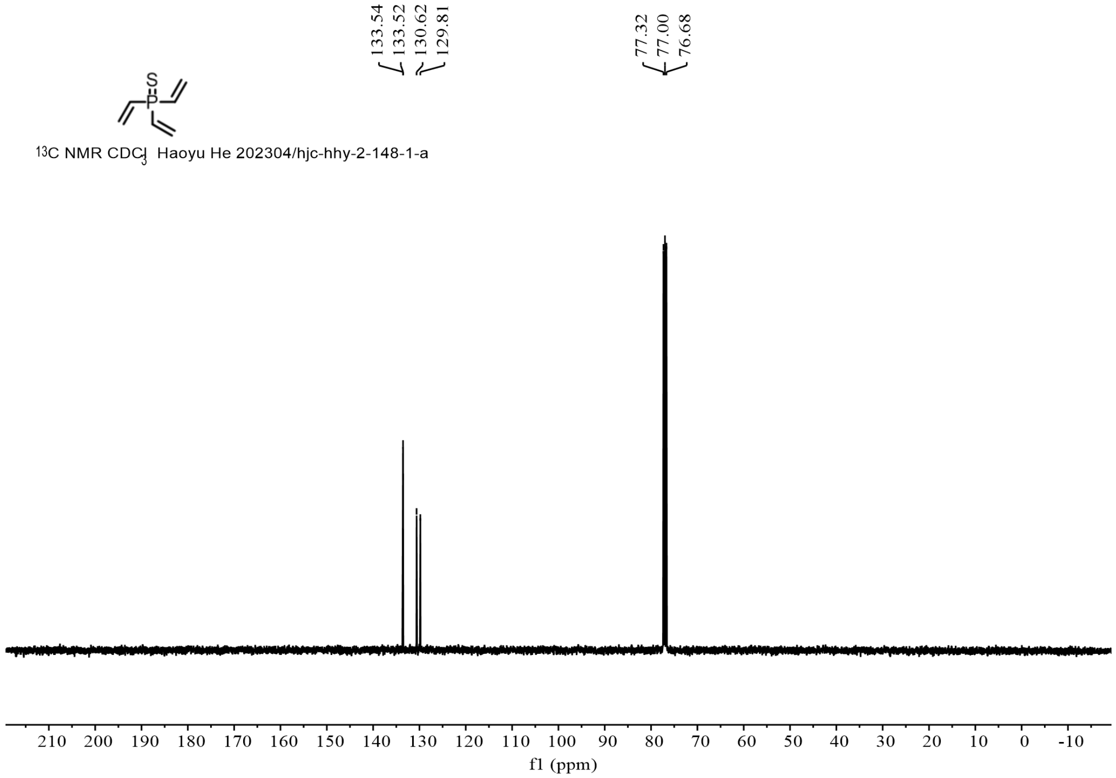

^{31}P NMR

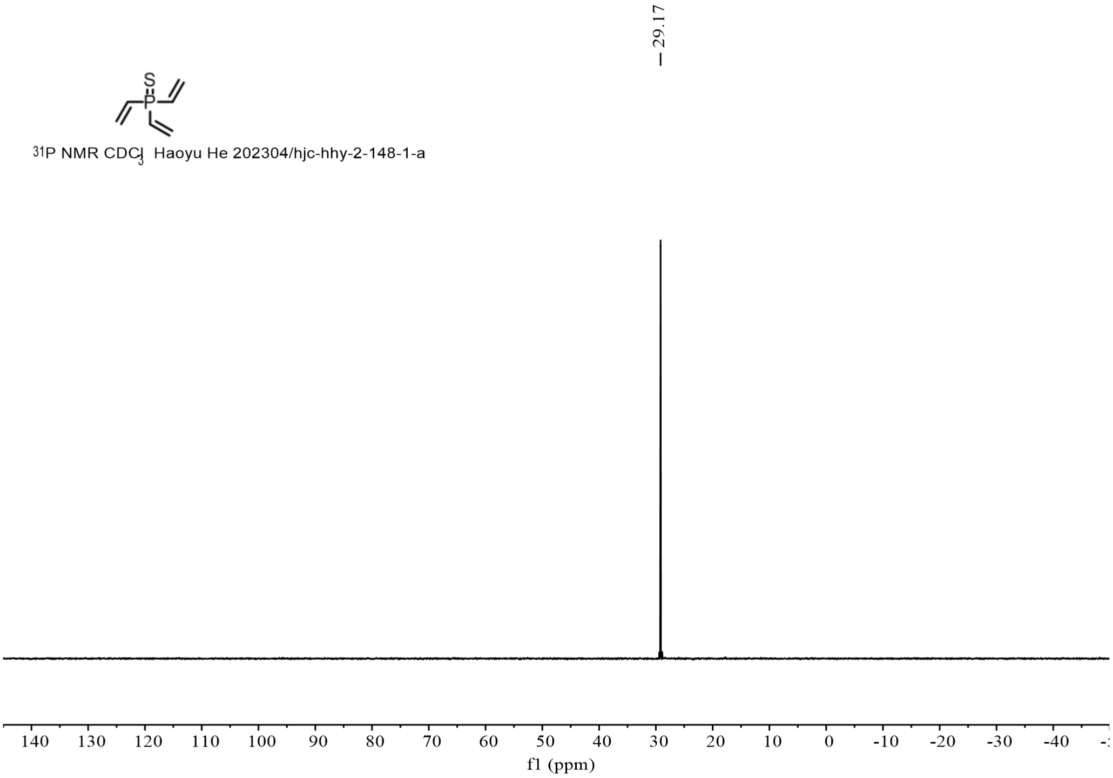

29.17
31P NMR CDCl3 Haoyu He 202304/hjc-hhy-2-148-1-a
140 130 120 110 100 90 80 70 60 50 40 30 20 10 0 -10 -20 -30 -40
f1 (ppm)

47

^{1}H NMR

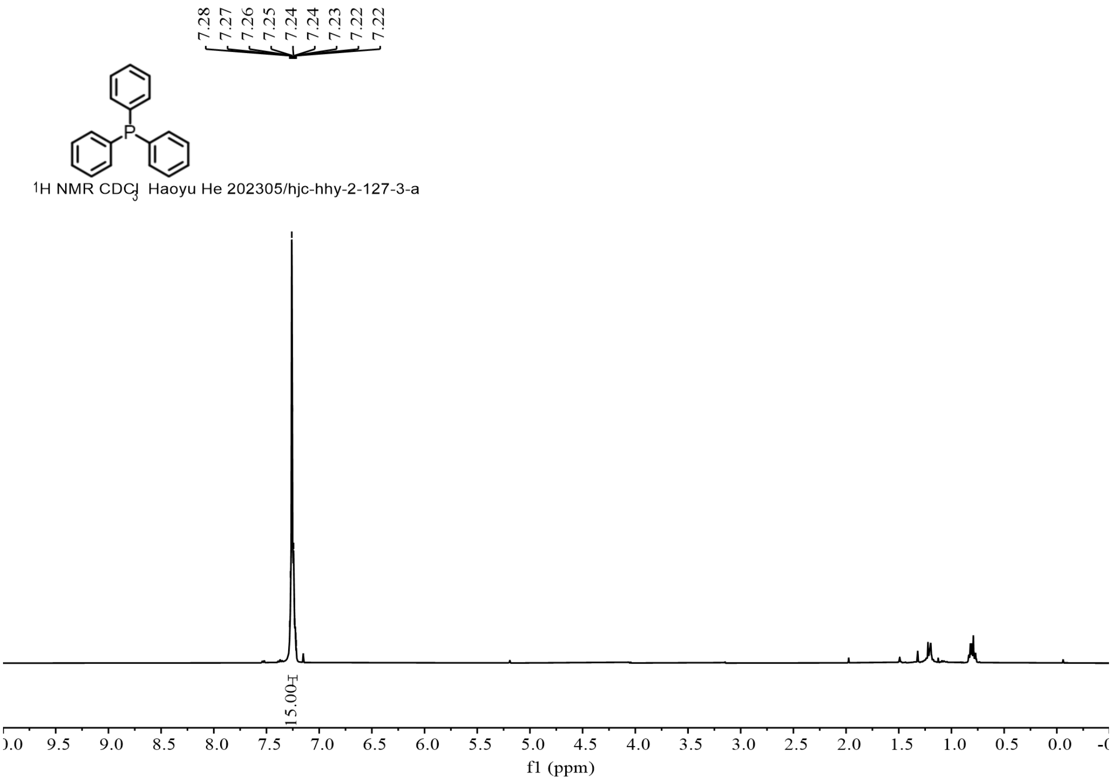

^{13}C NMR

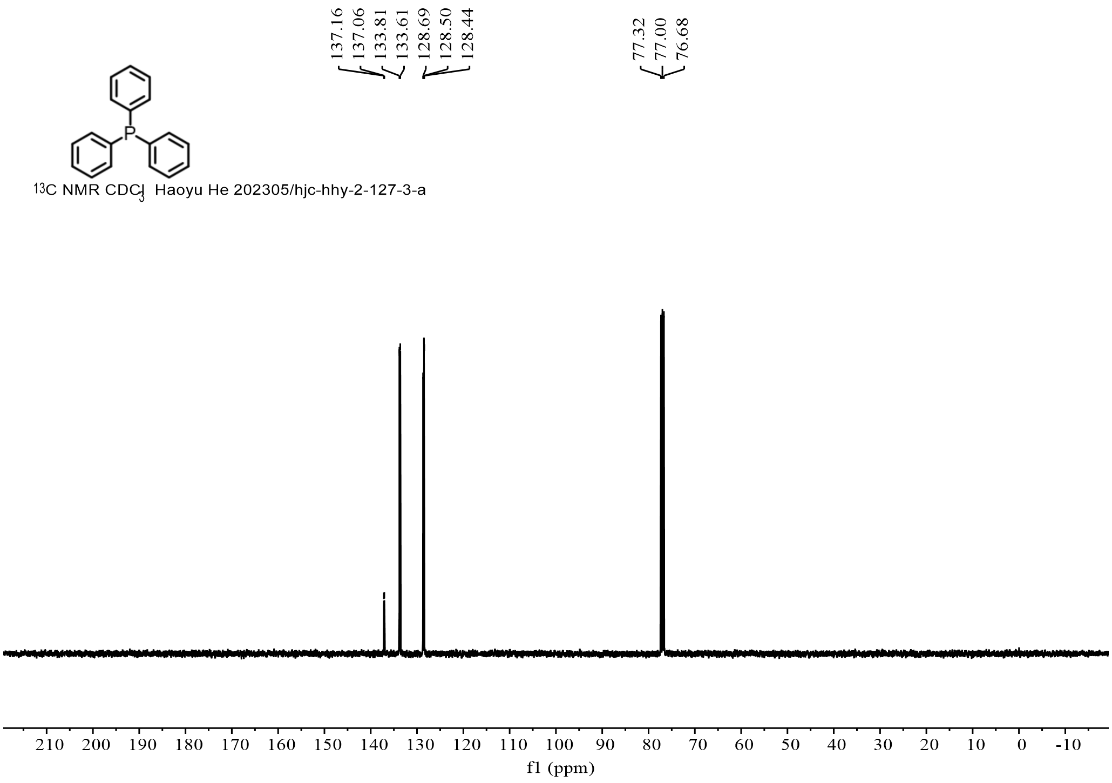

^{31}P NMR

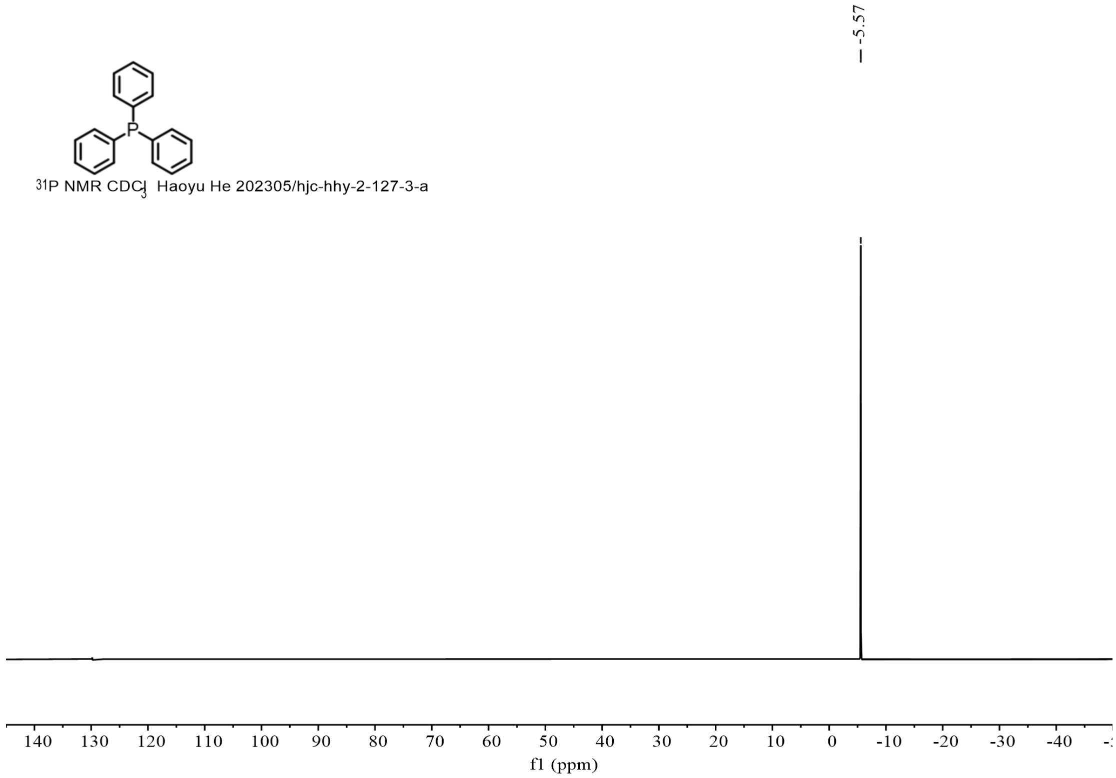

-5.57
P
31P NMR CDCl3 Haoyu He 202305/hjc-hhy-2-127-3-a
140 130 120 110 100 90 80 70 60 50 40 30 20 10 0 -10 -20 -30 -40
f1 (ppm)

48

^{1}H NMR

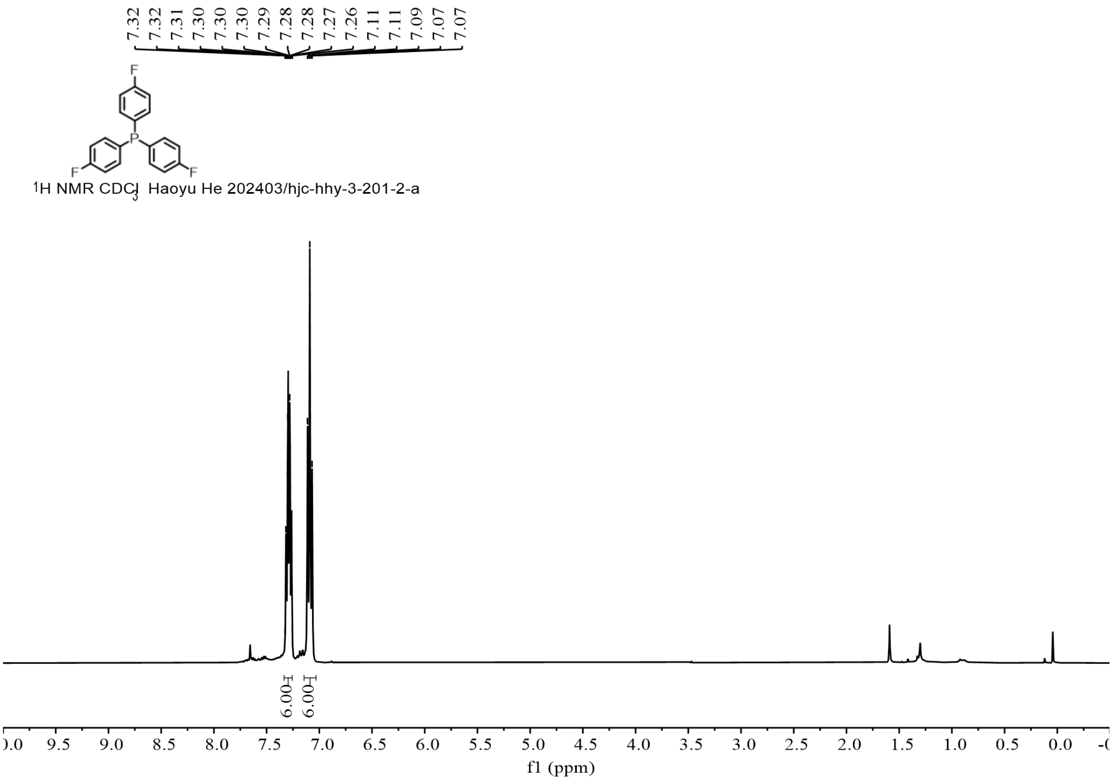

^{13}C NMR

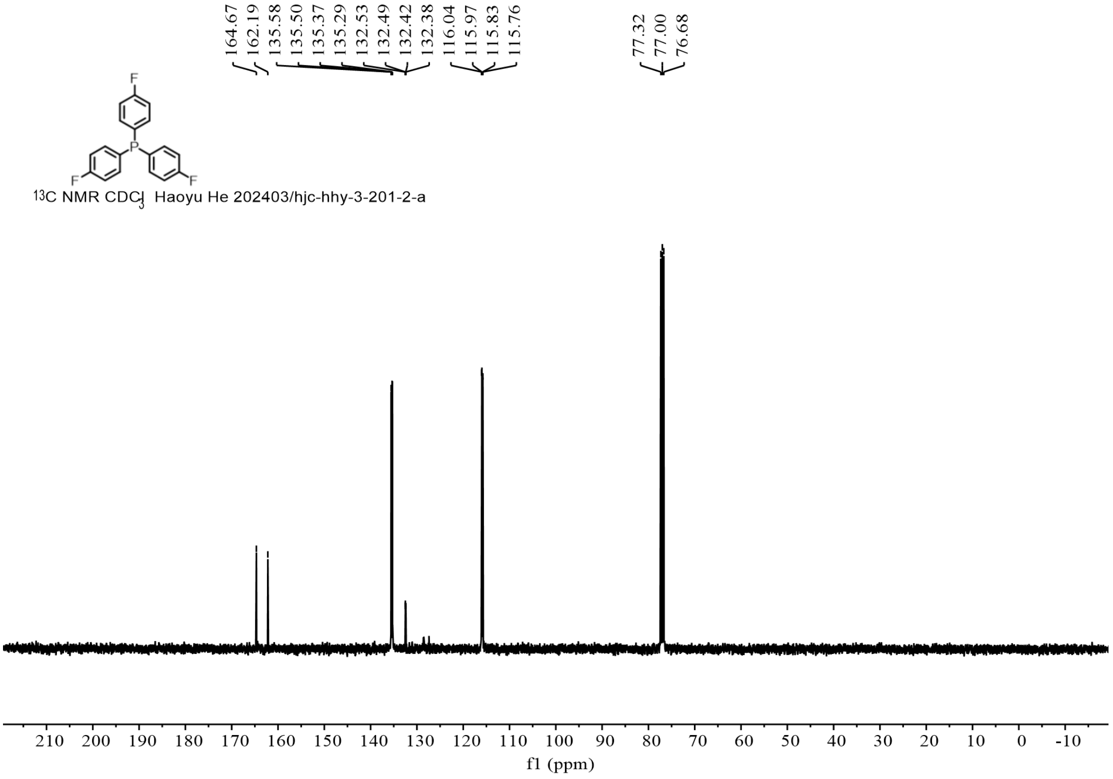

^{31}P NMR

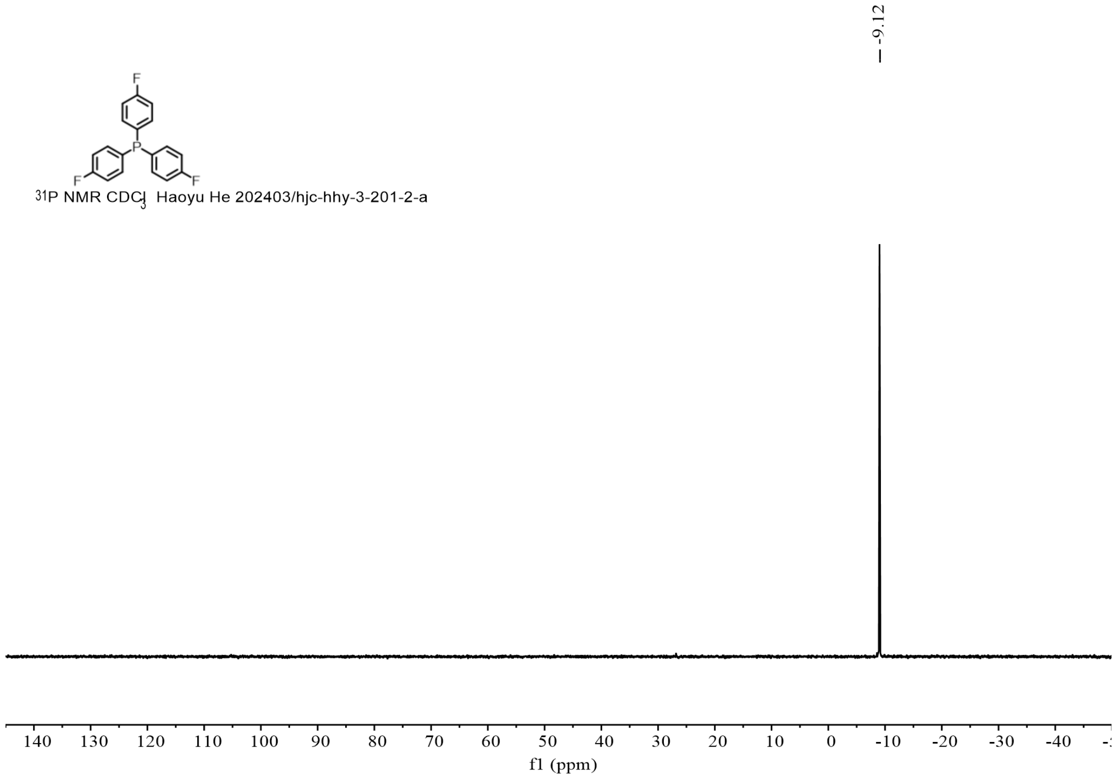

^{19}F NMR

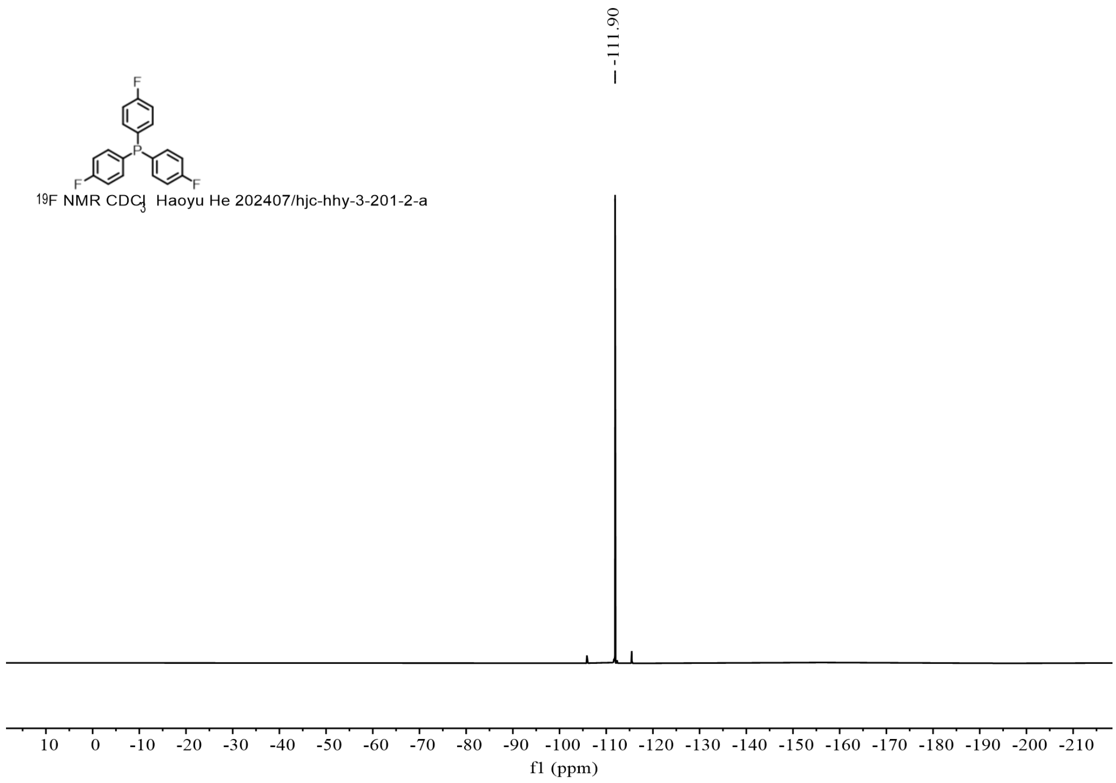

49

^{1}H NMR

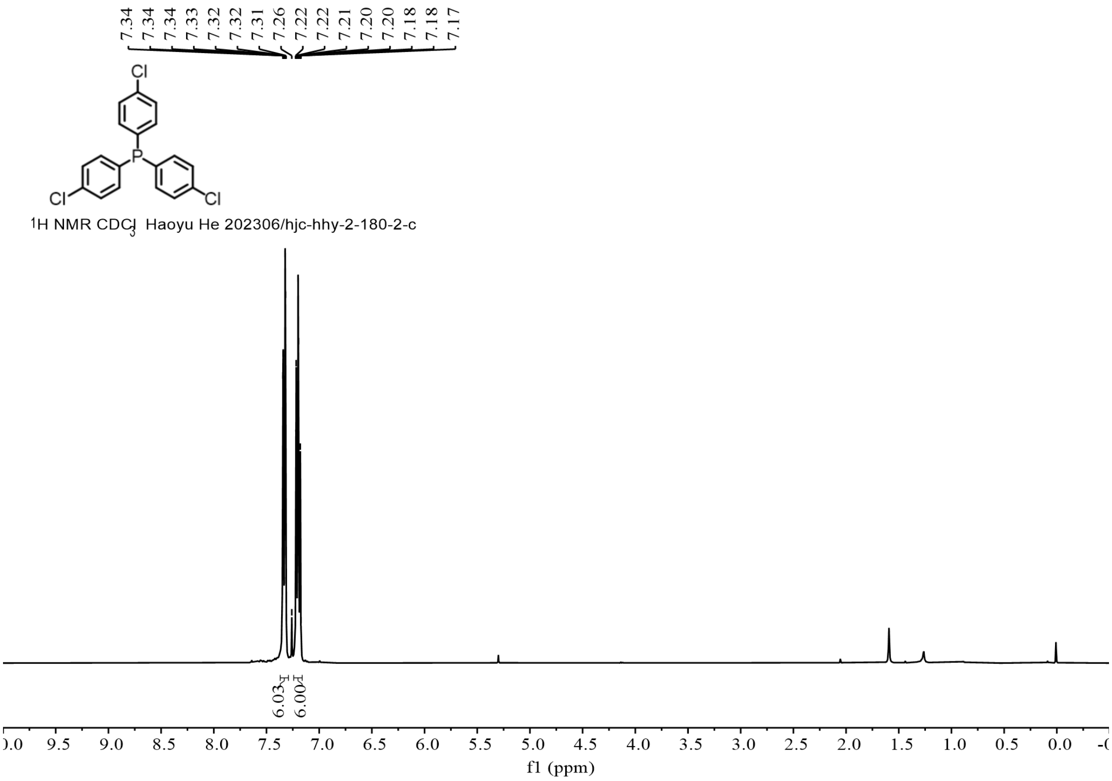

^{13}C NMR

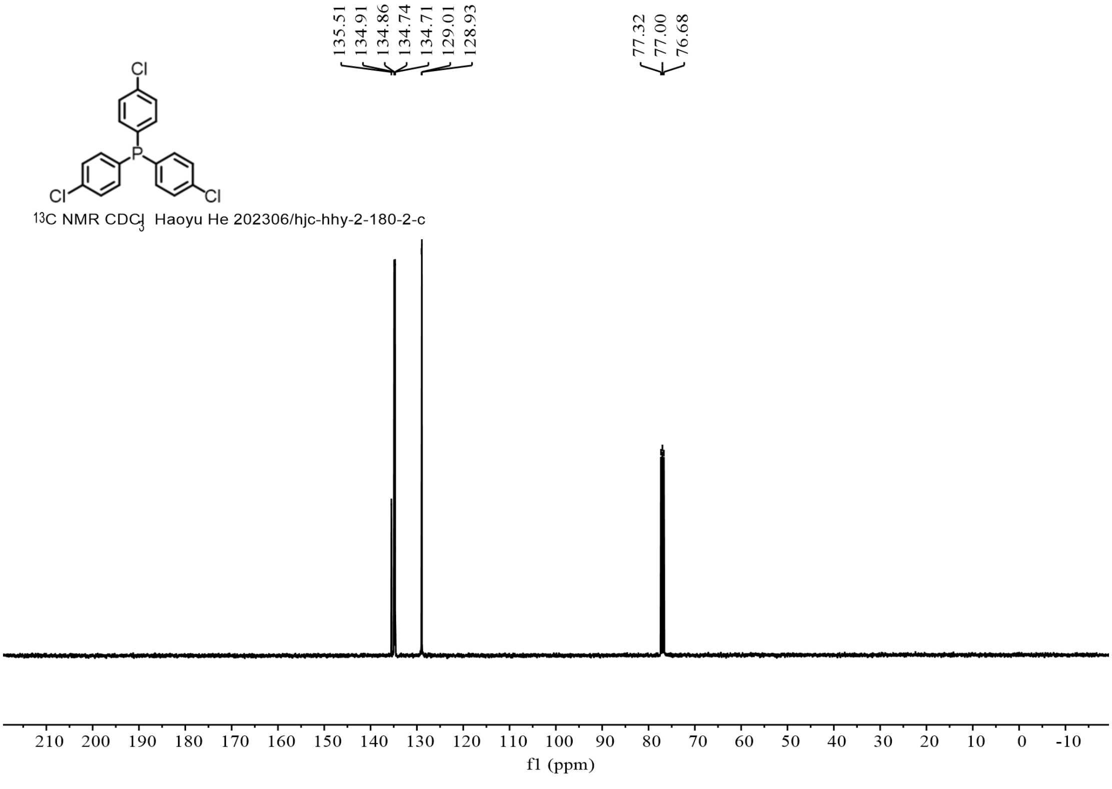

^{31}P NMR

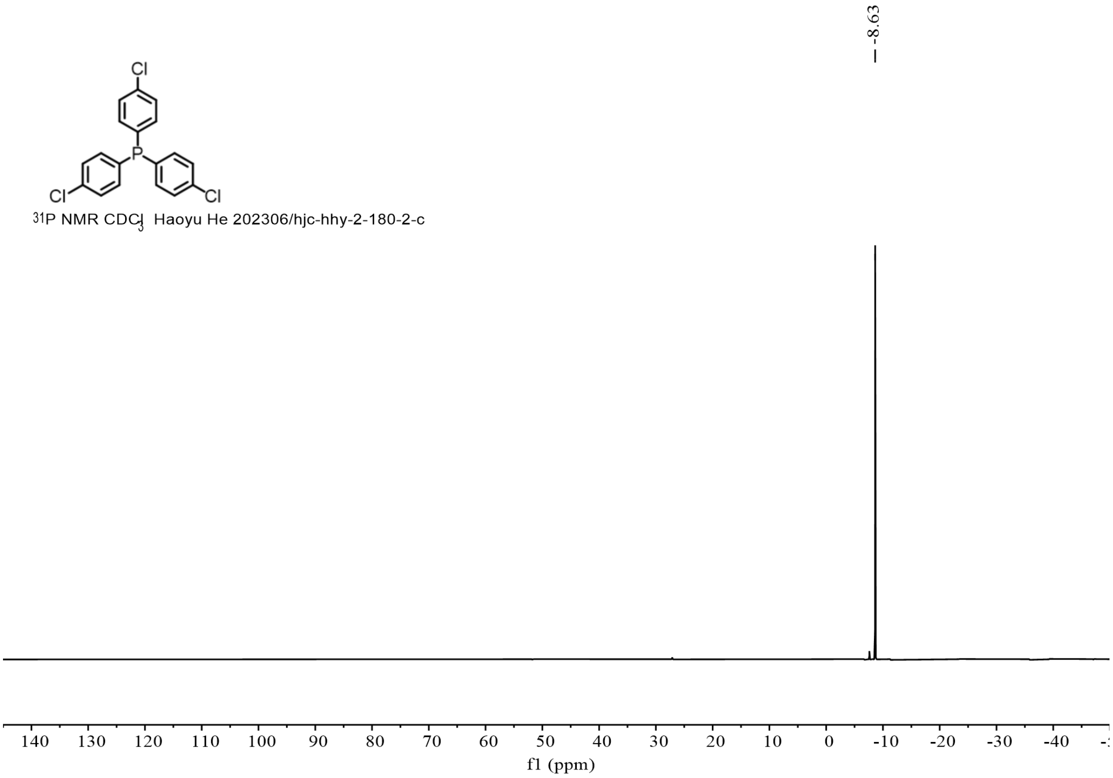

-8.63
Cl
P
Cl
Cl
31P NMR CDCl3 Haoyu He 202306/hjc-hhy-2-180-2-c
140
130
120
110
100
90
80
70
60
50
40
30
20
10
0
-10
-20
-30
-40
f1 (ppm)

50

^{1}H NMR

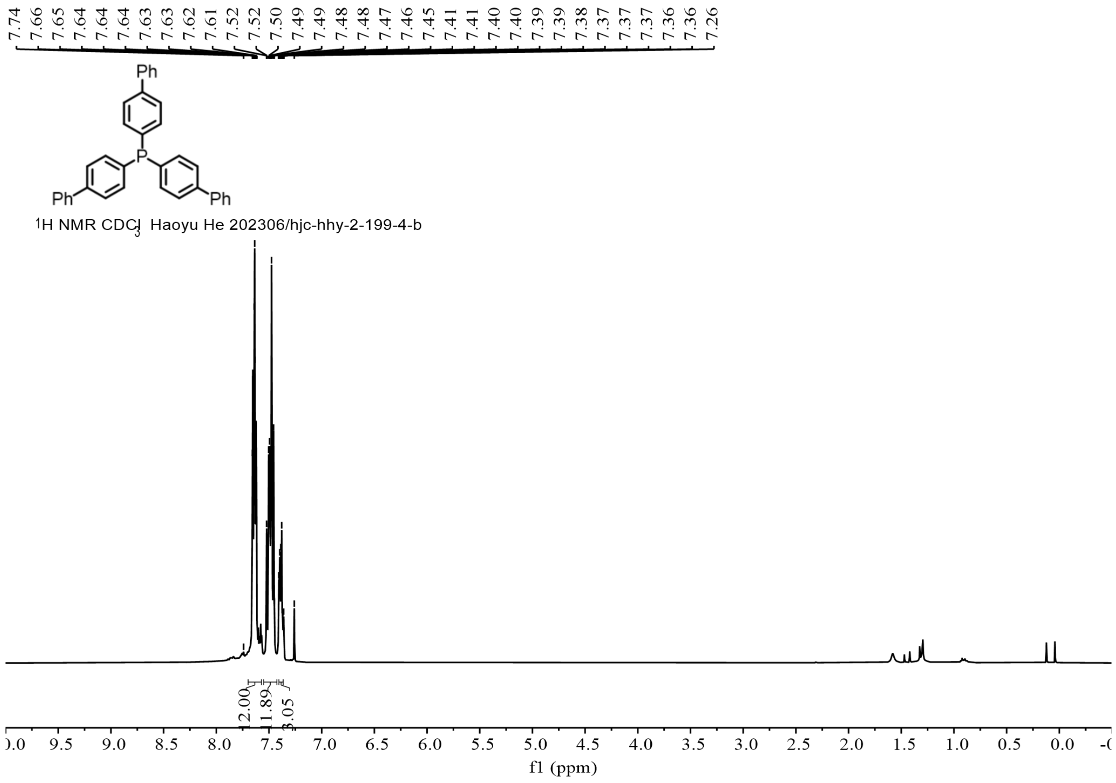

^{13}C NMR

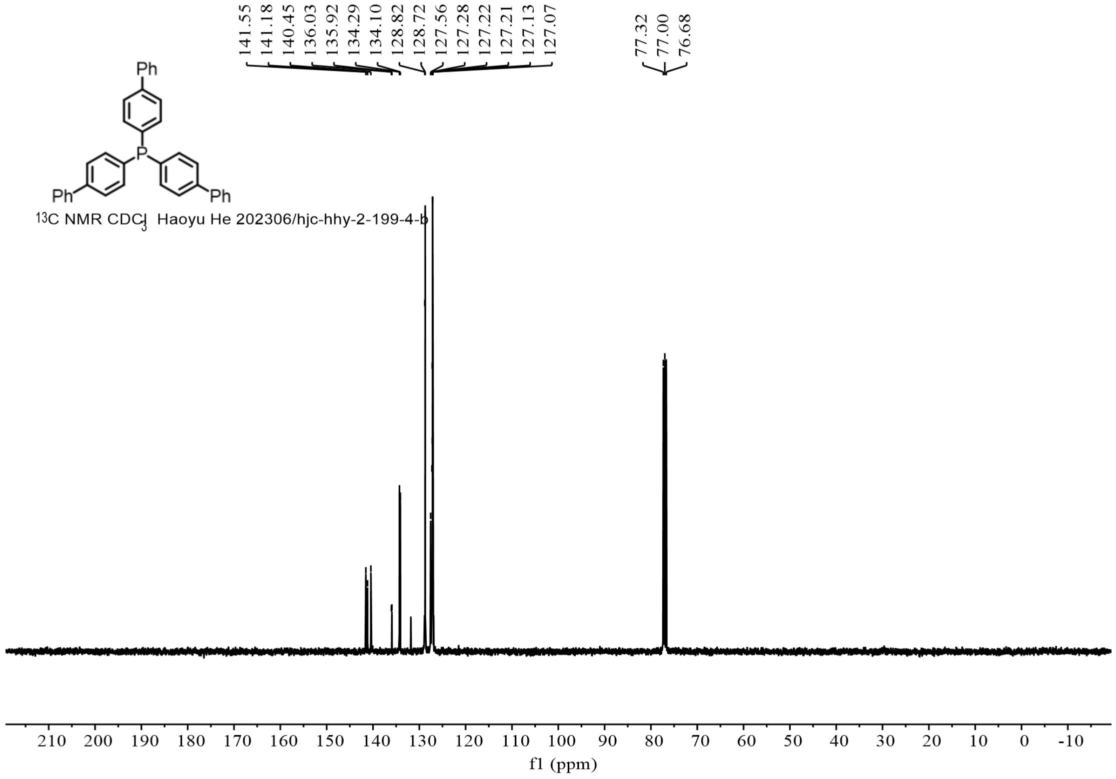

^{31}P NMR

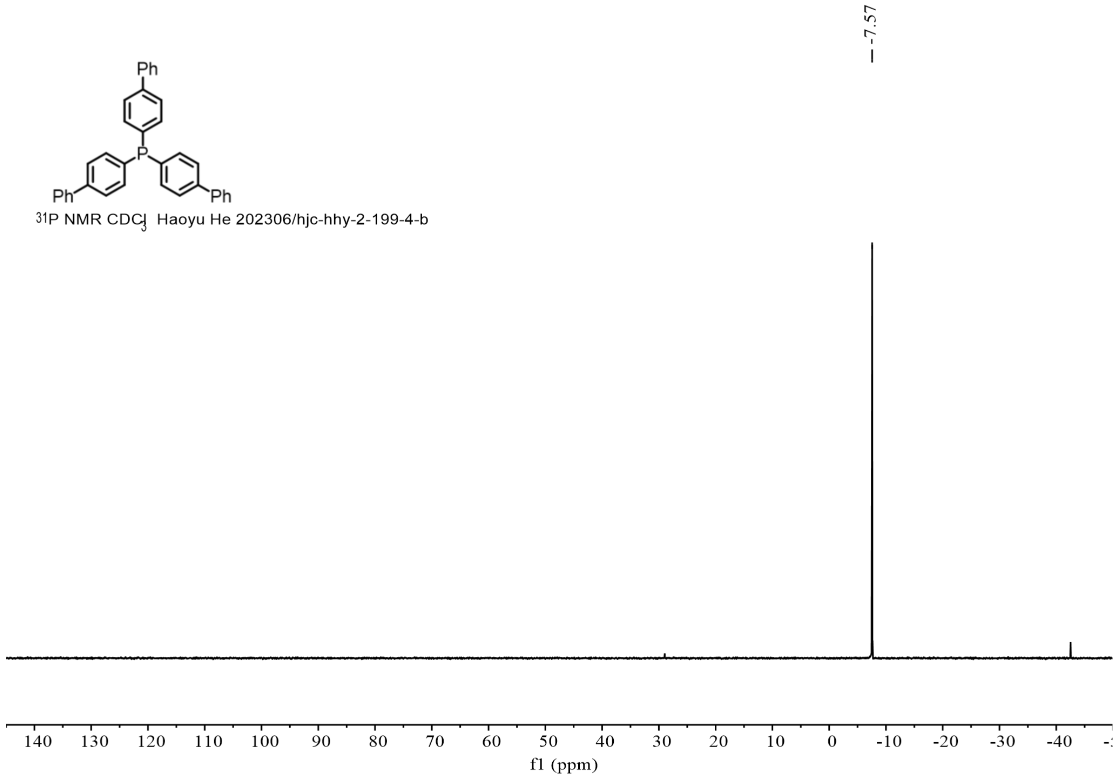

-7.57
Ph
P
Ph
Ph
31P NMR CDCl3 Haoyu He 202306/hjc-hhy-2-199-4-b
140 130 120 110 100 90 80 70 60 50 40 30 20 10 0 -10 -20 -30 -40
f1 (ppm)

51

^{1}H NMR

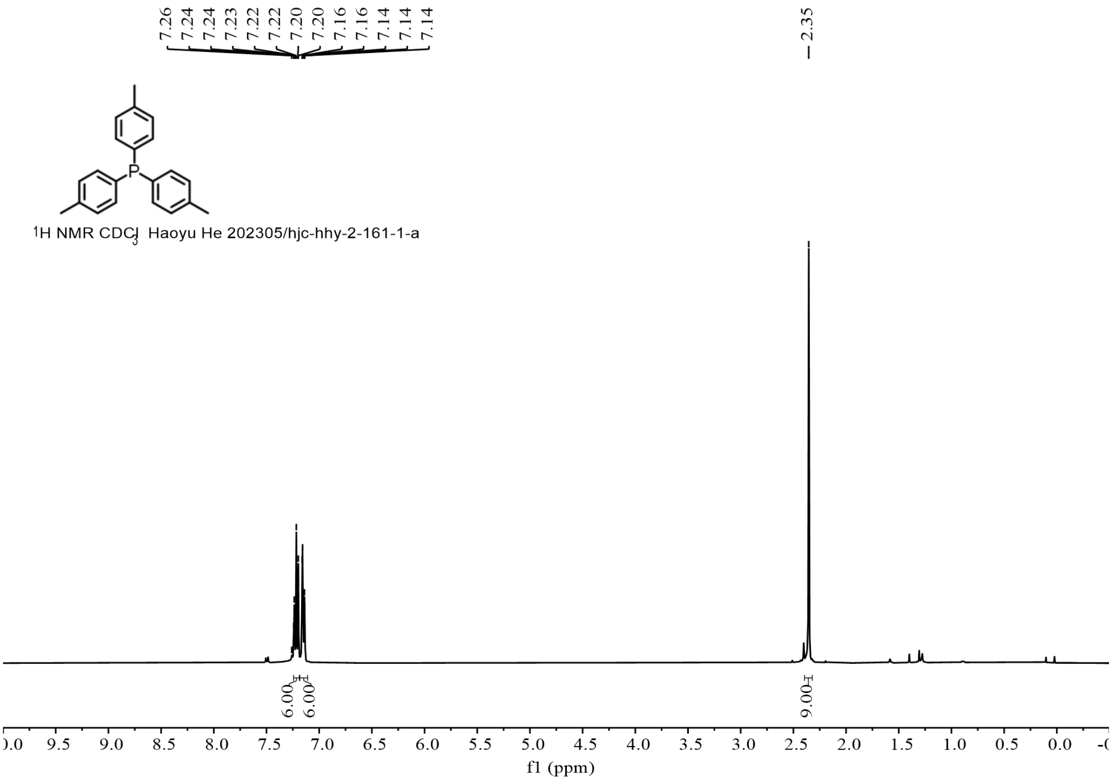

^{13}C NMR

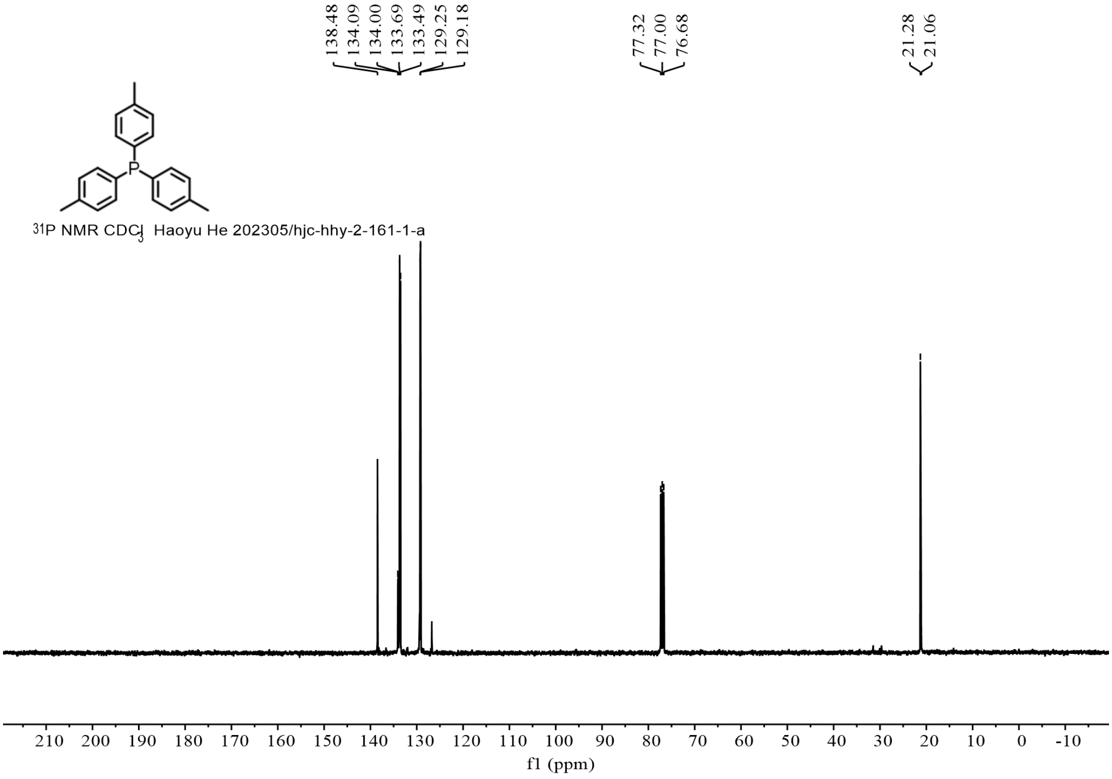

^{31}P NMR

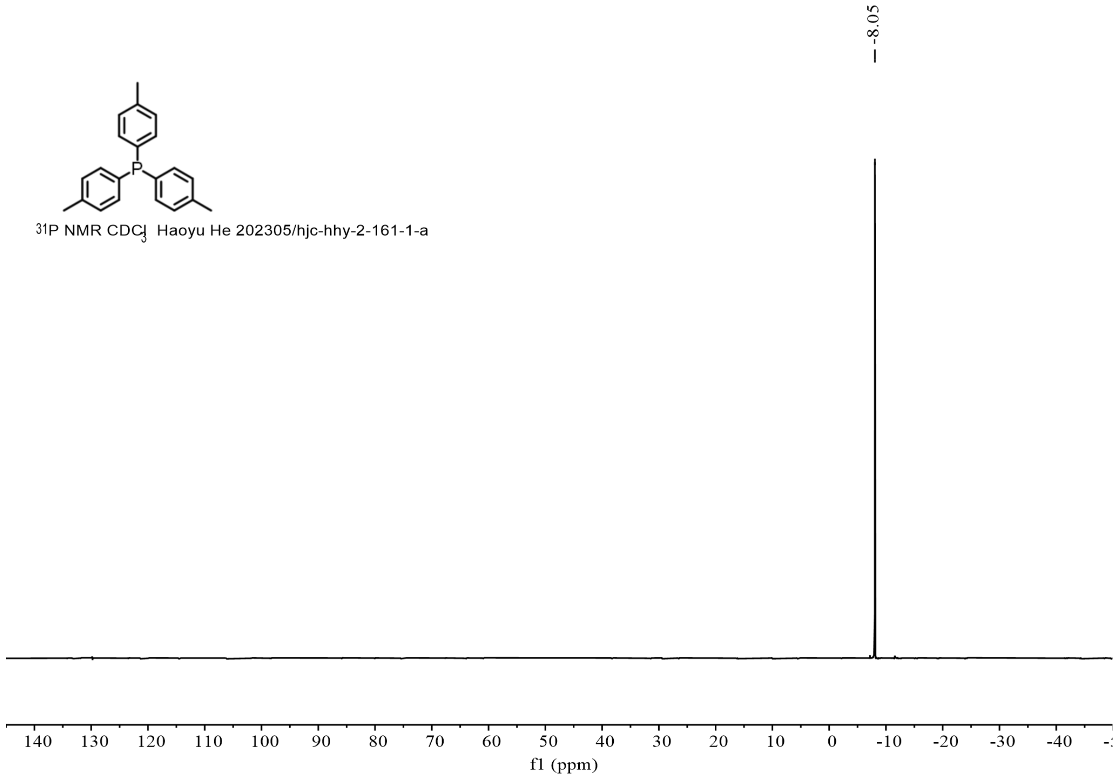

-8.05
31P NMR CDCl3 Haoyu He 202305/hjc-hhy-2-161-1-a
140
130
120
110
100
90
80
70
60
50
40
30
20
10
0
-10
-20
-30
-40
f1 (ppm)

52

^{1}H NMR

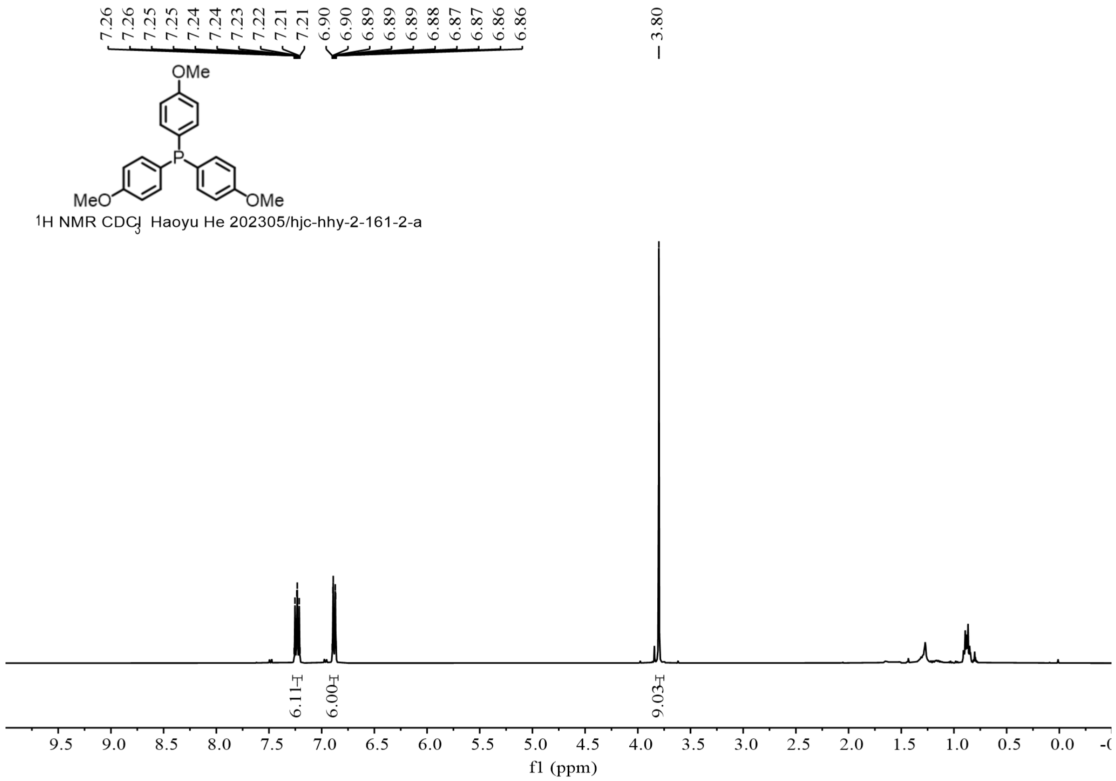

^{13}C NMR

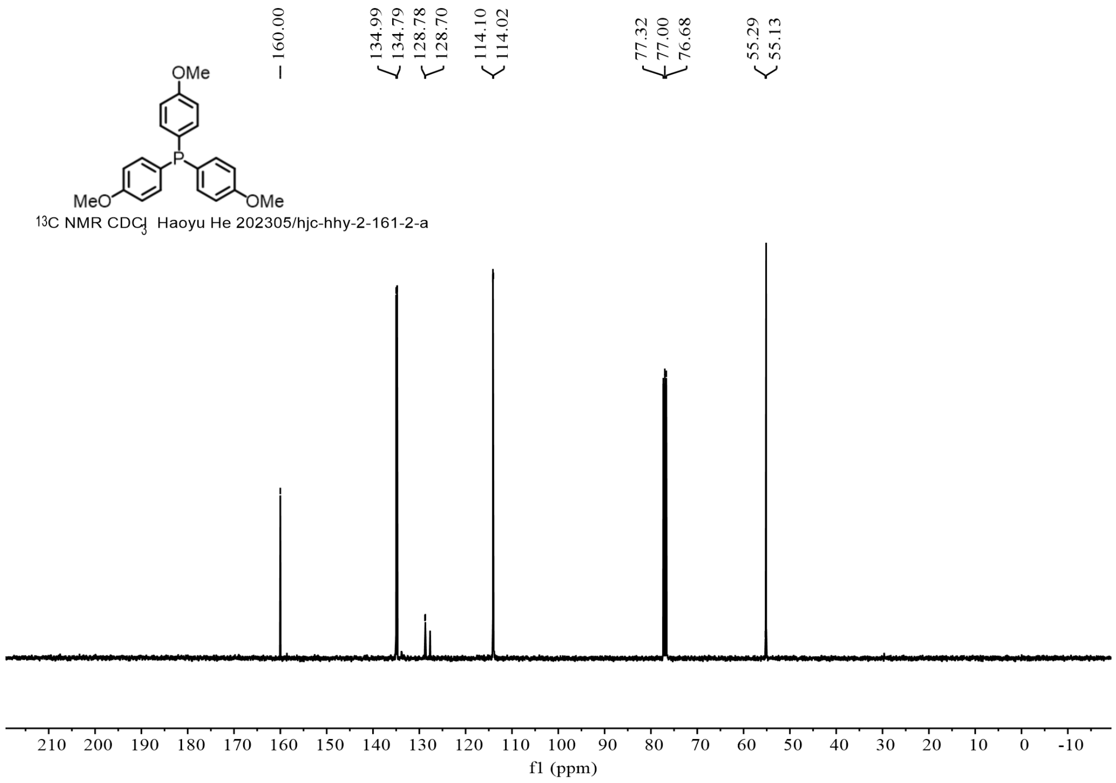

^{31}P NMR

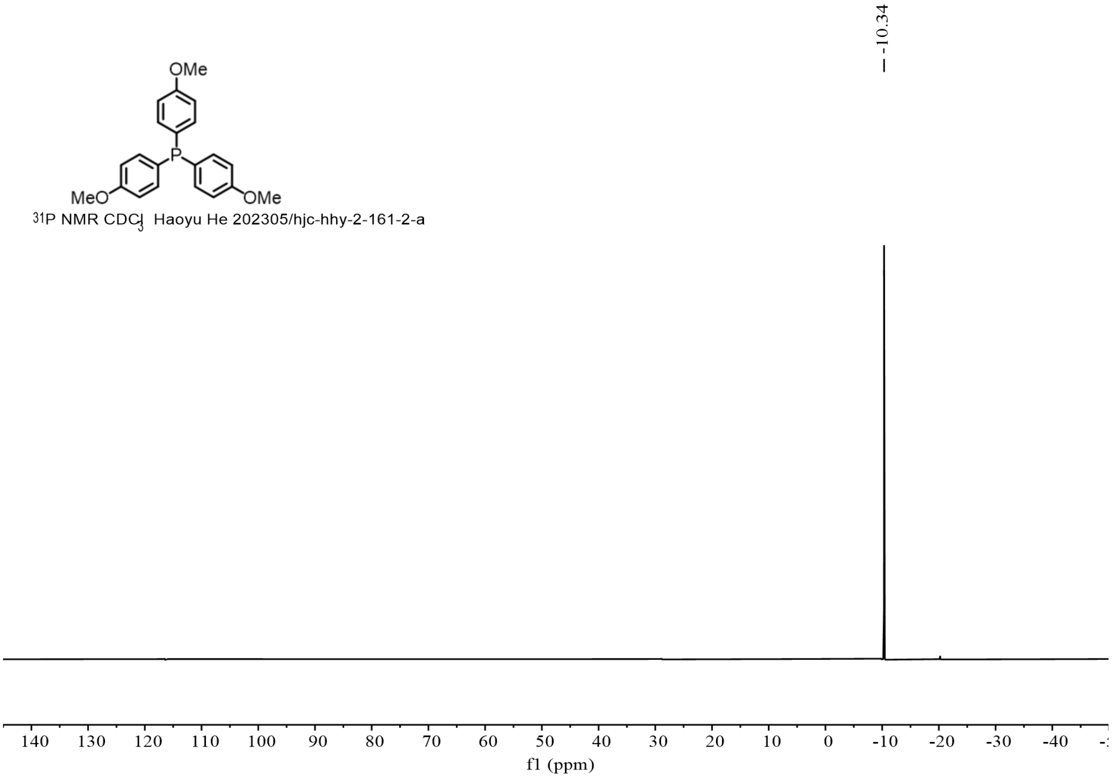

--10.34
OMe
P
MeO
OMe
31P NMR CDCl3 Haoyu He 202305/hjc-hhy-2-161-2-a
140
130
120
110
100
90
80
70
60
50
40
30
20
10
0
-10
-20
-30
-40
f1 (ppm)

53

^{1}H NMR

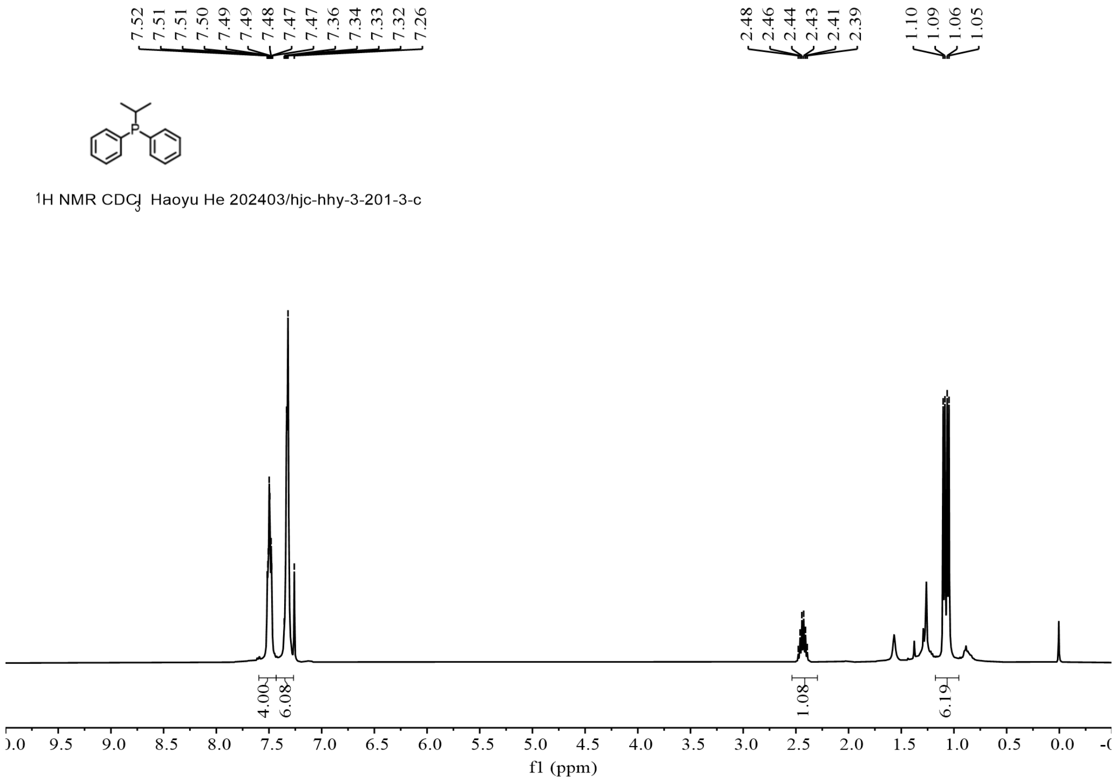

^{13}C NMR

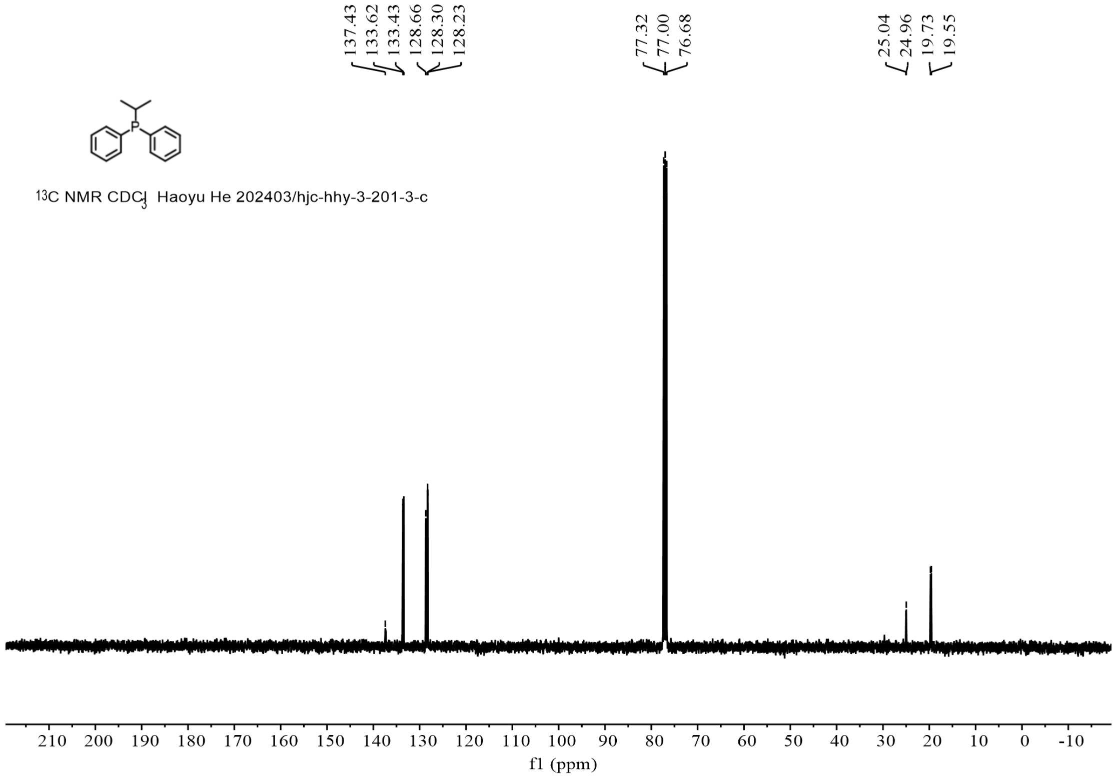

^{31}P NMR

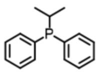

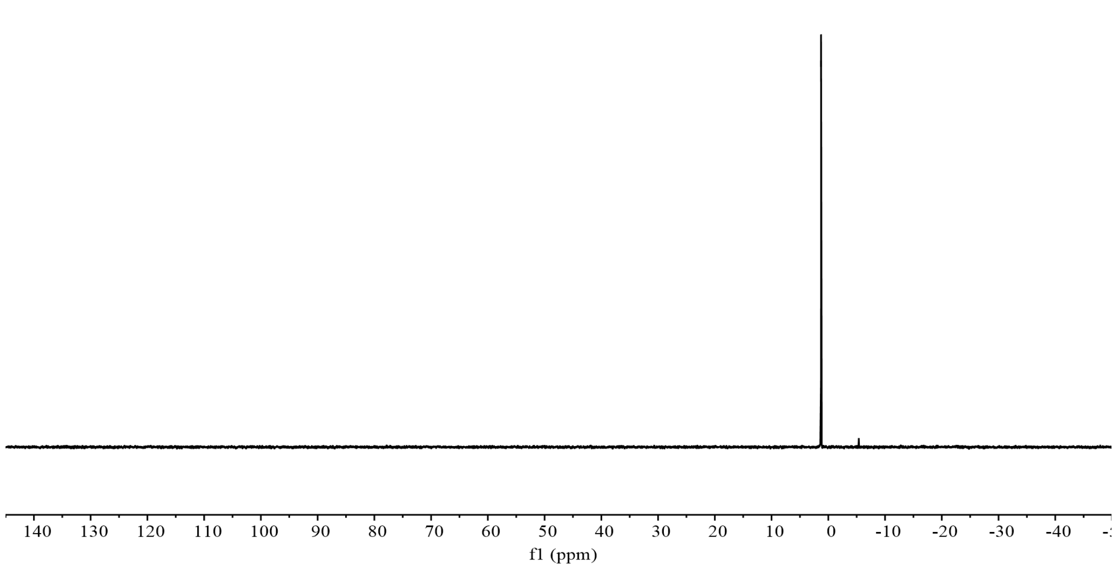

1.28
31P NMR CDCl3 Haoyu He 202403/hjc-hhy-3-201-3-c
140 130 120 110 100 90 80 70 60 50 40 30 20 10 0 -10 -20 -30 -40
f1 (ppm)

54

^{1}H NMR

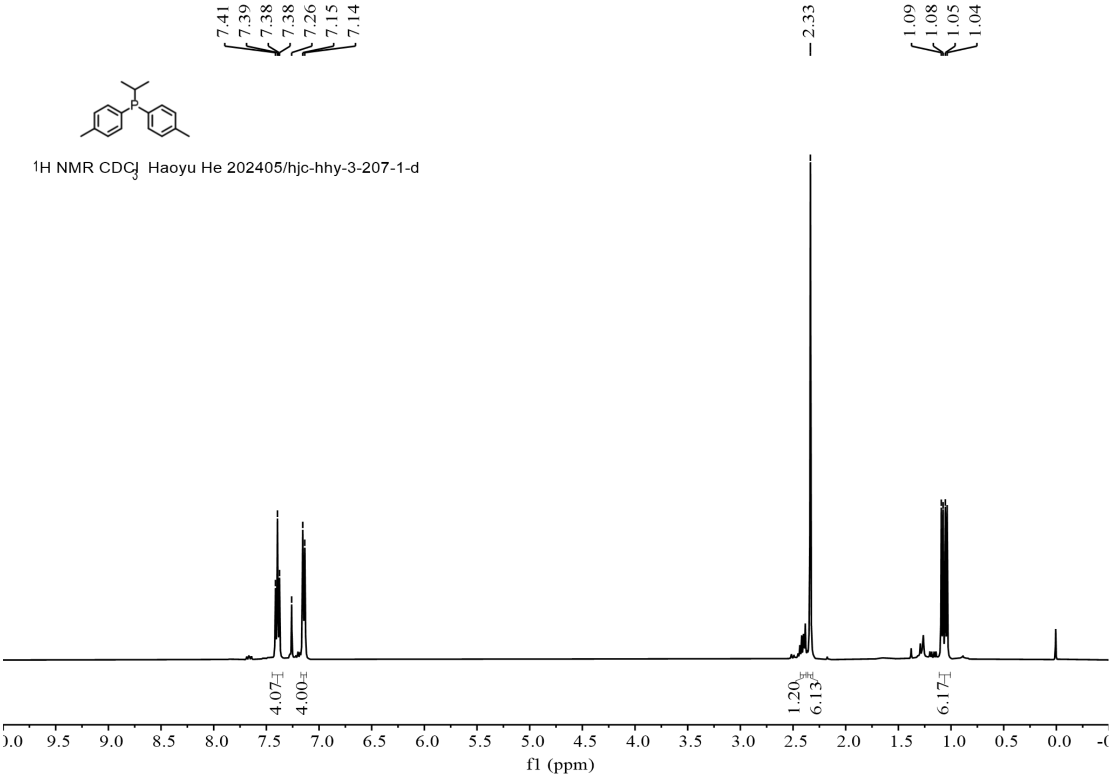

^{13}C NMR

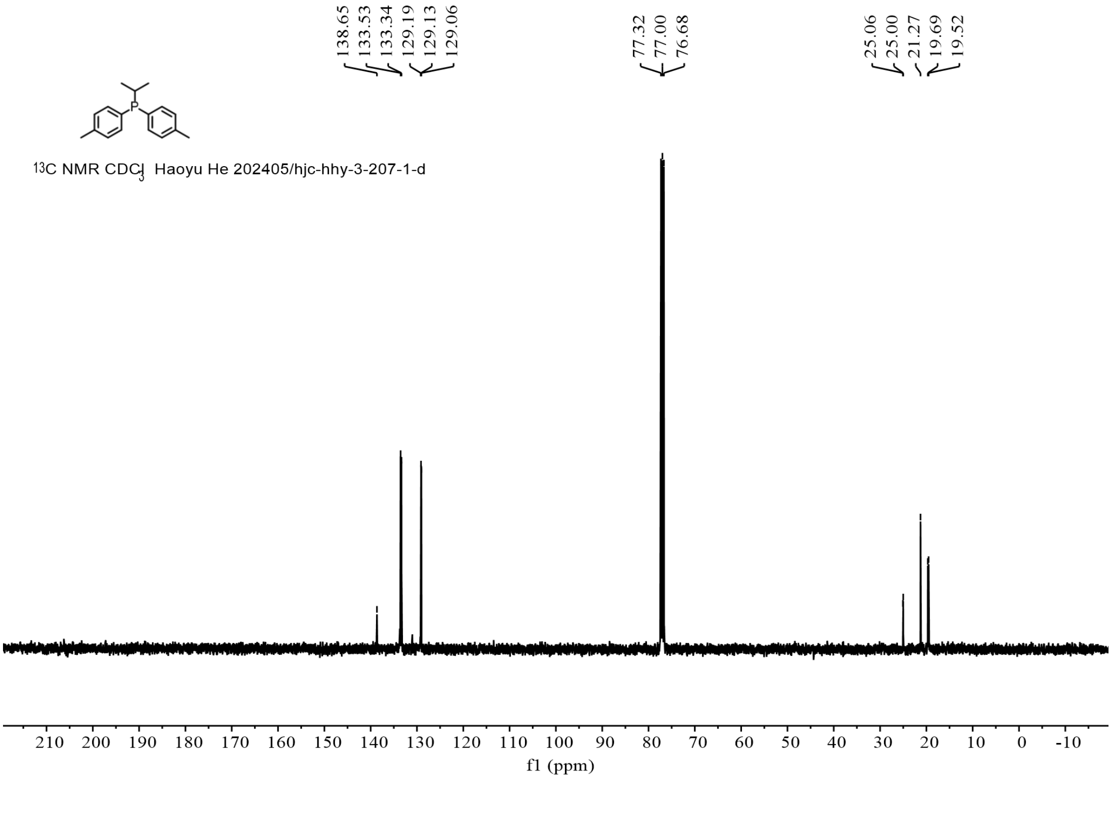

^{31}P NMR

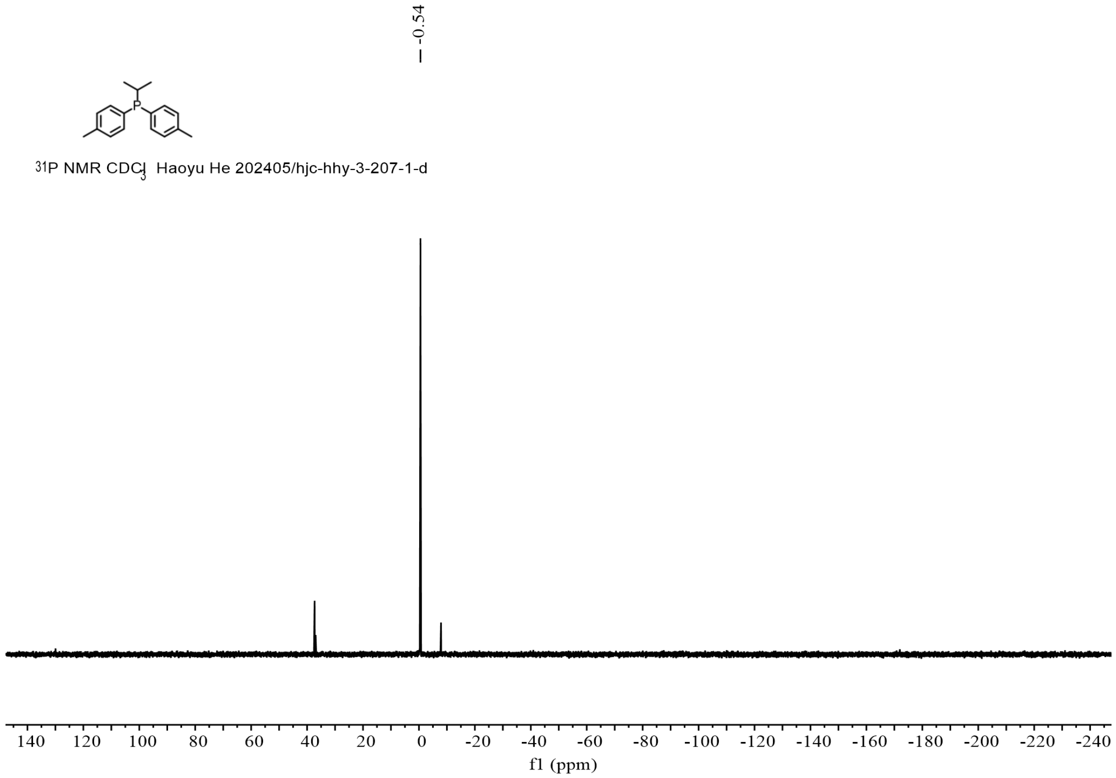
-0.54
31P NMR CDCl3 Haoyu He 202405/hjc-hhy-3-207-1-d
140 120 100 80 60 40 20 0 -20 -40 -60 -80 -100 -120 -140 -160 -180 -200 -220 -240
f1 (ppm)
